# Design, synthesis and antitumor activity of 5-trifluoromethylpyrimidine derivatives as EGFR inhibitors

**DOI:** 10.1080/14756366.2022.2128797

**Published:** 2022-09-29

**Authors:** Yaqing Zuo, Rongrong Li, Yan Zhang, Guochen Bao, Yi Le, Longjia Yan

**Affiliations:** a School of Pharmaceutical Sciences, Guizhou University, Guiyang, China; b Guizhou Engineering Laboratory for Synthetic Drugs, Guiyang, China; c Institute for Biomedical Materials and Devices (IBMD), Faculty of Science, University of Technology Sydney, Sydney, Australia

**Keywords:** EGFR, inhibitor, pyrimidine, antitumor, reagent

## Abstract

A new series of 5-trifluoromethylpyrimidine derivatives were designed and synthesised as EGFR inhibitors. Three tumour cells A549, MCF-7, PC-3 and EGFR kinase were employed to evaluate their biological activities. The results were shown that most of the target compounds existed excellent antitumor activities. In particular, the IC_50_ values of compound **9u** (*E*)-3-((2-((4-(3-(3-fluorophenyl)acrylamido)phenyl)amino)-5-(trifluoromethyl)pyrimidin-4-yl)amino)-*N*-methylthiophene-2-carboxamide against A549, MCF-7, PC-3 cells and EGFR kinase reached to 0.35 μM, 3.24 μM, 5.12 μM, and 0.091 μM, respectively. Additionally, further researches revealed that compound **9u** could induce early apoptosis of A549 cells and arrest the cells in G2/M phase. Taken together, these findings indicated that compound **9u** was potential for developing as antitumor reagent.

## Graphical Abstract

**Figure:**
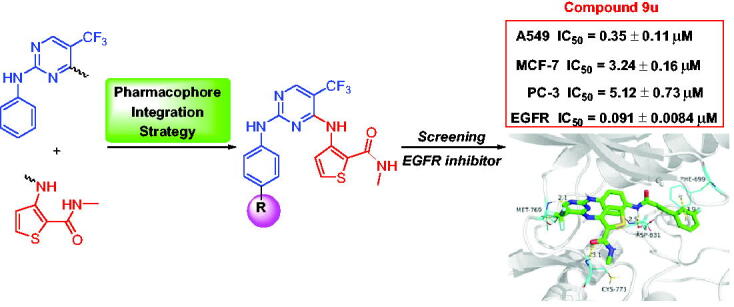

## Introduction

**Figure 1.**
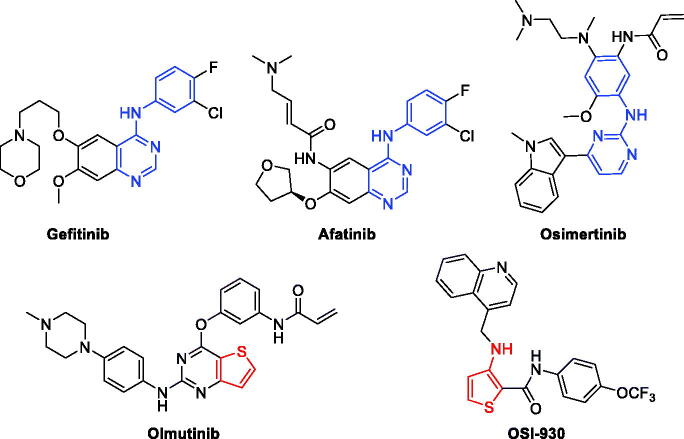
Structures of antitumor reagents in literature.

Epidermal growth factor receptor (EGFR) is widely distributed on the surface of epithelial cells, fibroblasts, glial cells, keratinocytes and other cells^1^. EGFR signalling pathway plays an important role in the physiological processes of cell growth, proliferation and differentiation^2^. Many researches have shown that EGFR is highly or abnormally expressed in many tumors^3^. EGFR is related to the inhibition of tumour cell proliferation, angiogenesis, tumour invasion, metastasis and apoptosis. Therefore, EGFR has become an important target for anti-tumour drug development^4^^,^^5^. At present, many EGFR inhibitors (Gefitinib, Afatinib, and Osimertinib in Figure 1) have been approved for treatment of various tumors^6^. However, the acquired mutations of EGFR therapeutic drugs in malignant tumours have seriously weakened the therapeutic effect, resulting in drug resistance and toxicity^7^^,^^8^. Therefore, discovery of novel EGFR inhibitors is of great significance for the treatment of malignant tumors^9–14^.

Thiophene derivatives exist broad activities in the field of pharmaceutical researches especially in anti-microbial, anti-tumour, anti-oxidation, and anti-inflammatory activity^15–17^. As shown in Figure 1, Olmutinib, a kind of thienopyrimidine compound was an important EGFR inhibitor in market to treat nonsmall-cell lung cancer (NSCLC)^18^. Moreover, OSI-930 in Figure 1 was a good anticancer reagent as kinase inhibitor in clinical trials for multiple tumors^19^. In addition, more and more thiophene derivatives were successfully developed as antitumor reagents^20–23^.

**Figure 2.**
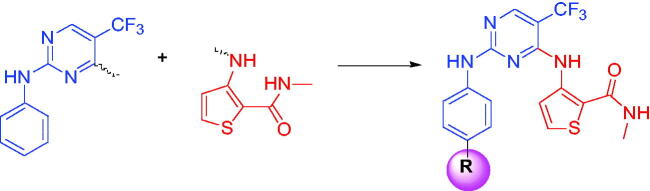
Designed strategy of target compounds.

The traditional pharmacophore integration strategy was usually adopted in design novel kinase inhibitors to discovery anticancer drugs^24–26^. Under this principle, we recently introduced 2-aminothiophene to phenylaminopyrimidine derivatives to discovery new EGFR inhibitors^27^. To obtain much more efficient EGFR inhibitors, 3-aminothiophene fragment was employed in our research. According to the excellent antitumor activities of 3-aminothiophene derivatives as kinase inhibitors, 5-trifluoromethyl-2-phenylpyrimidine (Blue part in Figure 2) was the main skeleton and 3-aminothiophene-2-carboxylic acid methylamide (Red part in Figure 2) was introduced into the 4-position of pyrimidine ring. In this paper, we will synthesis this novel series of 5-trifluoromethylpyrimidines derivatives and evaluate their antitumor activities as EGFR inhibitors.

## Experimental section

### Synthesis

#### Synthesis of 3-amino-thiophene-2-carboxylic acid methyl ester (1)

Methyl thioglycolate (10.6 g, 100 mmol) and NaOCH_3_ (10.8 g, 200 mmol) were dissolved in MeOH (90 mL). Then, 2-chloroacrylonitrile (8.75 g, 100 mmol) in MeOH (10 mL) solution was added dropwise into the mixture at 0 °C. The reaction mixture was stirred at room temperature for 1 h. After completion of the reaction, the solution was concentrated under vacuum, and H_2_O (50 mL) was added. The mixture was extracted with EtOAc (4 × 50 mL), washed with saturated NaCl solution, dried over Na_2_SO_4_, filtered and concentrated under vacuum. The residue was recrystallized from petroleum ether to give a yellow solid (8.95 g, 57%). mp: 42.3–44.8 °C; ^1^H NMR (400 MHz, DMSO-*d_6_*) δ 7.55 (d, *J* = 5.6 Hz, 1H), 6.62 (d, *J* = 5.6 Hz, 1H), 6.53 (s, 2H), 3.71 (s, 3H); ^13^C NMR (100 MHz, DMSO-*d_6_*) δ 164.6, 155.7, 132.8, 120.7, 97.7, 51.3. Spectral properties were in accordance with the literature^28^.

#### Synthesis of 3-tert-butoxycarbonylamino-thiophene-2-carboxylic acid methyl ester (2)

A mixture of compound **1** (1572 mg, 10 mmol), DMAP (61 mg, 0.5 mmol) and DCM (16 mL) was sequentially added di-tert-butyl dicarbonate (1572 mg, 10 mmol) and DIEA (1935 mg, 15 mmol). The solution was stirred at 40 °C for 4 h. The reaction mixture was evaporated under vacuum, and the residue was purified by silica gel column using PE/EA = 20/L as fluent solvents to obtain a white solid (1.28 g, 50%). mp: 83.0–84.7 °C; ^1^H NMR (400 MHz, DMSO-*d*_6_) δ 9.28 (s, 1H), 7.90 (d, *J* = 5.6 Hz, 1H), 7.74 (d, *J* = 5.6 Hz, 1H), 3.82 (s, 3H), 1.49 (s, 9H); ^13^C NMR (100 MHz, DMSO-*d*_6_) δ 164.4, 151.7, 145.2, 133.9, 121.2, 108.4, 81.5, 52.5, 28.3. Spectral properties were in accordance with the literature^29^.

#### Synthesis of 3-tert-butoxycarbonylamino-thiophene-2-carboxylic acid (3)

Compound **2** (2158 mg, 8.40 mmol) was dissolved in THF (10 mL). And then, NaOH solution (8.2 mL, 4 mol/L, 33.60 mmol) was added. The mixture was stirred at 70 °C for 12 h. After the reaction finished, the solution was cooled to room temperature and HCl (4 M) was added to adjust the pH to 2. The solid was filtered, washed with water, and dried to obtain as a white solid (1.98 g, 97%). mp: 152.1–157.2 °C; ^1^H NMR (400 MHz, DMSO-*d_6_*) δ 13.44 (s, 1H), 9.44 (s, 1H), 7.84 (d, *J* = 5.6 Hz, 1H), 7.74 (d, *J* = 5.6 Hz, 1H), 1.49 (s, 9H); ^13^C NMR (100 MHz, DMSO-*d_6_*) δ 165.7, 151.7, 144.7, 133.0, 121.0, 109.7, 81.3, 28.3. Spectral properties were in accordance with the literature^29^.

#### Synthesis of (2-methylcarbamoyl-thiophen-3-yl)-carbamic acid tert-butyl ester (4)

A mixture of compound **3** (1970 mg, 8.11 mmol) and methylamine hydrochloride (2737 mg, 40.55 mmol) was stirred in DMF (5 mL) at room temperature. Then, HATU (4625 mg, 12.17 mmol) was added into the solution and DIEA (5231 mg, 40.55 mmol) was added dropwise. The mixture was stirred at room temperature for 16 h. After the reaction finished, H_2_O (30 mL) was added and extracted with EtOAc (3 × 30 mL). The organic phase was washed with saturated NaCl, dried over Na_2_SO_4_, filtered and concentrated under vacuum. The crude product was purified by recrystallization through MeOH to give a white solid (2.162 g, 96%). mp: 107.9–109.3 °C; ^1^H NMR (400 MHz, DMSO-*d_6_*) δ 10.39 (s, 1H), 8.16 (q, *J* = 4.8 Hz, 1H), 7.72 (d, *J* = 5.6 Hz, 1H), 7.68 (d, *J* = 5.6 Hz, 1H), 2.73 (d, *J* = 4.4 Hz, 3H), 1.47 (s, 9H); ^13^C NMR (100 MHz, DMSO-*d_6_*) δ 164.6, 152.0, 143.2, 129.2, 121.2, 111.6, 80.7, 28.4, 26.4; ESI-HRMS C_11_H_8_ClF_3_N_4_OS ([M**+**Na]^+^) calcd 277.0774, found 277.0773.

#### Synthesis of 3-amino-thiophene-2-carboxylic acid methylamide (5)

A mixture of compound **4** (2 g, 7.81 mmol) and HCl (20 mL, 2 mol/L ethyl acetate solution) was stirred at room temperature for 24 h. After the reaction completed, the white solid was precipitated and dissolved in water (18 mL). The pH was adjusted to 9–10 with saturated sodium bicarbonate, resulting in a large amount of white solid. The reaction mixture was extracted with DCM (4 × 30 mL). The organic phase was dried over Na_2_SO_4_, filtered and concentrated under vacuum to give the compound as a brown solid (0.903 g, 74%). mp: 99.8–101.7 °C; ^1^H NMR (400 MHz, DMSO-*d_6_*) δ 7.36 (t, *J* = 5.6 Hz, 1H), 6.58 (d, *J* = 5.2 Hz, 1H), 6.38 (s, 1H), 2.67 (d, *J* = 4.4 Hz, 3H); ^13^C NMR (100 MHz, DMSO-*d_6_*) δ 165.5, 153.3, 127.9, 121.4, 101.9, 26.3; ESI-HRMS C_6_H_8_N_2_OS ([M + Na]^+^): calcd 179.0229, found 179.0246.

#### Synthesis of 3-(2-chloro-5-trifluoromethyl-pyrimidin-4-ylamino)-thiophene-2-carboxylic acid methylamide (6)

A mixture of 2,4-dichloro-5-trifluoromethylpyrimidine (1.08 g, 5 mmol) and compound **5** (795 mg, 5.5 mmol) was stirred in DMF (10 mL) at room temperature. Then the solution was added sodium hydride (555 mg, 25 mmol) at 0 °C and stirred overnight. After the reaction completed, H_2_O (30 mL) was carefully added, and the mixture was extracted with EtOAc (3 × 30 mL). The organic phase was washed with saturated NaCl, dried over Na_2_SO_4_, filtered and concentrated under vacuum. The residue was purified by silica gel column using PE/EA = 2/1 to give a white solid (584 mg, 37%). mp:192.5–193.3 °C; ^1^H NMR (400 MHz, DMSO-*d_6_*) δ 12.30 (s, 1H), 8.71 (d, *J* = 1.2 Hz, 1H), 8.45 (q, *J* = 4.4 Hz, 1H), 8.17 (d, *J* = 5.6 Hz, 1H), 7.85 (d, *J =* 5.6 Hz, 1H), 2.78 (d, *J* = 4.8 Hz, 3H); ^13^C NMR (100 MHz, DMSO-*d_6_*) δ 164.7, 162.8, 157.3, 155.4, 141.7, 129.4, 125.0, 123.1, 122.3, 115.3, 107.2 (d, *J* = 32.4 Hz), 26.6; ESI-HRMS C_11_H_8_ClF_3_N_4_OS ([M **+** H]^+^) calcd 337.0132, found 337.0127.

#### Synthesis of 3-[2-(4-nitro-phenylamino)-5-trifluoromethyl-pyrimidin-4-ylamino]-thiophene-2-carboxylic acid methylamide (7)

A mixture of compound **6** (650 mg, 1.93 mmol), p-nitroaniline (320 mg, 2.32 mmol), and trifluoroethanol (7 mL) was carefully added trifluoroacetic acid (660.18 mg, 5.79 mmol). The solution was stirred at 80 °C for overnight under argon atmosphere. After the reaction completed, the mixture was cooled to room temperature, extracted with EtOAc (3 × 30 mL), and washed with saturated NaHCO_3_ solution. The organic phase was dried over Na_2_SO_4._ After filtration, it was concentrated under reduced pressure, slurried with MeOH, and the filter cake was dried to obtain a yellow solid (0.7 g, 83%). mp: 230.4–231.2 °C; ^1^H NMR (400 MHz, DMSO-*d_6_*) δ 11.80 (s, 1H), 10.52 (s, 1H), 8.58 (s, 1H), 8.46 (d, *J* = 5.6 Hz, 1H), 8.31 (q, *J* = 4.4 Hz, 1H), 8.27 − 8.19 (m, 2H), 8.04 − 7.97 (m, 2H), 7.83 (d, *J* = 5.6 Hz, 1H), 2.78 (d, *J* = 4.4 Hz, 3H)； ^13^C NMR (100 MHz, DMSO-*d_6_*) δ 164.8, 160.8, 156.3 (d, *J* = 5.6 Hz), 155.1, 155.0, 146.7, 142.6, 141.7, 128.7, 126.0, 125.3, 124.3, 119.4, 114.1, 101.2 (d, *J* = 31.6 Hz), 26.6; ESI-HRMS C_17_H_13_F_3_N_6_O_3_S ([M **+** H]^+^) calcd 439.0794, found 439.0791.

#### Synthesis of 3-[2-(4-amino-phenylamino)-5-trifluoromethyl-pyrimidin-4-ylamino]-thiophene-2-carboxylic acid methylamide (8)

A mixture of compound **7** (700 mg) and palladium on carbon (70 mg) was stirred in MeOH (7 mL). The solution was stirred at room temperature for overnight under hydrogen atmosphere. After the reaction completed, the reaction solution was filtered in celite. The filtration was concentrated under vacuum, and dryness to give a yellow solid (450 mg, 69%). mp: 227.0–227.8 °C; ^1^H NMR (400 MHz, DMSO-*d_6_*) δ 11.57 (s, 1H), 9.42 (s, 1H), 8.66 (s, 1H), 8.34 (s, 1H), 8.20 (s, 1H), 7.60 (d, *J* = 68.0 Hz, 1H), 7.20 (s, 2H), 6.57 (d, *J* = 8.4 Hz, 2H), 4.92 (s, 2H), 2.85 − 2.68 (m, 3H); ^13^C NMR (100 MHz, DMSO-*d_6_*) δ 164.9, 164.1, 160.9, 160.0, 156.4, 143.3, 128.1, 125.3, 124.4, 122.3, 114.3, 113.1, 112.0, 110.0, 26.5; ESI-HRMS C_17_H_15_F_3_N_6_OS ([M + H]^+^) calcd 409.1052, found 409.1044.

#### Synthesis of 9a-9x

A mixture of compound **8** (150 mg, 0.37 mmol) and DIEA (72.24 mg, 0.56 mmol) was stirred in DMF (4 mL). And then, the solution was added corresponding carboxylic acid (0.37 mmol) and HATU (212.8 mg, 0.56 mmol). The mixture was stirred at room for 24 h under nitrogen atmosphere. After the reaction completed, the mixture was extracted with EtOAc (30 mL × 3). The combined organic phases were washed with saturated NaCl (30 mL × 3), dried over Na_2_SO_4,_ filtered and concentrated in vacuum to give the crude compound. The crude product purified by slurring with methanol to give the products **9a–9x**.

##### 3-[2-(4-Benzoylamino-phenylamino)-5-trifluoromethyl-pyrimidin-4-ylamino]-thiophene-2-carboxylic acid methylamide (9a)

Yellow solid; 37% yield; mp: 212.5-213.8 °C; ^1^H NMR (400 MHz, DMSO-*d*_6_) δ 11.65 (s, 1H), 10.19 (s, 1H), 9.84 (s, 1H), 8.44 (s, 1H), 8.24 (s, 1H), 7.67 (s, 3H), 7.64 (s, 3H), 7.58 (s, 1H), 7.48 − 7.43 (m, 4H), 6.85 (d, *J* = 12.0 Hz, 2H), 2.80 − 2.75 (m, 3H); ^13^C NMR (100 MHz, DMSO-*d*_6_) δ 164.9, 163.7, 161.7, 161.6, 161.3, 160.7, 156.4, 155.1, 143.0, 142.9, 140.3, 135.4, 135.3, 130.2, 129.5, 128.2, 124.4, 122.9, 120.1 (d, *J* = 9.8 Hz), 113.4, 92.7, 26.5; ESI-HRMS C_24_H_19_F_3_N_6_O_2_S ([M**+**Na]^+^) calcd 535.1134, found 535.1135. Anal. calcd for C_24_H_19_F_3_N_6_O_2_S: C 56.25, H 3.74, N 16.40; found C 56.24, H 3.71, N 16.41.

##### 3-{2-[4-(2-Fluoro-benzoylamino)-phenylamino]-5-trifluoromethyl-pyrimidin-4-ylamino}-thiophene-2-carboxylic acid methylamide (9b)

Light yellow solid; 51% yield; mp: 213.3–215.4 °C; ^1^H NMR (400 MHz, DMSO-*d*_6_) δ 11.67 (s, 1H), 10.38 (s, 1H), 9.88 (s, 1H), 8.45 (s, 1H), 8.26 (q, *J* = 4.8 Hz, 1H), 7.69 (d, *J* = 8.8 Hz, 4H), 7.66 − 7.54 (m, 4H), 7.35 (q, *J* = 8.0, 7.2 Hz, 2H), 2.77 (d, *J* = 4.4 Hz, 3H)；^13^C NMR (100 MHz, DMSO-*d*_6_) δ 164.9, 162.9, 161.5, 160.6, 158.1, 156.4 (d, *J* = 4.9 Hz), 155.1, 143.0, 135.7, 134.7, 132.9, 130.4 (d, *J* = 3.2 Hz), 128.3, 125.6 (d, *J* = 15.2 Hz), 125.5, 125.0 (d, *J* = 3.6 Hz), 124.4, 123.7, 120.6, 116.6 (d, *J* = 21.6 Hz), 113.5, 26.5; ESI-HRMS C_24_H_18_F_4_N_6_O_2_S ([M **+** H]^+^) calcd 531.1220, found 531.1218. Anal. calcd for C_24_H_18_F_4_N_6_O_2_S: C 54.34, H 3.42, N 15.84; found C 54.33, H 3.40, N 15.86.

##### 3-{2-[4–(3-Fluoro-benzoylamino)-phenylamino]-5-trifluoromethyl-pyrimidin-4-ylamino}-thiophene-2-carboxylic acid methylamide (9c)

Light yellow solid; 46% yield; mp: 239.8–241.5 °C; ^1^H NMR (400 MHz, DMSO-*d*_6_) δ 11.66 (s, 1H), 10.31 (s, 1H), 9.88 (s, 1H), 8.46 (s, 1H), 8.26 (q, *J* = 4.8 Hz, 1H), 7.84 (dt, *J* = 8.0, 1.2 Hz, 1H), 7.79 (dt, *J* = 10.0, 2.0 Hz, 1H), 7.74 (d, *J* = 2.0 Hz, 1H), 7.72 (s, 2H), 7.67 − 7.57 (m, 3H), 7.48 − 7.43 (m, 1H), 2.77 (d, *J* = 4.8 Hz, 3H); ^13^C NMR (100 MHz, DMSO-*d*_6_) δ 164.3, 164.3, 163.6, 161.5, 161.2, 156.4, 155.1, 143.0, 137.8 (d, *J* = 6.8 Hz), 135.8, 131.0 (d, *J* = 8.0 Hz), 128.3, 126.4, 124.4, 124.3, 121.4, 118.8 (d, *J* = 21.2 Hz), 114.9 (d, *J* = 22.8 Hz), 113.5, 26.5; ESI-HRMS C_24_H_18_F_4_N_6_O_2_S ([M **+** H]^+^) calcd 531.1220, found 531.1223. Anal. calcd for C_24_H_18_F_4_N_6_O_2_S: C 54.34, H 3.42, N 15.84; found C 54.31, H 3.45, N 15.80.

##### 3-{2-[4-(4-Fluoro-benzoylamino)-phenylamino]-5-trifluoromethyl-pyrimidin-4-ylamino}-thiophene-2-carboxylic acid methylamide (9d)

Light yellow solid; 36% yield; mp: 217.2–219.3 °C; ^1^H NMR (400 MHz, DMSO-*d*_6_) δ 11.66 (s, 1H), 10.25 (s, 1H), 9.87 (s, 1H), 8.45 (s, 1H), 8.26 (q, *J* = 4.4 Hz, 1H), 8.09 − 8.03 (m, 2H), 7.75 − 7.70 (m, 3H), 7.64 (s, 2H), 7.43 − 7.34 (m, 2H), 2.77 (d, *J* = 4.4 Hz, 3H); ^13^C NMR (100 MHz, DMSO-*d*_6_) δ 165.7, 164.8 (d, *J* = 28.0 Hz), 163.2, 161. 6, 156.4, 155.1, 143.0, 135.6, 134.8, 131.9, 130.8 (d, *J* = 9.2 Hz), 129.1, 128.3, 126.4, 124.4, 123.7, 121.3, 115.8 (d, *J* = 21.6 Hz), 113.5, 26.5; ESI-HRMS C_24_H_18_F_4_N_6_O_2_S ([M **+** H]^+^) calcd 531.1220, found 531.1218. Anal. calcd for C_24_H_18_F_4_N_6_O_2_S: C 54.34, H 3.42, N 15.84; found C 54.33, H 3.41, N 15.82.

##### 3-{2-[4-(3,4-Difluoro-benzoylamino)-phenylamino]-5-trifluoromethyl-pyrimidin-4-ylamino}-thiophene-2-carboxylic acid methylamide (9e)

Light grey solid; 39% yield; mp: 218.9–219.7 °C; ^1^H NMR (400 MHz, DMSO-*d*_6_) δ 11.65 (s, 1H), 10.29 (s, 1H), 9.86 (s, 1H), 8.45 (s, 1H), 8.24 (s, 1H), 8.09 − 8.01 (m, 1H), 7.89 (s, 1H), 7.73 − 7.68 (m, 3H), 7.65 (s, 3H), 2.78 (s, 3H); ^13^C NMR (100 MHz, DMSO-*d*_6_) δ 164.9, 163.4, 161.5, 156.4, 155.1, 150.9, 150.8, 150.6, 148.4 (d, *J* = 12.8 Hz), 143.0, 135.8, 134.5, 132.8, 128.3, 126.4, 125.7 (d, *J* = 3.6 Hz), 125.6 (d, *J* = 3.6 Hz), 124.4, 121.4, 118.1 (d, *J* = 17.6 Hz), 117.6 (d, *J* = 18.4 Hz), 26.5; ESI-HRMS C_24_H_17_F_5_N_6_O_2_S ([M **+** H]^+^) calcd 549.1126, found 549.1130. Anal. calcd for C_24_H_17_F_5_N_6_O_2_S: C 52.56, H 3.12, N 15.32; found C 52.54, H 3.11, N 15.34.

##### 3-{5-Trifluoromethyl-2-[4-(3-trifluoromethyl-benzoylamino)-phenylamino]-pyrimidin-4-ylamino}-thiophene-2-carboxylic acid methylamide (9f)

Light grey solid; 34% yield; mp: 232.0- 233.5 °C; ^1^H NMR (400 MHz, DMSO-*d*_6_) δ 11.71 − 11.60 (m, 1H), 10.45 (s, 1H), 9.88 (s, 1H), 8.46 (s, 1H), 8.33 − 8.27 (m, 2H), 8.24 (d, *J* = 4.6 Hz, 1H), 7.98 (d, *J* = 7.6 Hz, 1H), 7.80 (t, *J* = 8.0 Hz, 1H), 7.74 (d, *J* = 8.8 Hz, 3H), 7.67 (s, 2H), 2.78 (d, *J* = 4.4 Hz, 3H); ^13^C NMR (100 MHz, DMSO-*d*_6_) δ 164.9, 164.2, 161.5, 156.4, 155.1, 143.0, 136.3, 135.9, 132.3, 130.2, 129.8, 129.5, 128.5, 128.3, 126.4, 125.8, 124.7 (d, *J* = 4.4 Hz), 124.4, 123.7, 123.1, 121.5, 113.5, 26.5; ESI-HRMS C_25_H_18_F_6_N_6_O_2_S ([M **+** H]^+^) calcd 581.1188, found 581.1184. Anal. calcd for C_25_H_18_F_6_N_6_O_2_S: C 51.73, H 3.13, N 14.48; found C 51.70, H 3.11, N 14.52.

##### 3-{2-[4-(4-Methoxy-benzoylamino)-phenylamino]-5-trifluoromethyl-pyrimidin-4-ylamino}-thiophene-2-carboxylic acid methylamide (9g)

Light yellow solid; 16% yield; mp: 229.4–230.6 °C; ^1^H NMR (400 MHz, DMSO-*d*_6_) δ 11.64 (s, 1H), 10.06 (s, 1H), 9.83 (s, 1H), 8.45 (s, 1H), 8.23 (d, *J* = 4.8 Hz, 1H), 8.01 − 7.95 (m, 2H), 7.72 (d, *J* = 8.8 Hz, 3H), 7.62 (s, 2H), 7.10 − 7.04 (m, 2H), 3.85 (s, 3H), 2.77 (d, *J* = 4.8 Hz, 3H); ^13^C NMR (100 MHz, DMSO-*d*_6_) δ 165.1, 164.9, 162.3, 161.6, 156.4, 155.1, 145.4, 143.0, 130.0, 129.1, 128.3, 127.5, 126.4, 124.4, 123.7, 121.2, 117.3, 114.1, 113.5, 55.9, 26.5; ESI-HRMS C_25_H_21_F_3_N_6_O_3_S ([M **+** H]^+^) calcd 543.1420, found 543.1422. Anal. calcd for C_25_H_21_F_3_N_6_O_3_S: C 55.35, H 3.90, N 15.49; found C 55.34, H 3.88, N 15.52.

##### 3-{5-Trifluoromethyl-2-[4-(3,4,5-trimethoxy-benzoylamino)-phenylamino]-pyrimidin-4-ylamino}-thiophene-2-carboxylic acid methylamide (9h)

Light yellow solid; 14% yield; mp: 211.9–213.1 °C; ^1^H NMR (400 MHz, DMSO-*d*_6_) δ 11.65 (s, 1H), 10.10 (s, 1H), 9.86 (s, 1H), 8.46 (s, 1H), 8.24 (d, *J* = 5.2 Hz, 1H), 7.69 (d, *J* = 8.4 Hz, 3H), 7.66 (s, 2H), 7.31 (s, 2H), 3.89 (s, 6H), 3.75 (s, 3H), 2.78 (d, *J* = 4.4 Hz, 3H); ^13^C NMR (100 MHz, DMSO-*d*_6_) δ 165.1, 164.9, 161.5, 156.4, 153.1, 151.8, 143.0, 140.8, 135.6, 134.7, 130.5 128.3, 126.4, 124.4, 123.7, 121.6, 117.4, 113.6, 105.8, 60.6, 56.6, 26.5; ESI-HRMS C_27_H_25_F_3_N_6_O_5_S ([M **+** H]^+^) calcd 603.1632, found 603.1630. Anal. calcd for C_27_H_25_F_3_N_6_O_5_S: C 53.82, H 4.18, N 13.95; found C 53.83, H 4.19, N 13.92.

##### 3-[2-(4-Propionylamino-phenylamino)-5-trifluoromethyl-pyrimidin-4-ylamino]-thiophene-2-carboxylic acid methylamide (9i)

Light yellow solid; 39% yield; mp: 233.5–235 °C; ^1^H NMR (400 MHz, DMSO-*d*_6_) δ 11.63 (s, 1H), 9.79 (d, *J* = 11.2 Hz, 2H), 8.43 (s, 1H), 8.26 − 8.19 (m, 1H), 7.67 (s, 1H), 7.55 (s, 4H), 2.77 (d, *J* = 4.8 Hz, 3H), 2.32 (q, *J* = 7.6 Hz, 2H), 1.10 (t, *J* = 7.6 Hz, 3H); ^13^C NMR (100 MHz, DMSO-*d*_6_) δ 172.1, 164.9, 161.5, 156.4, 155.1, 143.0, 135.3, 134.8, 129.8, 128.2, 126.4, 124.4, 123.7, 119.9, 113.4, 29.9, 26.5, 10.2; ESI-HRMS C_20_H_19_F_3_N_6_O_2_S ([M **+** H]^+^) calcd 465.1315, found 465.1316. Anal. calcd for C_20_H_19_F_3_N_6_O_2_S: C 51.72, H 4.12, N 18.09; found C 51.69, H 4.13, N 18.11.

##### 3-[2-(4-Isobutyrylamino-phenylamino)-5-trifluoromethyl-pyrimidin-4-ylamino]-thiophene-2-carboxylic acid methylamide (9j)

Light yellow solid; 39% yield; mp: 233.5–235 °C; ^1^H NMR (400 MHz, DMSO-*d*_6_) δ 11.63 (s, 1H), 9.79 (d, *J* = 11.2 Hz, 2H), 8.43 (s, 1H), 8.26 − 8.19 (m, 1H), 7.67 (s, 1H), 7.55 (s, 4H), 2.77 (d, *J* = 4.8 Hz, 3H), 2.32 (q, *J* = 7.6 Hz, 2H), 1.10 (t, *J* = 7.6 Hz, 3H); ^13^C NMR (100 MHz, DMSO-*d*_6_) δ 172.1, 164.9, 161.5, 156.4, 155.1, 143.0, 135.3, 134.8, 129.8, 128.2, 126.4, 124.4, 123.7, 119.9, 113.4, 29.9, 26.5, 10.2; ESI-HRMS C_20_H_19_F_3_N_6_O_2_S ([M **+** H]^+^) calcd 465.1315, found 465.1316. Anal. calcd for C_20_H_19_F_3_N_6_O_2_S: C 51.72, H 4.12, N 18.09; found C 51.70, H 4.14, N 18.12.

##### 3-[2-(4-Propynoylamino-phenylamino)-5-trifluoromethyl-pyrimidin-4-ylamino]-thiophene-2-carboxylic acid methylamide (9k)

Yellow solid; 18% yield; mp: 226.5–227.9 °C; ^1^H NMR (400 MHz, DMSO-d6) δ 11.65 (s, 1H), 9.83 (s, 1H), 8.44 (s, 1H), 8.23 (s, 1H), 7.99 − 7.41 (m, 7H), 2.92 (s, 1H), 2.77 (d, *J* = 4.4 Hz, 3H); ^13^C NMR (100 MHz, DMSO-*d*_6_) δ 164.9, 161.6, 159.1, 156.4, 155.1, 149.0, 143.0, 128.3, 124.4, 121.7, 120.6, 120.0, 113.5, 112.3, 108.1, 84.5, 82. 6, 26.5; ESI-HRMS C_20_H_15_F_3_N_6_O_2_S ([M **+** H]^+^) calcd 461.1002, found 461.0998. Anal. calcd for C_20_H_15_F_3_N_6_O_2_S: C 52.17, H 3.28, N 18.25; found C 52.19, H 3.27, N 18.22.

##### 3-{2-[4-(Cyclopropanecarbonyl-amino)-phenylamino]-5-trifluoromethyl-pyrimidin-4-ylamino}-thiophene-2-carboxylic acid methylamide (9l)

Yellow solid; 37% yield; mp > 250 °C; ^1^H NMR (400 MHz, DMSO-*d*_6_) δ 11.63 (s, 1H), 10.13 (s, 1H), 9.78 (s, 1H), 8.43 (s, 1H), 8.23 (q, *J* = 4.8 Hz, 1H), 7.68 (s, 1H), 7.55 (s, 4H), 2.77 (d, *J* = 4.4 Hz, 3H), 1.80 − 1.76 (m, 1H), 0.80 (t, *J* = 7.2 Hz, 4H)；^13^C NMR (100 MHz, DMSO-*d*_6_) δ 171.7, 164.9, 161.6, 156.4, 152.1, 145.4, 143.0, 134.8, 128.2, 124.4, 119.8, 117.7, 117.2, 115.3, 110.4, 26.5, 14.9, 7.5; ESI-HRMS C_21_H_19_F_3_N_6_O_2_S ([M**+**Na]^+^) calcd 499.1134, found 499.1135. Anal. calcd for C_21_H_19_F_3_N_6_O_2_S: C 52.94, H 4.02, N 17.64; found C 52.95, H 3.98, N 17.66.

##### 3-{2-[4-(Cyclobutanecarbonyl-amino)-phenylamino]-5-trifluoromethyl-pyrimidin-4-ylamino}-thiophene-2-carboxylic acid methylamide (9m)

Yellow solid; 18% yield; mp: 236.1–236.4 °C; ^1^H NMR (400 MHz, DMSO-*d*_6_) δ 11.63 (s, 1H), 9.78 (s, 1H), 9.66 (s, 1H), 8.43 (s, 1H), 8.23 (d, *J* = 5.6 Hz, 1H), 7.68 (s, 1H), 7.56 (s, 4H), 3.22 (t, *J* = 8.4 Hz, 1H), 2.77 (d, *J* = 4.4 Hz, 3H), 2.24 (t, *J* = 9.6 Hz, 2H), 2.11 (d, *J* = 9.2 Hz, 2H), 1.95 (q, *J* = 9.2 Hz, 1H), 1.82 (d, *J* = 10.0 Hz, 1H)；^13^C NMR (100 MHz, DMSO-*d*_6_) δ 173.0, 164.9, 161.6, 156.4, 155.1, 143.0, 136.2, 134.8, 132.1, 128.3, 126.4, 124.4, 123.7, 120.0, 113.4, 38.7, 26.5, 25.1, 18.2; ESI-HRMS C_22_H_21_F_3_N_6_O_2_S ([M**+**Na]^+^) calcd 513.1291, found 513.1292. Anal. calcd for C_22_H_21_F_3_N_6_O_2_S: C 53.87, H 4.32, N 17.13; found C 53.89, H 4.31, N 17.15.

##### 3-{2-[4-(Cyclopentanecarbonyl-amino)-phenylamino]-5-trifluoromethyl-pyrimidin-4-ylamino}-thiophene-2-carboxylic acid methylamide (9n)

Yellow solid; 46% yield; mp: 246.4–247.2 °C; ^1^H NMR (400 MHz, DMSO-*d*_6_) δ 11.63 (s, 1H), 9.79 (d, *J* = 9.2 Hz, 2H), 8.43 (s, 1H), 8.23 (d, *J* = 4.4 Hz, 1H), 7.68 (s, 1H), 7.56 (s, 3H), 7.50 (s, 1H), 2.77 (d, *J* = 4.4 Hz, 3H), 1.85 (t, *J* = 7.2 Hz, 2H), 1.78 − 1.66 (m, 5H), 1.59 − 1.53 (m, 2H); ^13^C NMR (100 MHz, DMSO-*d*_6_) δ 174.5, 174.1, 164.9, 161.5, 156.4, 155.1, 143.0, 135.2, 134.8, 128.2, 126.4, 124.4, 123.7, 119.9 (d, *J* = 4.8 Hz), 113.4, 45.7, 30.6, 26.5, 26.2 (d, *J* = 2.8 Hz); ESI-HRMS C_23_H_23_F_3_N_6_O_2_S ([M **+** H]^+^) calcd 505.1628, found 505.1634. Anal. calcd for C_23_H_23_F_3_N_6_O_2_S: C 54.75, H 4.60, N 16.66; found C 54.77, H 4.58, N 16.69.

##### 3-{2-[4-(Cyclohexanecarbonyl-amino)-phenylamino]-5-trifluoromethyl-pyrimidin-4-ylamino}-thiophene-2-carboxylic acid methylamide (9o)

Light yellow solid; 17% yield; mp: 246.7–248.2 °C; ^1^H NMR (400 MHz, DMSO-*d*_6_) δ 11.63 (s, 1H), 9.76 (d, *J* = 12.4 Hz, 2H), 8.42 (s, 1H), 8.23 (q, *J* = 4.8 Hz, 1H), 7.67 (s, 1H), 7.55 (s, 4H), 2.77 (d, *J* = 4.4 Hz, 3H), 2.36 − 2.29 (m, 1H), 1.79 (t, *J* = 14.4 Hz, 4H), 1.43 (q, *J* = 11.6, 11.2 Hz, 2H), 1.32 − 1.15 (m, 4H); ^13^C NMR (100 MHz, DMSO-*d*_6_) δ 174.5, 164.9, 161.5, 156.3, 155.0, 150.6, 143.0, 134.8, 129.4, 128.2, 126.8, 126.4, 124.4, 119.9, 113.4, 45.3, 29.7, 26.5, 25.9, 25.7; ESI-HRMS C_24_H_25_F_3_N_6_O_2_S ([M **+** H]^+^) calcd 519.1784, found 519.1790. Anal. calcd for C_24_H_25_F_3_N_6_O_2_S: C 55.59, H 4.86, N 16.21; found C 55.61, H 4.88, N 16.18.

##### Tetrahydro-pyran-4-carboxylicacid{4-[4-(2-methylcarbamoyl-thiophen-3-ylamino)-5-trifluoromethyl-pyrimidin-2-ylamino]-phenyl}-amide (9p)

Light yellow solid; 69% yield; mp: 232.3–233.8 °C; ^1^H NMR (400 MHz, DMSO-*d*_6_) δ 11.63 (s, 1H), 9.92 (s, 1H), 9.80 (s, 1H), 8.43 (s, 1H), 8.25 (q, *J* = 4.4 Hz, 1H), 7.68 (s, 1H), 7.57 (s, 4H), 3.93 (d, *J* = 3.2 Hz, 1H), 3.90 (t, *J* = 3.6 Hz, 1H), 3.39 (d, *J* = 4.0 Hz, 2H), 3.36 (d, *J* = 3.6 Hz, 2H), 2.77 (d, *J* = 4.4 Hz, 3H), 2.61 (dt, *J* = 9.6, 4.8 Hz, 1H), 1.69 (m, 4H)；^13^C NMR (100 MHz, DMSO-*d*_6_) δ 173.2, 168.8, 164.9, 161.5, 156.3, 155.7, 155.1, 153.8, 143.0, 128.3, 124.4, 123.7, 120.0, 108.6, 107.0, 66.9, 42.1, 29.4, 26.5; ESI-HRMS C_23_H_23_F_3_N_6_O_3_S ([M **+** H]^+^) calcd 521.1577, found 521.1576. Anal. calcd for C_23_H_23_F_3_N_6_O_3_S: C 53.07, H 4.45, N 16.15; found C 53.04, H 4.47, N 16.14.

##### *N*-Methyl-3-((2-((4-(4-oxocyclohexane-1-carboxamido)phenyl)amino)-5-(trifluoromethyl)pyrimidin-4-yl)amino)thiophene-2-carboxamide (9q)

Light yellow solid; 59% yield; mp: 191.3–192.1 °C; ^1^H NMR (400 MHz, DMSO-*d*_6_) δ 11.63 (s, 1H), 9.96 (s, 1H), 9.80 (s, 1H), 8.43 (s, 1H), 8.23 (d, *J* = 5.2 Hz, 1H), 7.68 (s, 1H), 7.57 (s, 4H), 2.83 (s, 1H), 2.77 (d, *J* = 4.4 Hz, 3H), 2.44 (m, 2H), 2.33 (d, *J* = 14.8 Hz, 2H), 2.14 (s, 2H), 1.91 − 1.86 (m, 2H)；^13^C NMR (100 MHz, DMSO-*d*_6_) δ 210.3, 173.1, 164.9, 161.5, 156.3, 155.1, 143.0, 135.0, 129.1, 128.2, 126.4, 124.4, 123.7, 121.8, 120.1, 113.4, 42.7, 29.4, 26.9, 26.5; ESI-HRMS C_24_H_23_F_3_N_6_O_3_S ([M **+** H]^+^) calcd 533.1577, found 533.1576. Anal. calcd for C_24_H_23_F_3_N_6_O_3_S: C 54.13, H 4.35, N 15.78; found C 54.15, H 4.32, N 15.74.

##### 1-Methyl-piperidine-4-carboxylicacid{4-[4-(2-methylcarbamoyl-thiophen-3-ylamino)-5-trifluoromethyl-pyrimidin-2-ylamino]-phenyl}-amide (9r)

Light yellow solid; 47% yield; mp: 210.8–211.6 °C; ^1^H NMR (400 MHz, DMSO-*d*_6_) δ 11.64 (s, 1H), 9.90 − 9.75 (m, 2H), 8.42 (s, 1H), 8.26 (s, 1H), 7.56 (s, 4H), 2.84 (s, 3H), 2.77 (s, 3H), 2.28 (s, 1H), 2.18 (s, 2H), 2.00 (s, 1H), 1.89 (s, 1H), 1.74 (s, 3H), 1.20 (s, 1H); ^13^C NMR (100 MHz, DMSO-*d*_6_) δ 173.8, 171.5, 168.6, 167.1, 164.9, 161.5, 156.3, 147.1, 143.0, 140.9, 139.8, 128.2, 124.4, 121.5, 119.9, 55.3, 46.6, 42.7, 28.9, 26.5; ESI-HRMS C_24_H_26_F_3_N_7_O_2_S ([M **+** H]^+^) calcd 534.1893, found 534.1885. Anal. calcd for C_24_H_26_F_3_N_7_O_2_S: C 54.03, H 4.91, N 18.38; found C 54.04, H 4.93, N 18.36.

##### 1-Acetyl-piperidine-4-carboxylicacid{4-[4-(2-methylcarbamoyl-thiophen-3-ylamino)-5-trifluoromethyl-pyrimidin-2-ylamino]-phenyl}-amide (9s)

light yellow solid; 67% yield; mp > 250 °C; ^1^H NMR (400 MHz, DMSO-*d*_6_) δ 11.63 (s, 1H), 9.87 (s, 1H), 9.79 (s, 1H), 8.43 (s, 1H), 8.23 (d, *J* = 4.8 Hz, 1H), 7.68 (s, 1H), 7.56 (s, 4H), 4.42 (d, *J* = 12.8 Hz, 1H), 3.88 (d, *J* = 13.2 Hz, 1H), 3.08 (t, *J* = 12.8 Hz, 1H), 2.77 (d, *J* = 4.4 Hz, 3H), 2.64 − 2.56 (m, 1H), 2.02 (s, 3H), 1.87 − 1.78 (m, 2H), 1.62 (m, 1H), 1.46 (m, 1H); ^13^C NMR (100 MHz, DMSO-*d*_6_) δ 173.1, 168.5, 164.9, 161.5, 156.4, 155.1, 151.9, 149.9, 148.8, 143.0, 128.2, 126.4, 124.4, 123.7, 120.0, 113.4, 43.1, 29.3, 28.6, 26.5, 21.8; ESI-HRMS C_25_H_26_F_3_N_7_O_2_S ([M **+** H]^+^) calcd 562.1842, found 562.1843. Anal. calcd for C_25_H_26_F_3_N_7_O_2_S: C 53.47, H 4.67, N 17.46; found C 53.48, H 4.64, N 17.51.

##### 3-{2-[4-(3-Phenyl-acryloylamino)-phenylamino]-5-trifluoromethyl-pyrimidin-4-ylamino}-thiophene-2-carboxylic acid methylamide (9t)

Yellow solid; 37% yield; mp: 212.5–213.8 °C; ^1^H NMR (400 MHz, DMSO-*d*_6_) δ 11.65 (s, 1H), 10.19 (s, 1H), 9.84 (s, 1H), 8.44 (s, 1H), 8.24 (s, 1H), 7.67 (s, 3H), 7.64 (s, 3H), 7.58 (s, 1H), 7.48 − 7.43 (m, 4H), 6.85 (d, *J* = 12.0 Hz, 2H), 2.80 − 2.75 (m, 3H); ^13^C NMR (100 MHz, DMSO-*d*_6_) δ 164.9, 163.7, 161.7, 161.6, 161.3, 160.7, 156.4, 155.1, 143.0, 142.9, 140.3, 135.4, 135.3, 130.2, 129.5, 128.2, 124.4, 122.9, 120.1 (d, *J* = 9.8 Hz), 113.4, 92.7, 26.5; ESI-HRMS C_26_H_21_F_3_N_6_O_2_S ([M **+** H]^+^) calcd 539.1471, found 539.1477. Anal. calcd for C_26_H_21_F_3_N_6_O_2_S: C 57.99, H 3.93, N 15.61; found C 58.01, H 3.95, N 15.59.

##### 3–(2-{4-[3-(3-Fluoro-phenyl)-acryloylamino]-phenylamino} -5-trifluoromethyl-pyrimidin-4-ylamino)-thiophene-2-carboxylic acid methylamide (9u)

Light yellow solid; 23% yield; mp: 237.5–238.8 °C; ^1^H NMR (400 MHz, DMSO-*d*_6_) δ 11.65 (s, 1H), 10.22 (s, 1H), 9.84 (s, 1H), 8.44 (s, 1H), 8.24 (s, 1H), 7.70 − 7.65 (m, 3H), 7.62 (s, 2H), 7.57 (s, 1H), 7.49 (s, 3H), 7.25 (s, 1H), 6.88 (d, *J* = 15.6 Hz, 1H), 2.78 (s, 3H); ^13^C NMR (100 MHz, DMSO-*d*_6_) δ 164.9, 164.2, 163.4, 161.7, 156.4, 155.1, 152.9, 152.3, 150.6, 148.2, 143.2, 139.0, 137.9, 137.8, 135.3, 131.5 (d, *J* = 8.4 Hz), 128.3, 124.4, 124.2 (d, *J* = 2.4 Hz), 120.2 (d, *J* = 10.8 Hz), 116.8 (d, *J* = 21.2 Hz), 114. 6 (d, *J* = 22.0 Hz), 113.5, 111.9, 26.5; ESI-HRMS C_26_H_20_F_4_N_6_O_2_S ([M **+** H]^+^) calcd 557.1377, found 557.1364. Anal. calcd for C_26_H_20_F_4_N_6_O_2_S: C 56.11, H 3.62, N 15.10; found C 56.13, H 3.63, N 15.07.

##### 3–(2-{4-[3-(4-Fluoro-phenyl)-acryloylamino]-phenylamino}-5-trifluoromethyl-pyrimidin-4-ylamino)-thiophene-2-carboxylic acid methylamide (9v)

Light yellow solid; 27% yield; mp: 248.8–249.1 °C; ^1^H NMR (400 MHz, DMSO-*d*_6_) δ 11.67 − 11.61 (m, 1H), 10.18 (s, 1H), 9.83 (s, 1H), 8.44 (s, 1H), 8.23 (d, *J* = 5.2 Hz, 1H), 7.70 (d, *J* = 8.8 Hz, 2H), 7.67 (d, *J* = 4.4 Hz, 3H), 7.61 (s, 2H), 7.57 (s, 1H), 7.29 (t, *J* = 8.4 Hz, 3H), 6.78 (m, 1H), 2.77 (d, *J* = 4.8 Hz, 3H); ^13^C NMR (100 MHz, DMSO-*d*_6_) δ 164. 9, 164.5, 163.7, 162.1, 161.5, 156.4, 155.9, 155.1, 143.6, 143.0, 139.1, 138.6, 135.3, 131.9, 130.3 (d, *J* = 8.6 Hz), 128.3, 126.4, 124.4, 122.7, 120.1 (d, *J* = 9.6 Hz), 116.5 (d, *J* = 21.6 Hz), 26.5; ESI-HRMS C_26_H_20_F_4_N_6_O_2_S ([M **+** H]^+^) calcd 557.1377, found 557.1376. Anal. calcd for C_26_H_20_F_4_N_6_O_2_S: C 56.11, H 3.62, N 15.10; found C 56.08, H 3.64, N 15.12.

##### 3-(2-{4-[3–(3-Fluoro-phenyl)-ureido]-phenylamino}-5-trifluoromethyl-pyrimidin-4-ylamino)-thiophene-2-carboxylic acid methylamide (9w)

Light yellow solid; 19% yield; mp > 250 °C; ^1^H NMR (400 MHz, DMSO-*d*_6_) δ 11.63 (s, 1H), 9.77 (s, 1H), 8.85 (d, *J* = 15.6 Hz, 1H), 8.67 (s, 1H), 8.60 (s, 1H), 8.42 (s, 1H), 8.23 (s, 1H), 7.68 (s, 3H), 7.51 (s, 1H), 7.43 (s, 1H), 7.37 (s, 1H), 7.30 (s, 1H), 7.12 (s, 1H), 6.77 (s, 1H), 2.77 (s, 3H)；^13^C NMR (100 MHz, DMSO-*d*_6_) δ 164.9, 164.1, 161.7, 156.4, 155.0, 152.9, 143.0, 142.3 (d, *J* = 3.6 Hz), 142.2, 134.5, 130.7 (d, *J* = 9.6 Hz), 128.3, 124.3, 119.7, 119.3, 114.3, 113.5, 108.5, 108.4 (d, *J* = 6.0 Hz), 105.4 (d, *J* = 4.0 Hz), 105.1 (d, *J* = 4.0 Hz), 94.2, 26.5; ESI-HRMS C_20_H_21_ClN_6_O_2_S ([M **+** H]^+^) calcd 546.1329, found 546.1334. Anal. calcd for C_20_H_21_ClN_6_O_2_S: C 52.84, H 3.51, N 17.97; found C 52.86, H 3.53, N 17.92.

##### 3–(2-{4-[3–(4-Fluoro-phenyl)-ureido]-phenylamino}-5-trifluoromethyl-pyrimidin-4-ylamino)-thiophene-2-carboxylic acid methylamide (9x)

Light green solid; 63% yield; mp > 250 °C; ^1^H NMR (400 MHz, DMSO-*d*_6_) δ 11.63 (s, 1H), 9.75 (s, 1H), 8.66 (s, 1H), 8.59 (s, 1H), 8.42 (s, 1H), 8.23 (d, *J* = 4.8 Hz, 1H), 7.68 (s, 1H), 7.54 (s, 1H), 7.47 (m, 2H), 7.43 (s, 1H), 7.41 (s, 1H), 7.12 (t, *J* = 8.8 Hz, 2H), 2.77 (d, *J* = 4.4 Hz, 3H)；^13^C NMR (100 MHz, DMSO-*d*_6_) δ 164.9, 164.1, 161.7, 156.4, 155.0, 152.9, 143.0, 142.3 (d, *J* = 3.6 Hz), 142.16, 134.5, 130.7 (d, *J* = 9.6 Hz), 128.3, 124.3, 119.7, 119.3, 114.3, 113.5, 108.5, 108.4 (d, *J* = 6.0 Hz), 105.4 (d, *J* = 4.0 Hz), 105.1 (d, *J* = 4.0 Hz), 94.2, 26.5; ESI-HRMS C_24_H_19_F_4_N_7_O_2_S ([M**+**Na]^+^) calcd 568.1149, found 568.1146. Anal. calcd for C_24_H_19_F_4_N_7_O_2_S: C 52.84, H 3.51, N 17.97; found C 52.85, H 3.48, N 18.01.

### In vitro *EGFR^wt^-TK assay*

The preliminary inhibition rates of compounds **9a**–**9x** at 1 μM against wild type EGFR (EGFR^wt^) was evaluated through ELISA kit, as our previous report. The IC_50_ values of target compounds against EGFR^wt^ were calculated from the dose − response curve under Graph-Pad Prism 9, which was tested at the different concentrations.

### In vitro *activity against cancer cell lines*

Three tumour cells A549, MCF-7, and PC-3 with high expression of EGFR^wt^ were used to investigate antitumor activities of compounds **9a–9x**. NRK-52E (Normal rat kidney cell line) cells were used to evaluate cytotoxicity of compounds **9a–9x**. They were all tested with typical MTT method, and Gefitinib were used as positive controls.

### Cytotoxicity evaluation against normal cell lines

NRK-52E (Normal rat kidney cell line) cells were used to evaluate cytotoxicity of compounds **9a–9x**. They were all tested at 10 μM with typical MTT method, and Gefitinib were used as positive controls.

### Predicted ADMET and stability properties

The absorption, distribution, metabolism, elimination, toxicity (ADMET) and stability parameters of compounds **9c**, **9e**, **9h**, **9k**, **9t**, **9u**, **9v** and Gefitinib were calculated in ADMETlab 2.0 (Website: https://admetmesh.scbdd.com/).

### A549 cell apoptosis and A549 cycle analysis

A549 cells were cultured in a cell incubator at 37 °C and 5% CO_2_ with DMEM containing double antibodies. And then, the original culture medium was replaced with 0.1 μM, 0.5 μM, and 1 μM compounds (Geftinib or the testing compound) in the cell incubator for 48 h. After trypsin digests cells, the cells were collected after centrifugation, resuspended with PBS and counted. After suspension of the cells with Annexin V – FITC binding solution and mixing with PI staining solution, the fluorescence density was detected by flow cytometry in BD Accuri C6 flow cytometry, which was provided by School of Pharmaceutical Sciences, Guizhou University, China.

### Molecular docking

The X-ray crystal structures of EGFR were obtained from the PDB bank (PDB entry 1M17 and PDB entry 6DUK), which defined the binding modes. The possible binding modes of compound **9u** were carried out with the docking module of Sybyl X-2.0 software from Tripos Inc. USA. First, the ligand with the original crystal structure was extracted as a control. For 1M17, the parameter was set to threshold 0.6 and bloat 1. For 6DUK, the parameter was set to threshold 0.5 and bloat 1. Molecular docking adopted semi flexible docking mode. The number of optimised conformation, initial conformation and the maximum conformation of each fragment was set to 50. The final conformation RMSD was less than 0.05 as the output result. Analysis and image drawing of molecular docking were completed in the open source PyMOL version 2.5.

## Results and discussions

### Chemistry

**Scheme 1.**
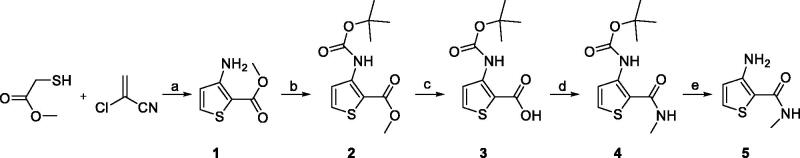
Synthetic route of compound **5**. Reagents and conditions: (a) NaOCH_3_, MeOH, rt, 57% yield; (b) di-tert-butyl dicarbonate, DMAP, DIEA, 40 °C, 50% yield; (c) THF, NaOH, 70 °C, 97% yield; (d) CH_3_NH_2_HCl, HATU, DIEA, DMF, 25 °C, 96% yield; (e) HCl, NaHCO_3_, DCM, 25 °C, 76% yield.

**Scheme 2.**
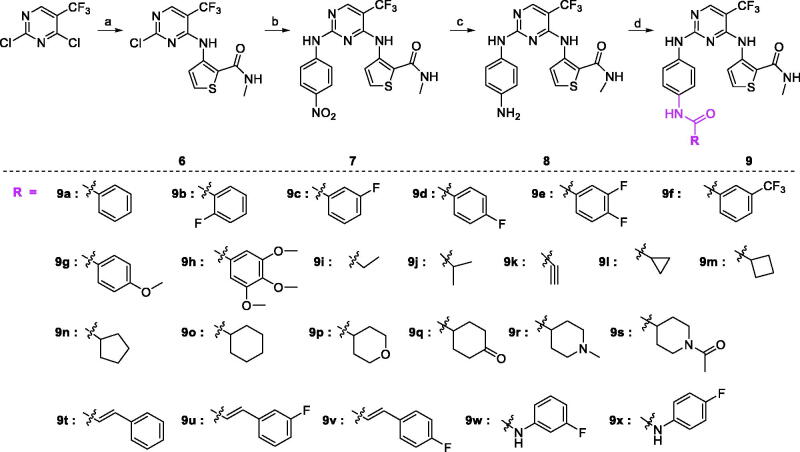
Synthetic route of compound **9a-9x**. Reagents and conditions: (a) NaH, DMF, rt, 37% yield; (b) TFA, TFE, 80 °C, 83% yield; (c) H_2_, Pd/C, rt, 69% yield; (d) HATU, DIEA, DMF, rt, 14% −69% yield.

The synthetic route of target compounds **9a–9x** were described in Schemes 1 and 2. At first, methyl thioglycolate reacted with 2-chloroacrylonitrile under the help of NaOCH_3_ to give 3-amino-thiophene-2-carboxylic acid methyl ester (compound **1**) in 57% yield, which was consisted with the previous report^30^. At the presence of Boc_2_O, DIEA, and DMAP, compound **1** was protected by Boc group to produce compound **2** with moderate yield. And then, methyl ester (compound **2**) was hydrolysed to 3-tert-butoxycarbonylamino-thiophene-2-carboxylic acid (compound **3**) within 97% yield. Under the coupling reagent HATU, compound **3** reacted with methylamine hydrochloride, DIEA in DMF to obtain compound **4** in 96% yield. At last, the Boc group in the HCl/EtOAc solution was deprotected to give the key intermediate **5** in 76% yield.

With 3-amino-thiophene-2-carboxylic acid methylamide (compound **5**) in hand, 4-chloro position in 2,4-dichloro-5-trifluoromethylpyrimidine was substituted under the condition of NaH/DMF with 37% yield. The structure of compound **6** was characterised by NMR, HRMS, and Elemental analysis. Then, compound **6** reacted with 4-nitroaniline to obtain compound **7** in 93% yield. After experienced hydrogenation reaction, compound **7** was reduced to compound **8** with 69% yield. Finally, at the presence of coupling reagent HATU, compound **8** successfully reacted with the corresponding carboxylic acid to give the target compounds **9a–9x** with 14**–**69% yield.

### In vitro activity against cancer cell lines, normal cell line, and EGFR^wt^-TK

To evaluate *in intro* antitumor activities of target compounds, A549, MCF-7, and PC-3 with high expression of EGFR^wt^-TK were selected to test antitumor activities under typical MTT method, which was employed Gefitinib as the positive control^31^. As shown in **Table 1**, the IC_50_ values of A549 cells were suggested that compounds **9b–9h**, **9k**, **9p–9q**, and **9s–9x** were more potent than Gefitinib (IC_50_ = 8.23 μM). Against MCF-7 cells, compounds **9b–9f**, **9h**, **9k**, and **9t–9x** were more potent than Gefitinib (IC_50_ = 9.31 μM). Furthermore, compounds **9a**, **9b**, **9f**, **9h**, **9k**, and **9u–9x** were more potent than Gefitinib for PC-3 cells (IC_50_ = 15.09 μM). At the same time, the inhibition of compounds **9a–9x** against EGFR^wt^ tyrosine kinase at 1 μM was preliminarily estimated with ELISA assay. Interestingly, compounds **9c**, **9e**, **9h**, **9k**, **9t**, **9u**, and **9v** were more than 50%.

**Table 1.** *In vitro* activities of target compounds for EGFR^wt^-TK and cancer cell lines^a^.

Comp.	EGFR^wt^-TK inhibition rate (%, 1 μM)	IC_50_ (μM)^a^		
		A549	MCF-7	PC-3
**9a**	32.48 ± 2.56	8.51 ± 0.94	11.87 ± 1.36	14.57 ± 0.83
**9b**	46.31 ± 1.85	4.03 ± 0.47	6.05 ± 0.82	11.25 ± 1.68
**9c**	55.48 ± 1.71	2.23 ± 0.42	5.32 ± 0.74	16.35 ± 2.27
**9d**	49.38 ± 0.47	3.75 ± 0.55	5. 77 ± 0.64	16.74 ± 1.53
**9e**	54.49 ± 1.44	2.77 ± 0.59	5. 21 ± 0.68	17.02 ± 1.82
**9f**	41.08 ± 1.79	5.13 ± 0.46	6.54 ± 0.53	12.19 ± 1.54
**9g**	28.75 ± 0.62	7.63 ± 0.62	10.05 ± 1.18	>20
**9h**	56.43 ± 0.51	1.66 ± 0.54	3.37 ± 0.62	11.35 ± 1.42
**9i**	11.92 ± 0.35	17.84 ± 1.67	>20	>20
**9j**	8.76 ± 0.68	>20	>20	>20
**9k**	55.73 ± 2.14	1.82 ± 0.49	4.91 ± 0.83	15.44 ± 2.38
**9l**	12.55 ± 1.07	16.52 ± 2.31	>20	>20
**9m**	13.29 ± 0.83	15.14 ± 2.29	>20	>20
**9n**	18.91 ± 1.14	13.76 ± 1.57	>20	>20
**9o**	26.57 ± 1.38	8.49 ± 0.63	13.46 ± 1.24	>20
**9p**	36.46 ± 0.23	7.28 ± 0.47	10.03 ± 0.96	>20
**9q**	35.53 ± 0.89	7.65 ± 0.39	10.57 ± 1.58	>20
**9r**	33.02 ± 0.57	8.41 ± 0.92	11.25 ± 1.47	>20
**9s**	37.12 ± 0.46	6.92 ± 0.38	9.75 ± 0.85	>20
**9t**	53.56 ± 1.69	3.67 ± 0.61	6.45 ± 0.53	>20
**9u**	62.73 ± 1.86	0.35 ± 0.11	3.24 ± 0.16	5.12 ± 0.73
**9v**	58.14 ± 1.76	0.46 ± 0.27	4.13 ± 0.21	7.88 ± 1.34
**9w**	47.68 ± 1.72	3.98 ± 0.83	6.15 ± 0.77	10.63 ± 1.74
**9x**	44.55 ± 1.84	4.25 ± 0.86	6.38 ± 0.97	11.46 ± 1.23
**Gefitinib**	69.11 ± 0.82	8.23 ± 0.47	9.31 ± 0.85	15.09 ± 1.06

^a^The values are mean ± *SD* of three replicates.

**Figure 3.**
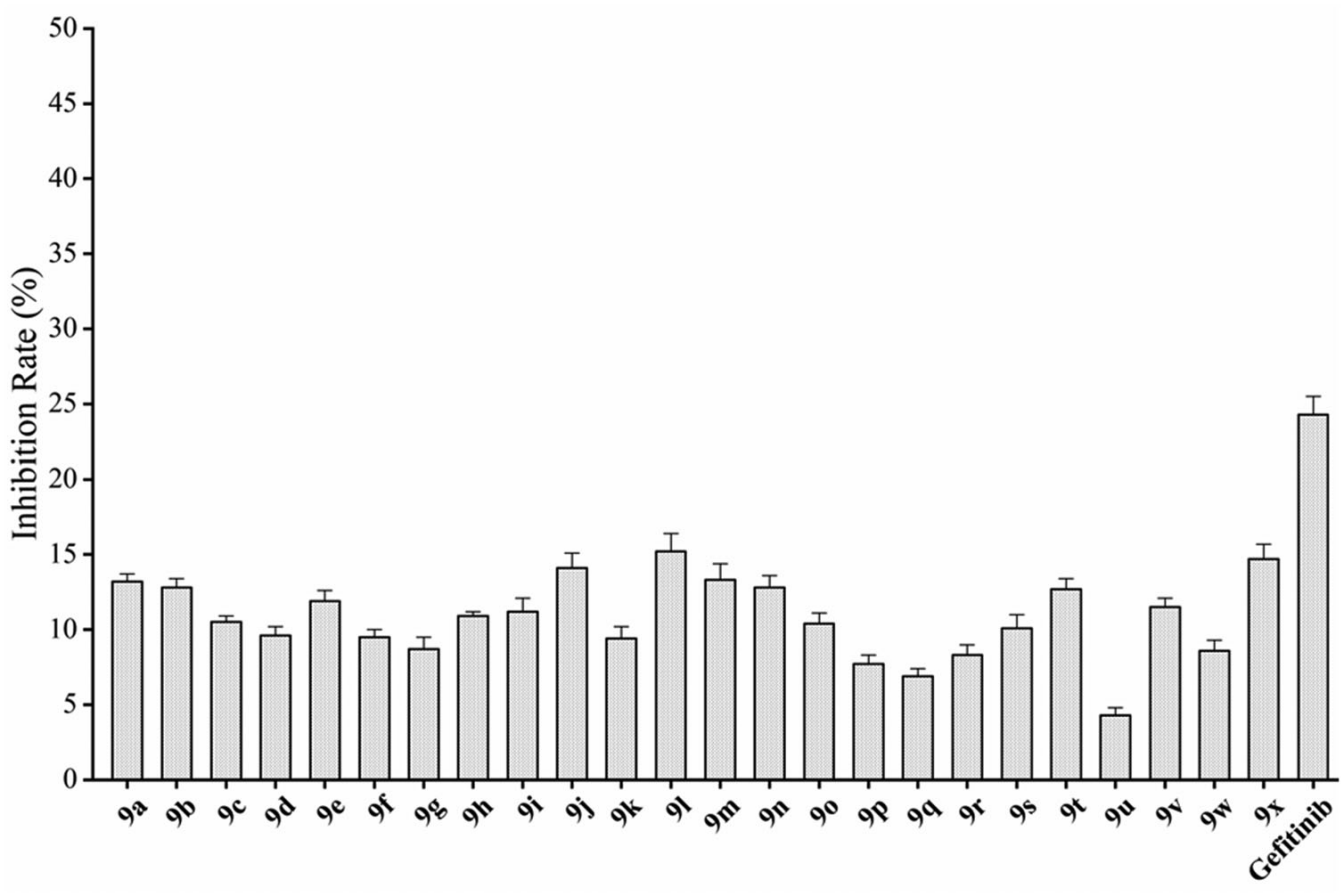
*In vitro* cytotoxicity of **9a–9x** and Gefitinib on NRK-52E.

Toxicity evaluation of synthetic compounds in early screening of antitumor drugs is very important^32^. The compounds only with less toxicity can be further developed as clinical candidate for antitumor drugs^33^. Therefore, NRK-52E (Normal rat kidney cell line) cells donated by Guizhou Medicinal University were used to evaluate the cytotoxicity. The results in Figure 3 indicated that the inhibition of compounds **9a–9x** at 10 μM were less than 15%, which was lower than Gefitinib. The results suggested that this series of 5-trifluoromethylpyrimidine derivatives had the potential to develop as antitumor agents.

According to the initial screening results in Table 1, the best seven compounds **9c**, **9e**, **9h**, **9k**, **9t**, **9u**, and **9v** were chosen to further study the IC_50_ values against EGFR^wt^. As shown in Table 2, the highest bioactive compound was compound **9u**. Its IC_50_ value against EGFR-TK reached to 0.091 μM. Meanwhile, the IC_50_ value of Gefitinib, the positive control reached to 0.0063 μM, which was consistent with the report in literature^27^.

**Table 2.** IC_50_ values for EGFR^wt^.

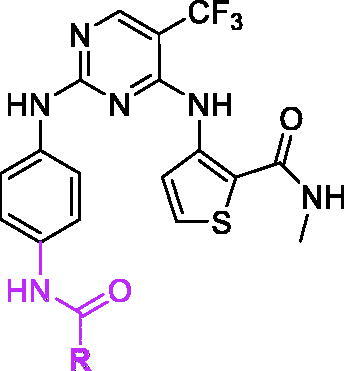 .			
Entry	Comp.	*R*	EGFR^wt^-TK IC_50_ (μM)
1	**9c**	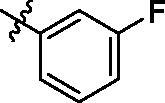	0.31 ± 0.095
2	**9e**	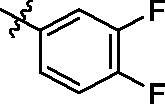	0.44 ± 0.11
3	**9h**	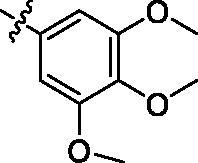	0.35 ± 0.062
4	**9k**	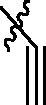	0.48 ± 0.073
5	**9t**	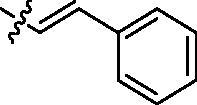	0.26 ± 0.071
6	**9u**	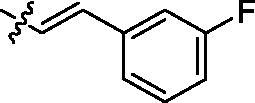	0.091 ± 0.0084
7	**9v**	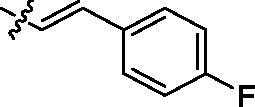	0.14 ± 0.053
8	**Gefitinib**		0.0063 ± 0.0005

^a^The values are mean ± *SD* of three replicates.

On the basis of i*n vitro* activities against cancer cell lines, and EGFR^wt^-TK, a preliminary structure-activity relationship (SAR) for compounds **9** could be discovered. When R substituents of compounds **9** were phenyl derivatives (**9a**–**9h**), antitumor activities were good. Particularly, 3-fluorophenyl compound **9c** against A549, MCF-7, and PC-3 reached to 2.23 μM, 5.32 μM, and 16.35 μM, respectively. However, the biological activities of the compounds with aliphatic substituent groups, including ethyl **9i**, isopropyl **9j**, cyclopropyl **9l**, cyclobutyl **9m**, cyclopentyl **9n**, and the other six-membered ring **9o–9s**, were very weak. It was interesting to note that compound **9k** containing ethynyl group performed well activities. Moreover, ketene compounds **9t–9v** exibited the most optimal activities. The urea substituents **9w** and **9v** also showed some activities against EGFR^wt^-TK and tumour cells. In general, these 5-trifluoromethylpyrimidine derivatives presented higher inhibition against A549 cells than MCF-7 and PC-3 cells. Excitingly, the IC_50_ values of most efficiently antitumor compound **9u** against A549, MCF-7, PC-3 cells and EGFR kinase reached to 0.35 μM, 3.24 μM, 5.12 μM, and 0.091 μM, respectively. Cytotoxicity evaluation also insisted that compound **9u** was the most potent candidate with less than 5% inhibition against NRK-52E cells.

### Predicted ADMET and stability studies

**Figure 4.**
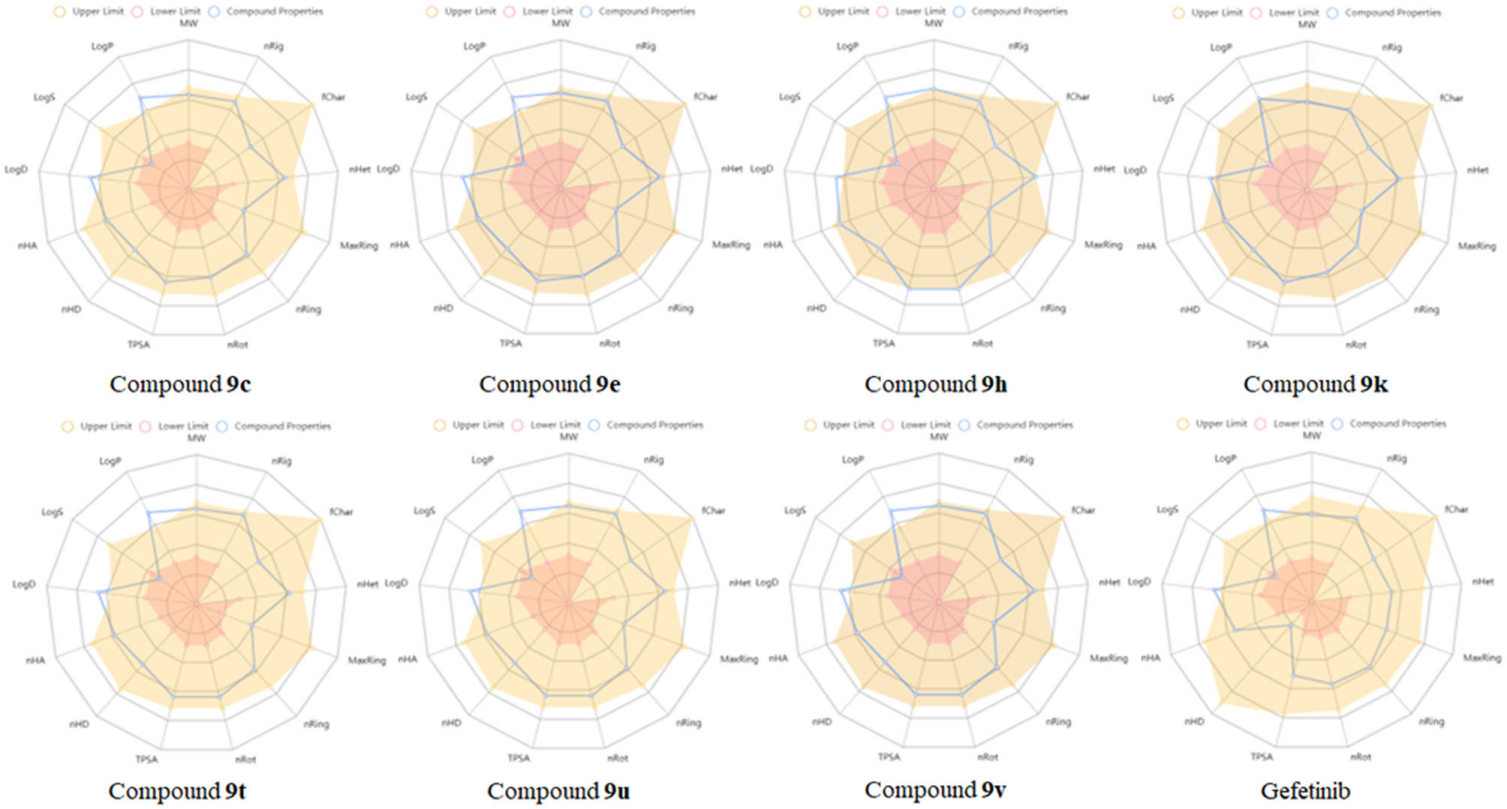
Predicted ADMET properties of the target compounds and Gefitinib.

An excellent drug candidate should possess high biochemical activities, and well absorption, distribution, metabolism, excretion, and toxicity (ADMET) profile^34–36^. Recently, many software and online websites have been developed to predict ADMET properties of antitumor candidates^37^. Last year, ADMETlab 2.0, a completely redesigned web server for the predictions of ADMET properties have been freely available^38^. A large number of researchers are now using this website for discovering new antitumor drugs^39^. We also employed this protocol to predict the ADMET properties of compounds **9c**, **9e**, **9h**, **9k**, **9t**, **9u**, **9v** and Gefitinib. As shown in Figure 4, LogD and LogP of all the test samples were a little exceeded the “Upper Limit” (Yellow area in Figure 4) and the other properties were performed well like the approved drug Gefitinib. These calculated results illustrated that the compounds **9c**, **9e**, **9h**, **9k**, **9t**, **9u**, **9v** had good ADMET properties. The specific calculated data of ADMET and stability could be found in supporting information.

### Effects of compound 9u against A549 cell apoptosis

Apoptosis is an important factor for the body to maintain its own stability. When the cell proliferation and apoptosis are unbalanced, it is easy to cause tumors^40^. More and more tumour cells such as A549 cells were found abnormal apoptosis. Meanwhile, lots of EGFR inhibitors were proved to induce A549 cell apoptosis^41^^,^^42^. As a result, compound **9u** was used to study the apoptosis of A549 cells.

**Figure 5.**
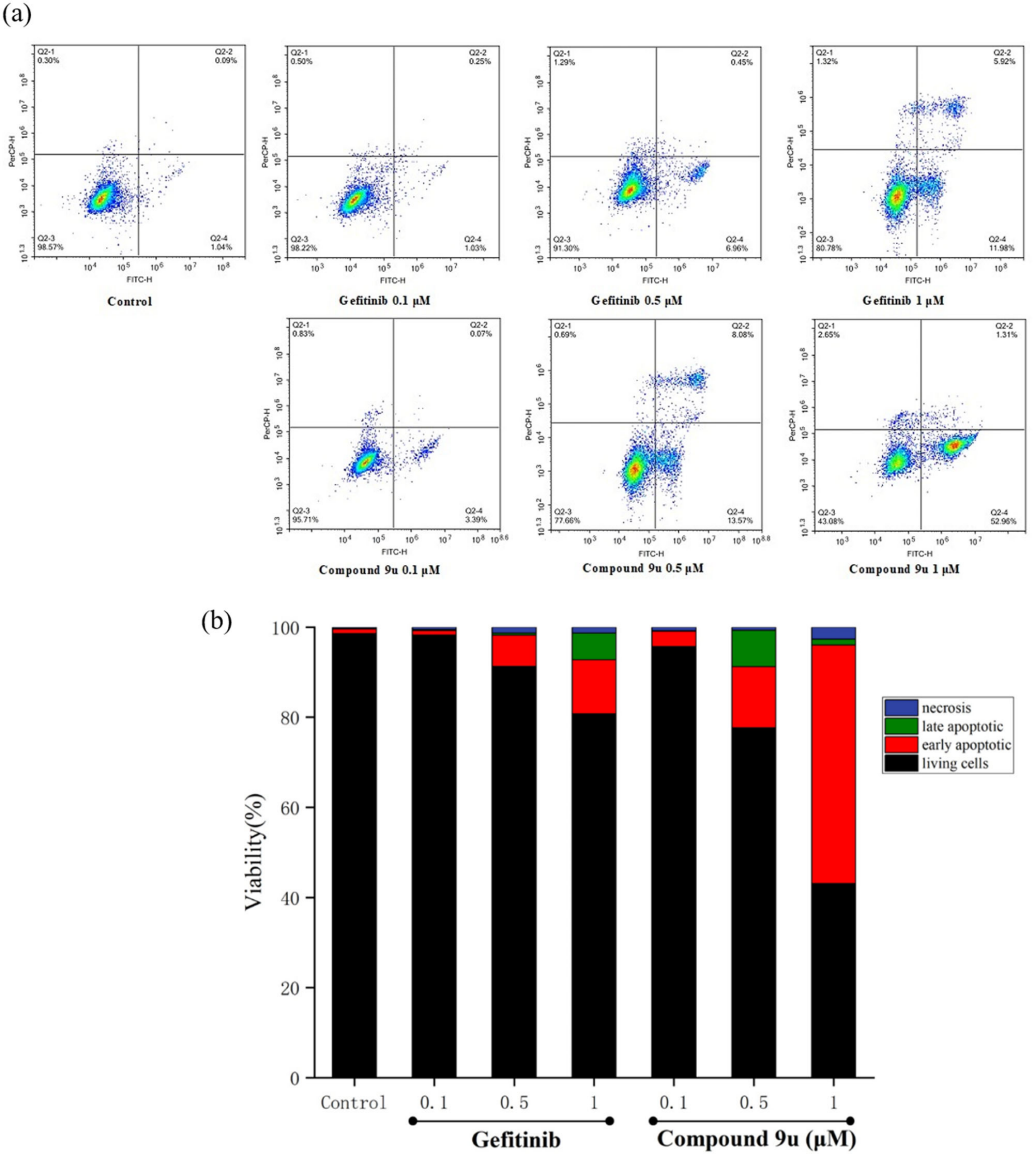
(a) Density plots were obtained by flow cytometry in the presence of different concentrations (0.1 μM, 0.5 μM and 1 μM); Gefitinib was used as the positive control. (b) Total apoptotic cells (%) at various concentrations of **9u** and Gefitinib.

As shown in Figure 5(a), Gefitinib and compound **9u** were tested in flow cytometer at 0.1 µM, 0.5 µM, and 1 µM, respectively. For Gefitinib, the early apoptosis increased from 1.03% to 11.98% accompanying with a dose-dependent characteristics. Interestingly, the early apoptotic A549 cells for compound **9u** sharply increased from 3.39% to 52.96%. Meanwhile, it could be found in Figure 5(b) that compound **9u** performed more effectively early apoptosis than Gefitinib at the same concentration. The total numbers of apoptosis (early and late apoptosis) compound **9u** were also greater than Gefitinib at the different concentrations.

### Effects of compound 9u against cell cycle of A549 cell line

The cell cycle of tumour cells is closely related to apoptosis. A great number of EGFR inhibitors could induce A549 cell apoptosis and block cell cycle^43^^,^^44^. According to these reports in literature, the cell cycle of compound **9u** against A549 was studied in flow cytometry.

**Figure 6.**
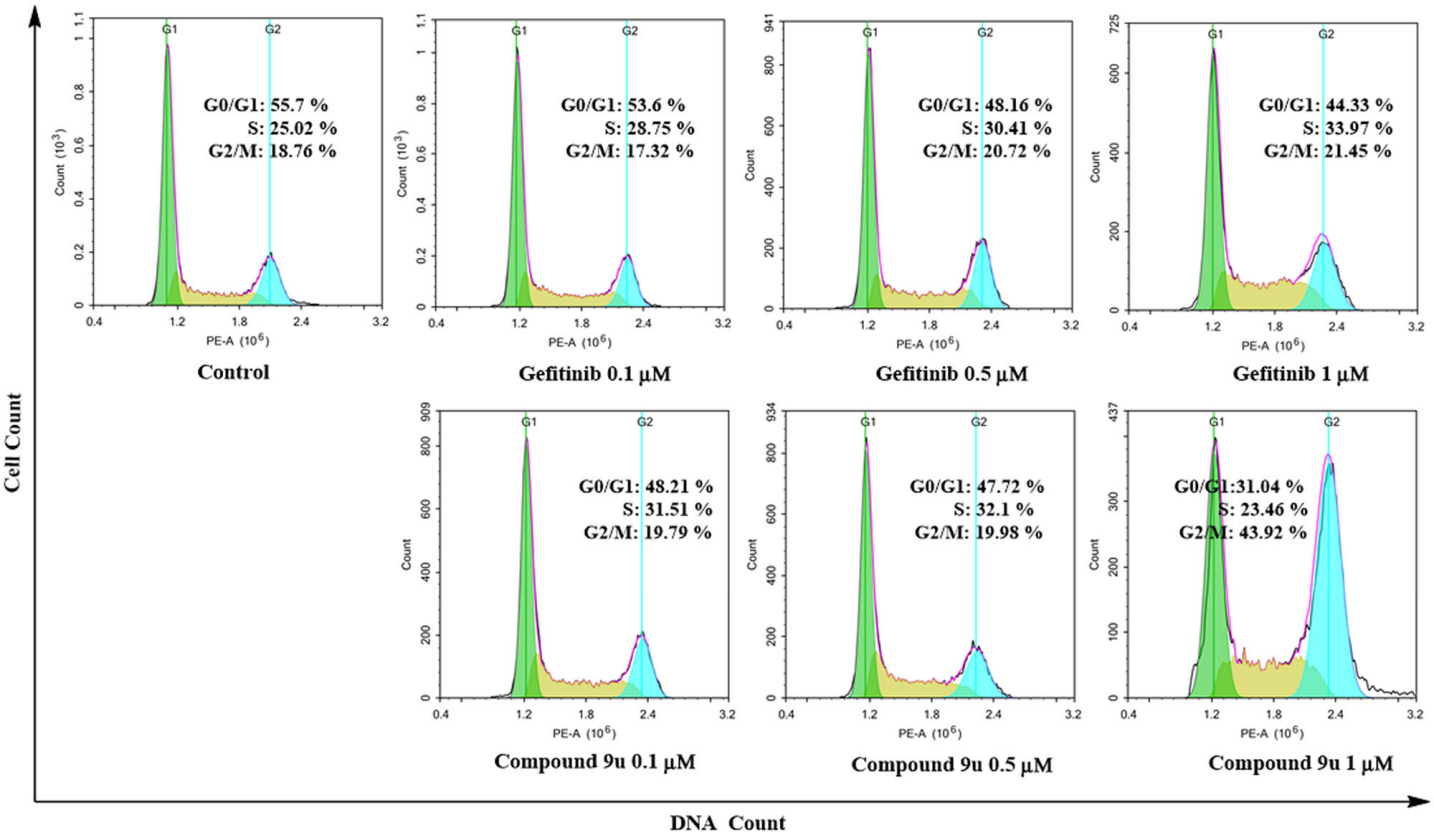
Proportion of A549 cells treated with compound **9u** (0.1 μM, 0.5 μM and 1 μM) or Gefitinib (0.1 μM, 0.5 μM and 1 μM) at G0/G1, S and G2/M phases.

As shown in Figure 6, Gefitinib and compound **9u** were evaluated at 0.1 µM, 0.5 µM, and 1 µM, respectively. With the increase of testing concentration, the G2/M phase A549 cells treated by Gefitinib were 17.32%, 20.72%, and 21.45%. However, the G2/M phase A549 cells treated by compound **9u** were sharply promoted from 19.79% to 43.92%. This indicate that compound **9u** showed better cell cycle arrest effect than Gefitinib and could arrest A549 cells in G2/M phase.

### Molecular docking of compound 9u

**Figure 7.**
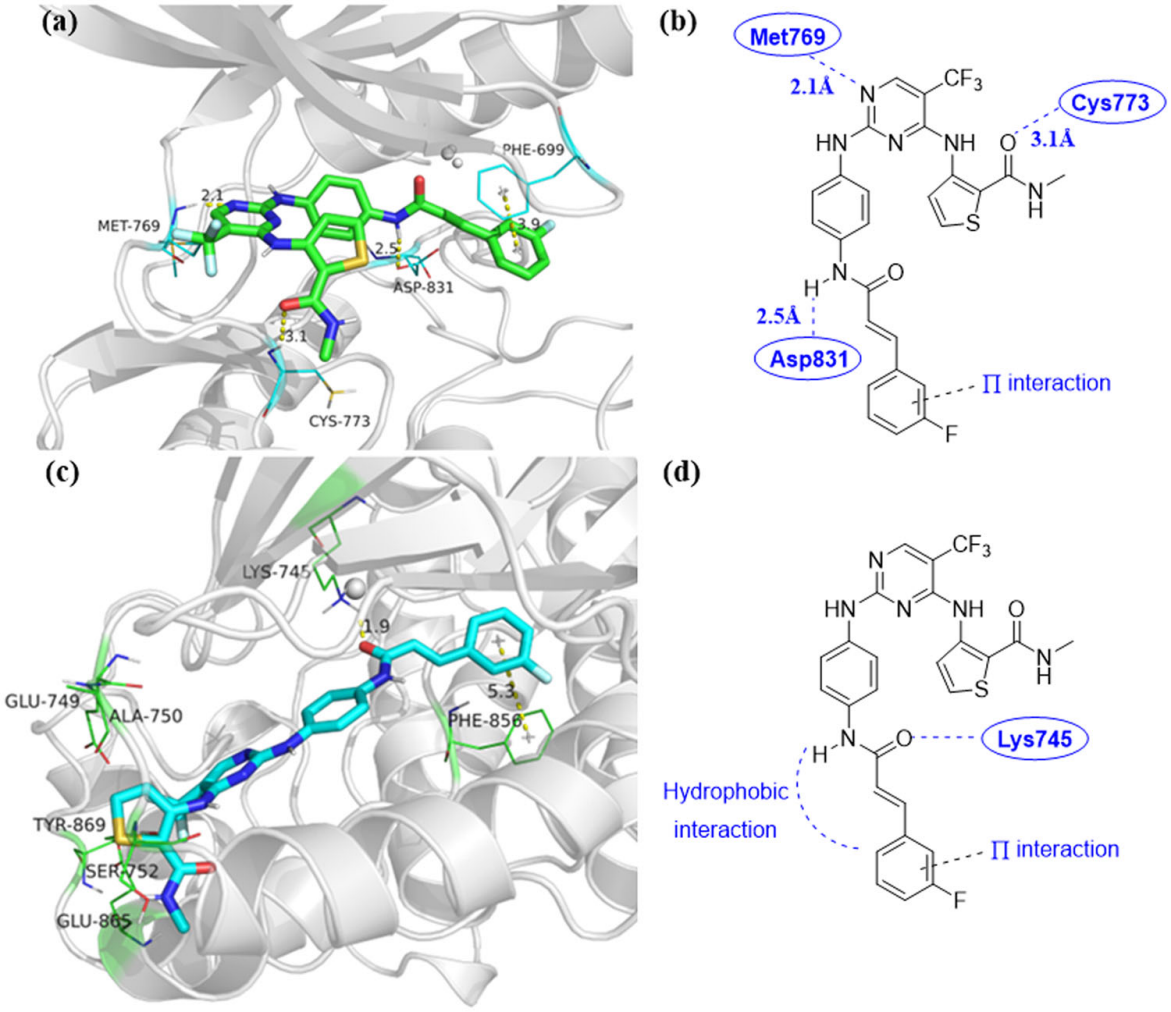
(a) Binding configuration of compound **9u** with EGFR (PDB: 1M17); (b) The 2 D model of compound **9u** bound to EGFR (PDB: 1M17); (c) Binding configuration of compound **9u** with EGFR (PDB: 6DUK); (d) The 2D model of compound **9u** bound to EGFR (PDB: 6DUK).

Molecular docking is one of the important methods of molecular simulation. Its essence is the recognition process between two or more molecules, which involves the spatial matching and energy matching between molecules^45^. In addition, molecular docking can simulate the interaction between small molecules and macromolecules, providing more information for optimising the activity of lead compounds^46^^,^^47^. To investigate the possible binding modes, compound **9u** and EGFR were employed in Sybyl X-2.0 software for molecular docking^48^. Two different crystal structures of EGFR were used in this method, including the ATP-competitive inhibitor (PDB entry 1M17)^49^ and the allosteric inhibitor (PDB entry 6DUK)^50^. For ATP-competitive inhibitor in Figure 7(a,b), compound **9u** could strongly formed three hydrogen bonds with Met769, Cys773, and Asp831 of EGFR, respectively. The hydrogen bond lengths were 2.1 Å, 3.1 Å, and 2.5 Å, respectively. Moreover, the benzene ring at the end of compound **9u** formed π-π interactions with EGFR tyrosine kinase. However, there were no hydrogen bonds in Figure 7(c,d) as the PDB entry 6DUK. Only hydrophobic and π–π interactions could be found at the end area of compound **9u**. This suggested that compound **9u** could tightly combine with EGFR, which could be used as ATP-competitive inhibitor.

## Conclusion

In this paper, a new series of 5-trifluoromethylpyrimidine derivatives were designed as EGFR inhibitors. Experienced a five-step process, 3-amino-thiophene-2-carboxylic acid methylamide was successfully prepared. Subsequently, the target compounds were rapidly synthesised by four steps, and the structures were confirmed by NMR, HRMS, and elemental analysis. After evaluation of biological activities including A549, MCF-7, PC-3 and EGFR kinase, some of the target compounds existed excellent antitumor activities. In particular, the IC_50_ values of compound **9u** against A549, MCF-7, PC-3 cells and EGFR kinase reached to 0.35 μM, 3.24 μM, 5.12 μM, and 0.091 μM, respectively. Moreover, flow cytometry analysis of A549 showed that compound **9u** could induce early apoptosis of and arrest cells in the G2/M phase. In addition, predicted ADMET properties and molecular docking studies indicated that compound **9u** was potential as new antitumor reagent.

## Supplementary Material

Supplemental Material
