# Supplementary material for: Design, synthesis and antitumor activity of 5-trifluoromethylpyrimidine derivatives as EGFR inhibitors
Source: J Enzyme Inhib Med Chem. 2022 Sep 29;37(1):2742–54. doi: 10.1080/14756366.2022.2128797 (PMC9542405; doi:10.1080/14756366.2022.2128797)
Supplement: Supplemental Material [file IENZ_A_2128797_SM2760.pdf]

# Supporting information

## Design, synthesis and antitumor activity of 5-trifluoromethylpyrimidine derivatives as EGFR inhibitors

Yaqing Zuo [1,2], Rongrong Li [1,2], Yan Zhang [1,2,3], Guochen Bao [4], Yi Le [1,2,3], Longjia Yan [1,2,3,*]

[1] School of Pharmaceutical Sciences, Guizhou University, Guiyang 550025

[2] State Key Laboratory of Functions and Applications of Medicinal Plants, Guizhou Medical University, Guiyang 550014, China

[3] Guizhou Engineering Laboratory for Synthetic Drugs, Guiyang 550025, China

[4] Institute for Biomedical Materials and Devices (IBMD), Faculty of Science, University of Technology Sydney, Sydney, New South Wales, Australia

E-mail: ylj1089@163.com (Longjia Yan).

## Table of contents

General reagents and instruments……….………………………...……………...…..**S2**

Molecular docking figures………...……….………………………...……………...…..**S3**

NMR and HRMS spectra ……………………………………………...………..…..**S4-S50**

Predicted ADMET compound **9c**………………...……………………………...…..**S51-S56**

Predicted ADMET compound **9e**………………...……………………………...…..**S57-S62**

Predicted ADMET compound **9h**………………...……………………………...…..**S63-S68**

Predicted ADMET compound **9k**………………...……………………………...…..**S69-S74**

Predicted ADMET compound **9t**………………...……………………………...…..**S75-S80**

Predicted ADMET compound **9u**………………...……………………………...…..**S81-S86**

Predicted ADMET compound **9v**………………...……………………………...…..**S87-S92**

Predicted ADMET Gefitinib…...…………………...……………………………...…..**S93-S98**

## General reagents and instruments

All chemical reagents were commercially available in Sigma-Aldrich or Energy Chemical Cooperation in China. The corresponding biochemical reagents were purchased from PTM Bio. Co., Ltd. A549 (Human non-small-cell lung cancer cell line) cells, MCF-7 (Human breast cancer cell line) cells, and PC-3 (Human prostate cancer cell line) cells. They were all purchased from the Shanghai Cell Bank of the Chinese Academy of Sciences. NRK-52E (Normal rat kidney cell line) cells donated by Guizhou Medicinal University were also used to evaluate the cytotoxicity.

The $^{1}$H-NMR and $^{13}$C-NMR spectra were measured on Bruker (Avance) 400 MHz instrument in School of Pharmaceutical Sciences, Guizhou University, China, which were reported as δ in $CDCl_3$ or DMSO-$d_6$. The high-resolution mass spectrometer (HRMS) was tested in TSQ 8000 high-resolution mass spectrometer and AB SCIEX X500R QTOF. Elemental analysis was recorded in vario EL Cube (Elementar, Germany). BD Accuri C6 flow cytometry (American BD Corporation Shanghai Co., Ltd.) was provided by the School of Pharmaceutical Sciences, Guizhou University, China.

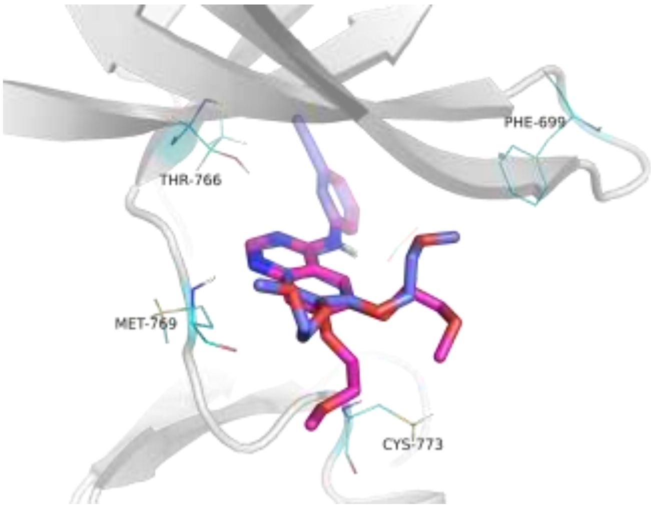


**Figure S1**. Binding configuration of Erlotinib with EGFR (PDB: 1M17). The purplish red conformation was the resolved conformation of the original crystal structure, and the dark blue was the molecular docking conformation of Erlotinib. The RMSD was less than 2, and the similarity score calculated in Sybyl was 0.753, indicating that the docking model was relatively reliable. Total docking score (1M17 ligand) was 10.42.

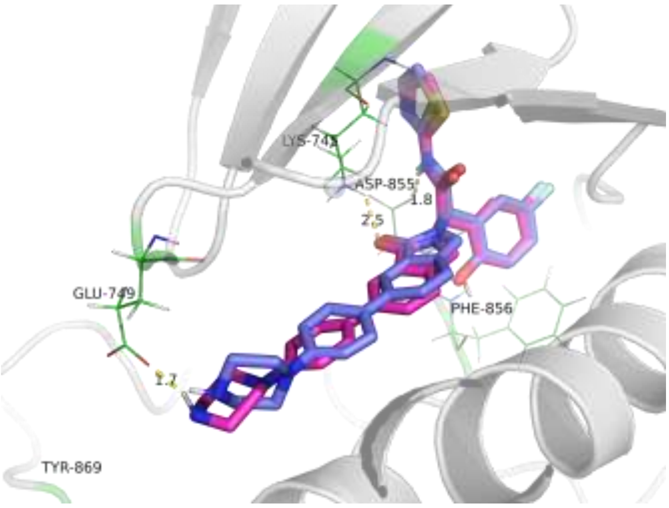


**Figure S2**. Binding configuration of JBJ with EGFR (PDB: 6DUK). The purplish red conformation was the resolved conformation of the original crystal structure, and the dark blue was the molecular docking conformation of JBJ, which was similar to the original crystal conformation. The docked conformation formed hydrogen bonds with Ala750, Asp855 and Lys745, with lengths of 1.7, 1.8 and 2.5 Å, respectively. The RMSD was less than 2, and the similarity score calculated in Sybyl was 0.802, indicating that the docking model was relatively reliable. Total docking score (6DUK ligand) was 15.07.

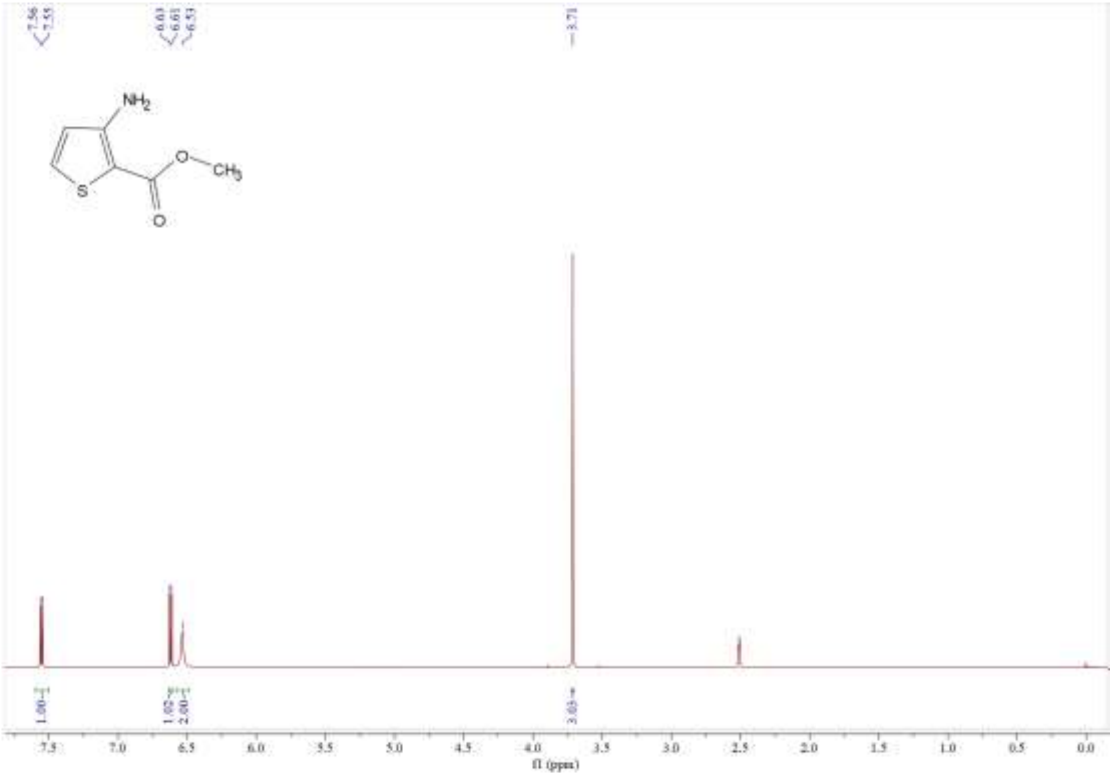


**Figure S3**. $^{1}$H NMR spectrum of compound **1**

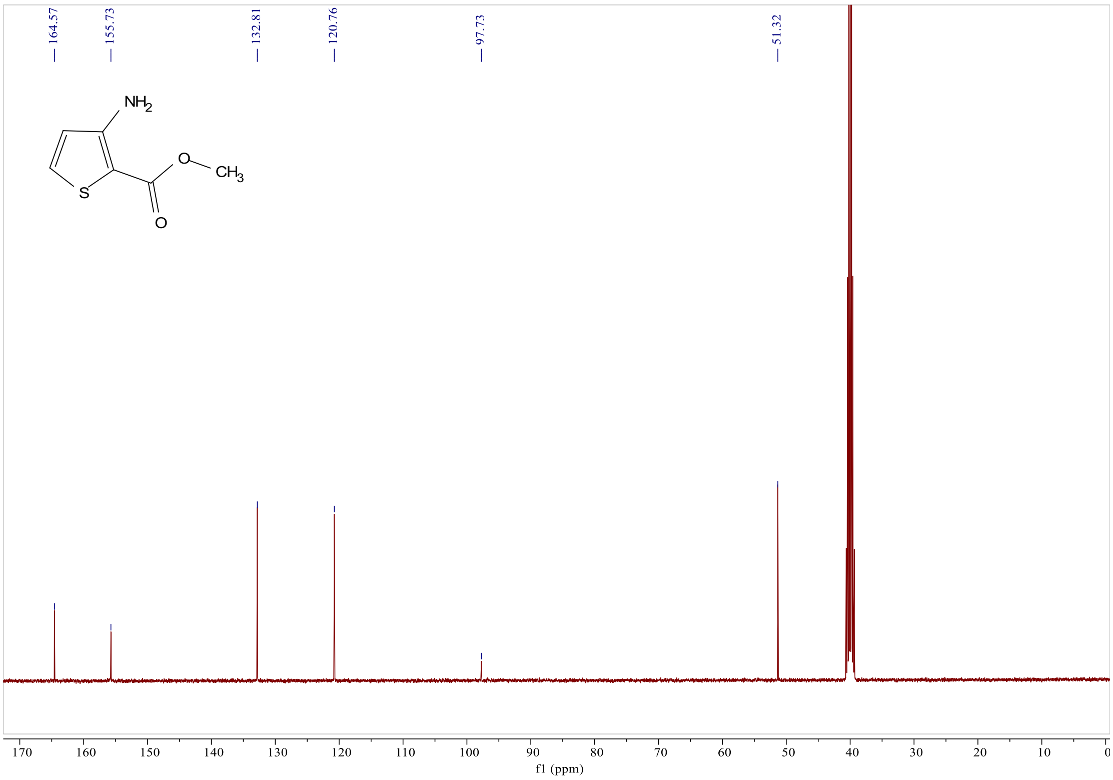


**Figure S4**. $^{13}$C NMR spectrum of compound **1**

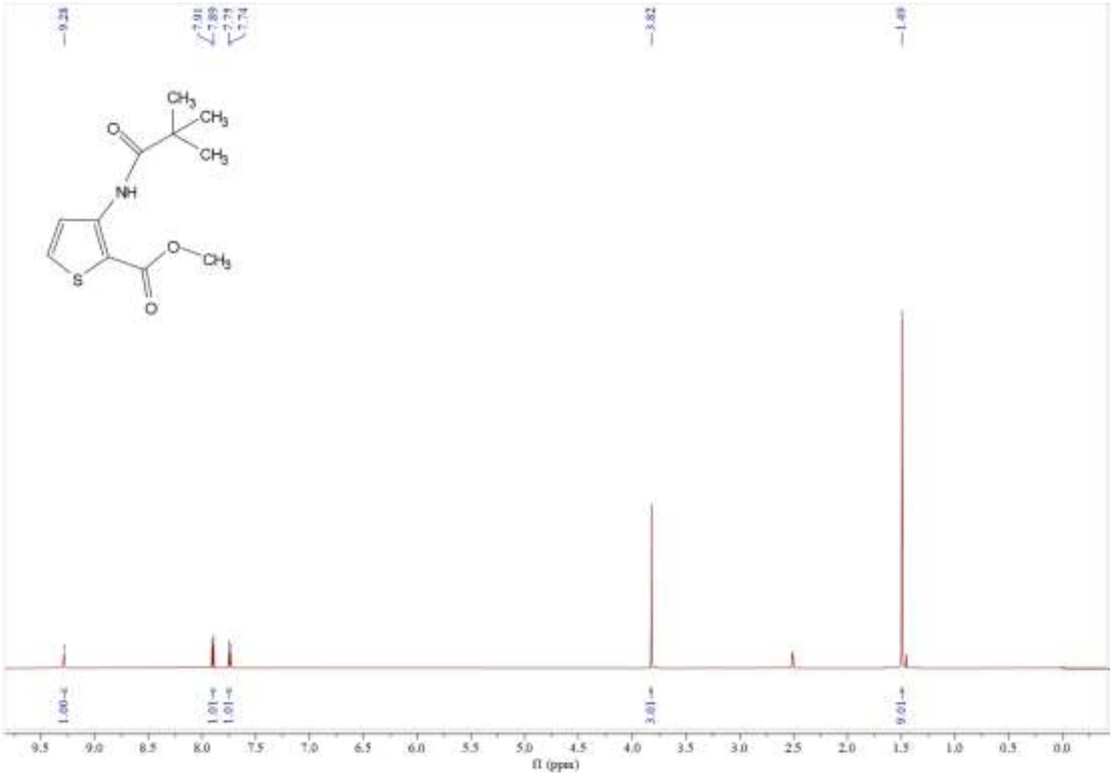

**Figure S5**. $^{1}$H NMR spectrum of compound **2**

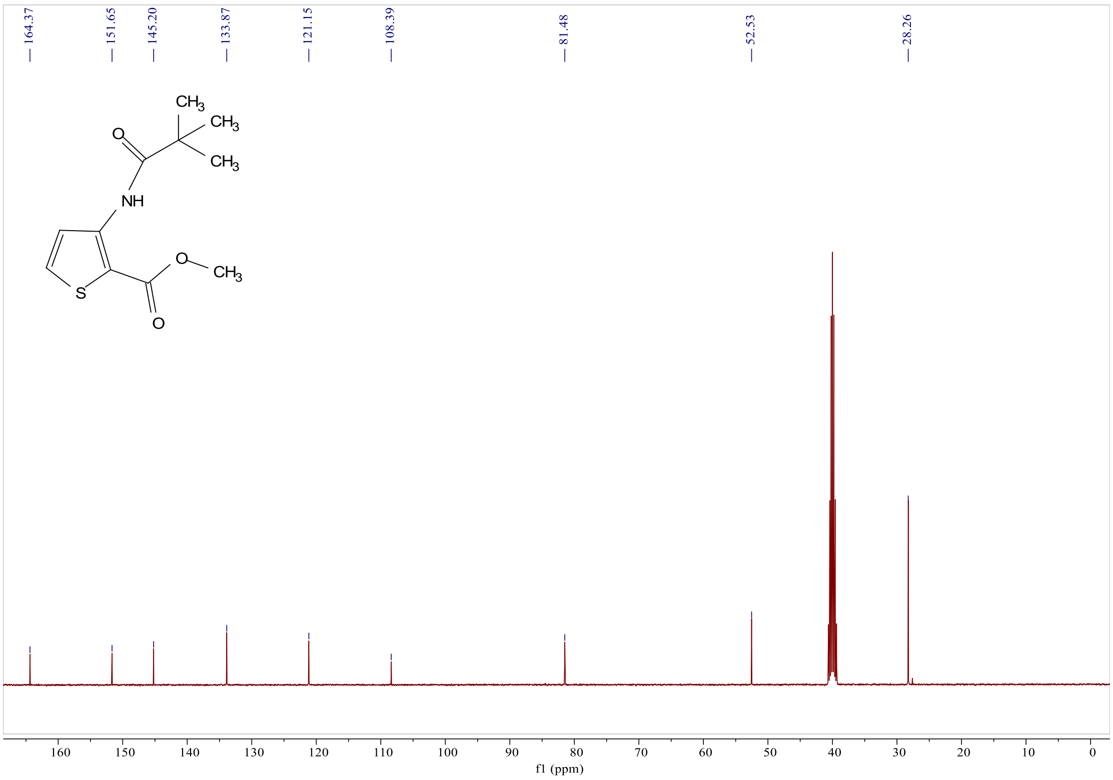

**Figure S6**. $^{13}$C NMR spectrum of compound **2**

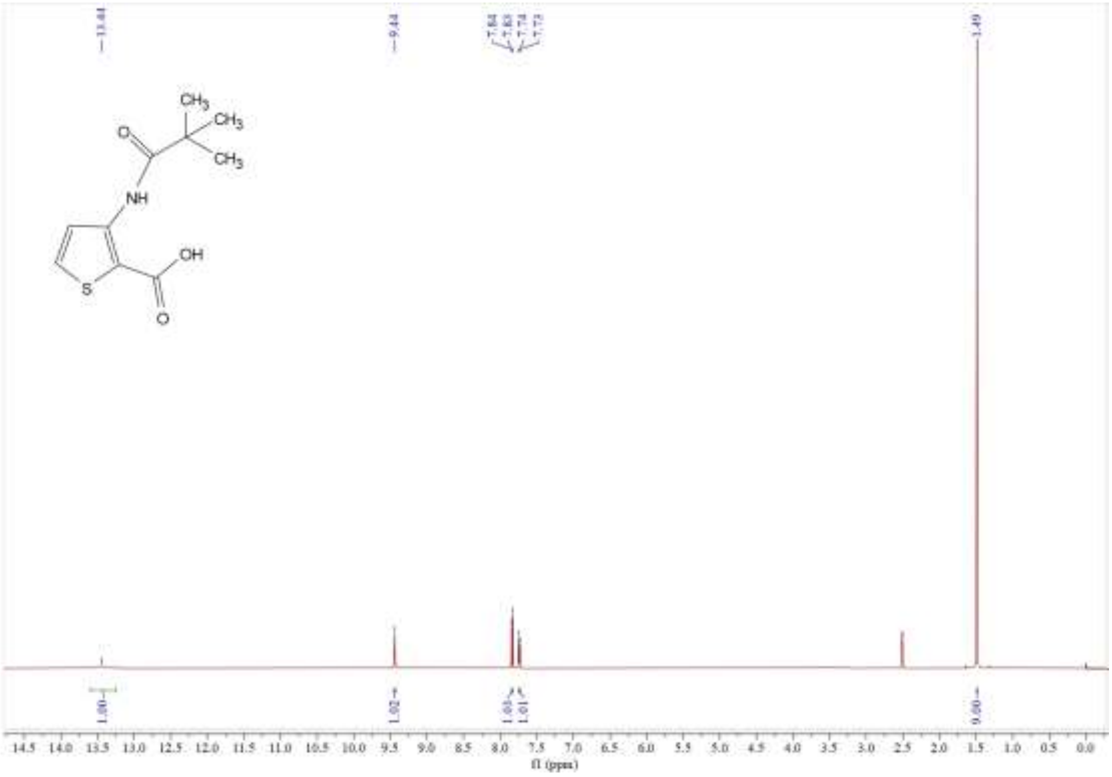

**Figure S7**. $^{1}$H NMR spectrum of compound **3**

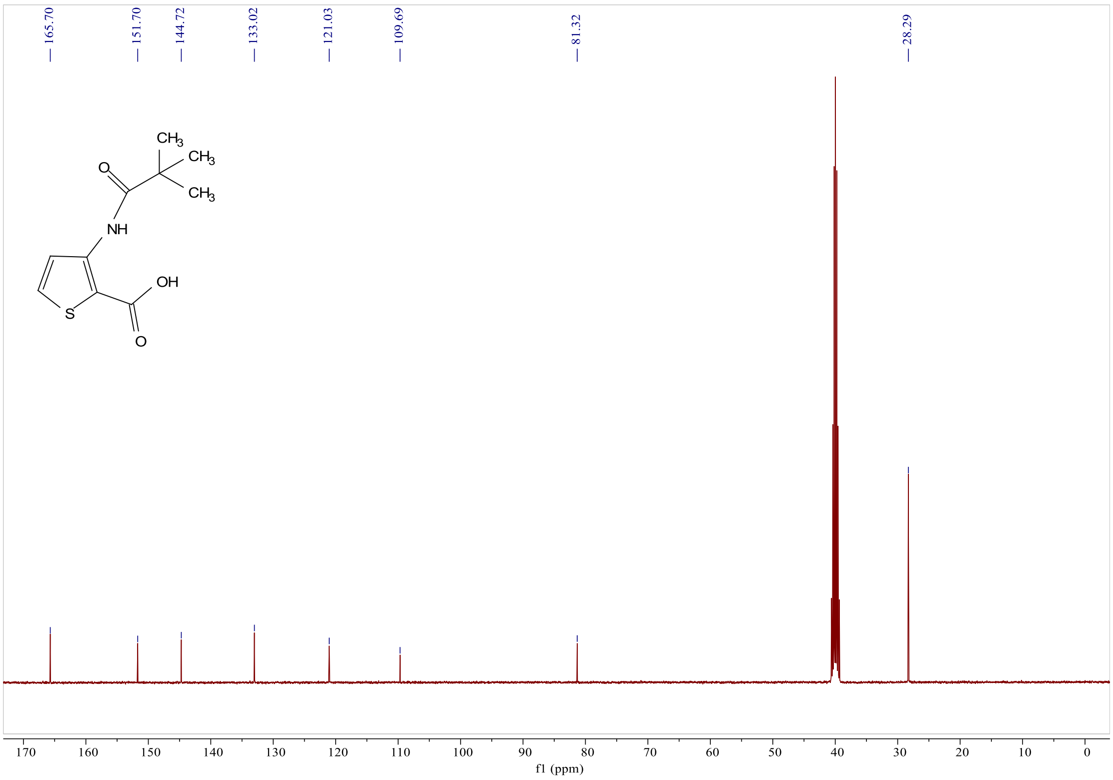

**Figure S8**. $^{13}$C NMR spectrum of compound **3**

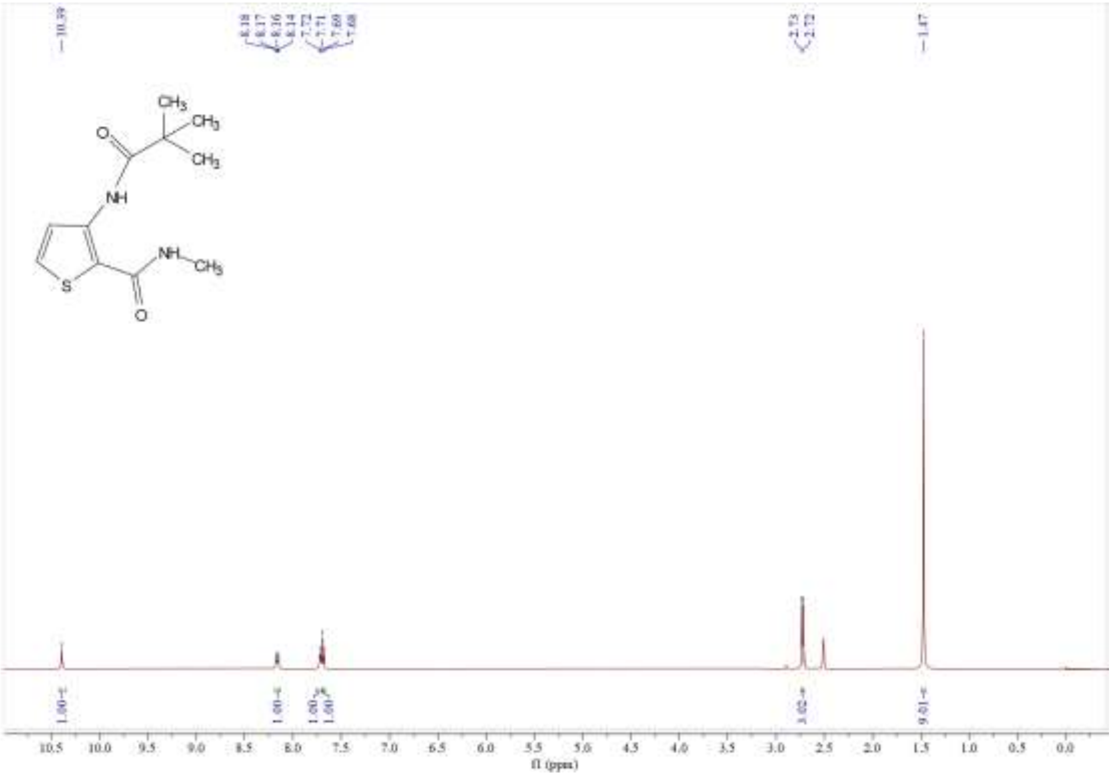

**Figure S9**. $^{1}$H NMR spectrum of compound **4**

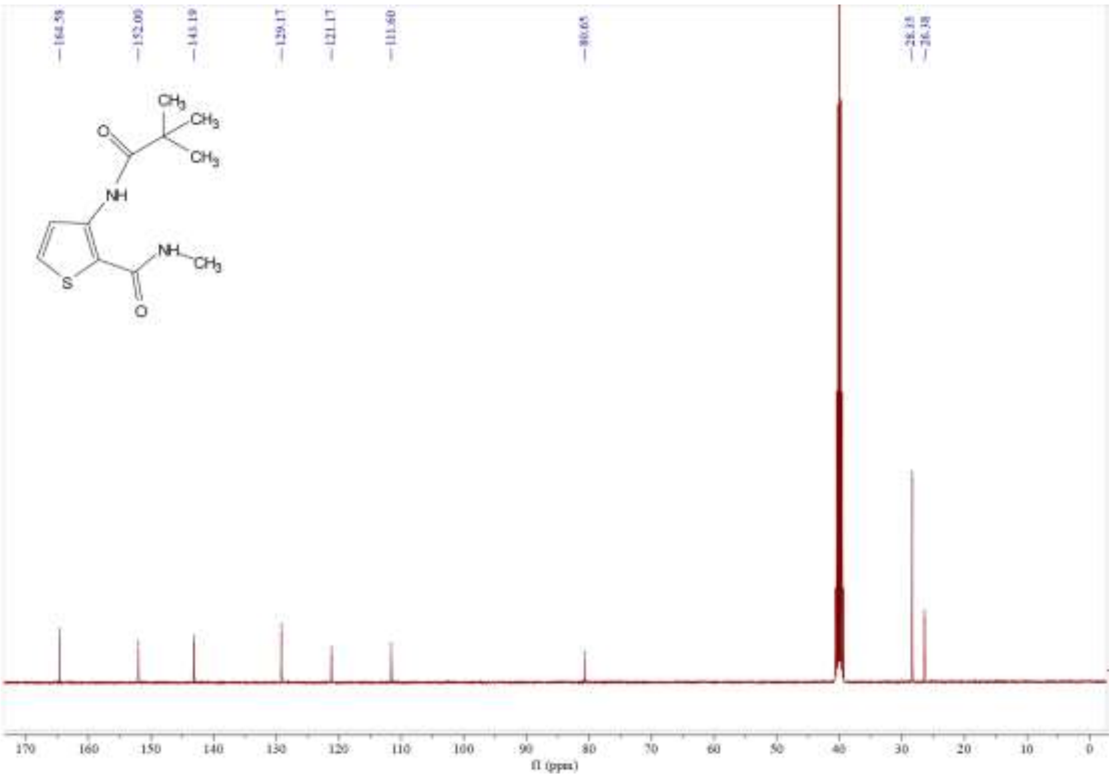

**Figure S10**. $^{13}$C NMR spectrum of compound **4**

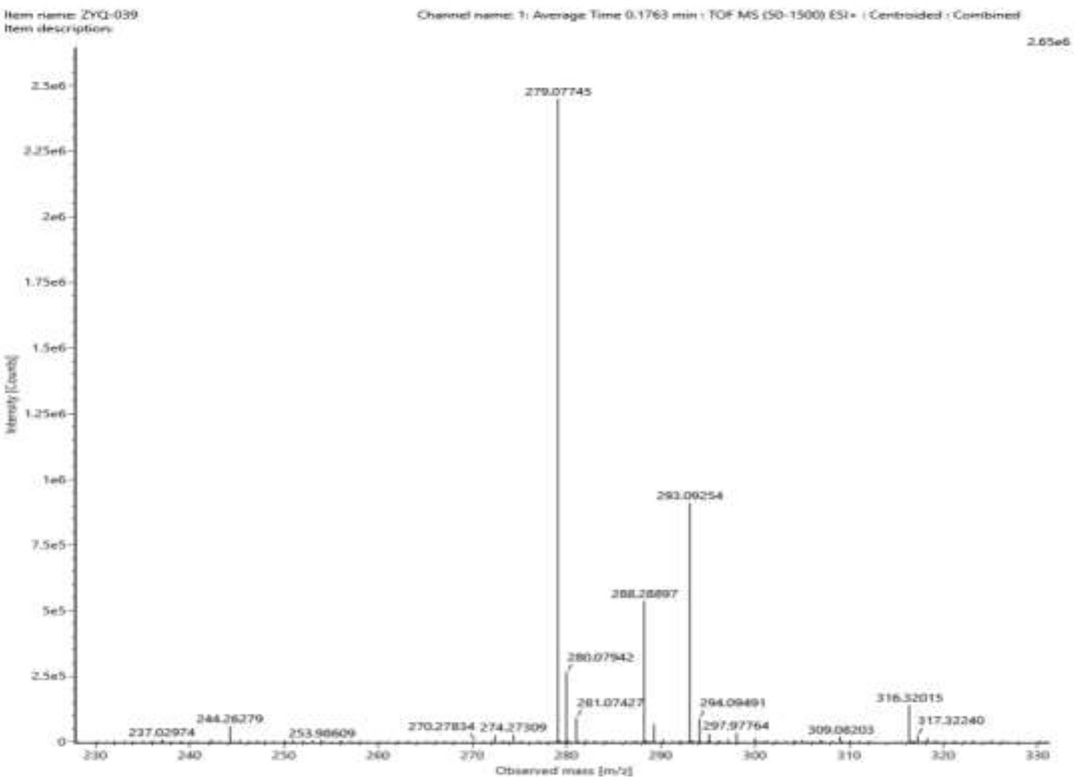


**Figure S11**. HR-MS (ESI) spectrogram of compound **4**

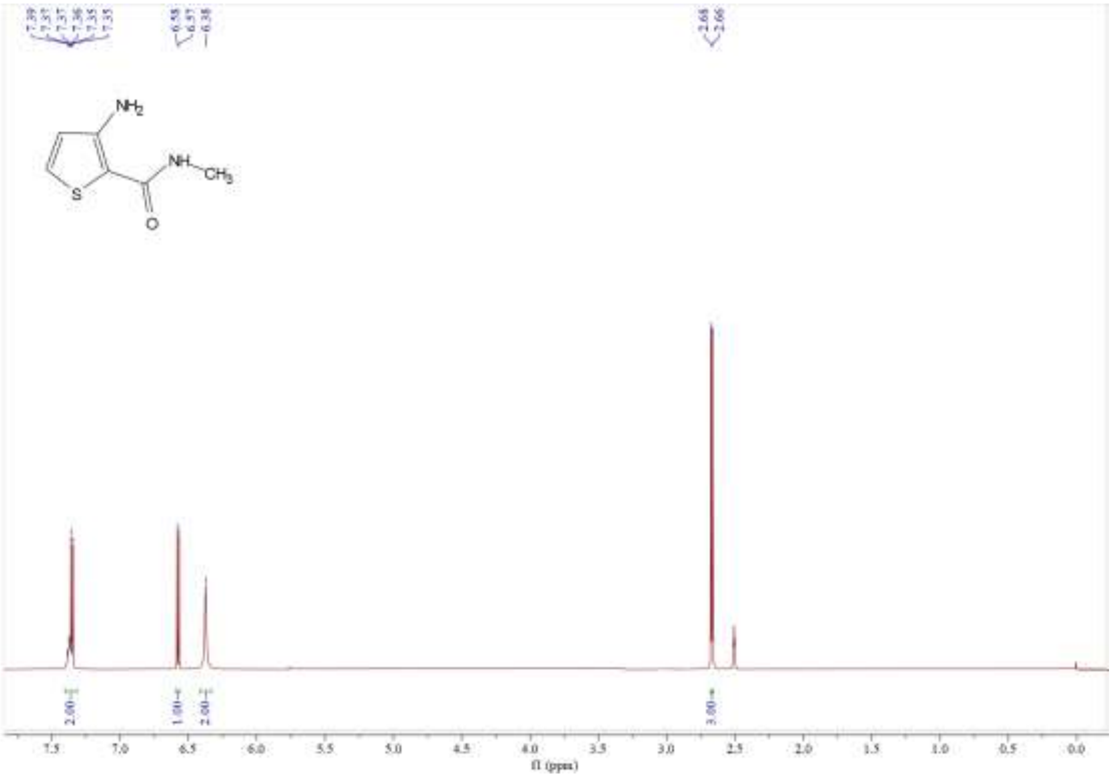


**Figure S12**. $^1$H NMR spectrum of compound **5**

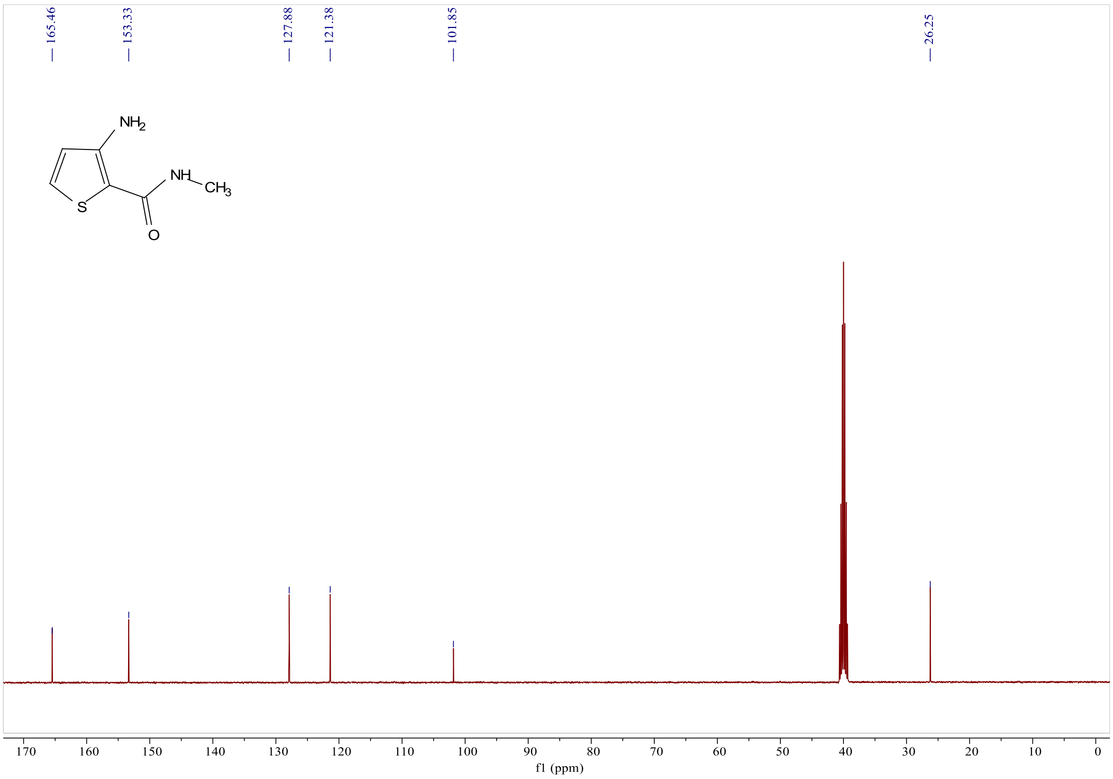


**Figure S13**. $^{13}C$ NMR spectrum of compound **5**

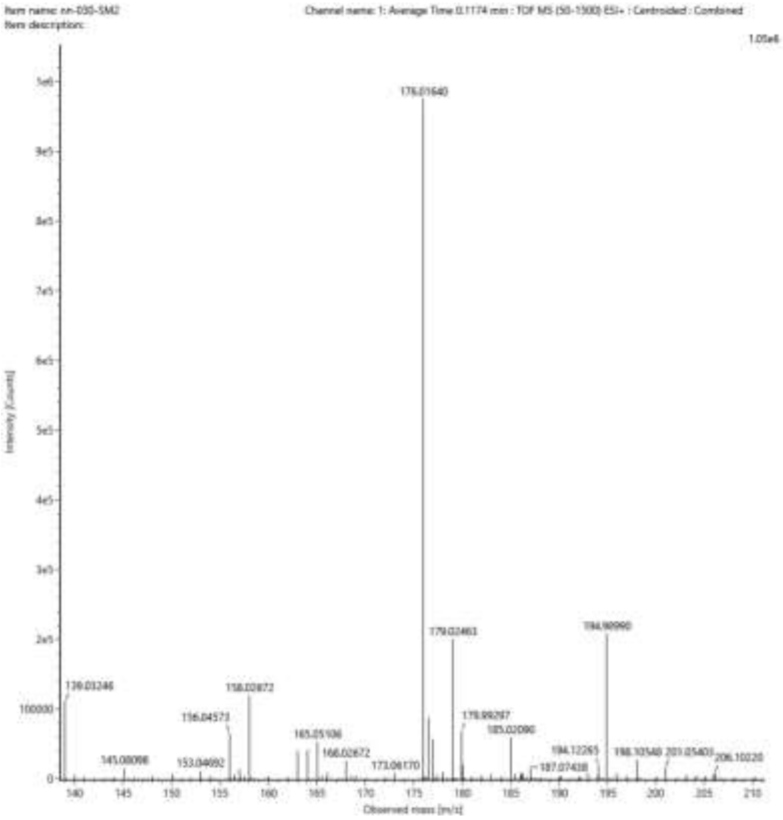

**Figure S14**. HR-MS (ESI) spectrogram of compound **5**

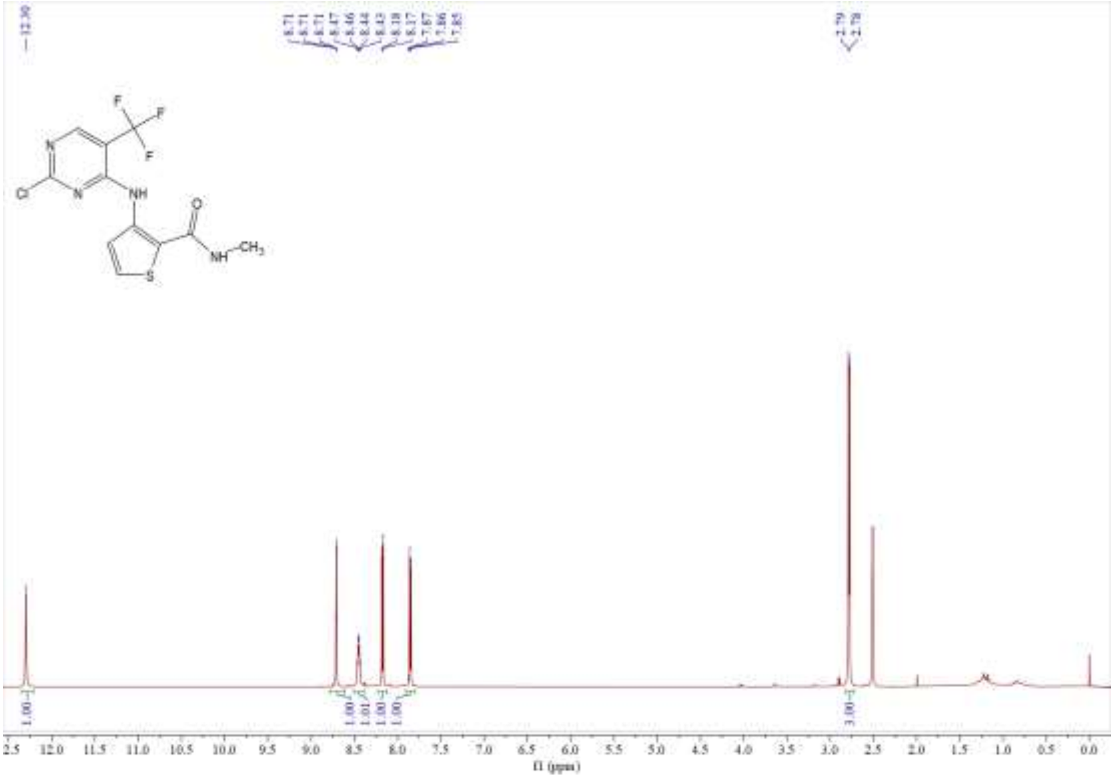

**Figure S15**. $^{1}H$ NMR spectrum of compound **6**

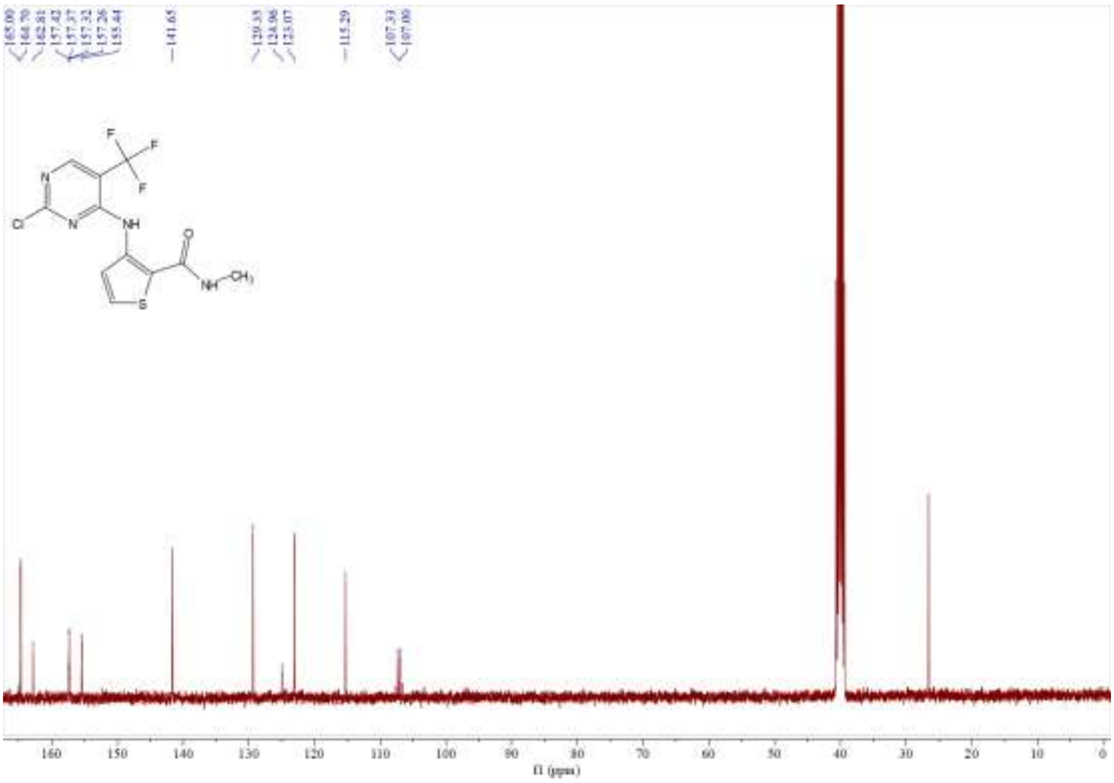

**Figure S16**. $^{13}C$ NMR spectrum of compound **6**

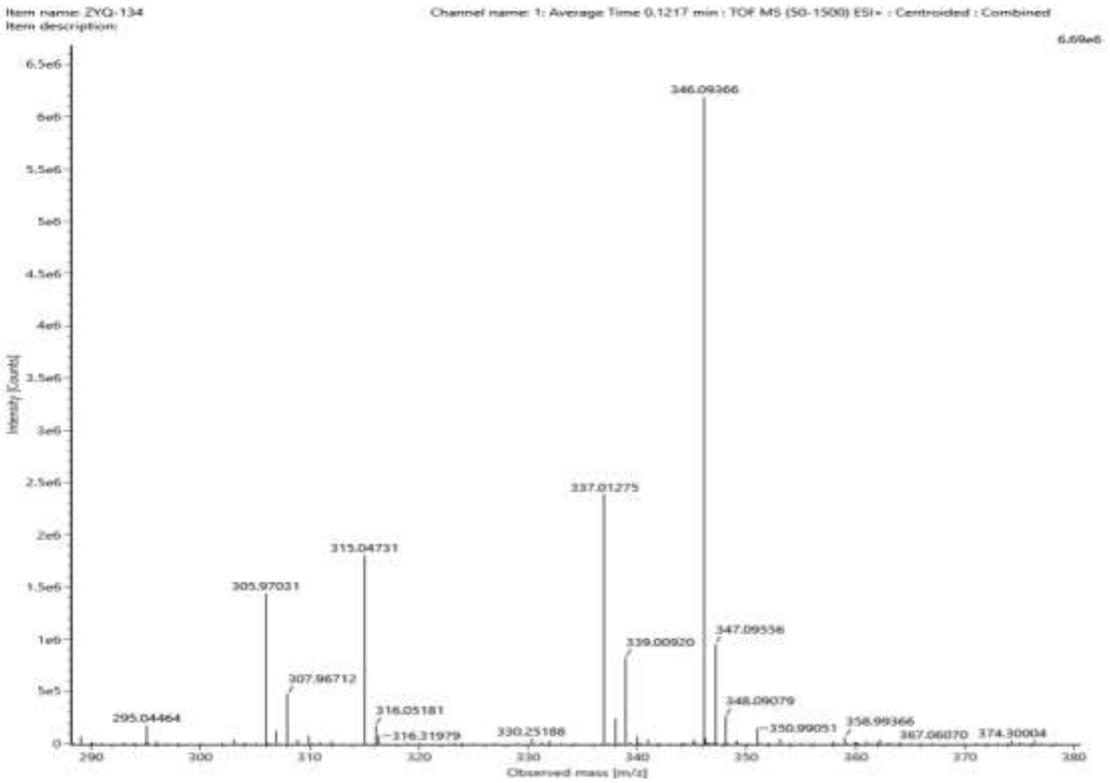


**Figure S17**. HR-MS (ESI) spectrogram of compound **6**

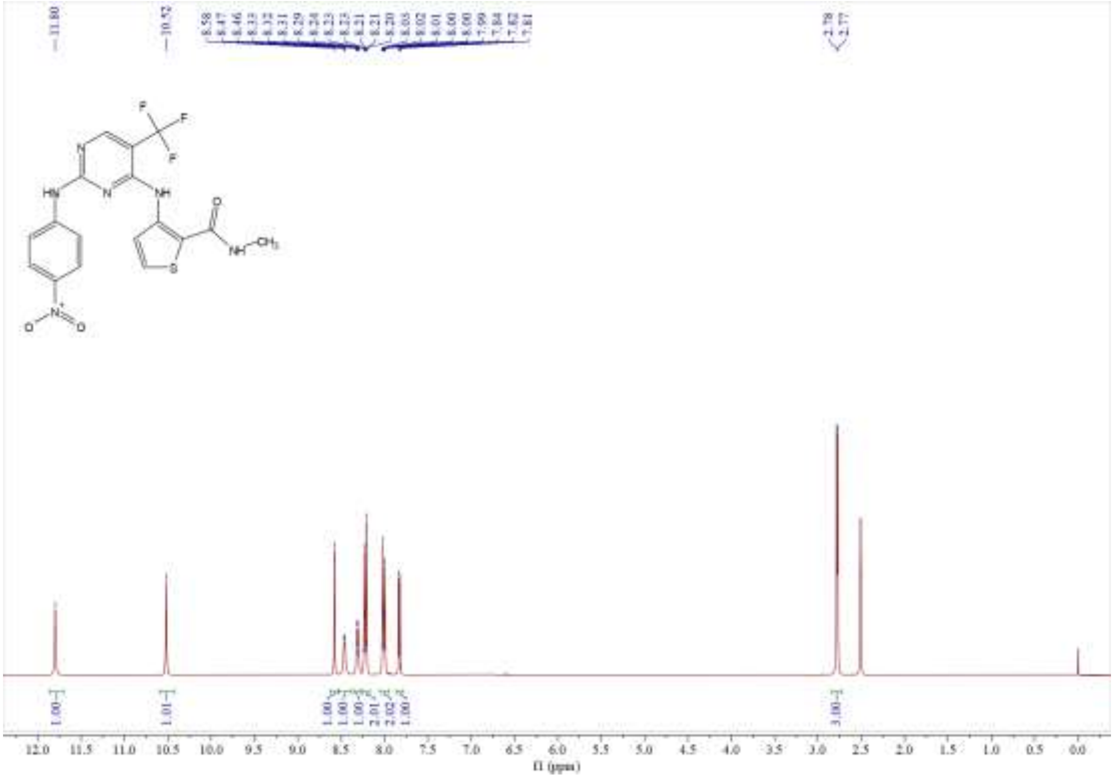


**Figure S18**. $^{1}$H NMR spectrum of compound **7**

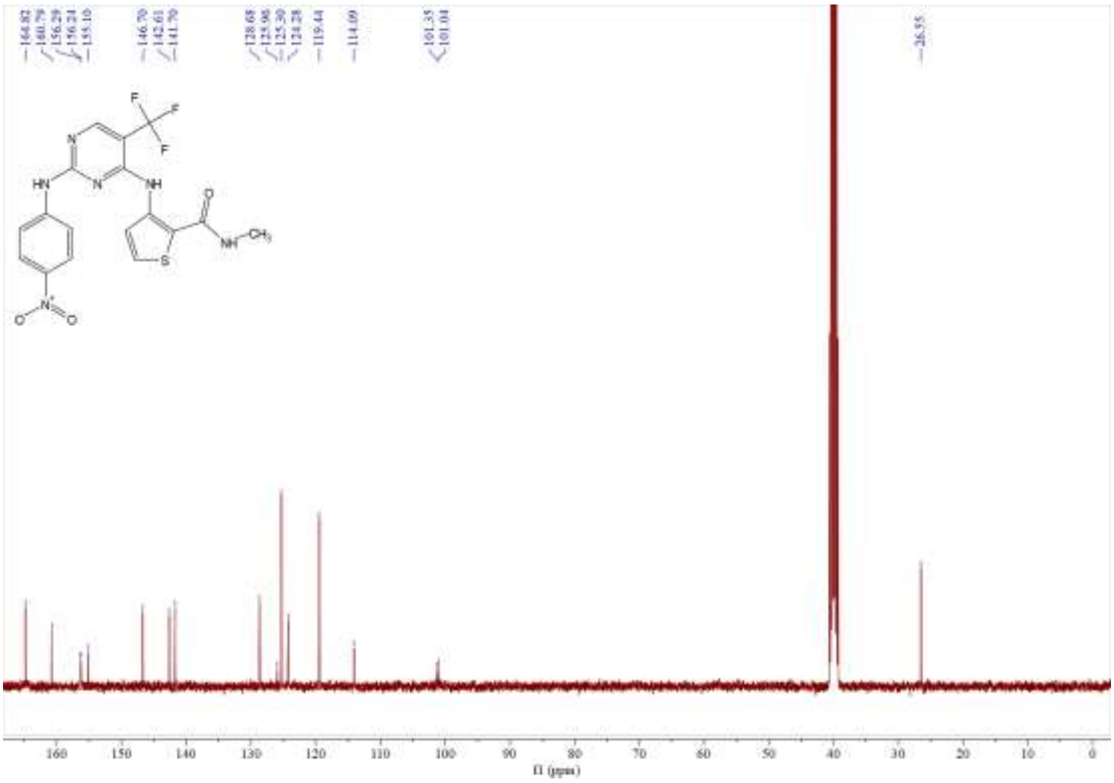


**Figure S19**. $^{13}$C NMR spectrum of compound **7**

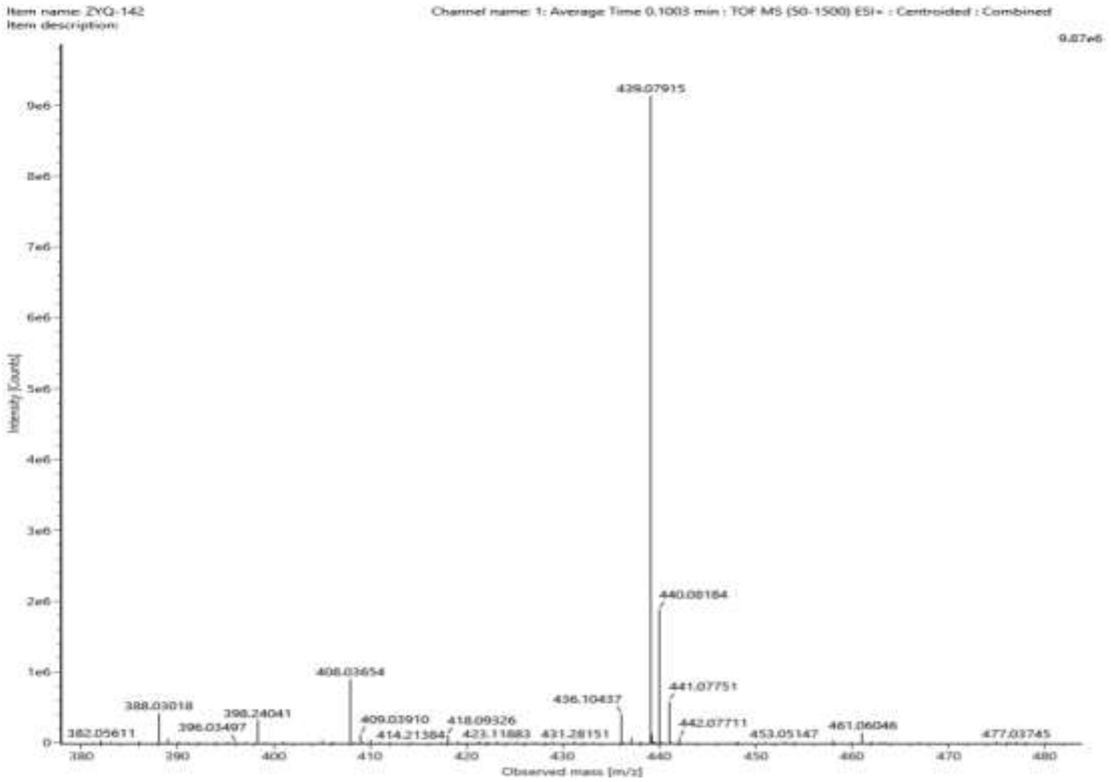


**Figure S20**. HR-MS (ESI) spectrogram of compound **7**

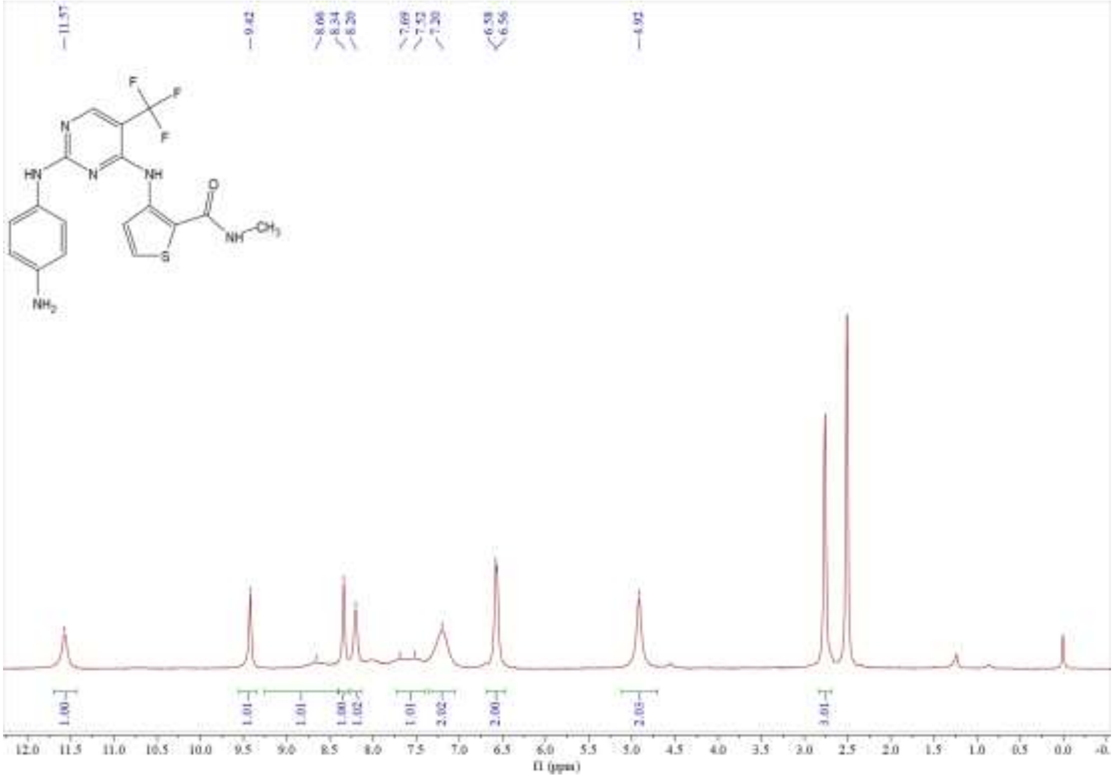

**Figure S21**. $^{1}$H NMR spectrum of compound **8**

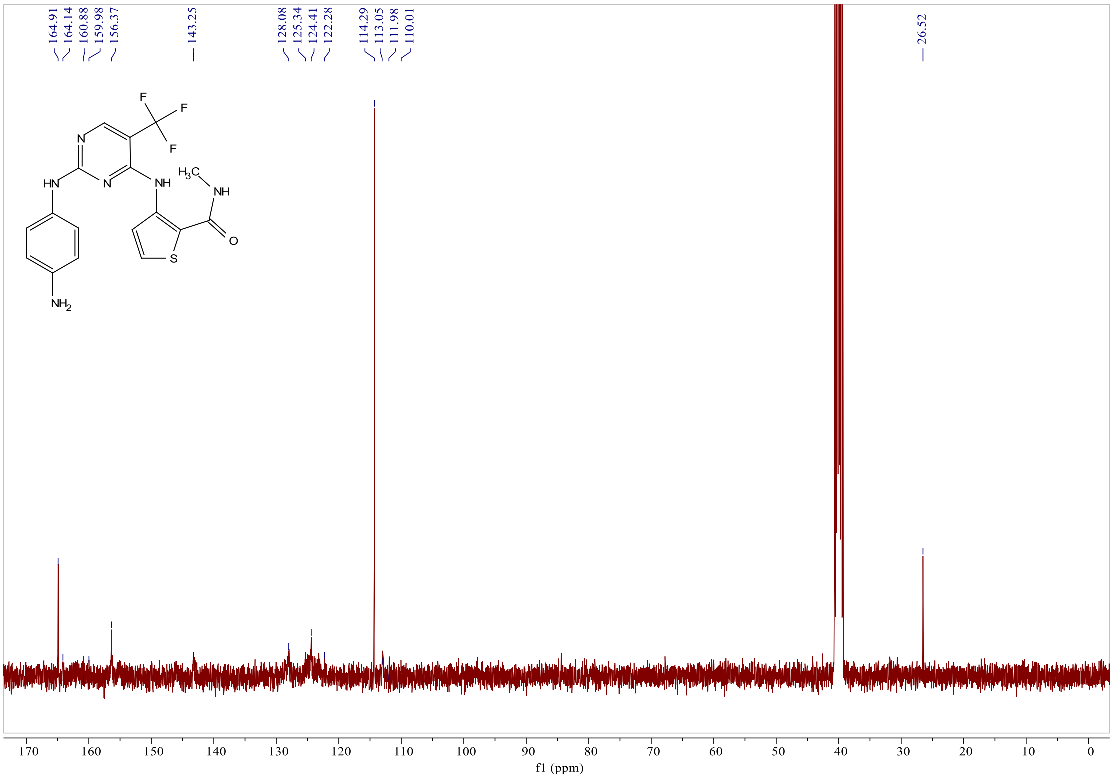

**Figure S22**. $^{13}$C NMR spectrum of compound **8**

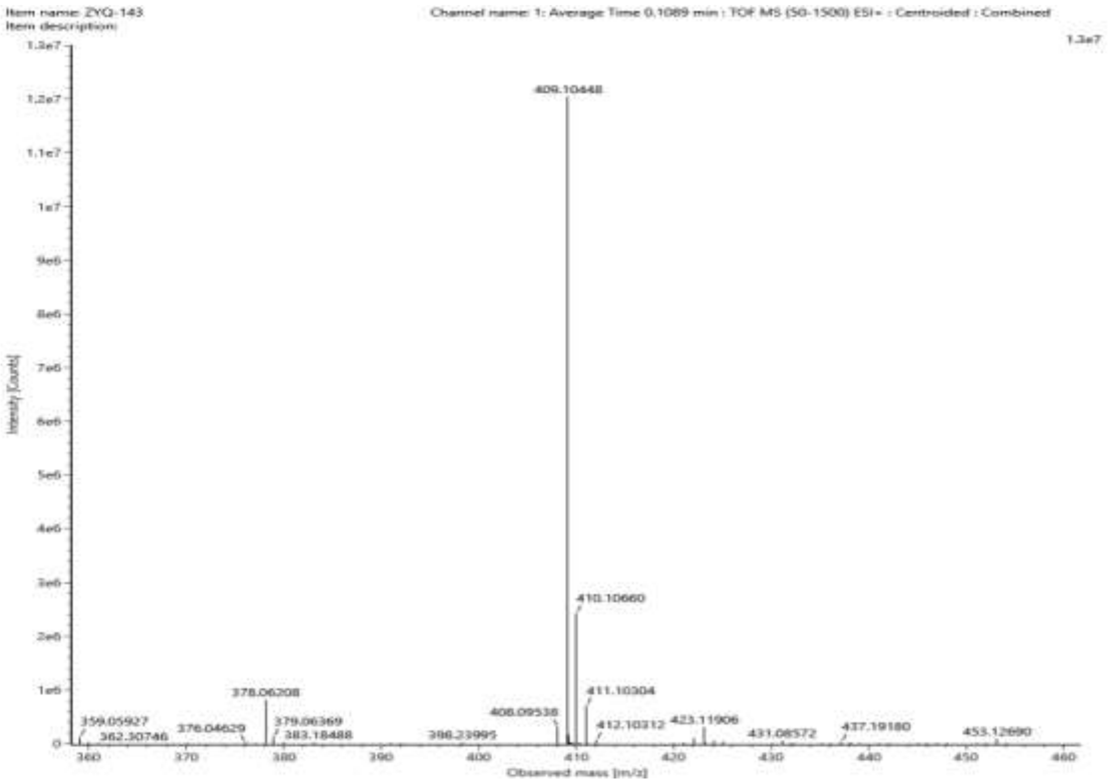


**Figure S23**. HR-MS (ESI) spectrogram of compound **8**

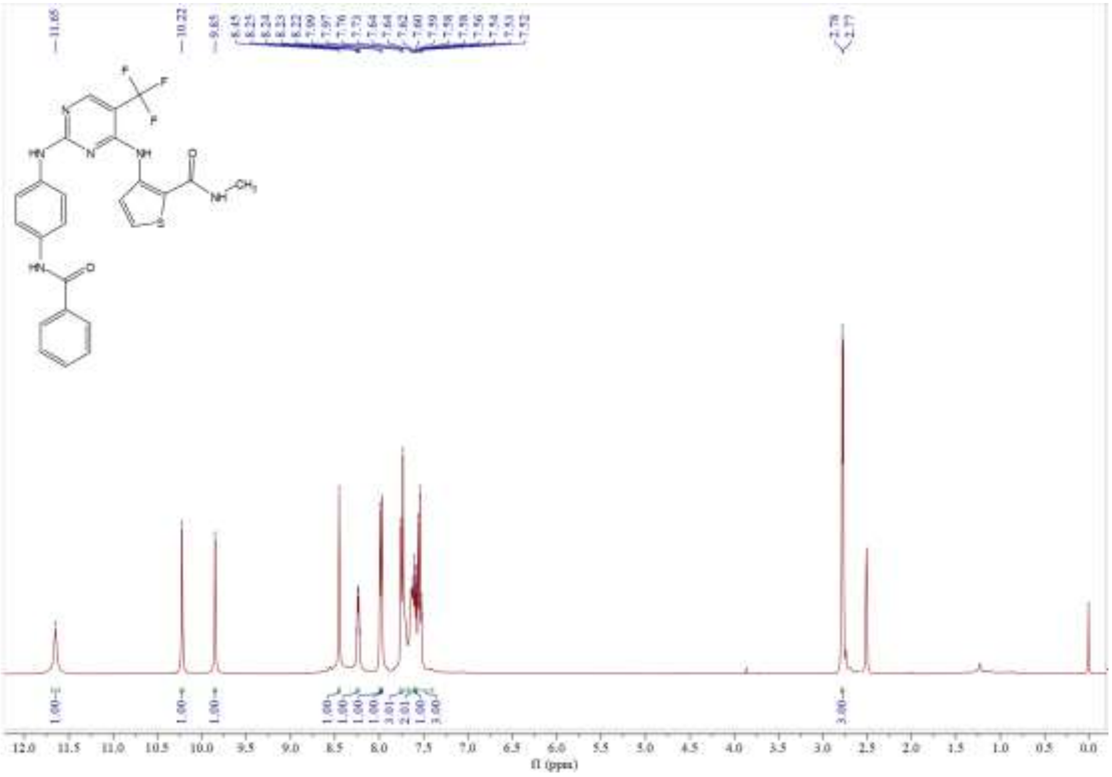


**Figure S24**. $^{1}$H NMR spectrum of compound **9a**

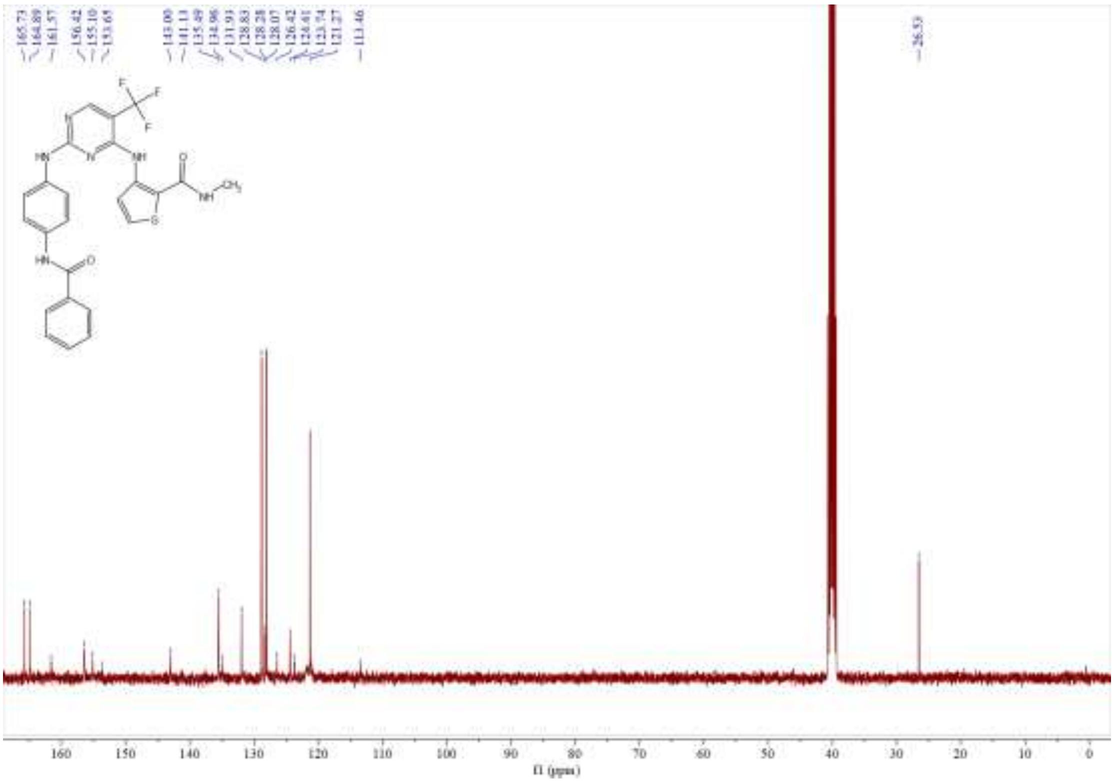


**Figure S25**. $^{13}C$ NMR spectrum of compound **9a**

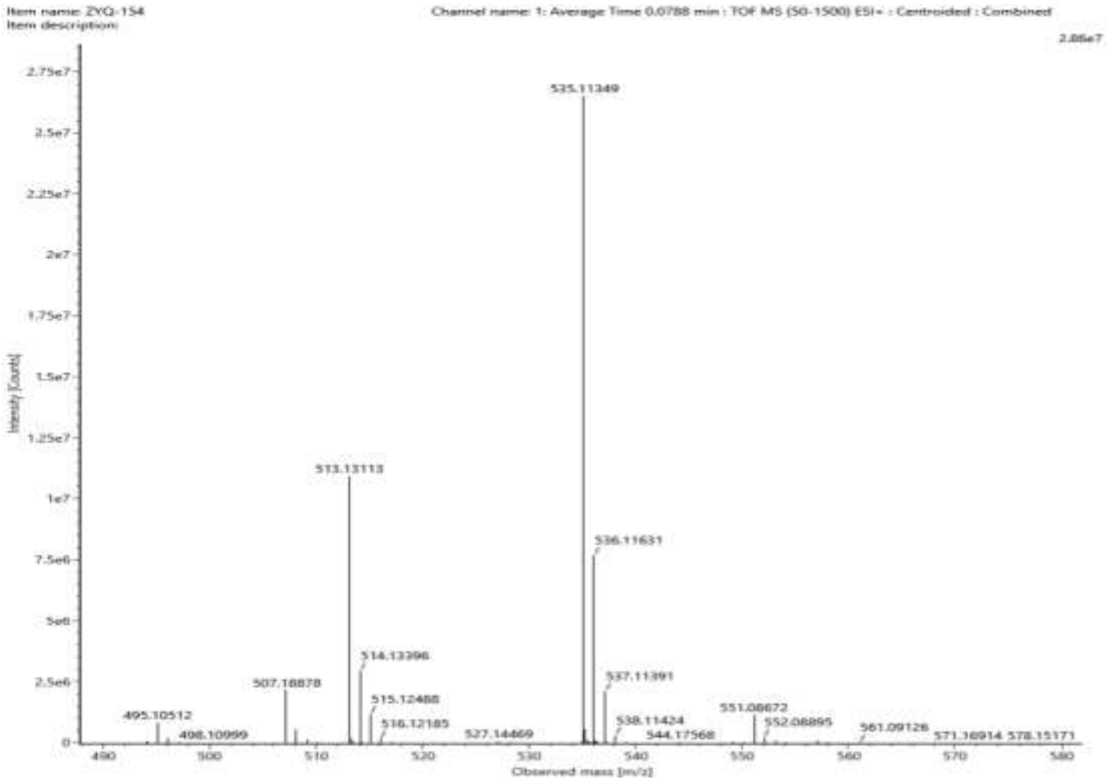


**Figure S26**. HR-MS (ESI) spectrogram of compound **9a**

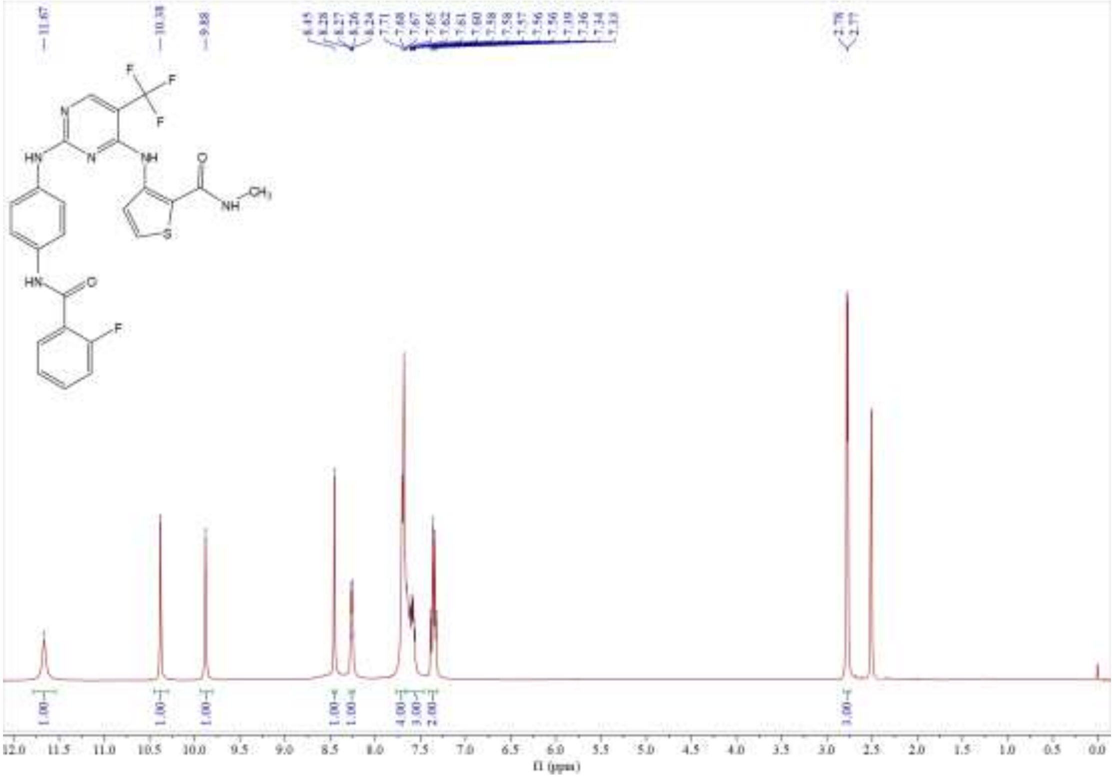

**Figure S27**. $^{1}H$ NMR spectrum of compound **9b**

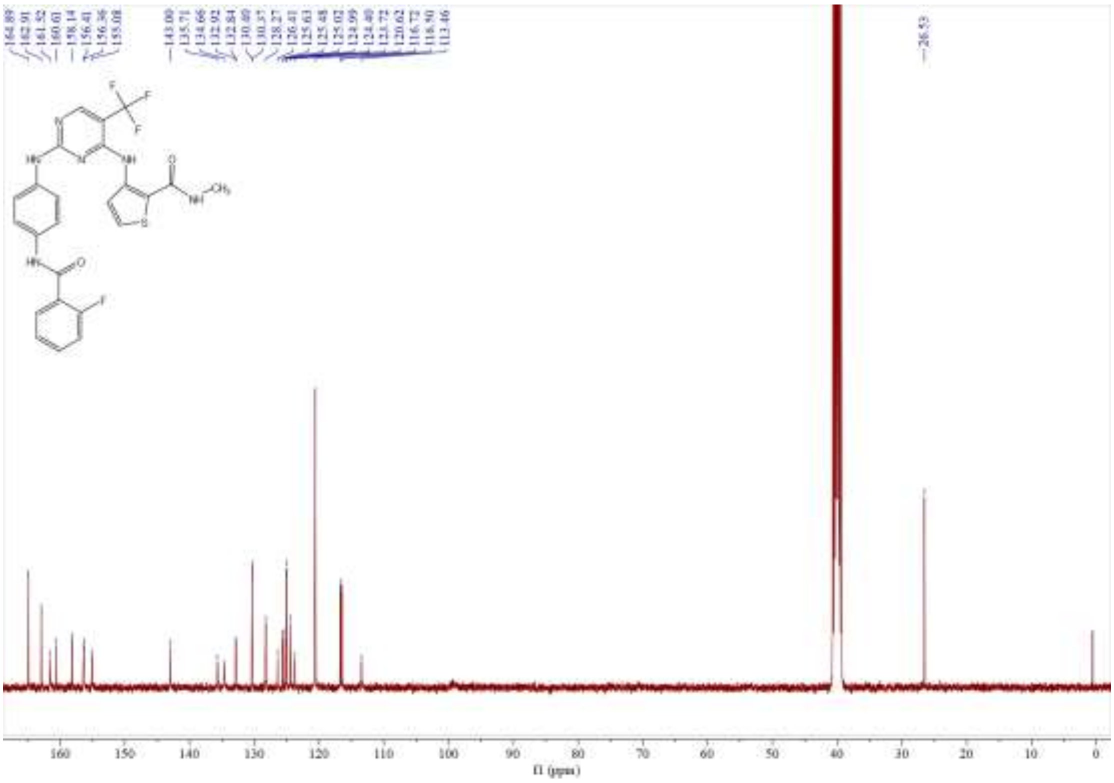

**Figure S28**. $^{13}C$ NMR spectrum of compound **9b**

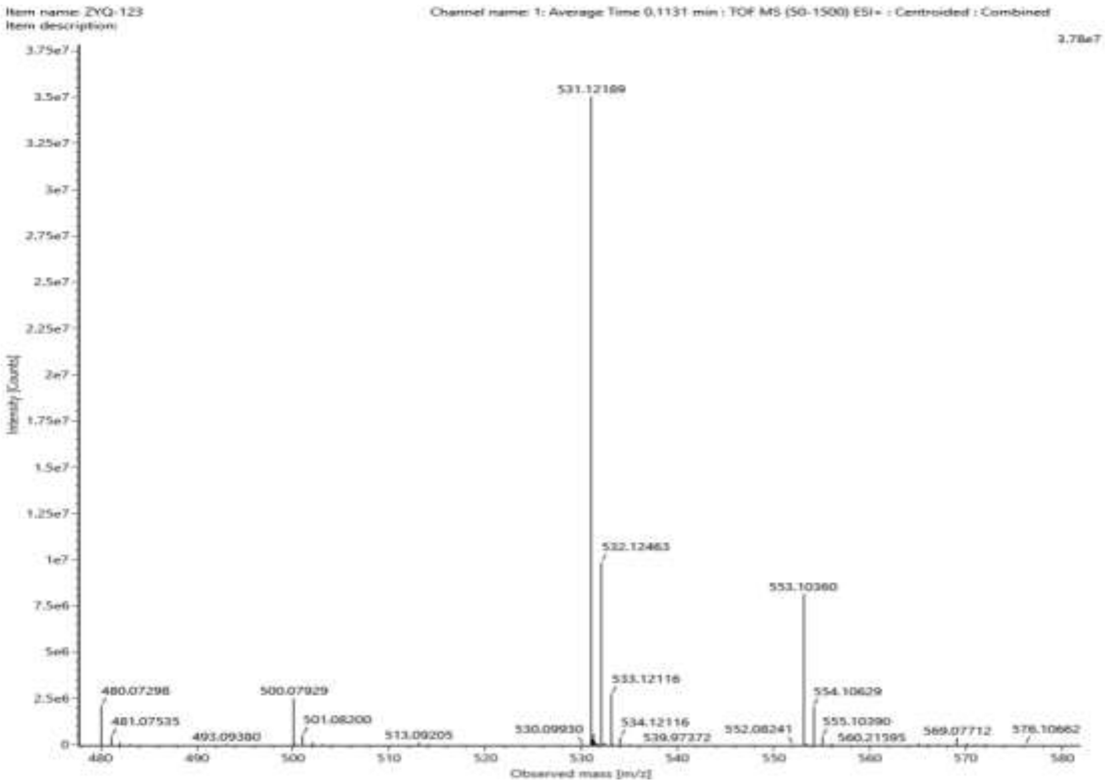

**Figure S29**. HR-MS (ESI) spectrogram of compound **9b**

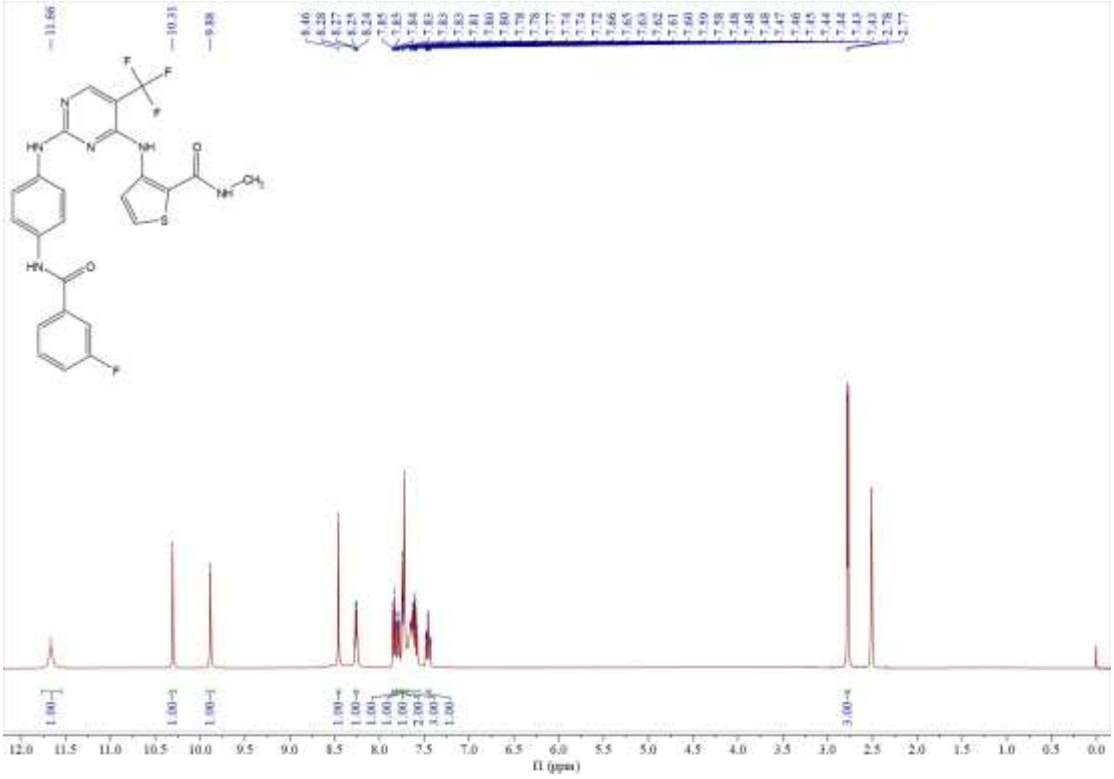

**Figure S30**. $^{1}$H NMR spectrum of compound **9c**

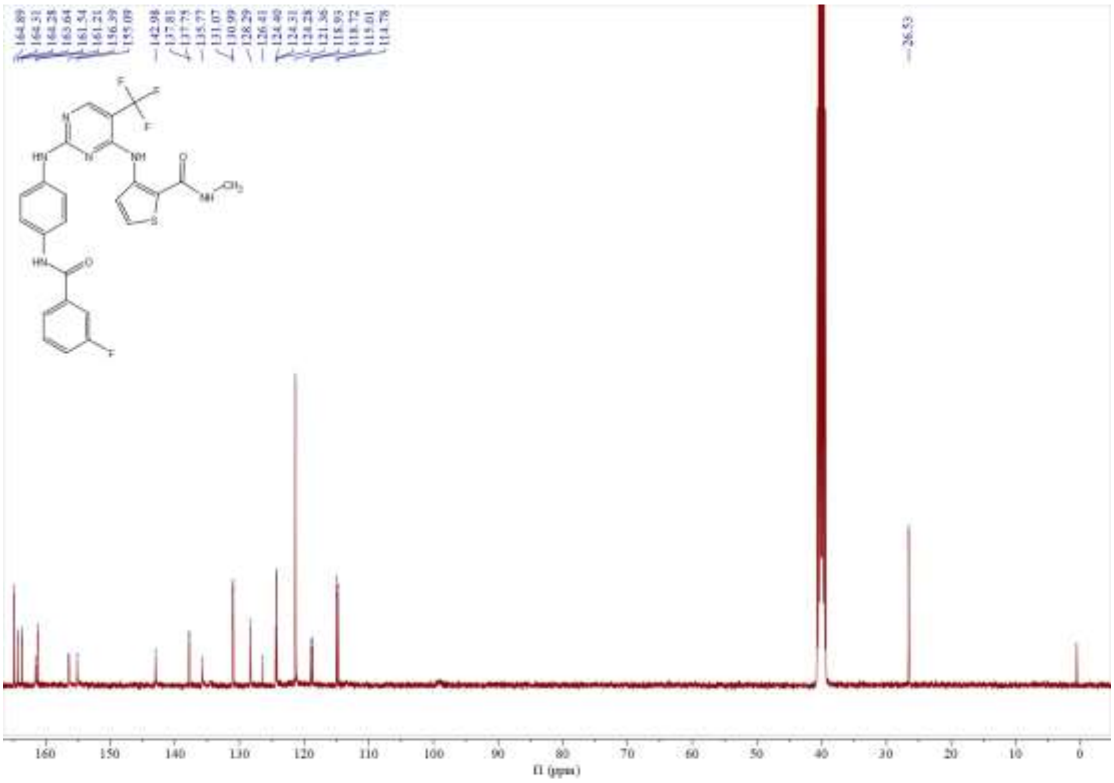

**Figure S31**. $^{13}C$ NMR spectrum of compound **9c**

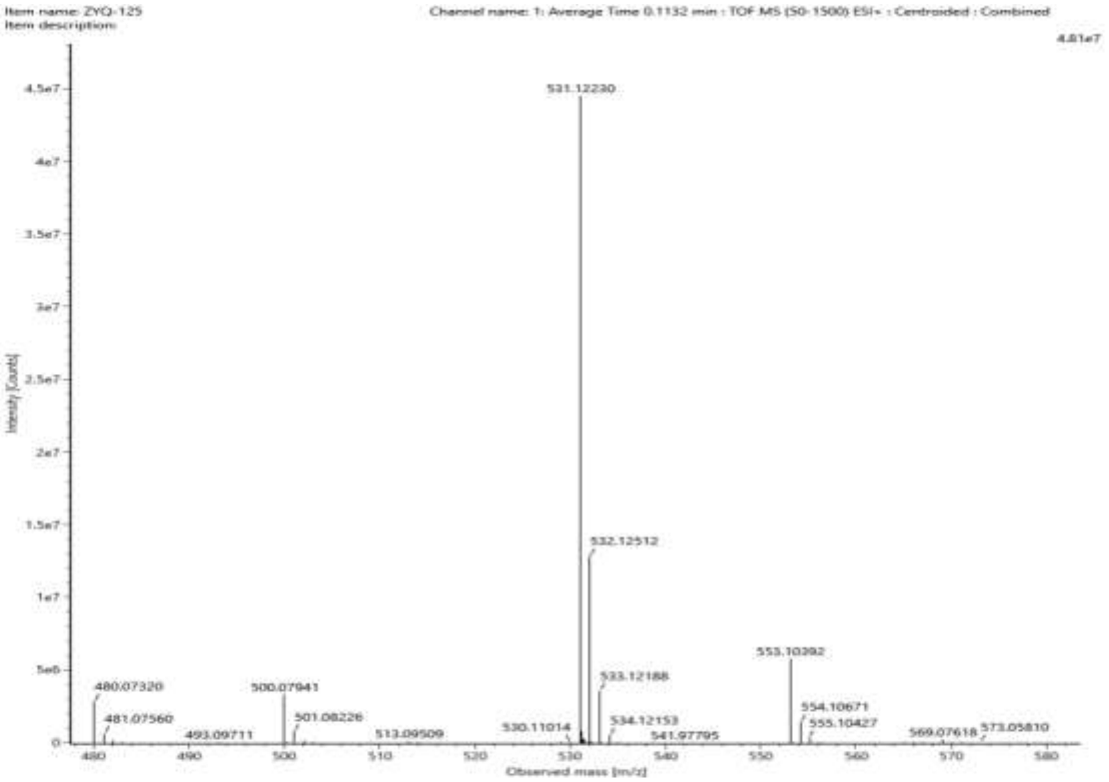


**Figure S32**. HR-MS (ESI) spectrogram of compound **9c**

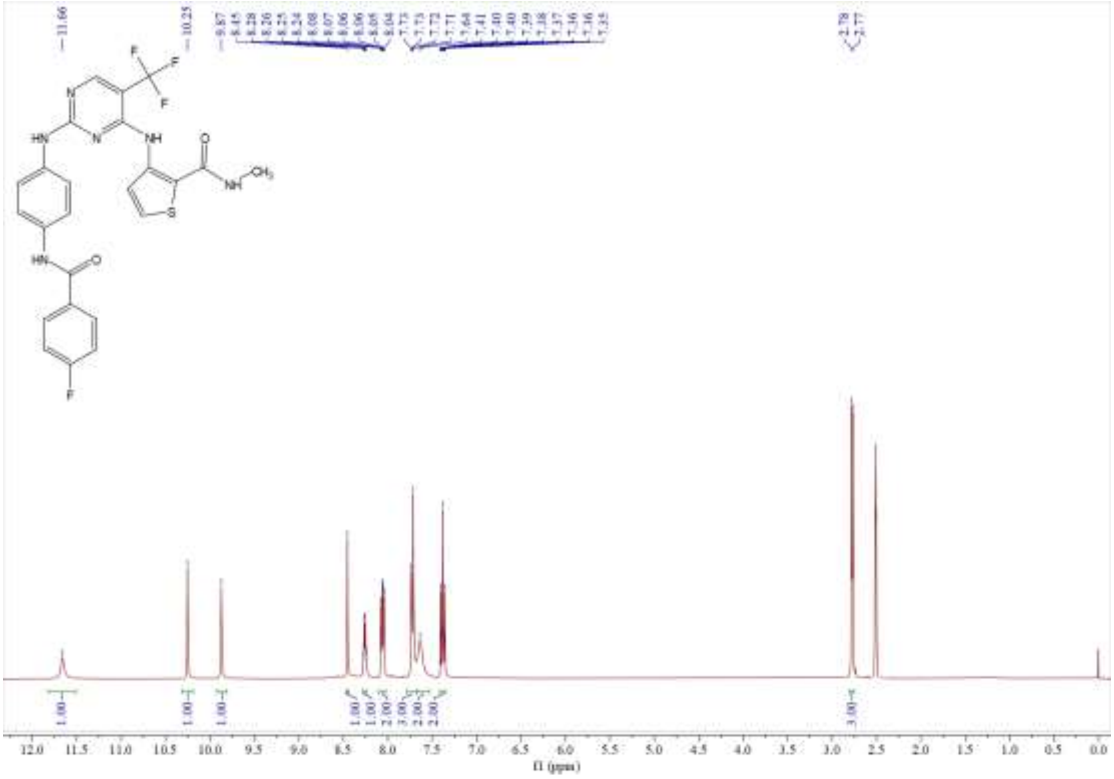

**Figure S33**. $^{1}$H NMR spectrum of compound **9d**

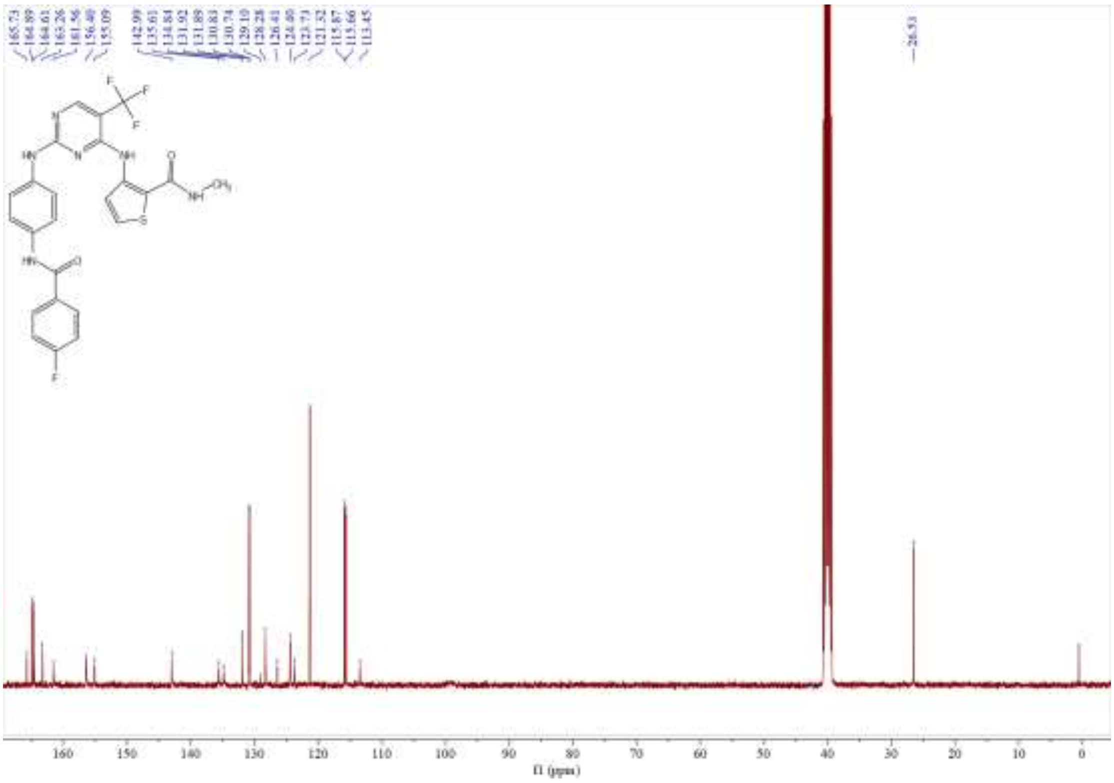

**Figure S34**. $^{13}$C NMR spectrum of compound **9d**

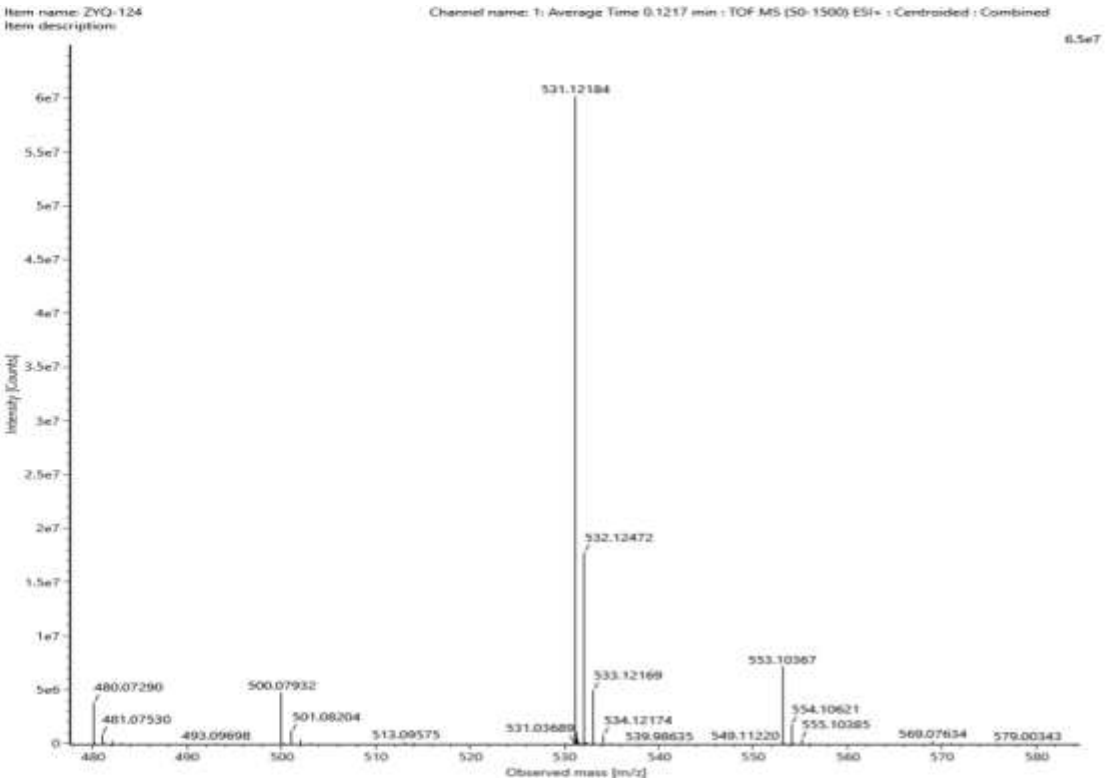


**Figure S35**. HR-MS (ESI) spectrogram of compound **9d**

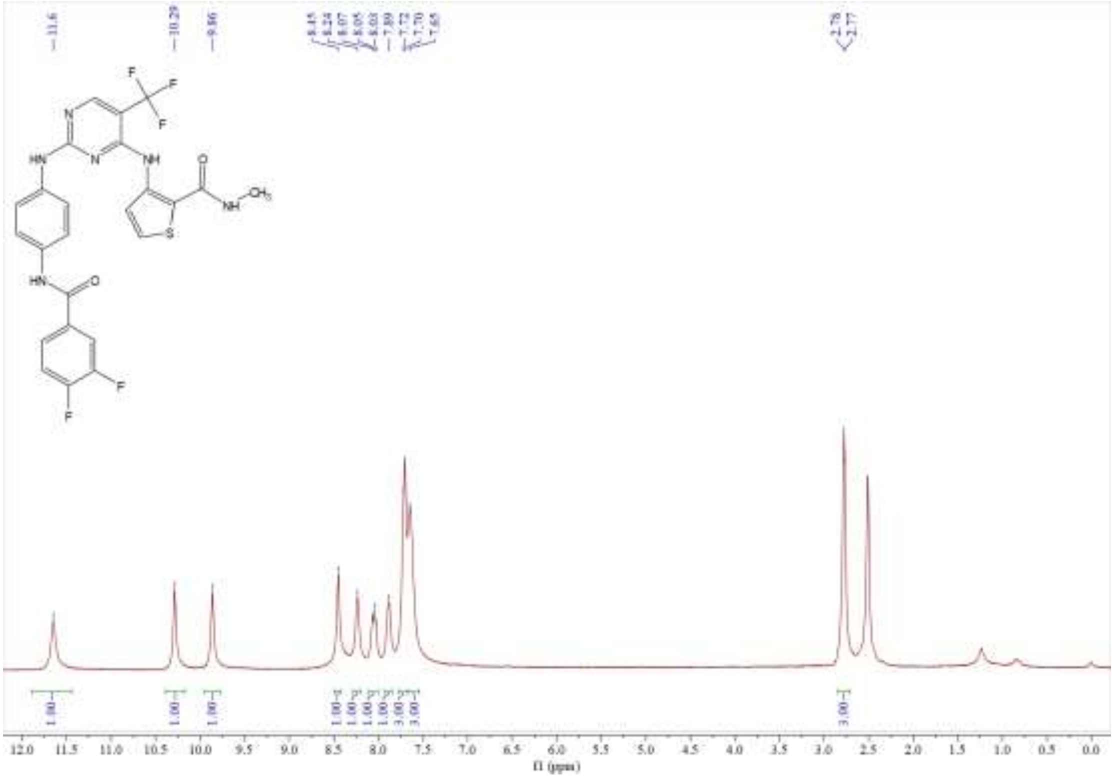


**Figure S36**. $^{1}$H NMR spectrum of compound **9e**

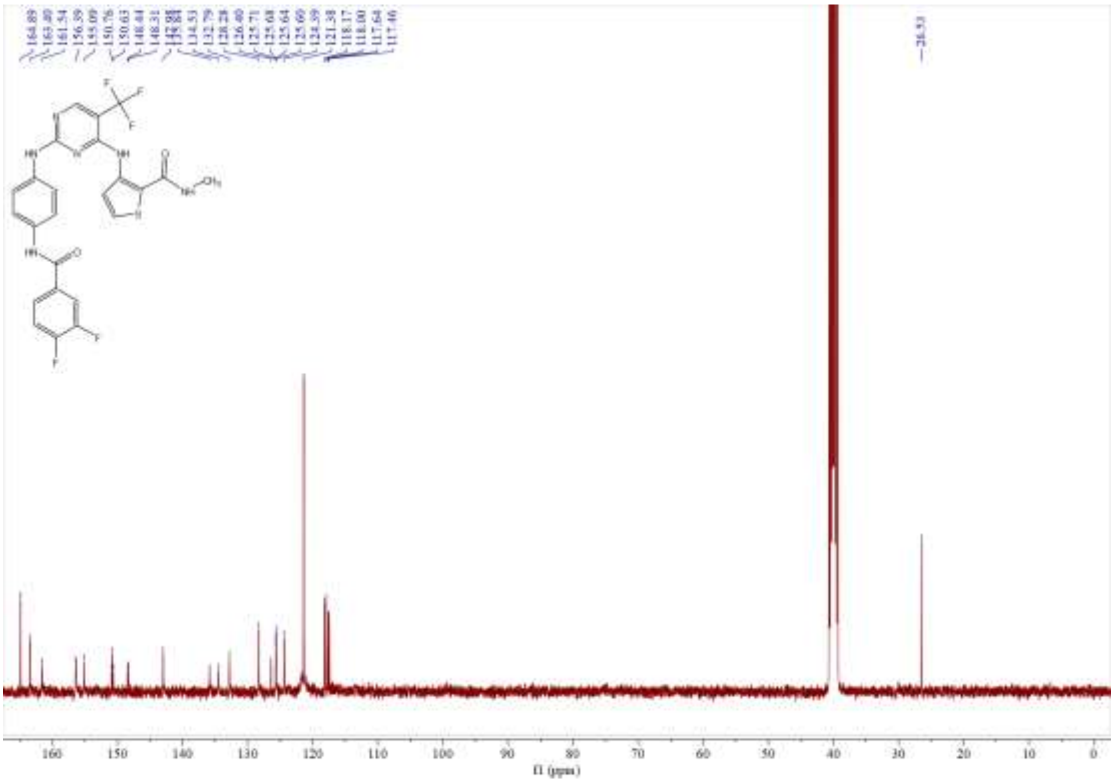


**Figure S37**. $^{13}$C NMR spectrum of compound **9e**

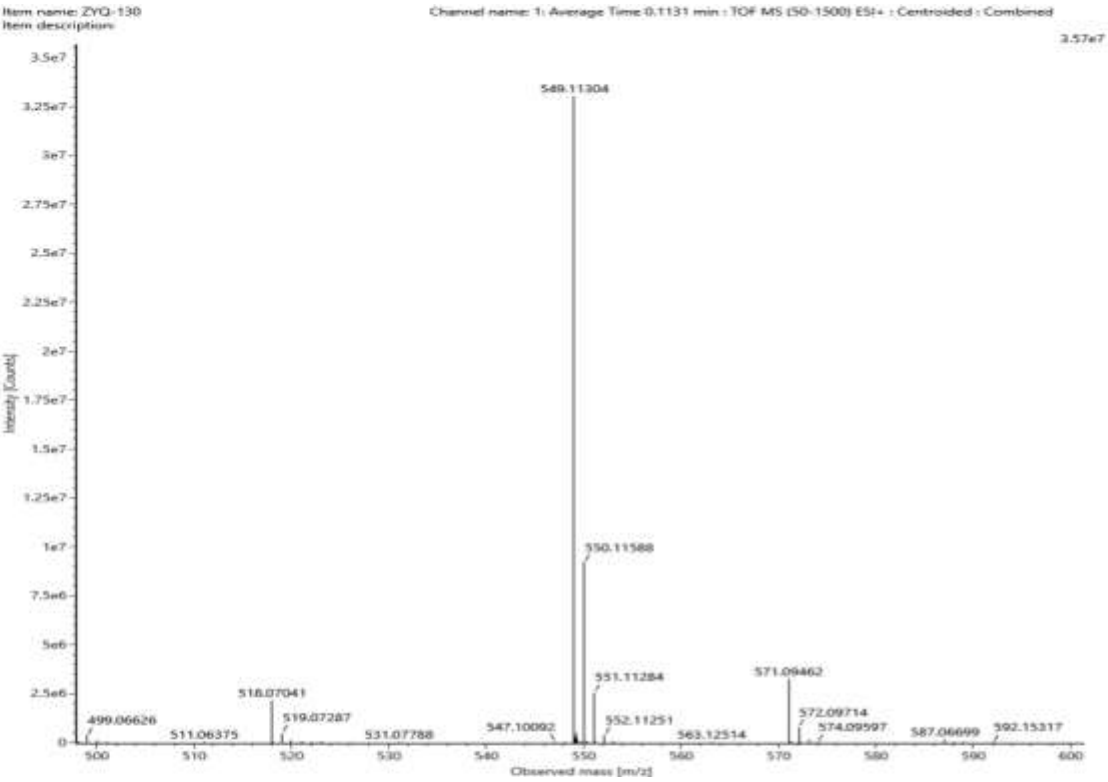


**Figure S38**. HR-MS (ESI) spectrogram of compound **9e**

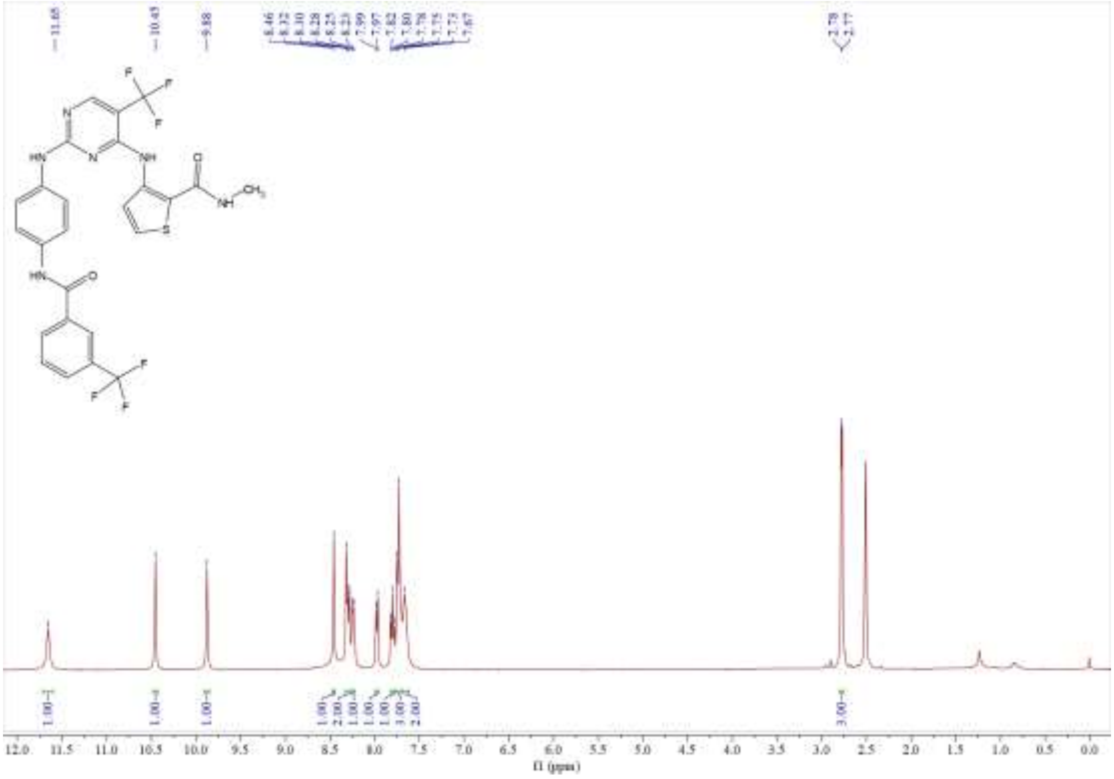

**Figure S39**. $^{1}$H NMR spectrum of compound **9f**

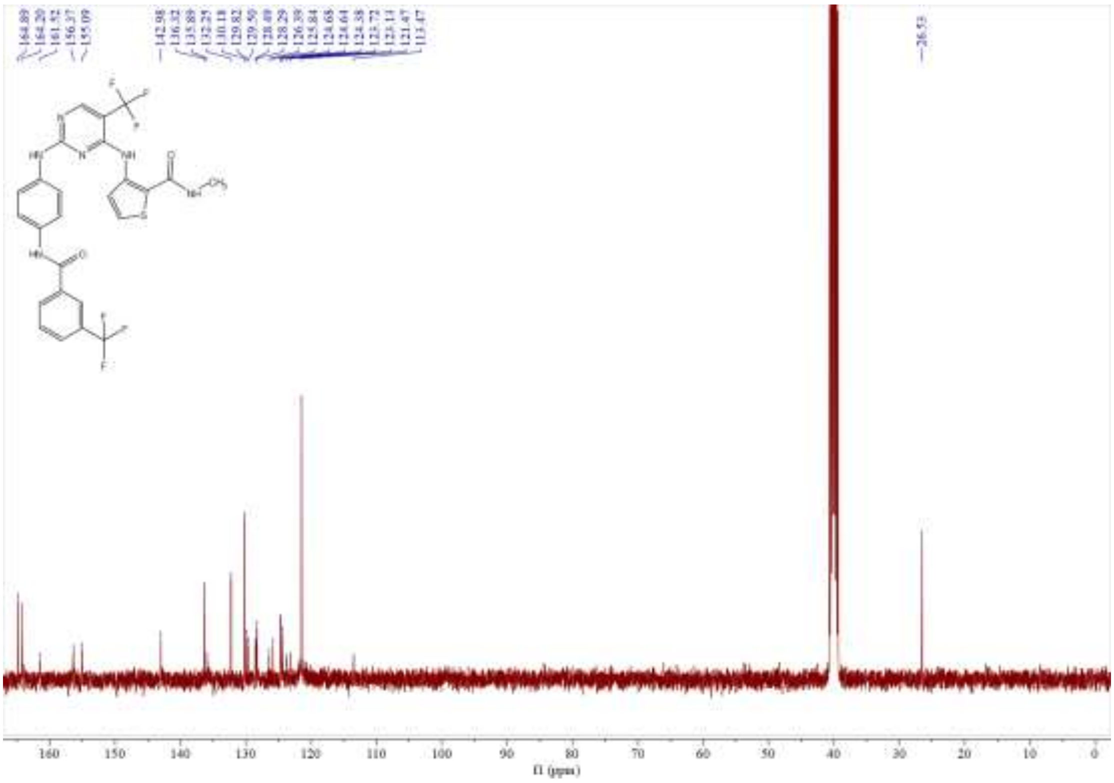

**Figure S40**. $^{13}$C NMR spectrum of compound **9f**

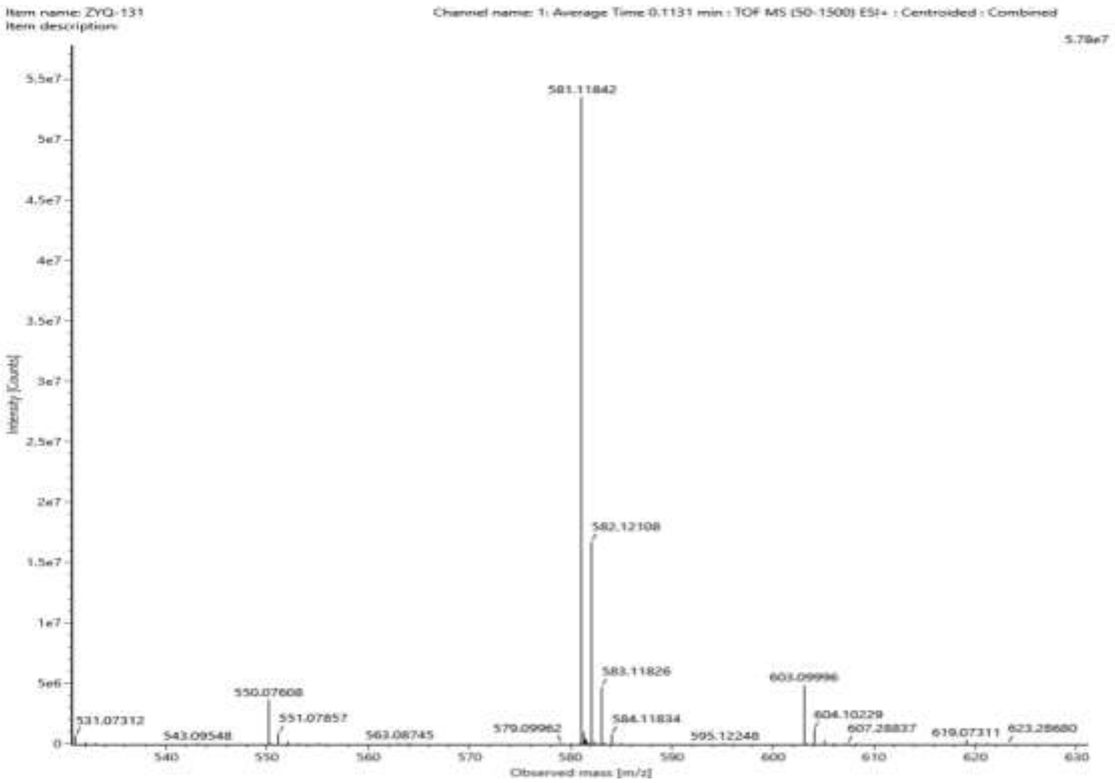

**Figure S41**. HR-MS (ESI) spectrogram of compound **9f**

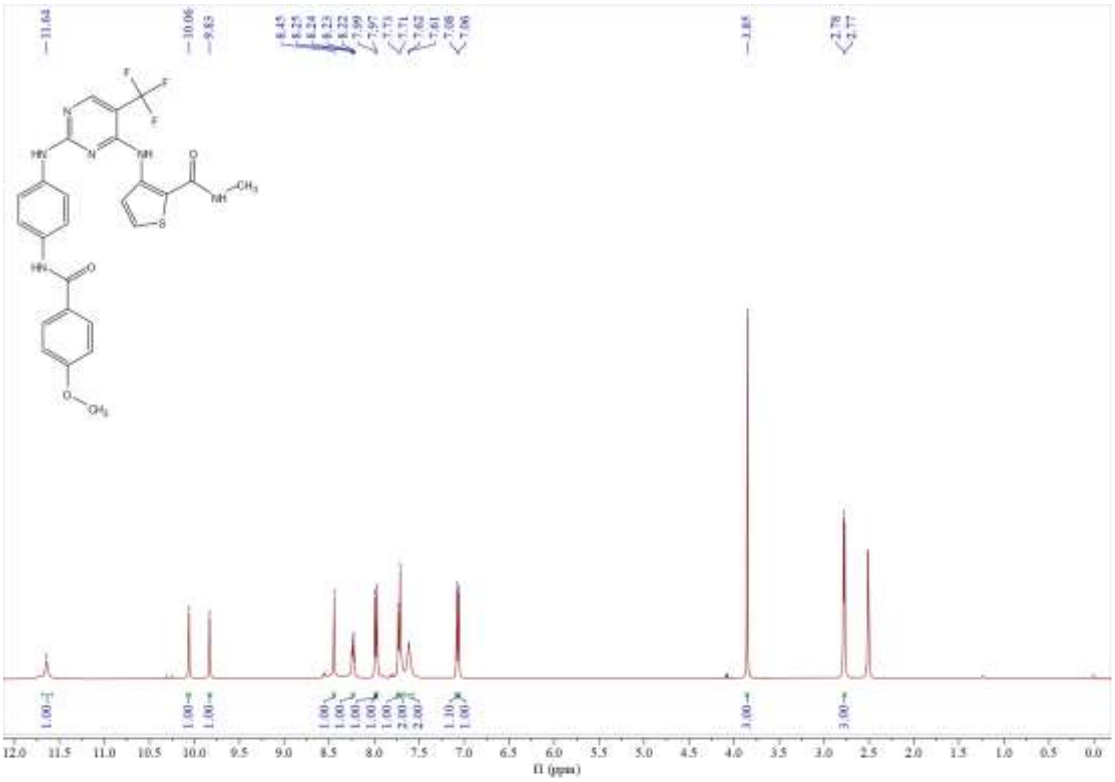

**Figure S42**. $^{1}$H NMR spectrum of compound **9g**

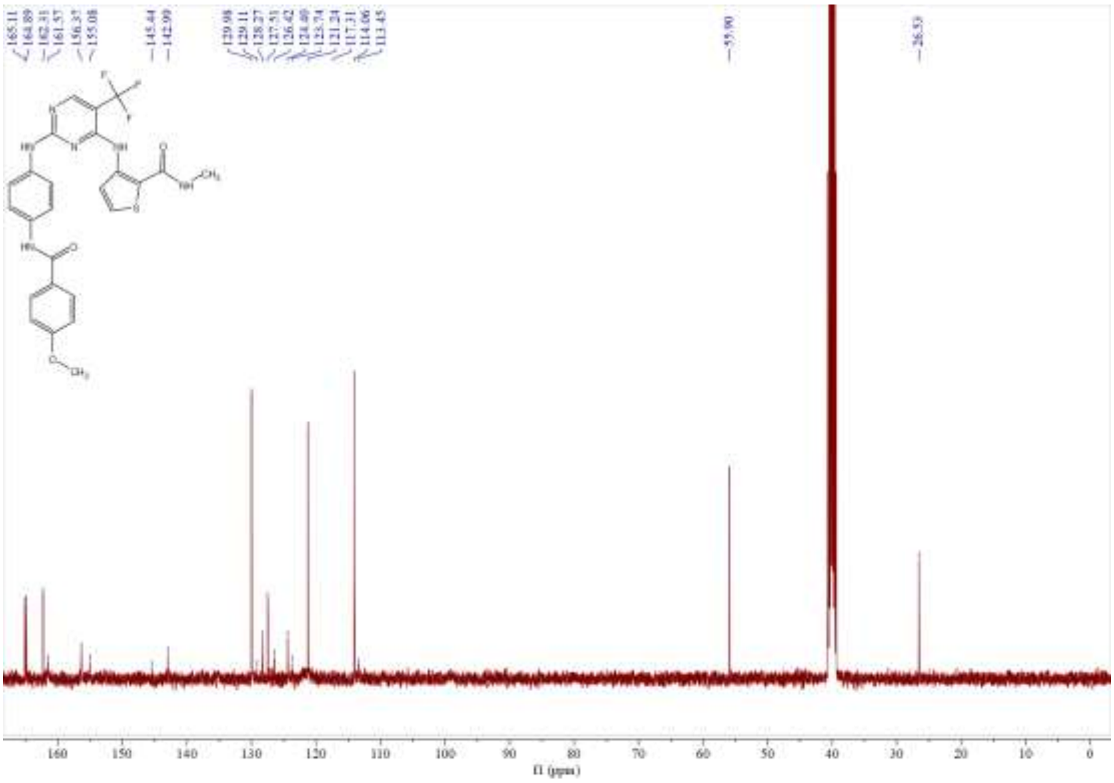


**Figure S43**. $^{13}$C NMR spectrum of compound **9g**

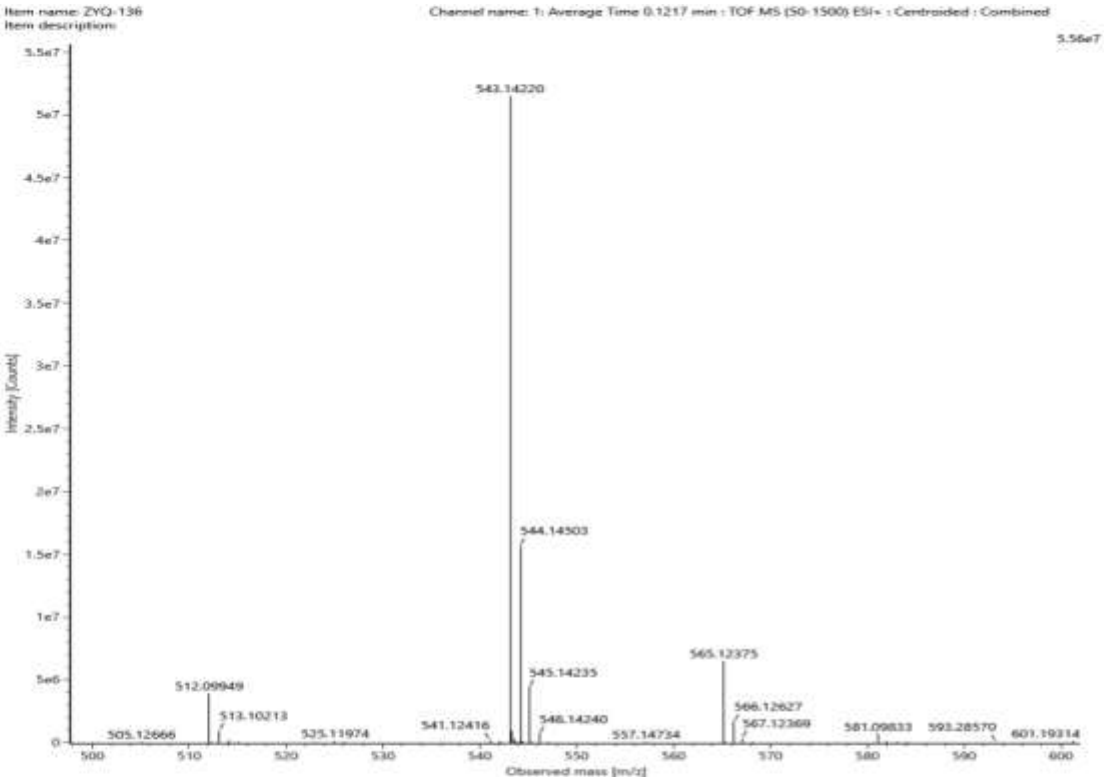


**Figure S44**. HR-MS (ESI) spectrogram of compound **9g**

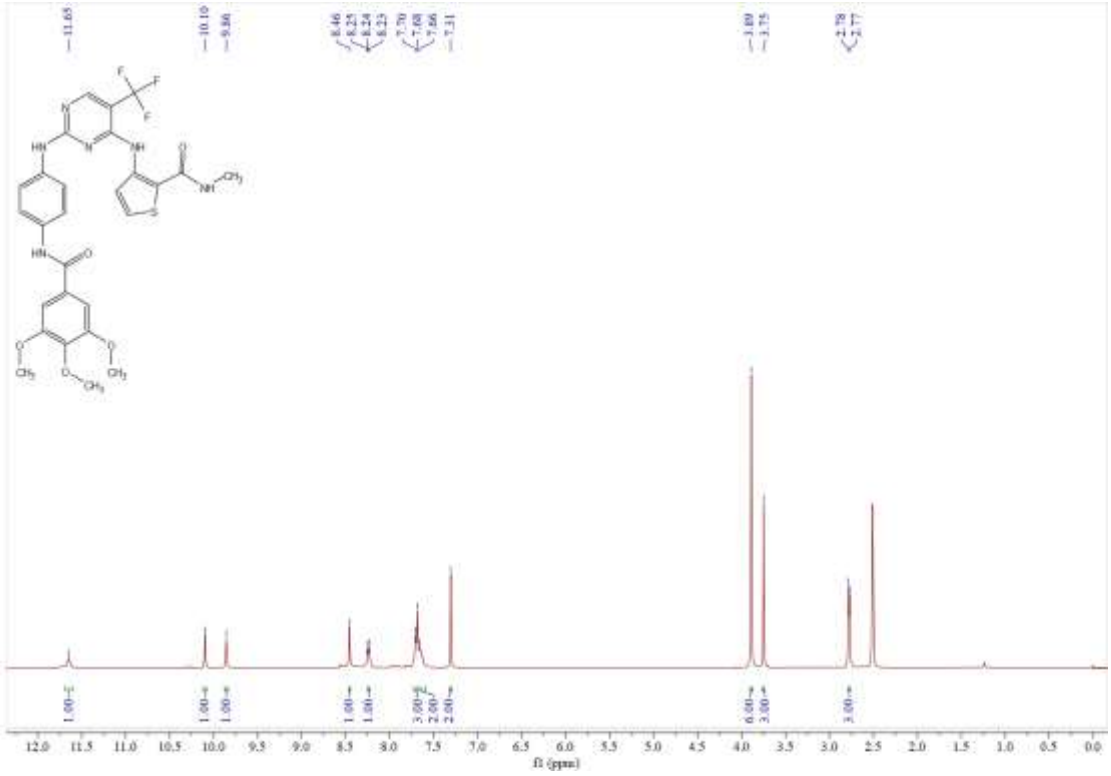

**Figure S45**. $^{1}H$ NMR spectrum of compound **9h**

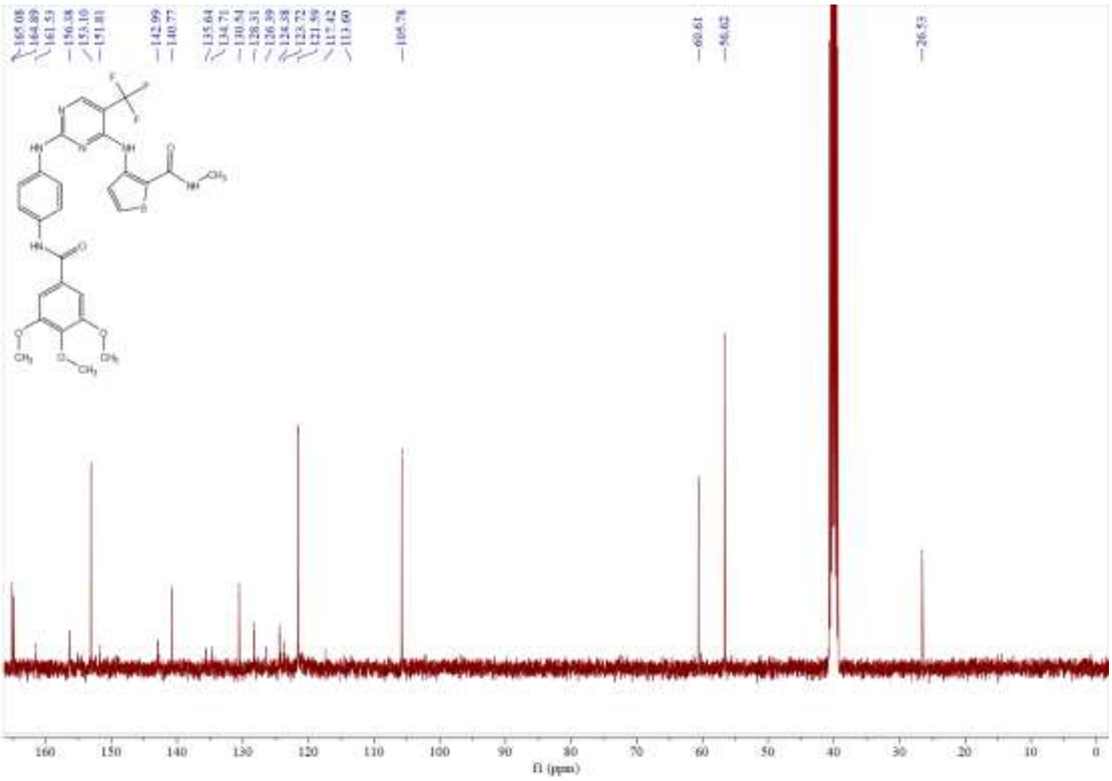

**Figure S46**. $^{13}C$ NMR spectrum of compound **9h**

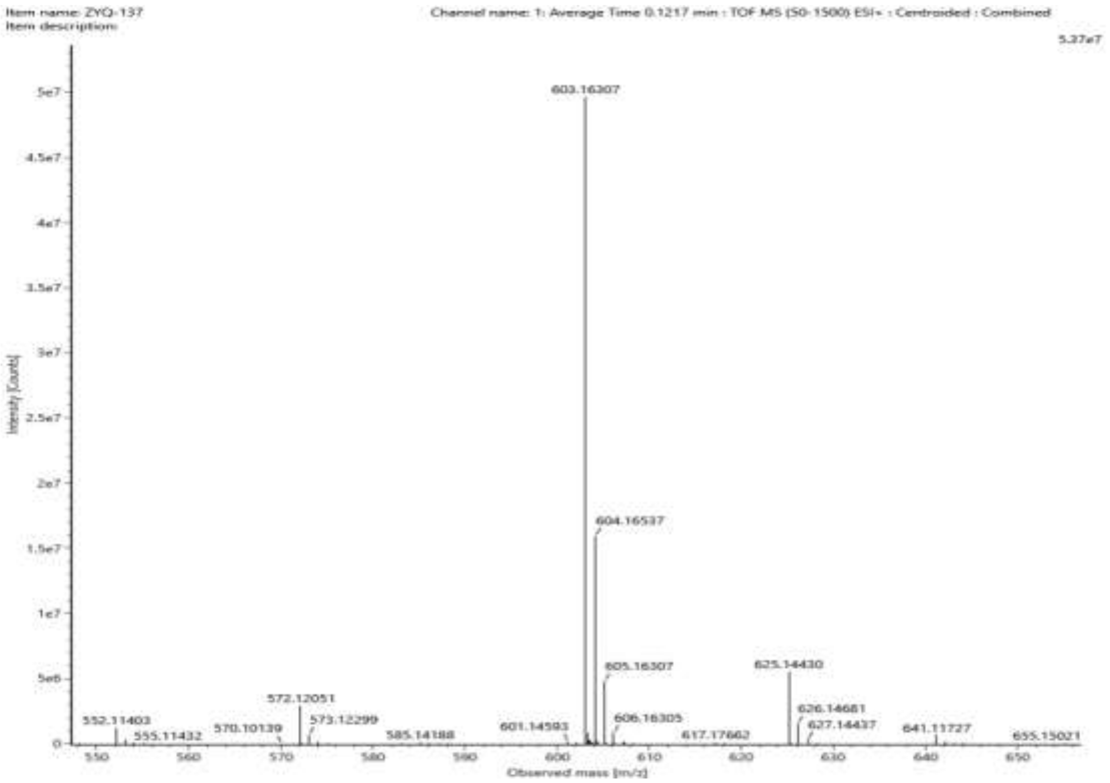

Figure S47. HR-MS (ESI) spectrogram of compound **9h**

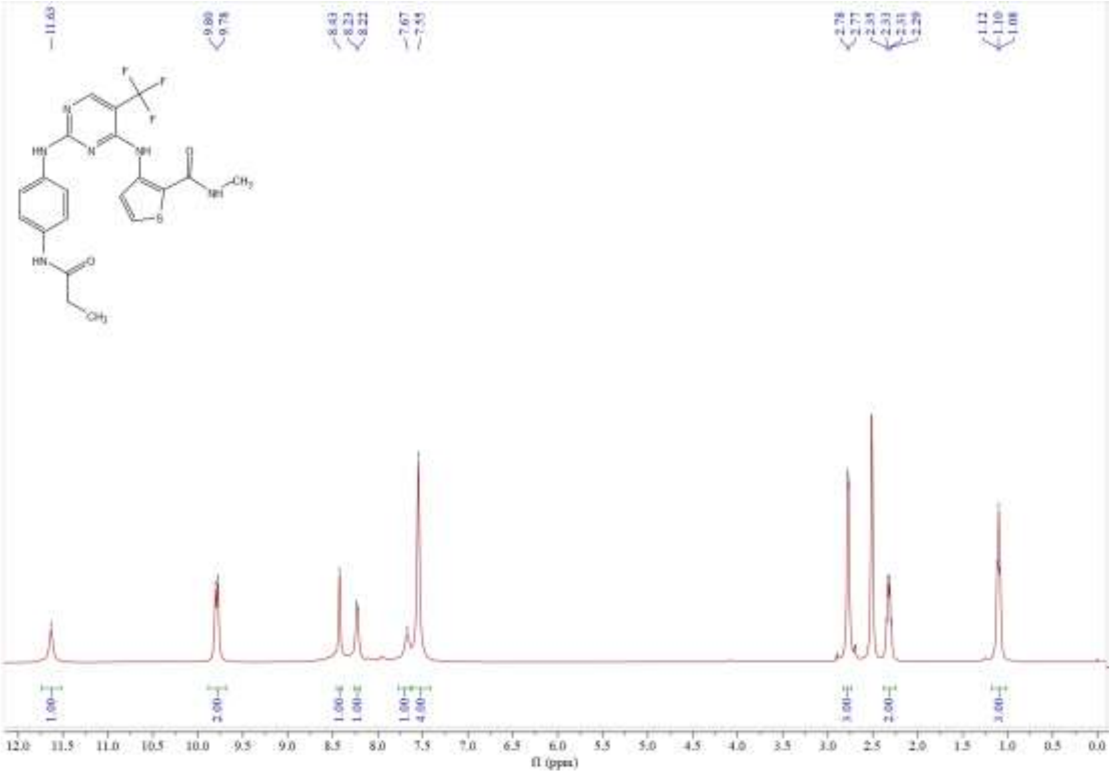

Figure S48. $^1$H NMR spectrum of compound **9i**

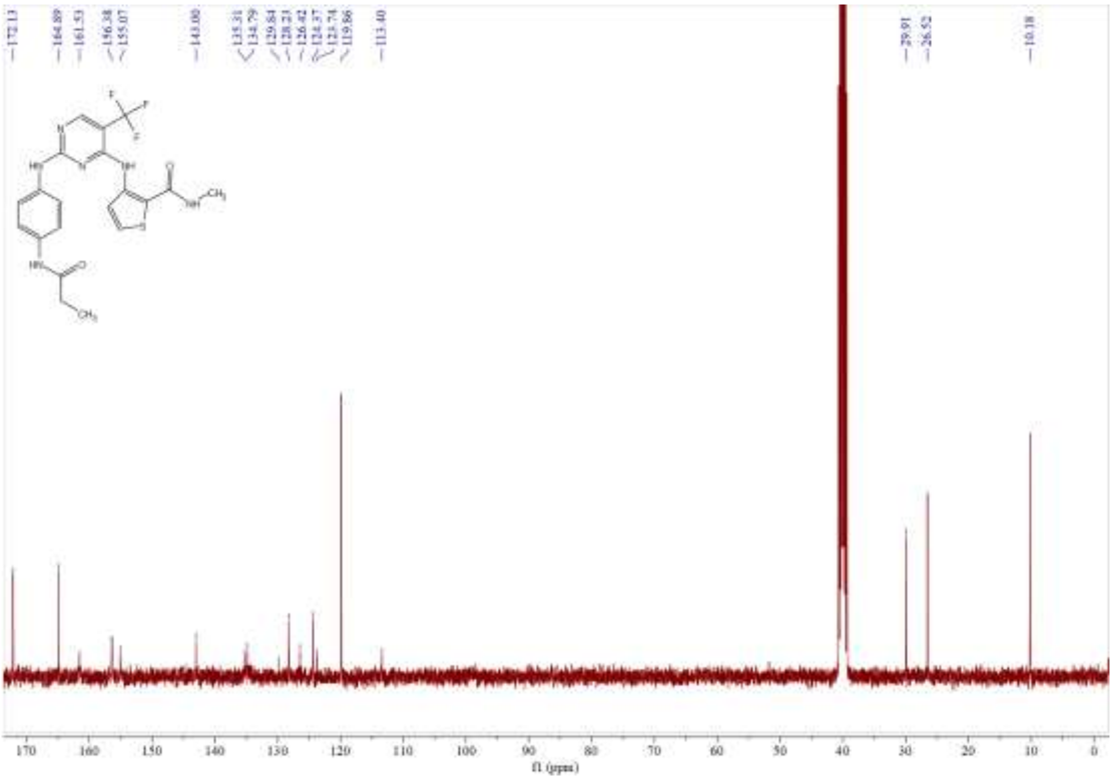


**Figure S49**. $^{13}$C NMR spectrum of compound **9i**

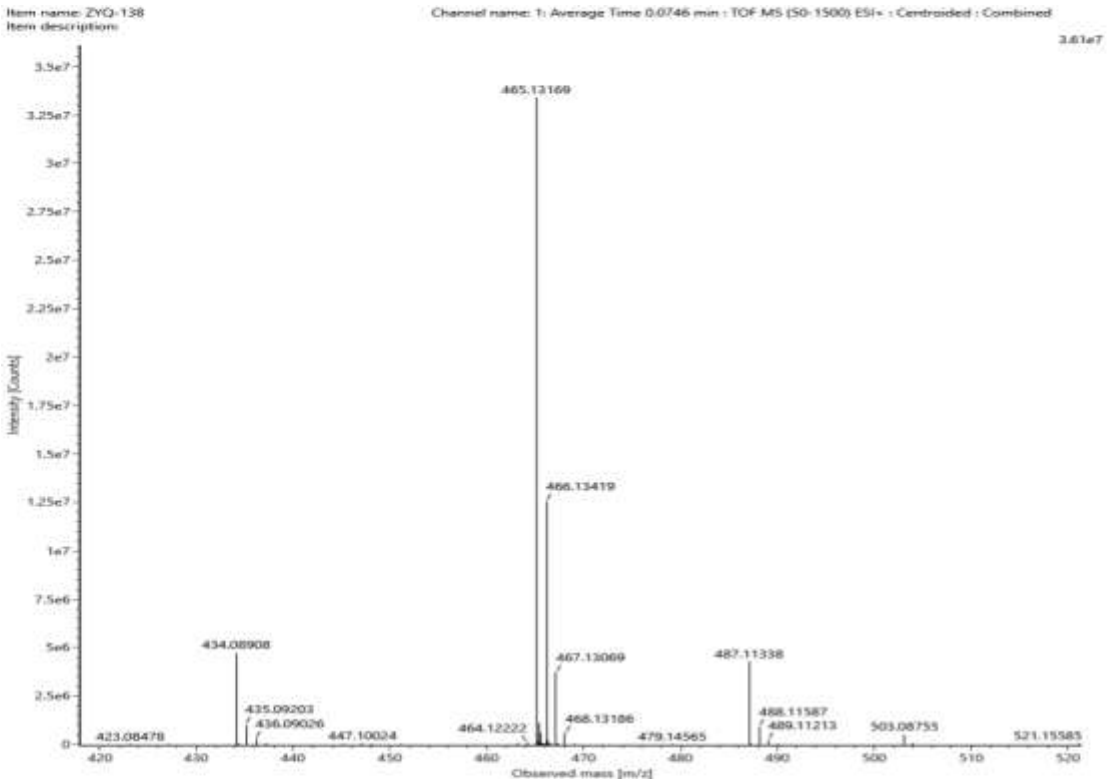


**Figure S50**. HR-MS (ESI) spectrogram of compound **9i**

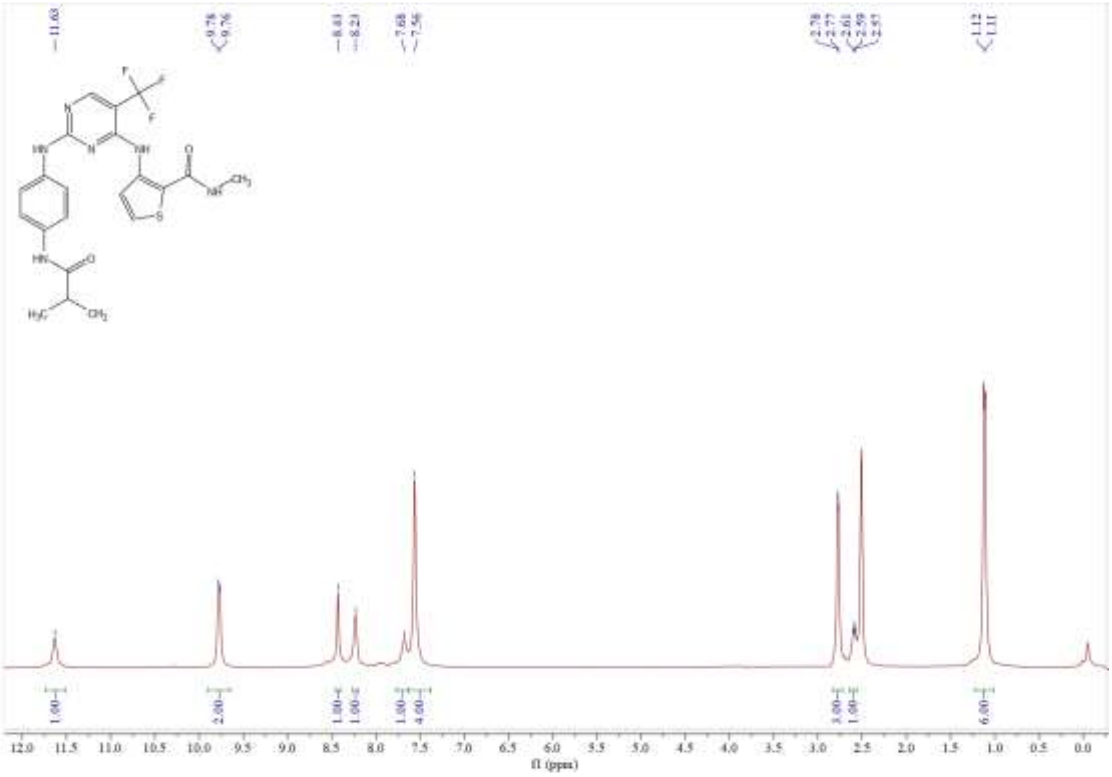

**Figure S51**. $^{1}H$ NMR spectrum of compound **9j**

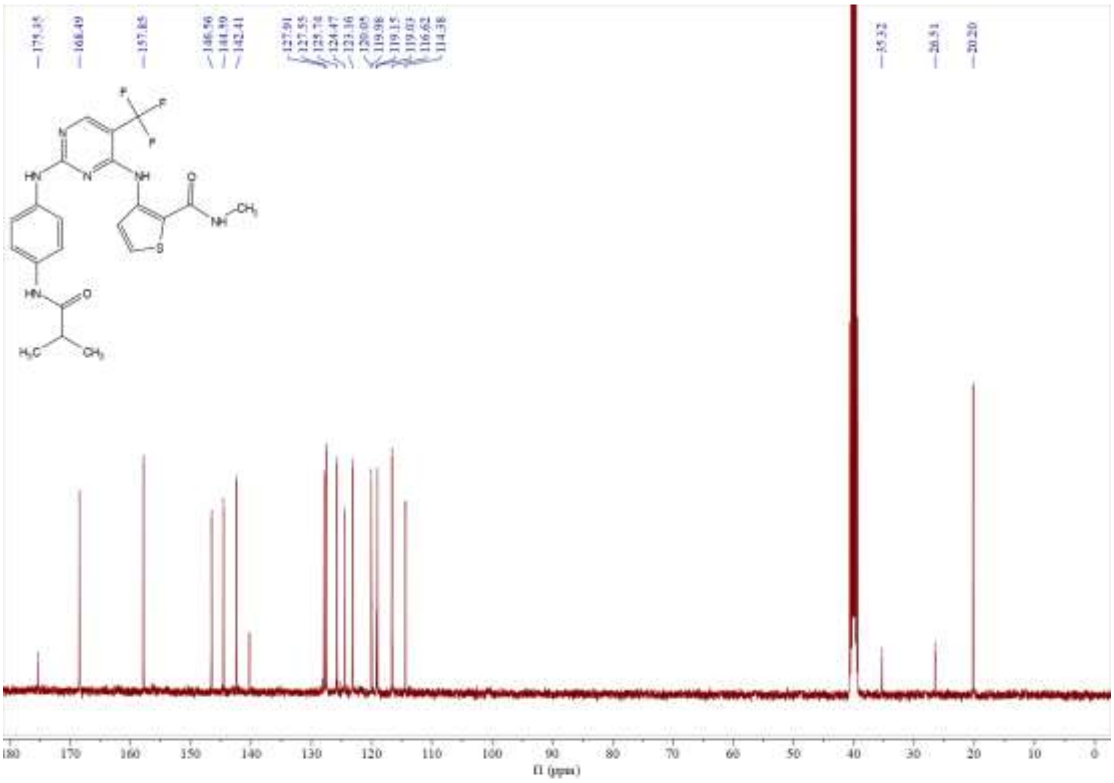

**Figure S52**. $^{13}C$ NMR spectrum of compound **9j**

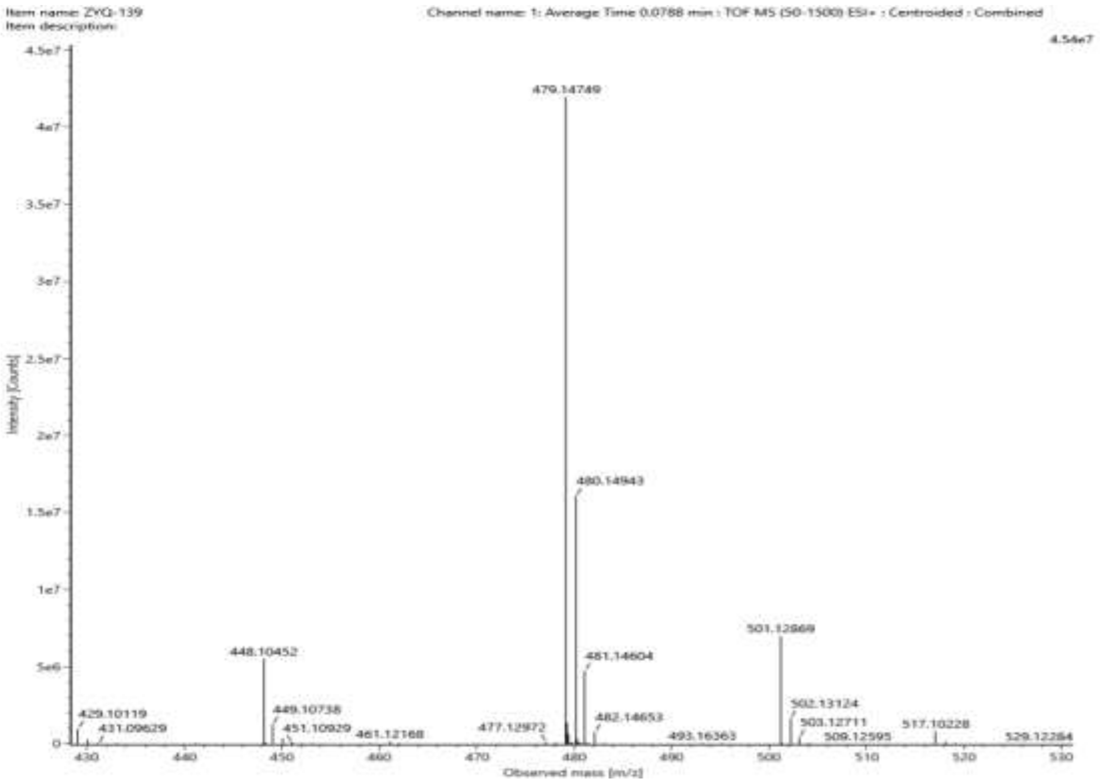


**Figure S53**. HR-MS (ESI) spectrogram of compound **9j**

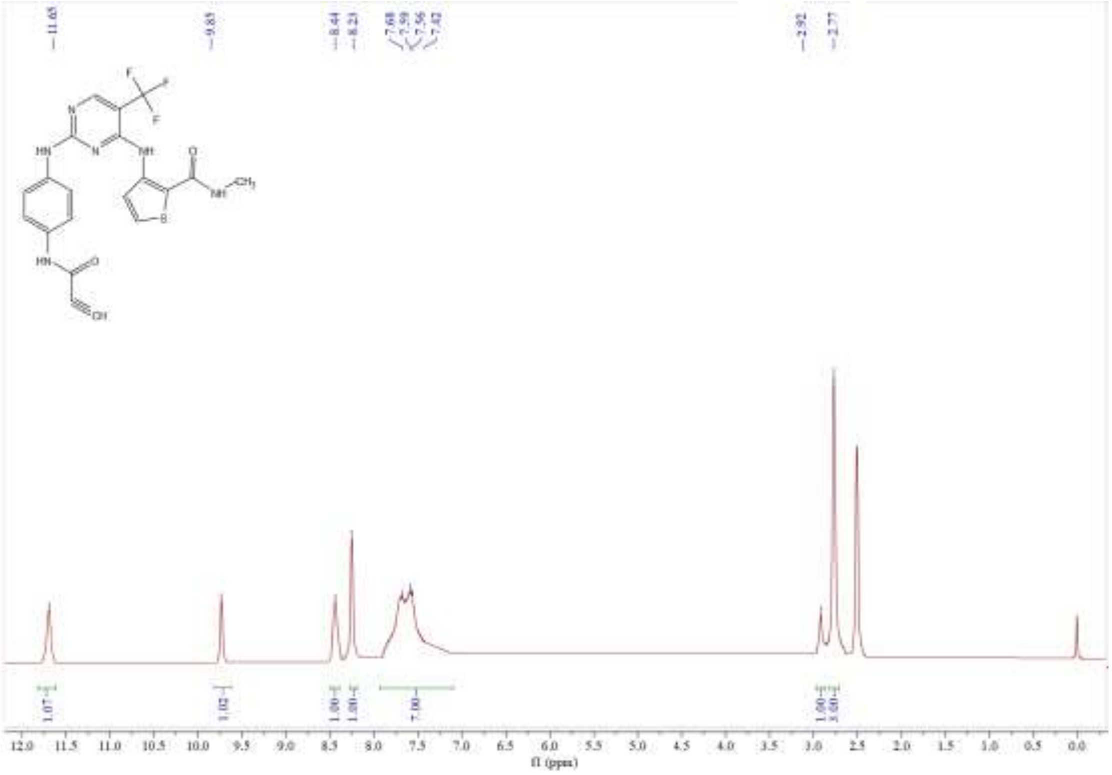


**Figure S54**. $^{1}$H NMR spectrum of compound **9k**

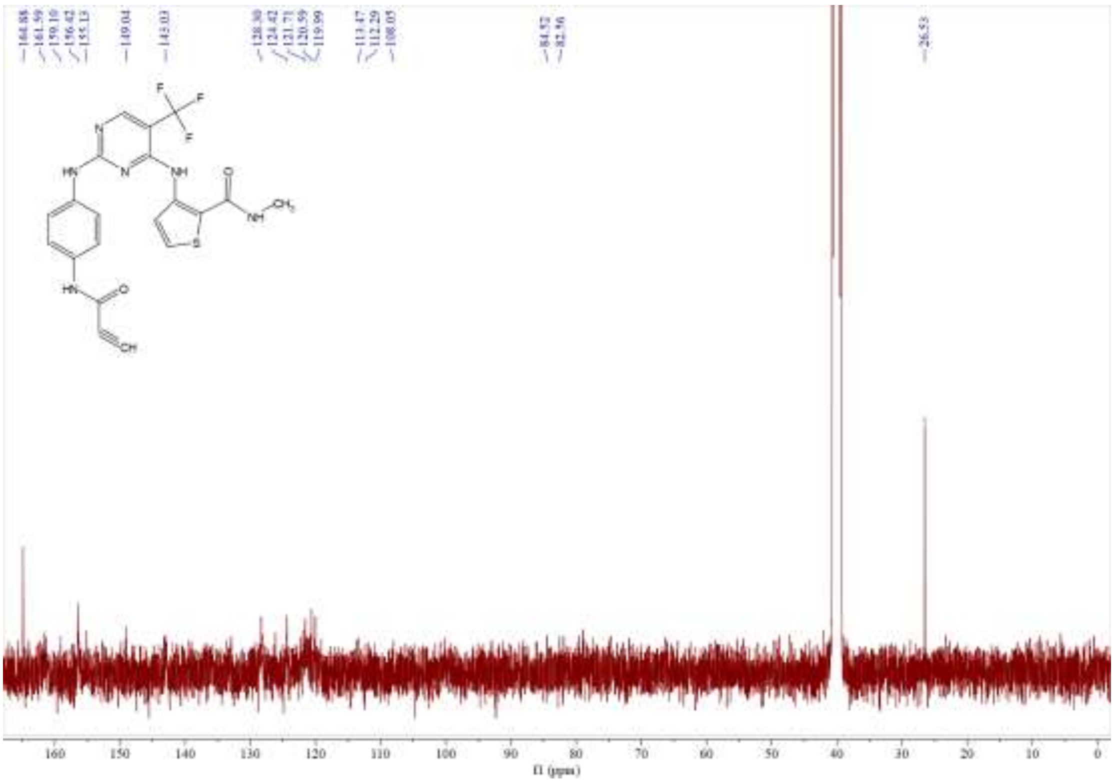

**Figure S55**. $^{13}C$ NMR spectrum of compound **9k**

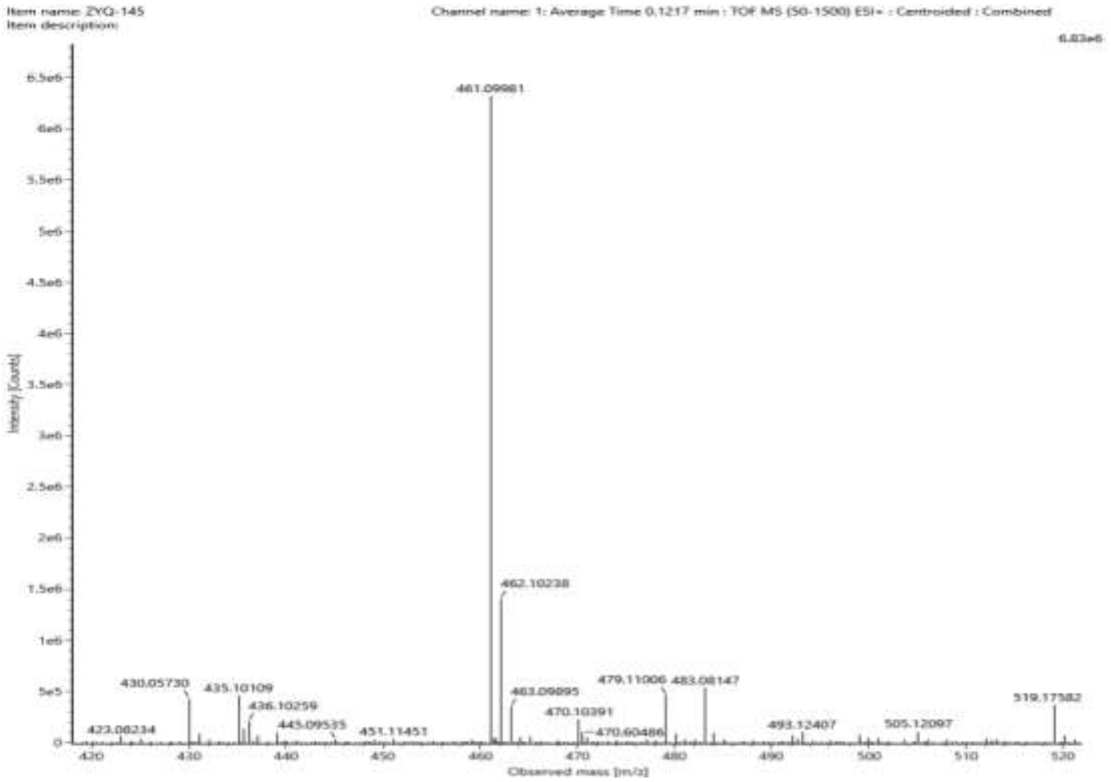

**Figure S56**. HR-MS (ESI) spectrogram of compound **9k**

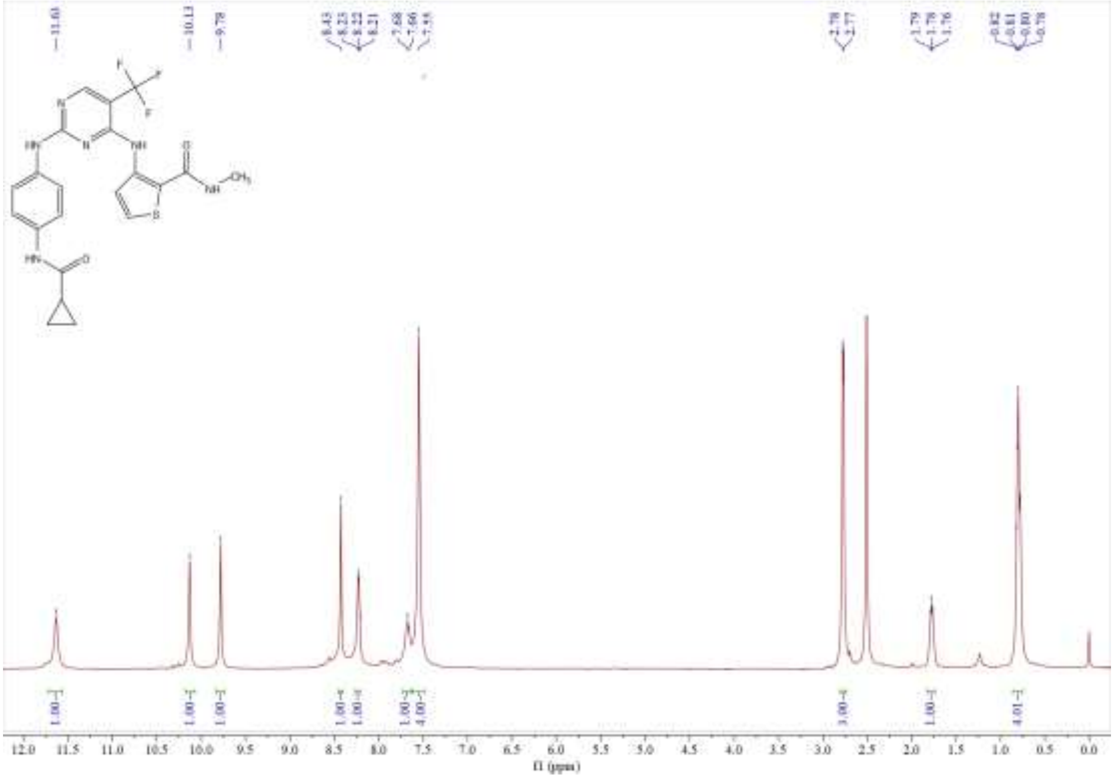

**Figure S57**. $^{1}$H NMR spectrum of compound **9l**

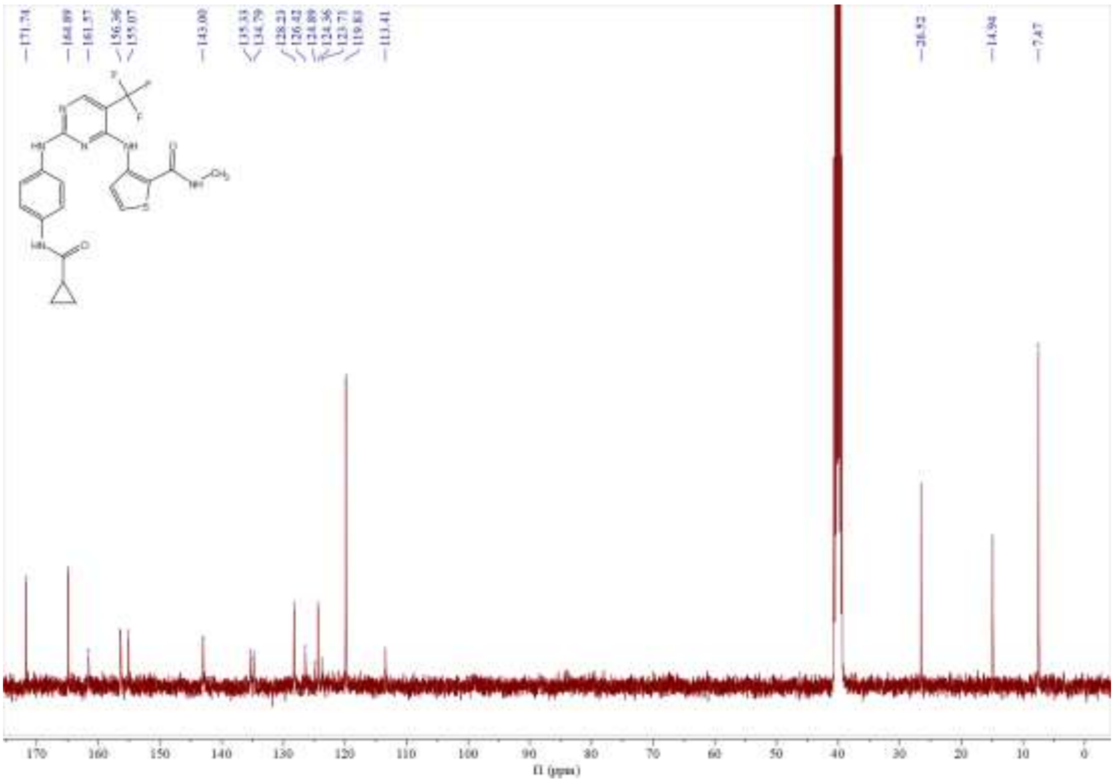

**Figure S58**. $^{13}$C NMR spectrum of compound **9l**

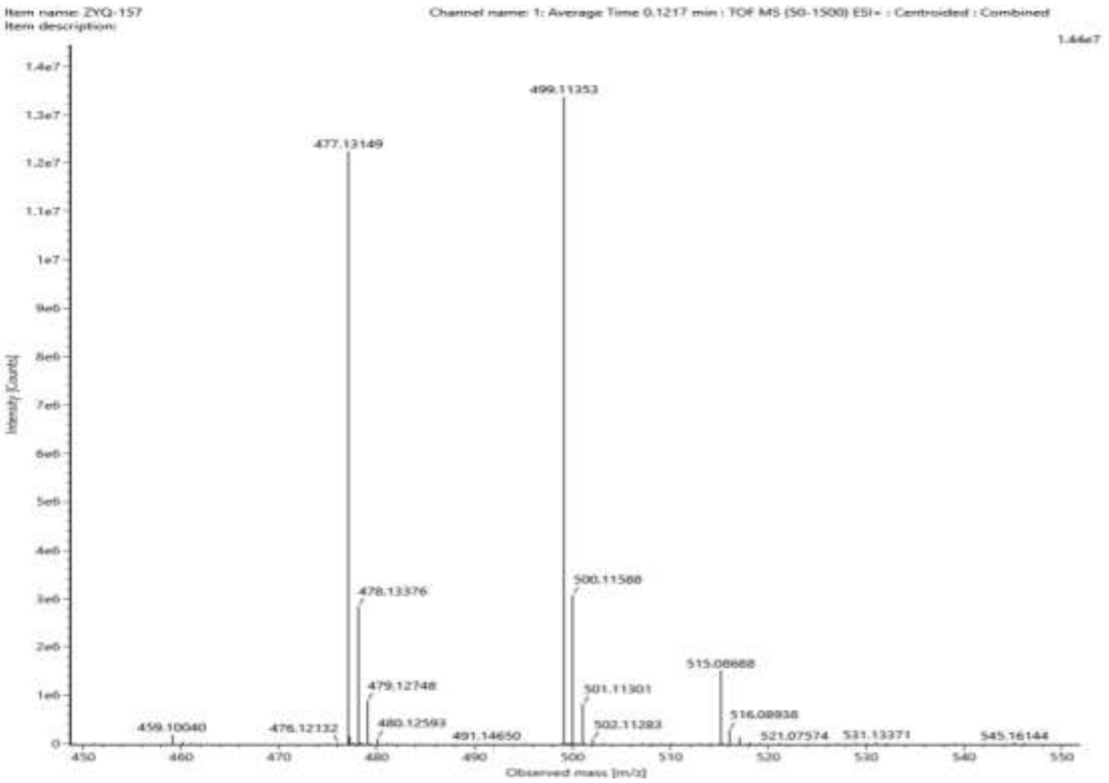


**Figure S59**. HR-MS (ESI) spectrogram of compound **9l**

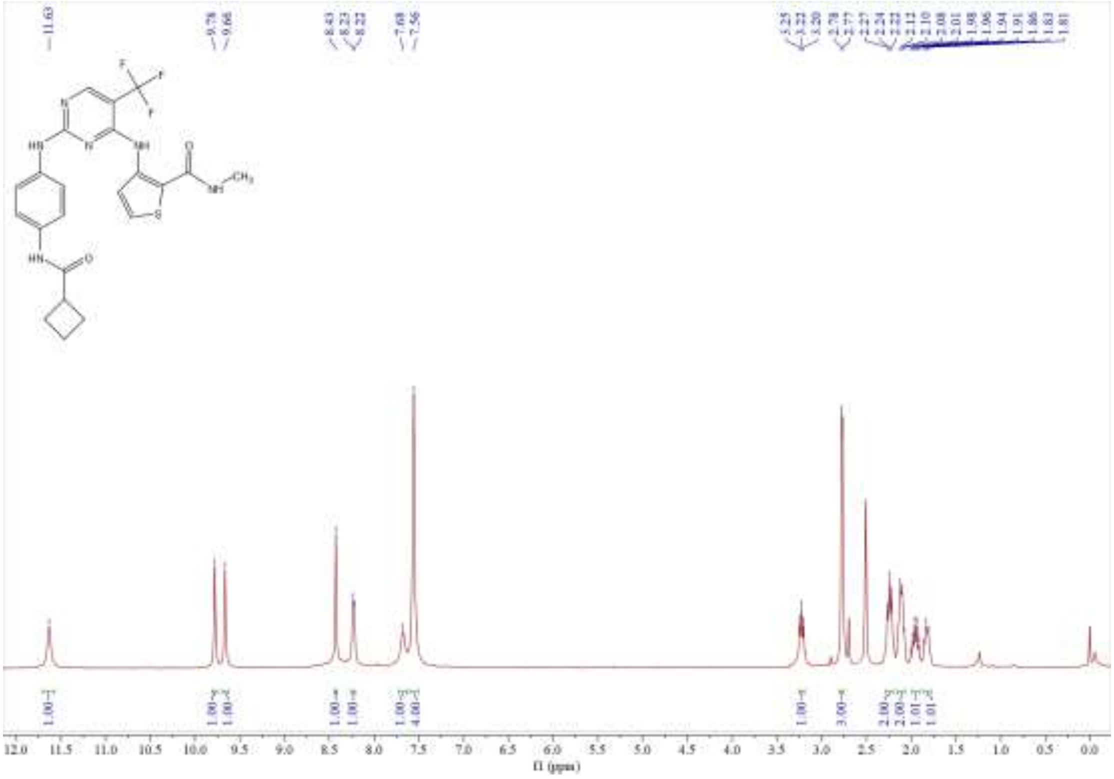


**Figure S60**. $^{1}$H NMR spectrum of compound **9m**

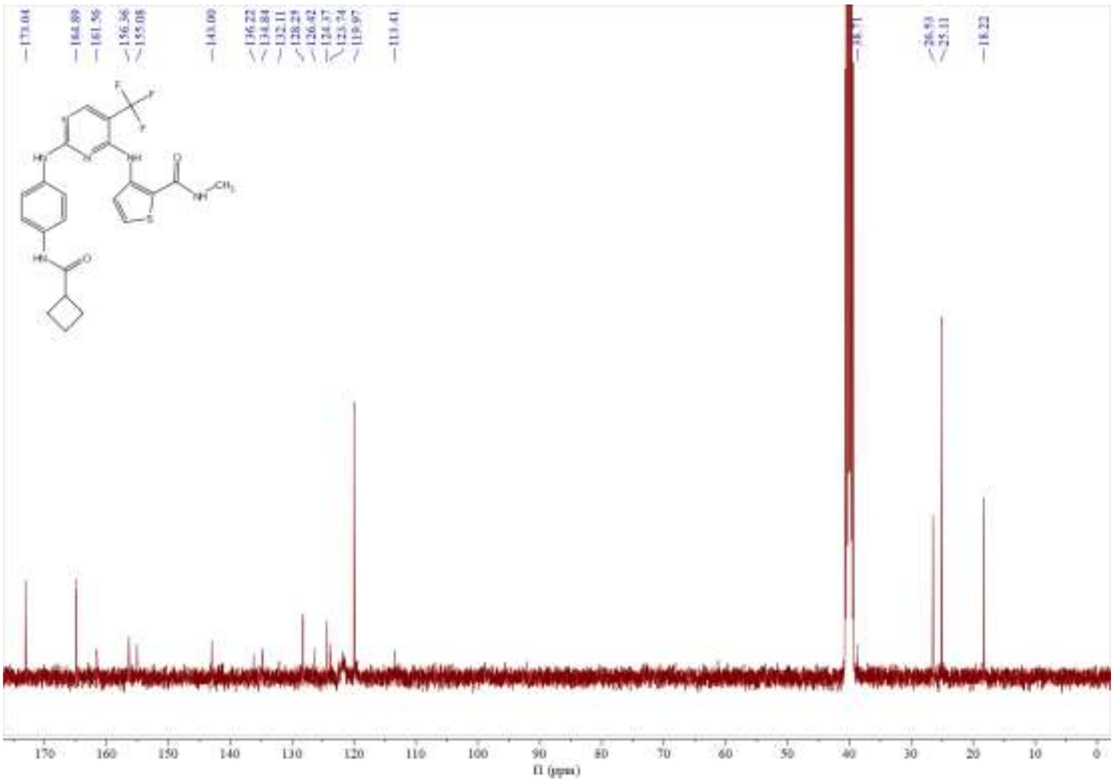


**Figure S61**. $^{13}C$ NMR spectrum of compound **9m**

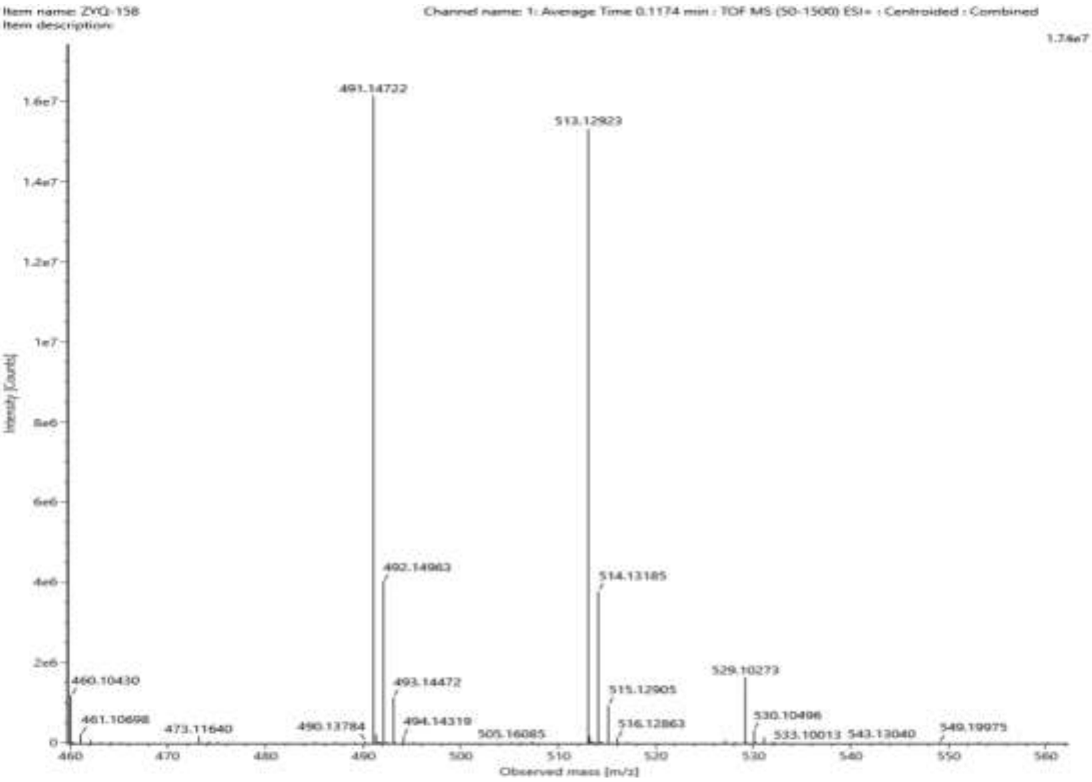


**Figure S62**. HR-MS (ESI) spectrogram of compound **9m**

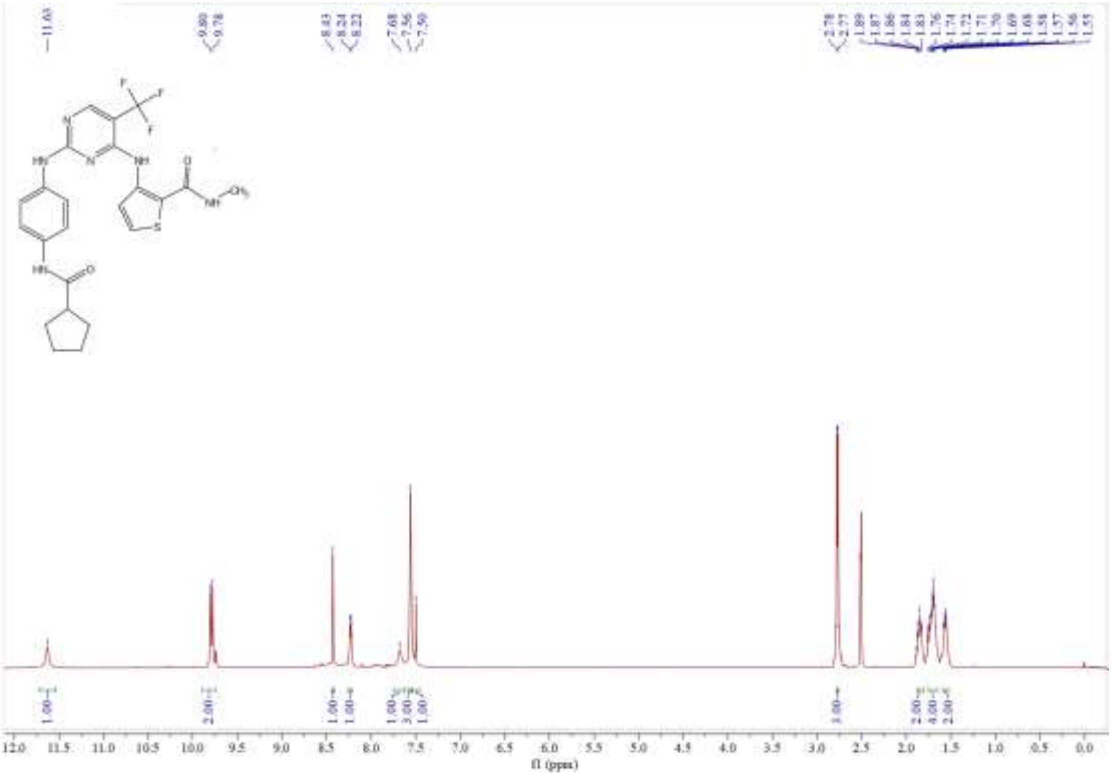

**Figure S63**. $^{1}$H NMR spectrum of compound **9n**

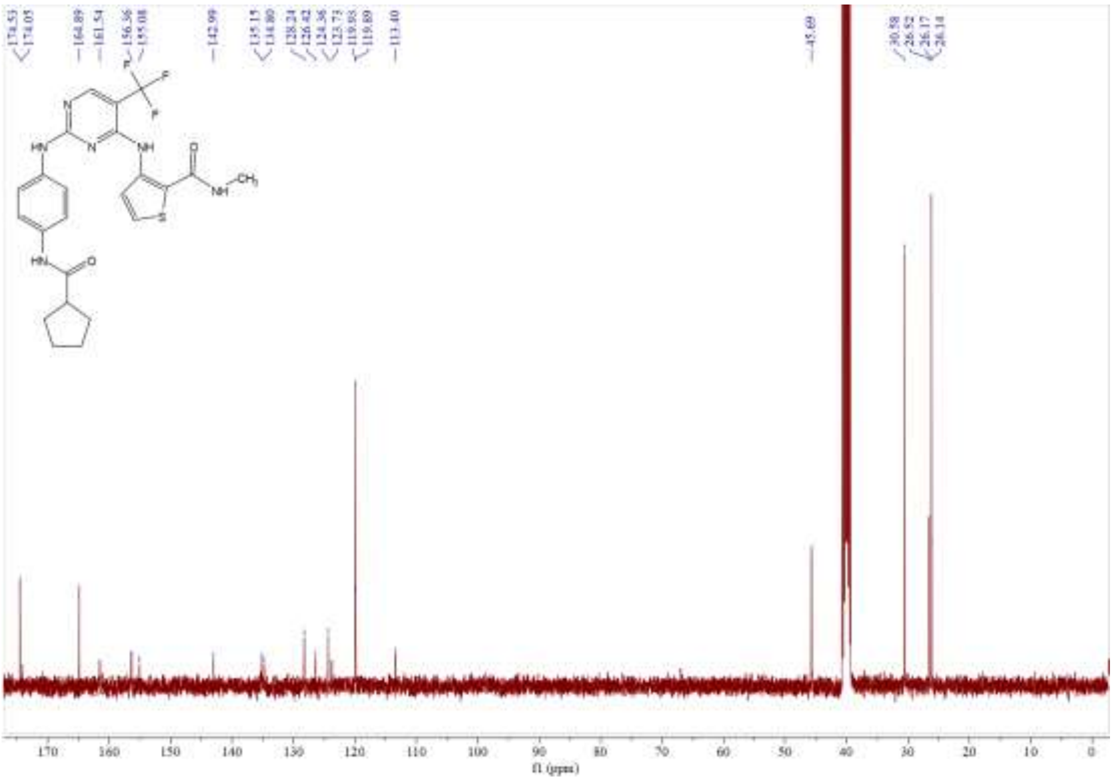

**Figure S64**. $^{13}$C NMR spectrum of compound **9n**

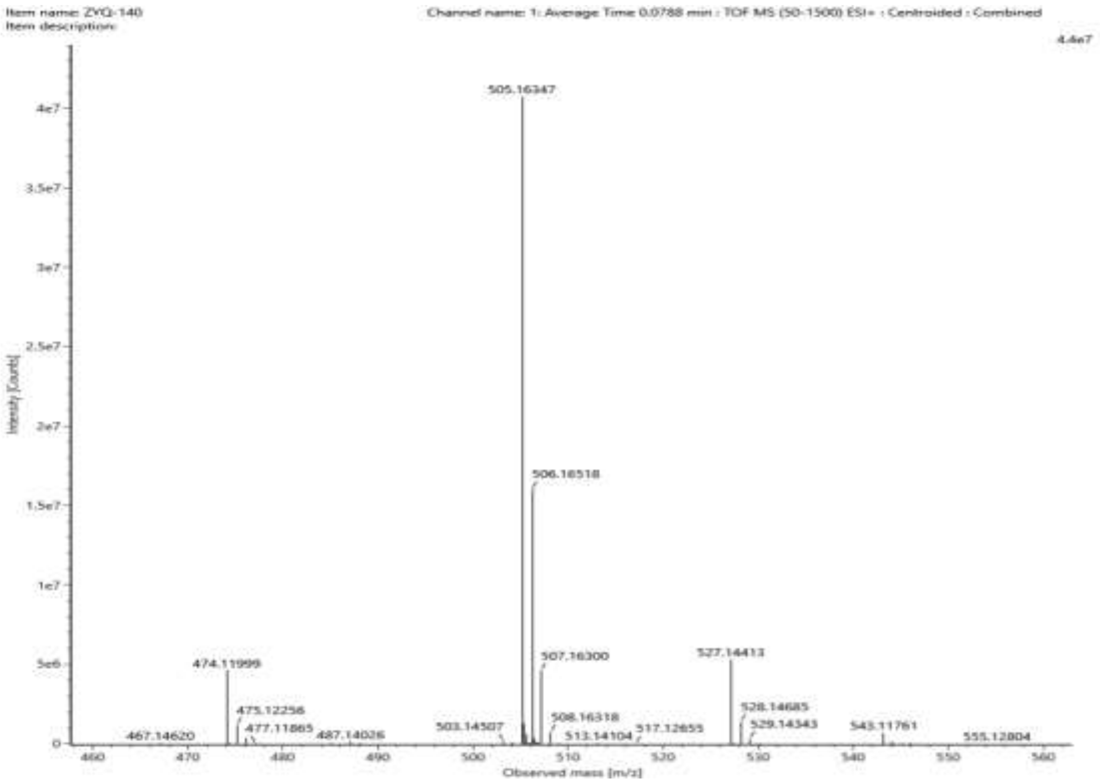

**Figure S65**. HR-MS (ESI) spectrogram of compound **9n**

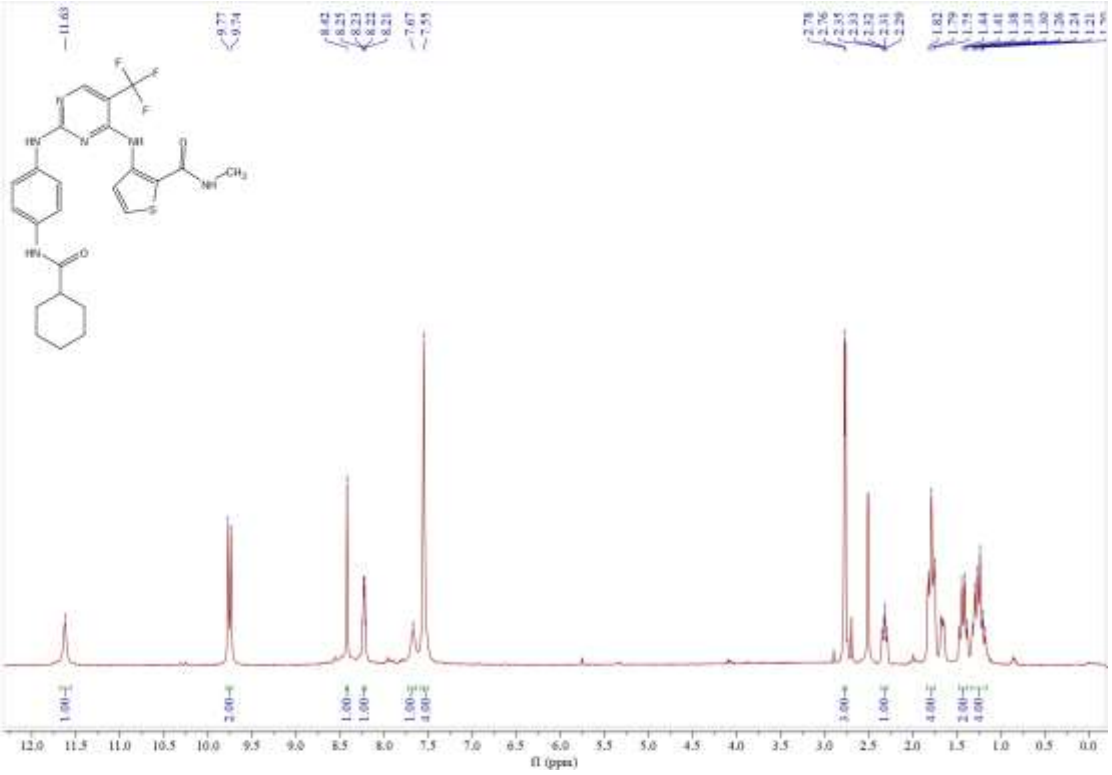

**Figure S66**. $^{1}$H NMR spectrum of compound **9o**

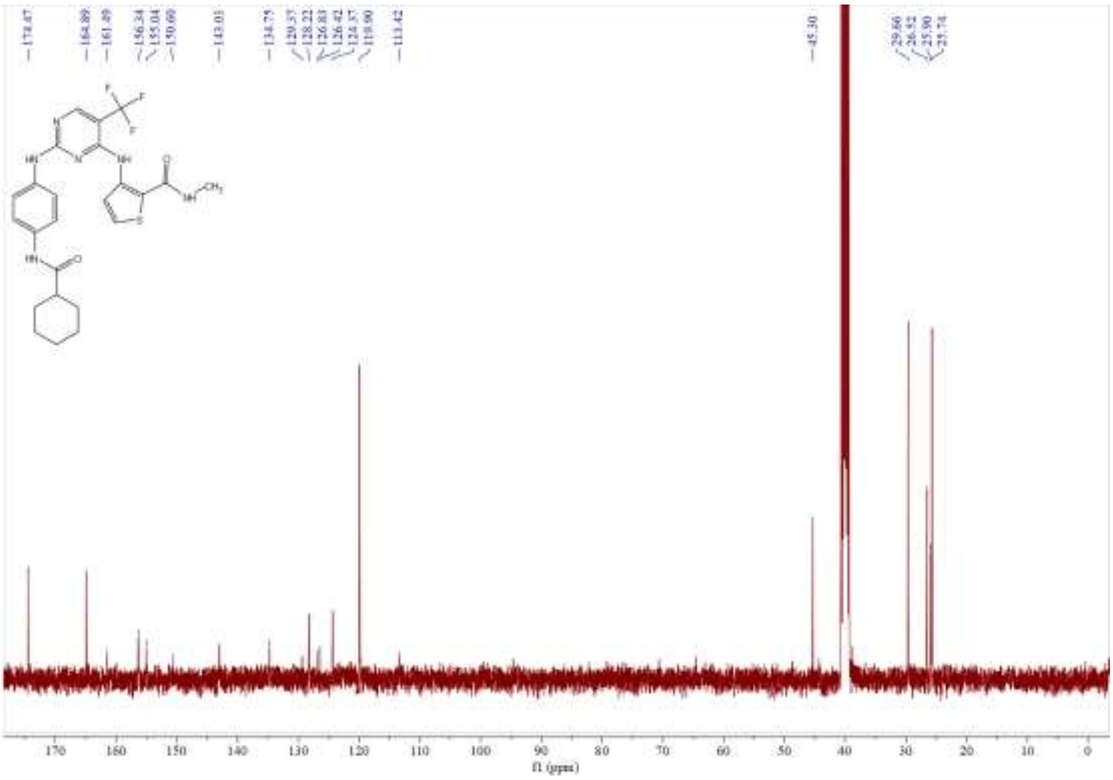


**Figure S67**. $^{13}$C NMR spectrum of compound **9o**

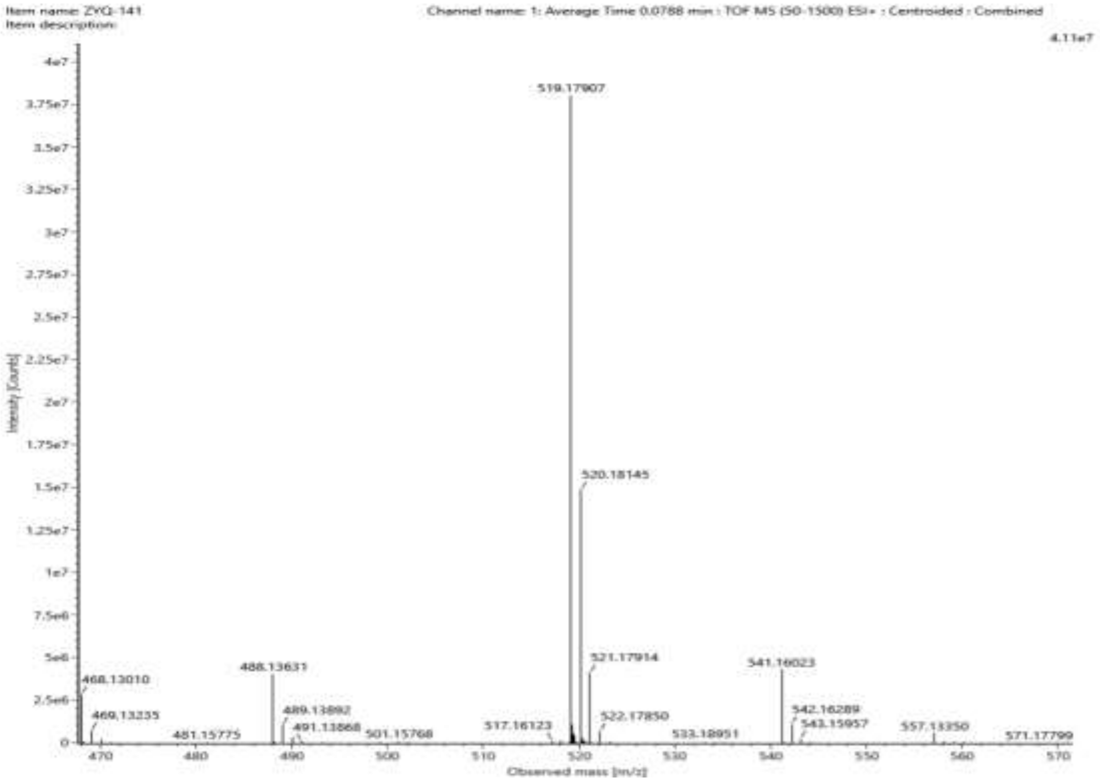


**Figure S68**. HR-MS (ESI) spectrogram of compound **9o**

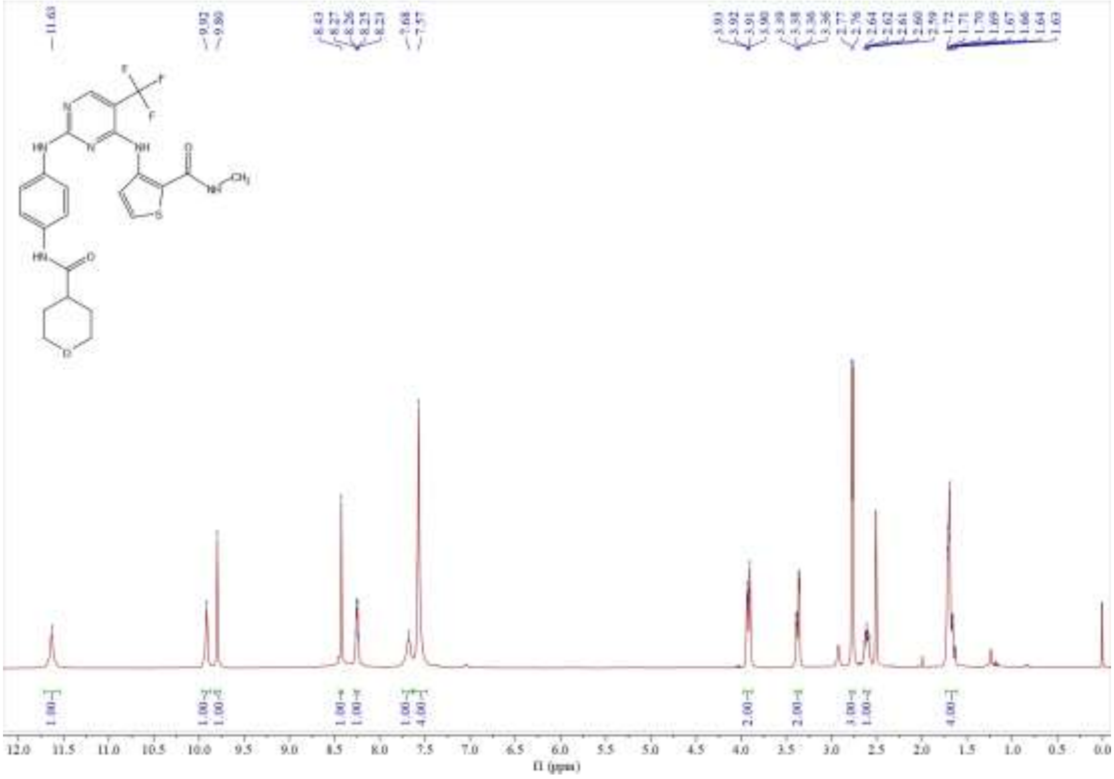

**Figure S69**. $^{1}H$ NMR spectrum of compound **9p**

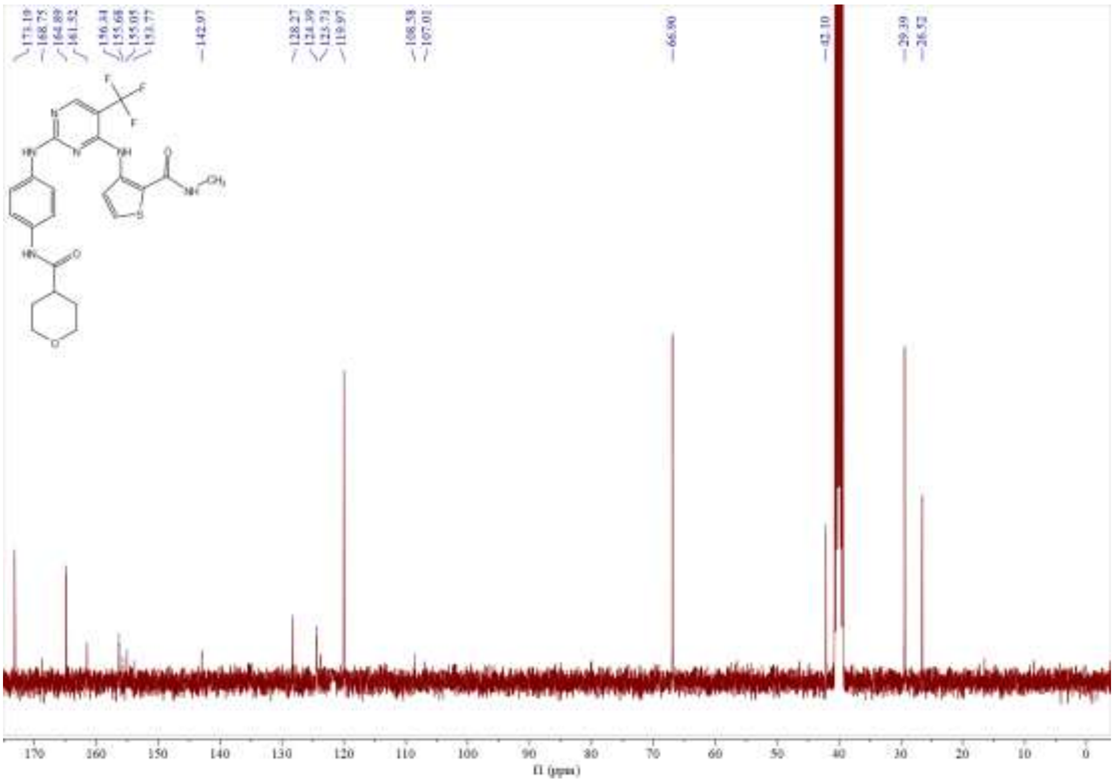

**Figure S70**. $^{13}C$ NMR spectrum of compound **9p**

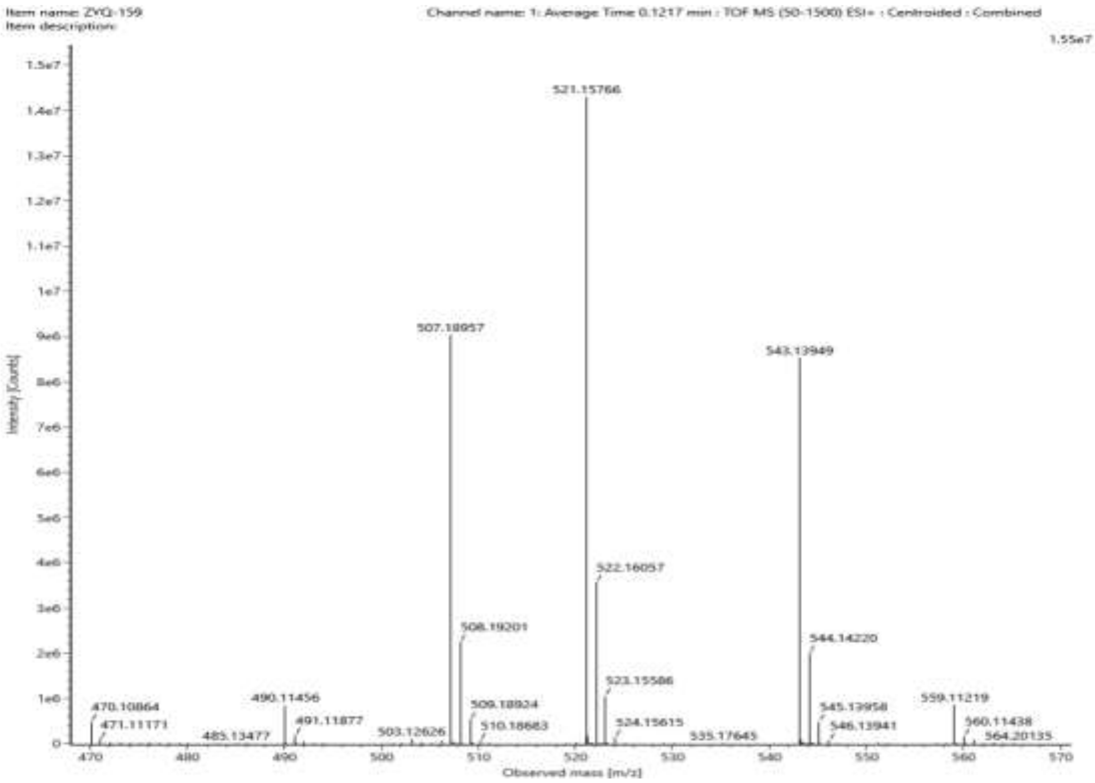

**Figure S71**. HR-MS (ESI) spectrogram of compound **9p**

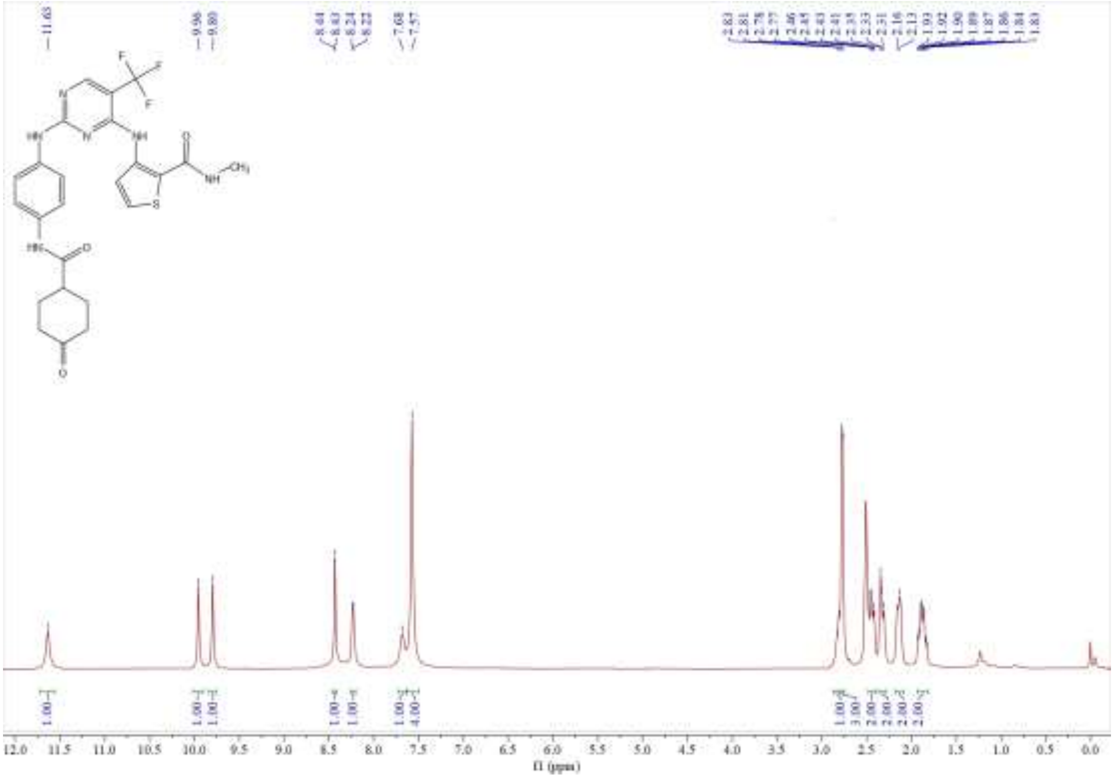

**Figure S72**. $^{1}$H NMR spectrum of compound **9q**

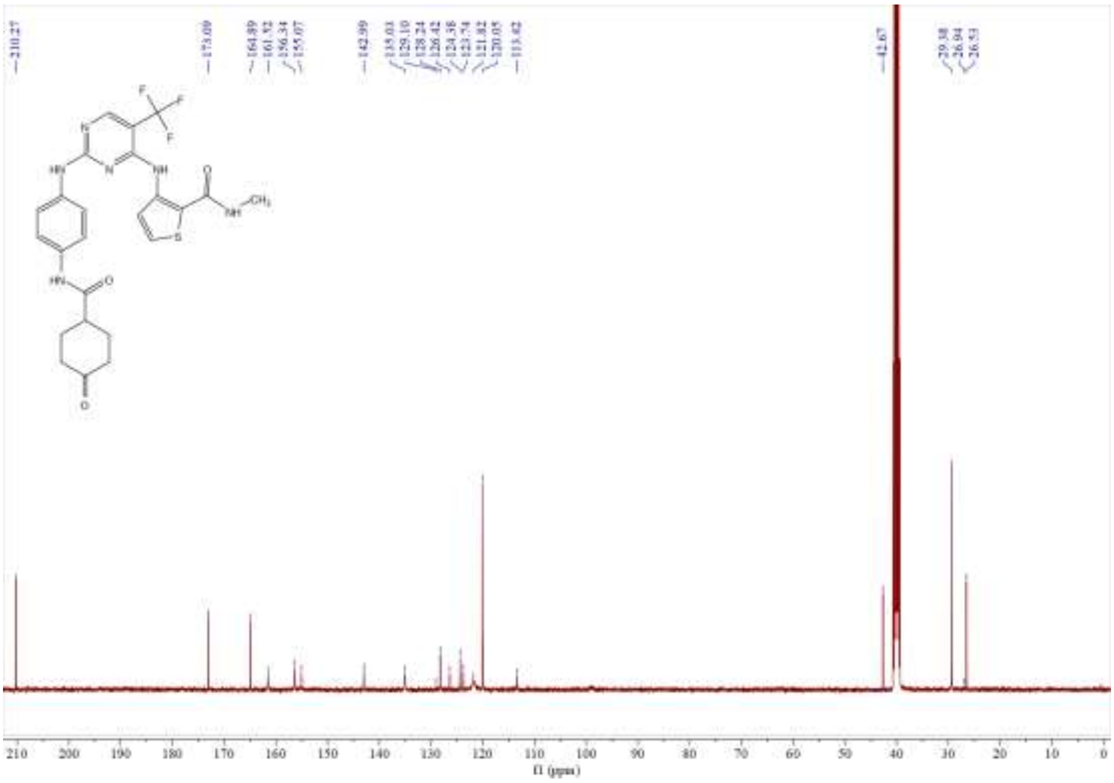

**Figure S73**. $^{13}C$ NMR spectrum of compound **9q**

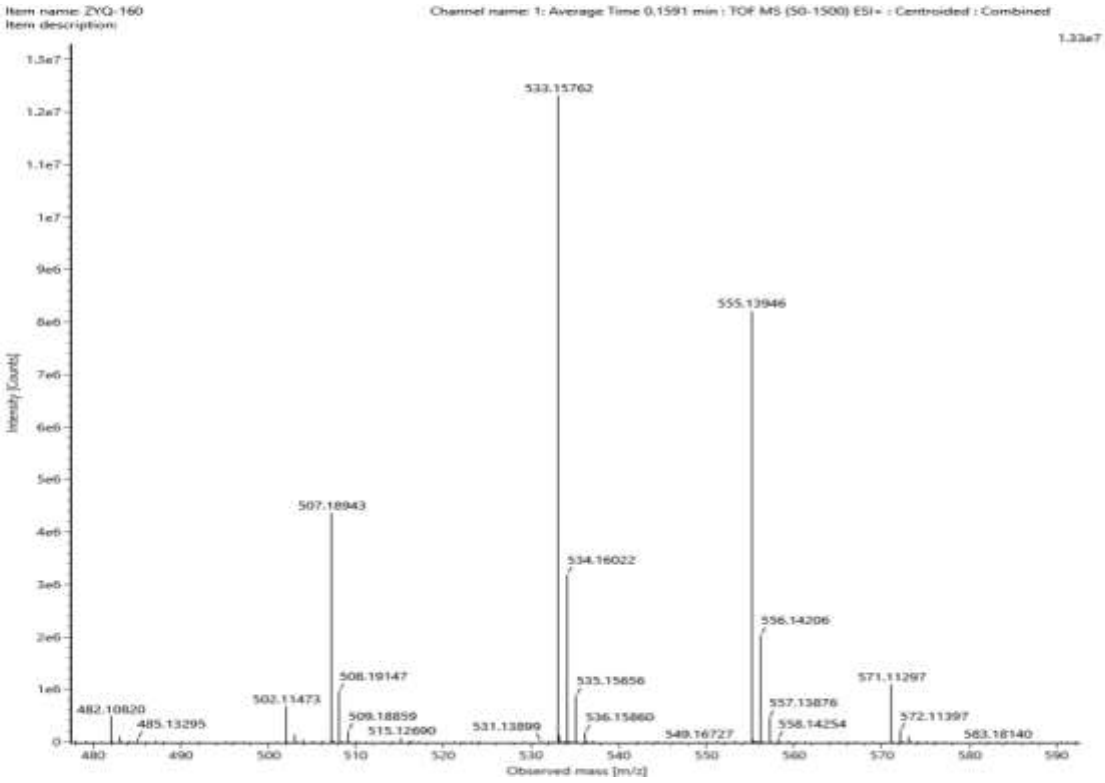


**Figure S74**. HR-MS (ESI) spectrogram of compound **9q**

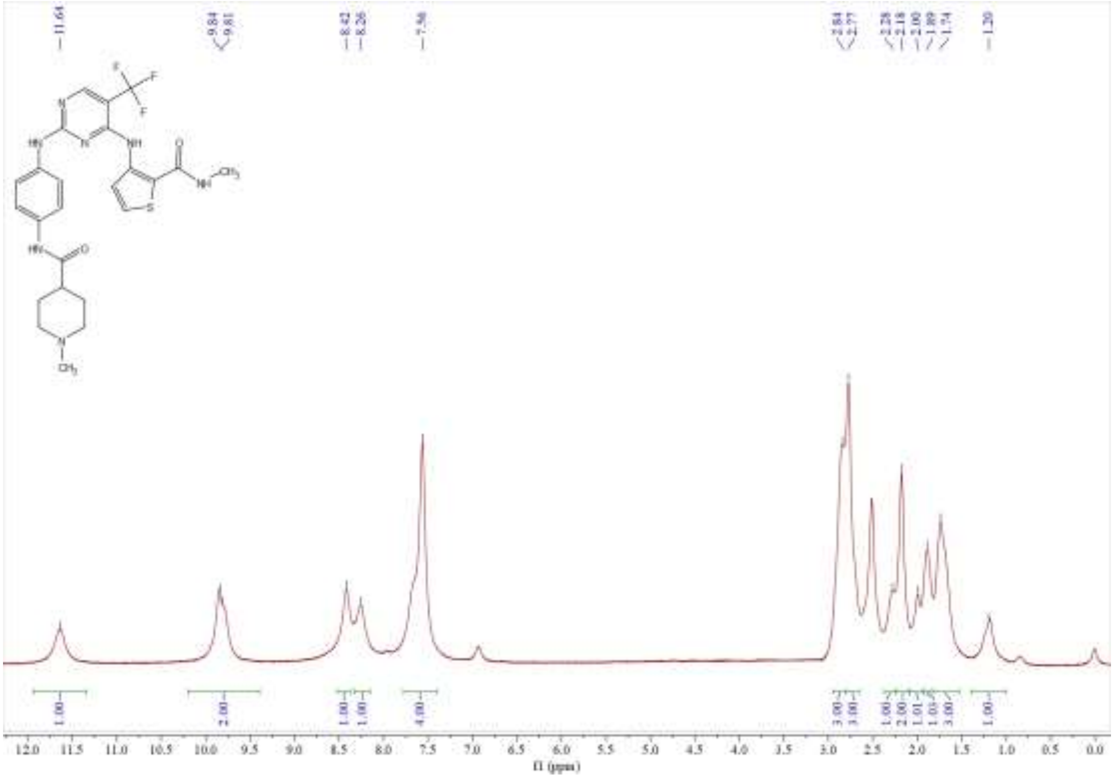

**Figure S75**. $^{1}H$ NMR spectrum of compound **9r**

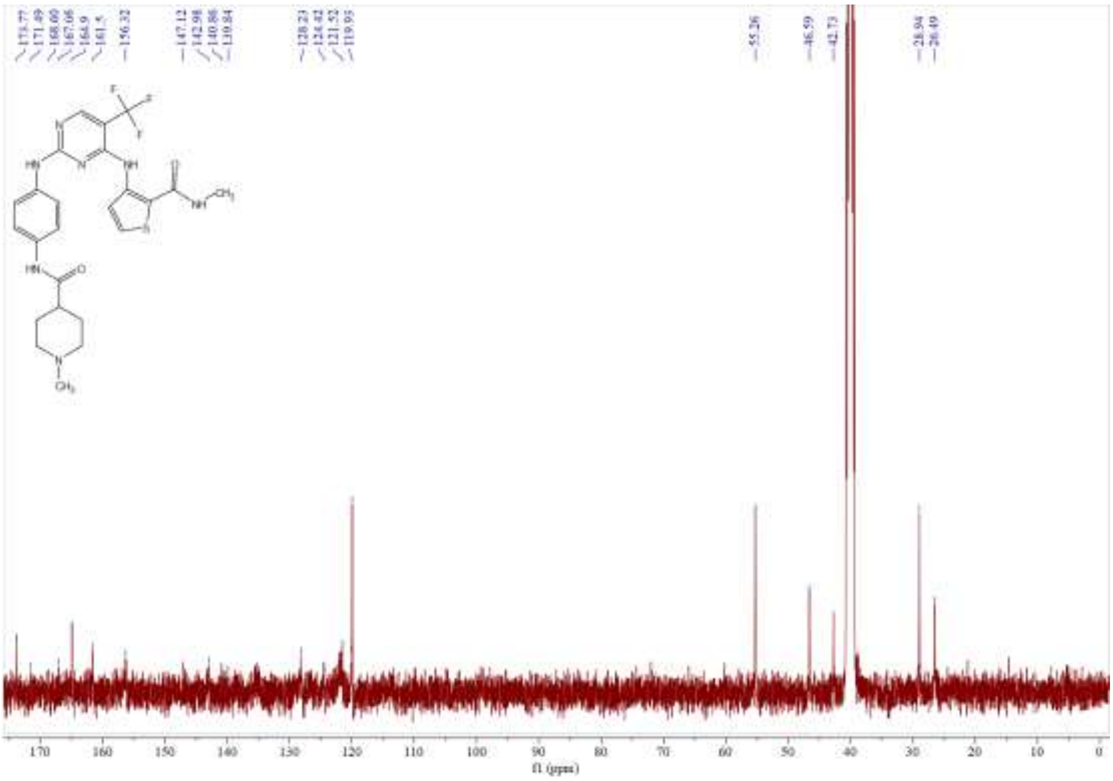

**Figure S76**. $^{13}C$ NMR spectrum of compound **9r**

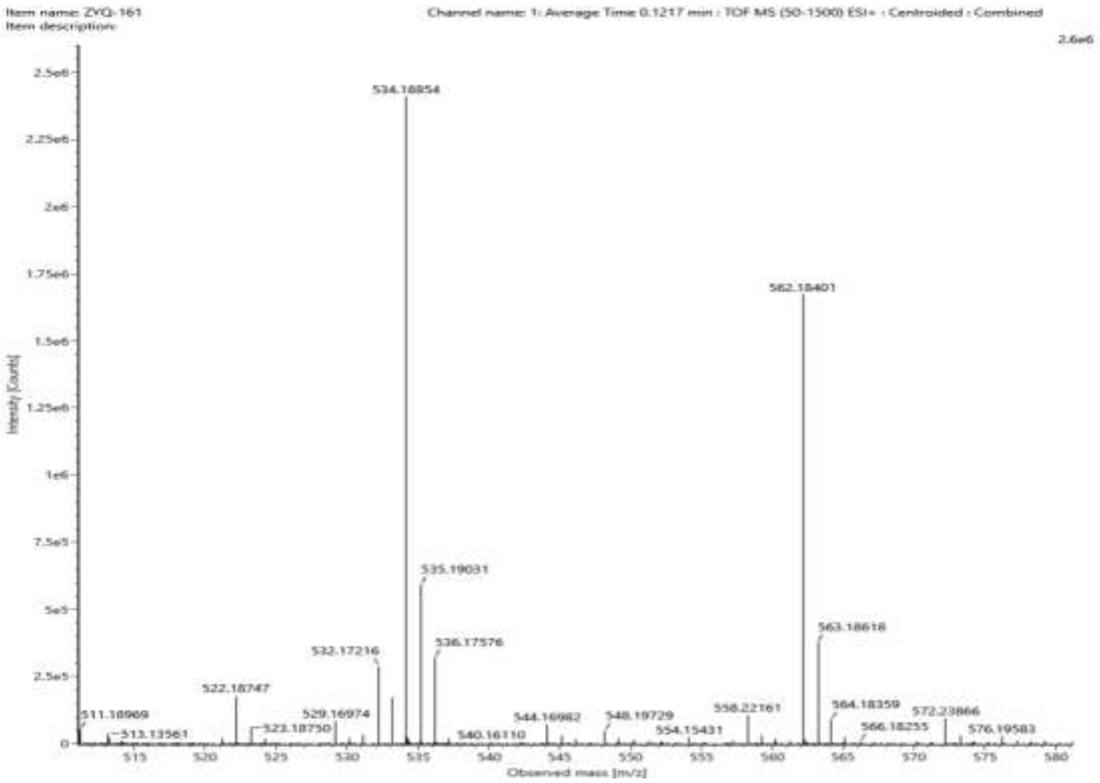


**Figure S77**. HR-MS (ESI) spectrogram of compound **9r**

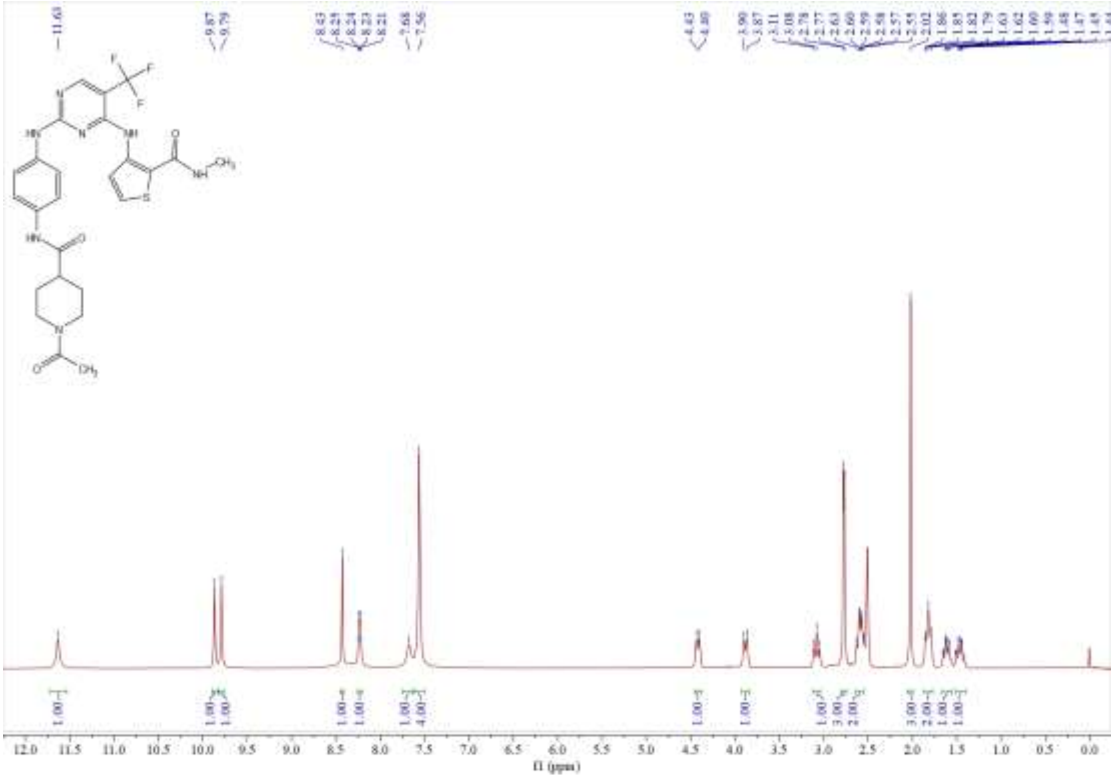


**Figure S78**. $^{1}$H NMR spectrum of compound **9s**

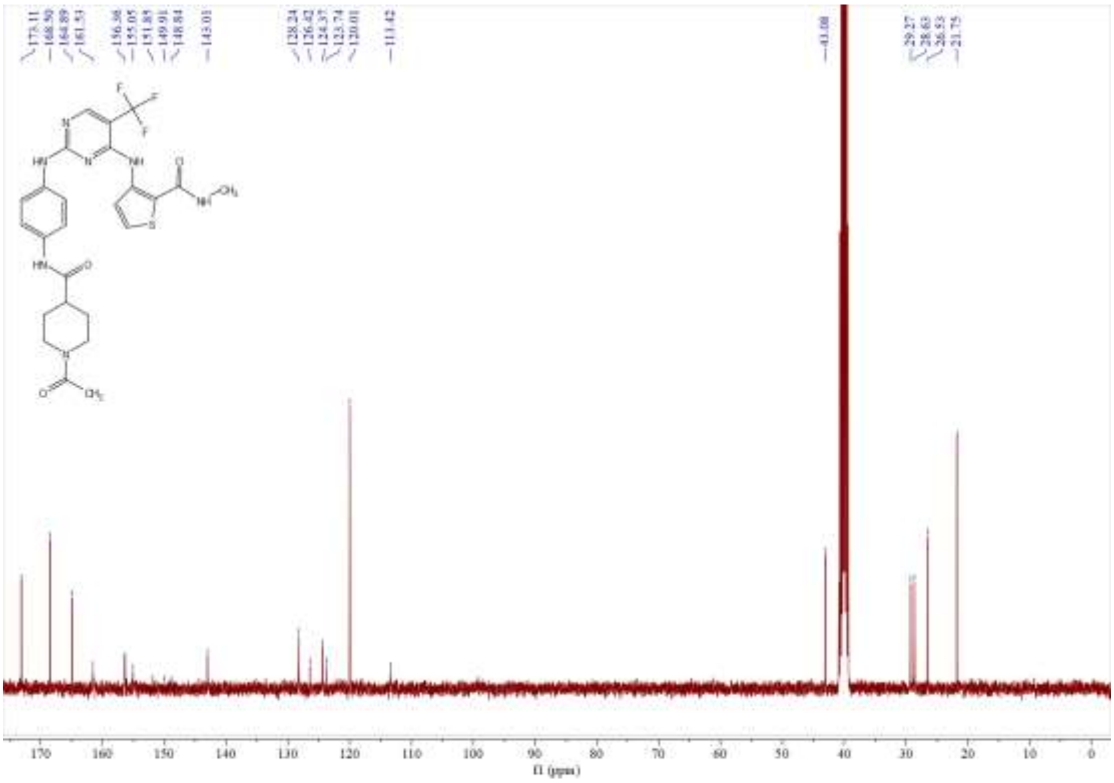

**Figure S79**. $^{13}$C NMR spectrum of compound **9s**

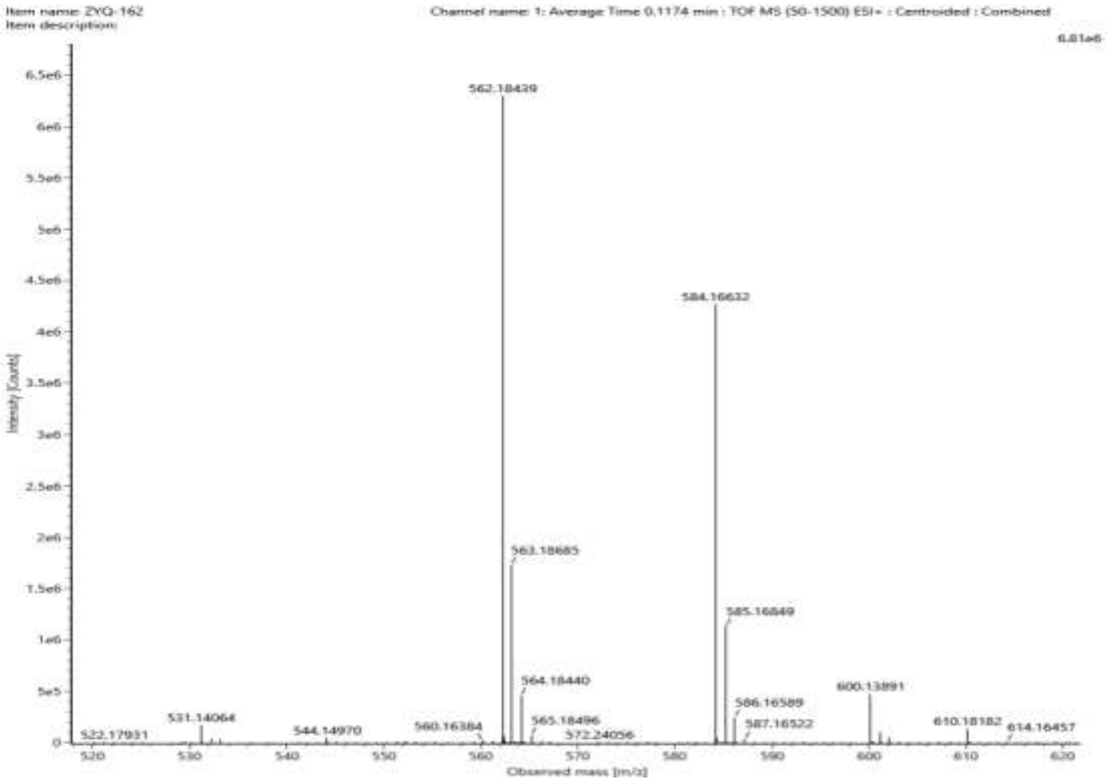

**Figure S80**. HR-MS (ESI) spectrogram of compound **9s**

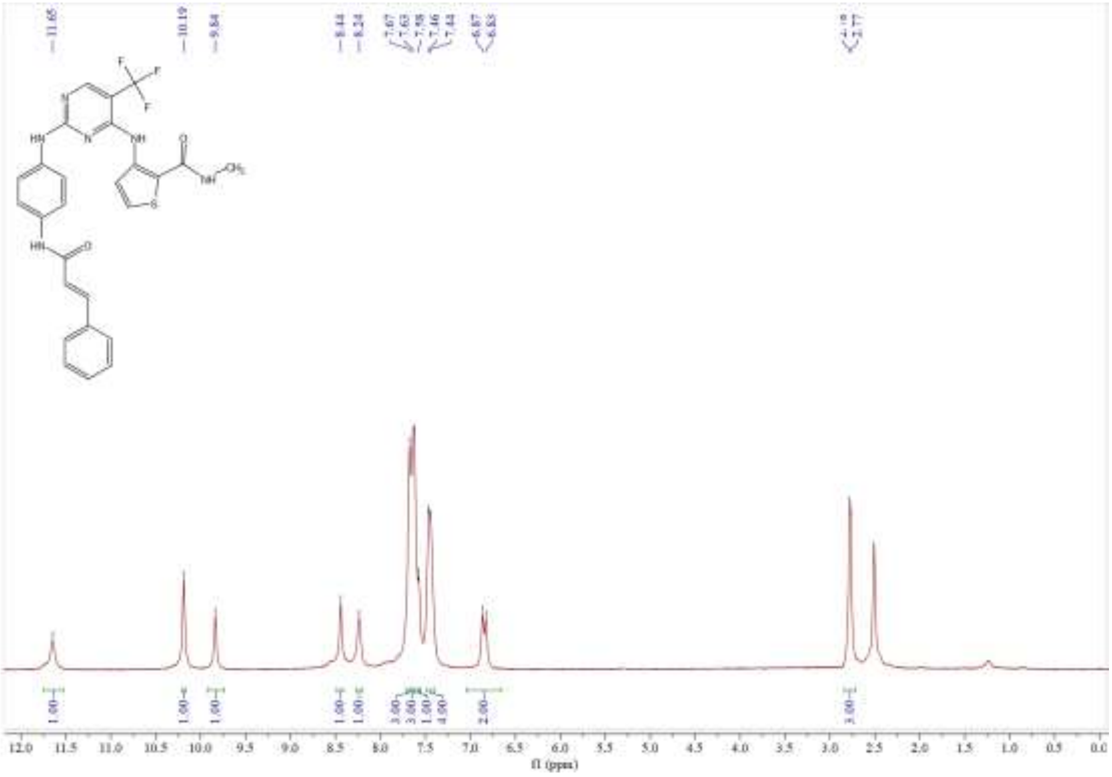

**Figure S81**. 1H NMR spectrum of compound **9t**

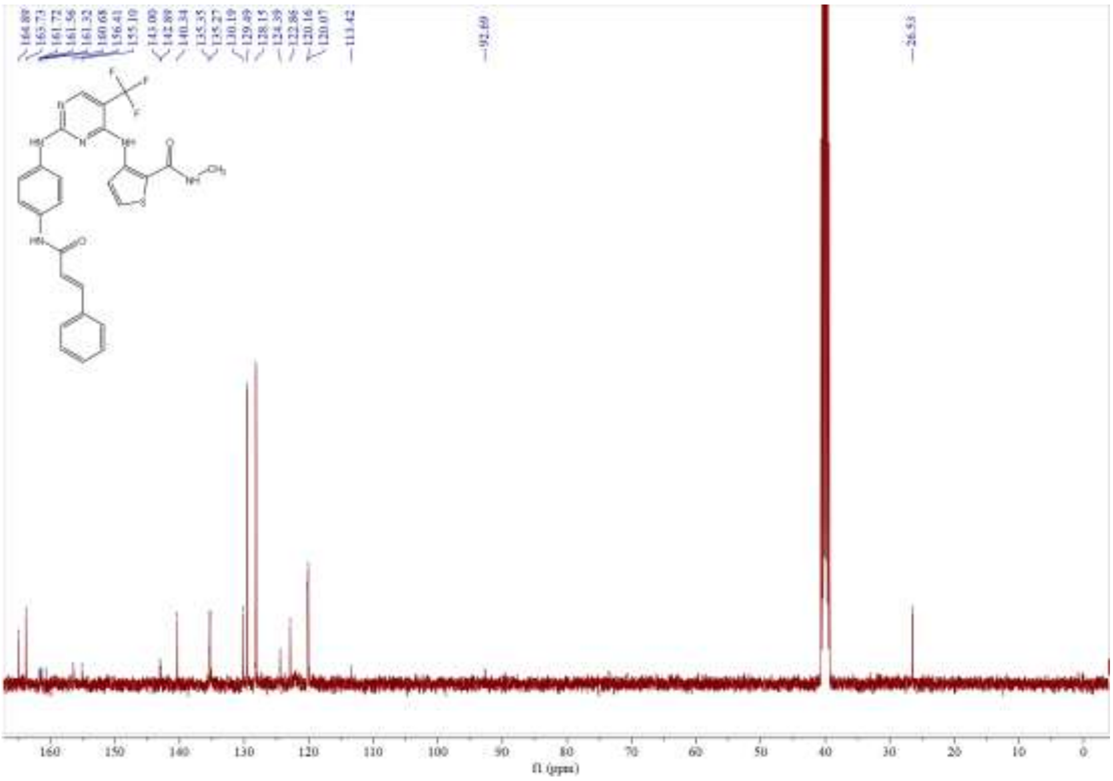

**Figure S82**. $^{13}C$ NMR spectrum of compound **9t**

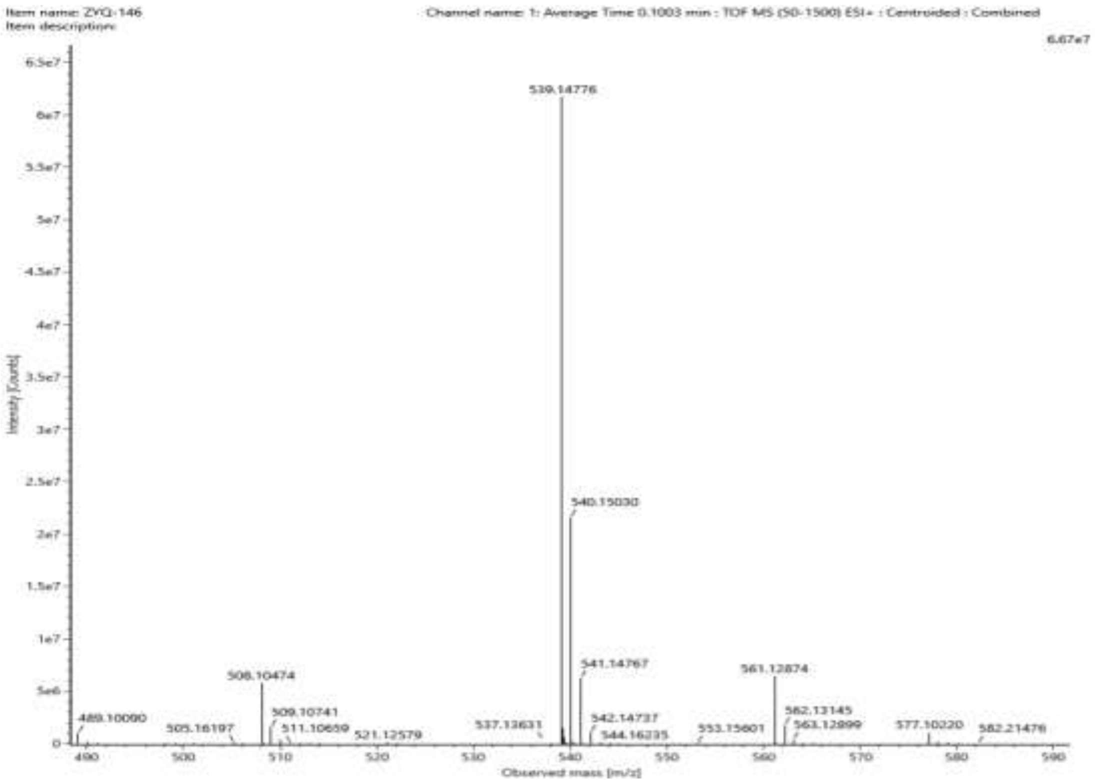


**Figure S83**. HR-MS (ESI) spectrogram of compound **9t**

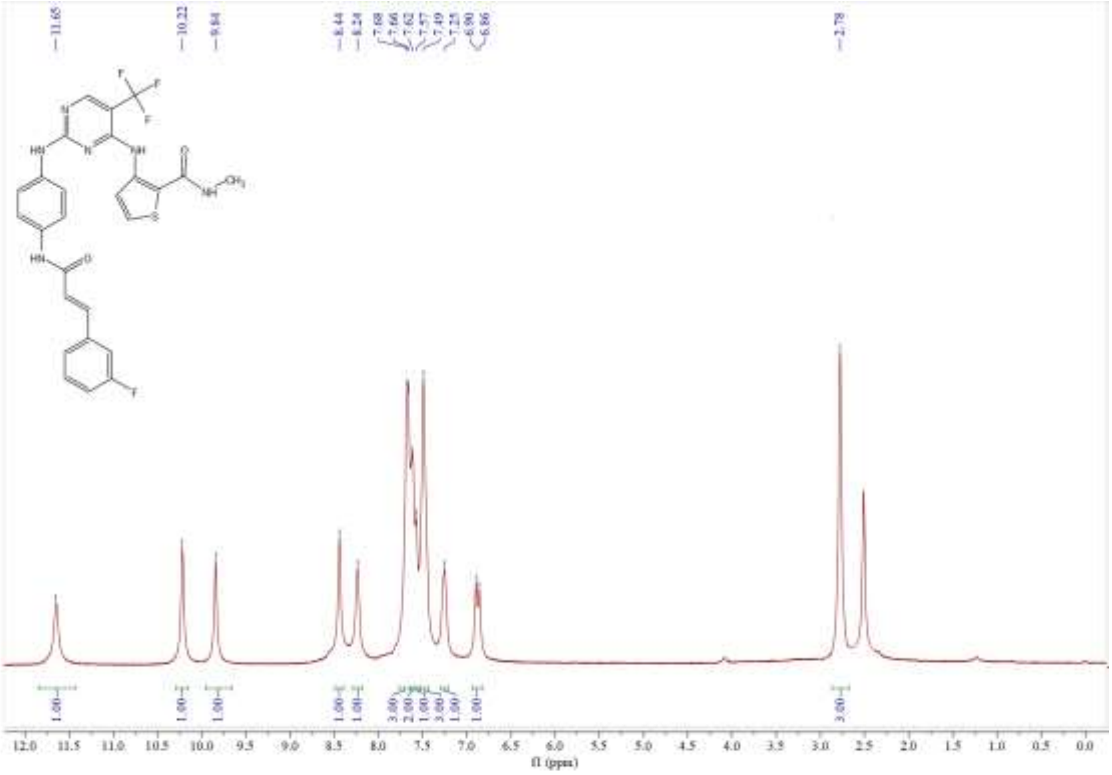


**Figure S84**. $^{1}$H NMR spectrum of compound **9u**

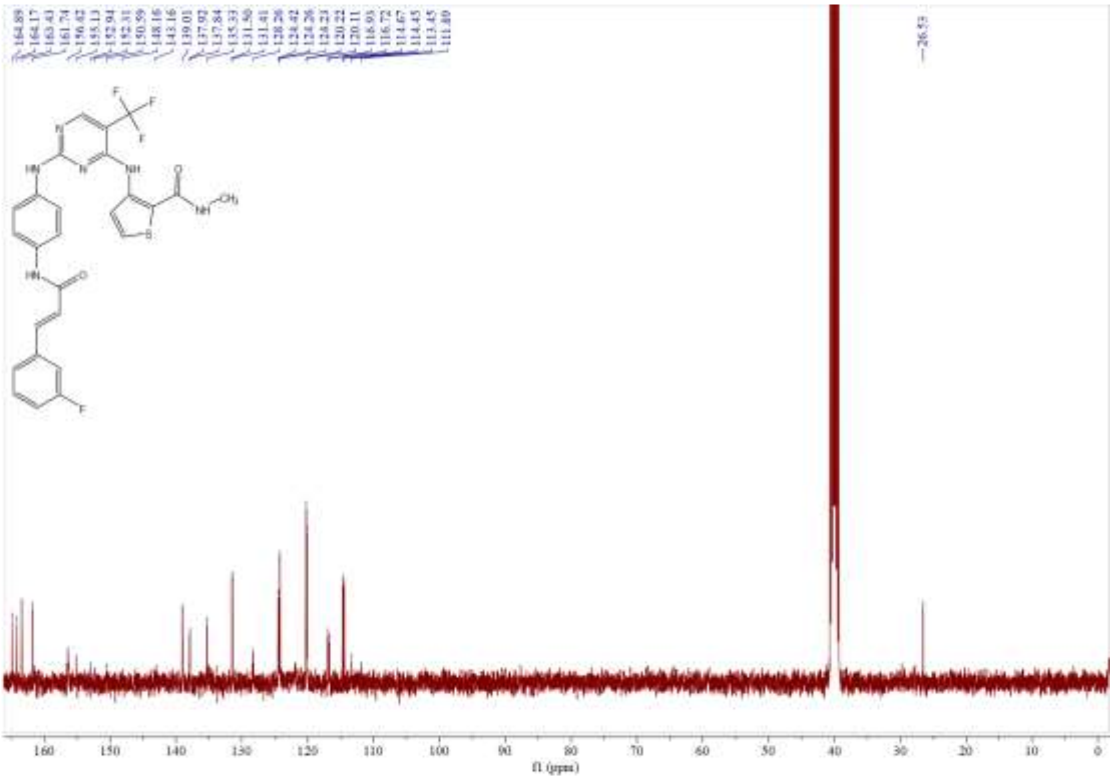

**Figure S85**. $^{13}C$ NMR spectrum of compound **9u**

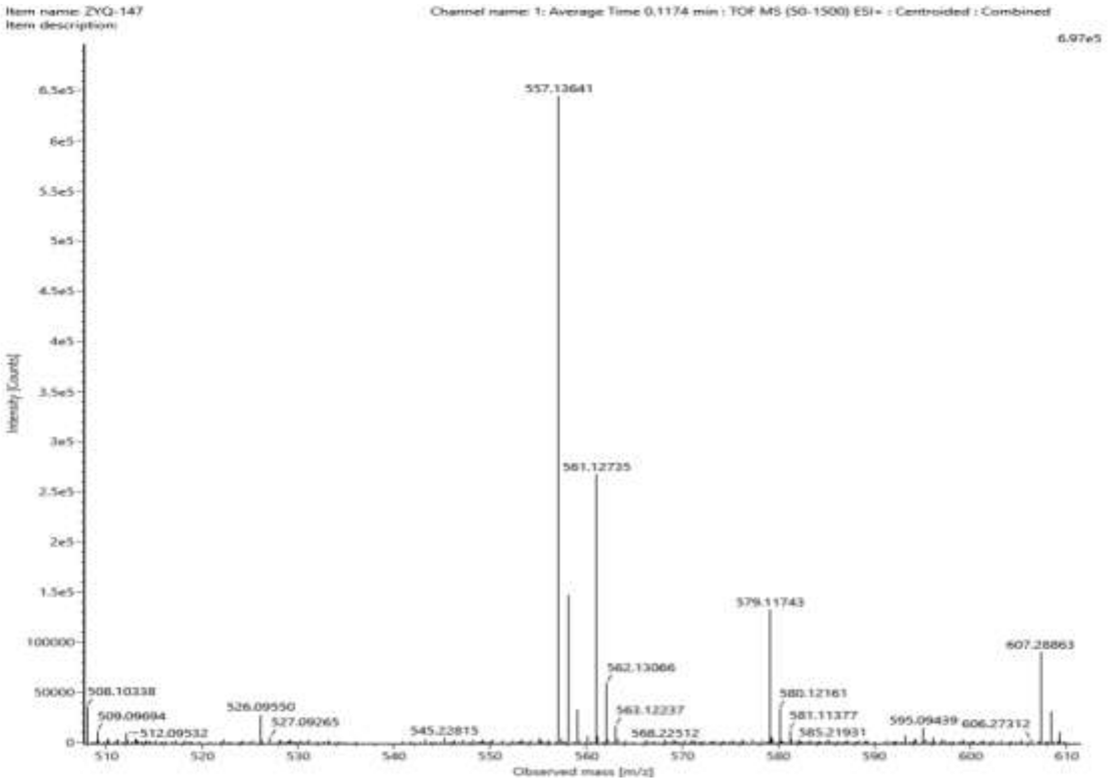

**Figure S86**. HR-MS (ESI) spectrogram of compound **9u**

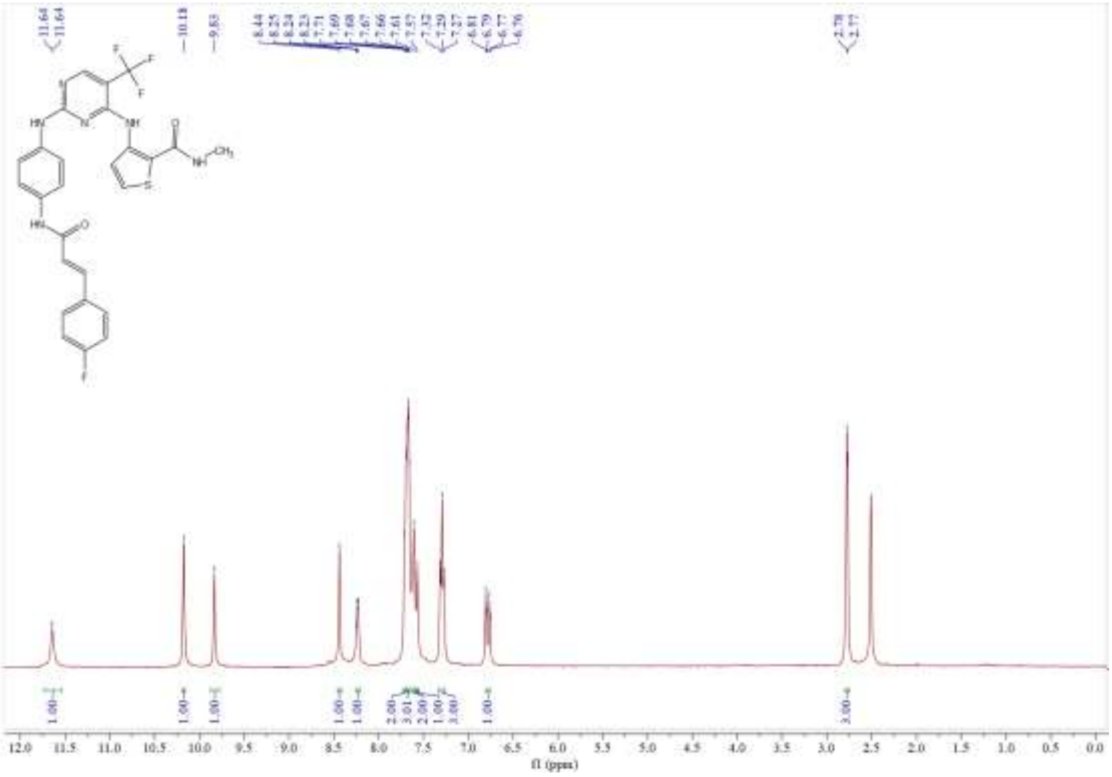

**Figure S87**. $^{1}H$ NMR spectrum of compound **9v**

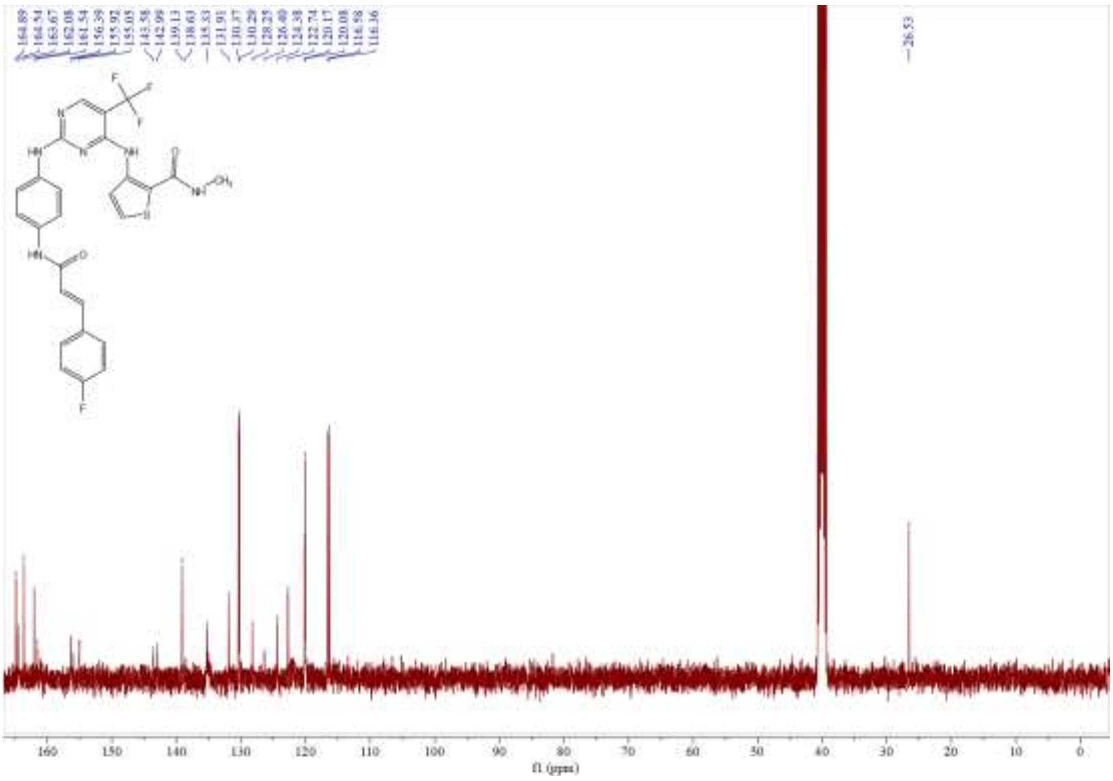

**Figure S88**. $^{13}C$ NMR spectrum of compound **9v**

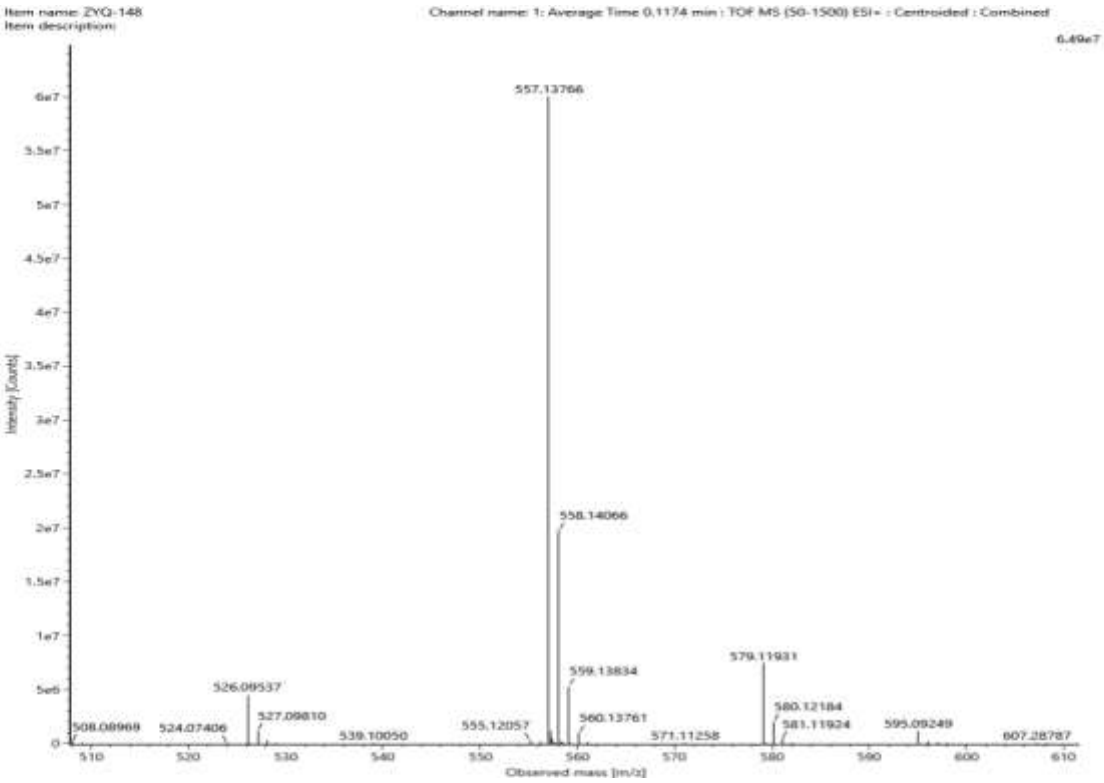


**Figure S89**. HR-MS (ESI) spectrogram of compound **9v**

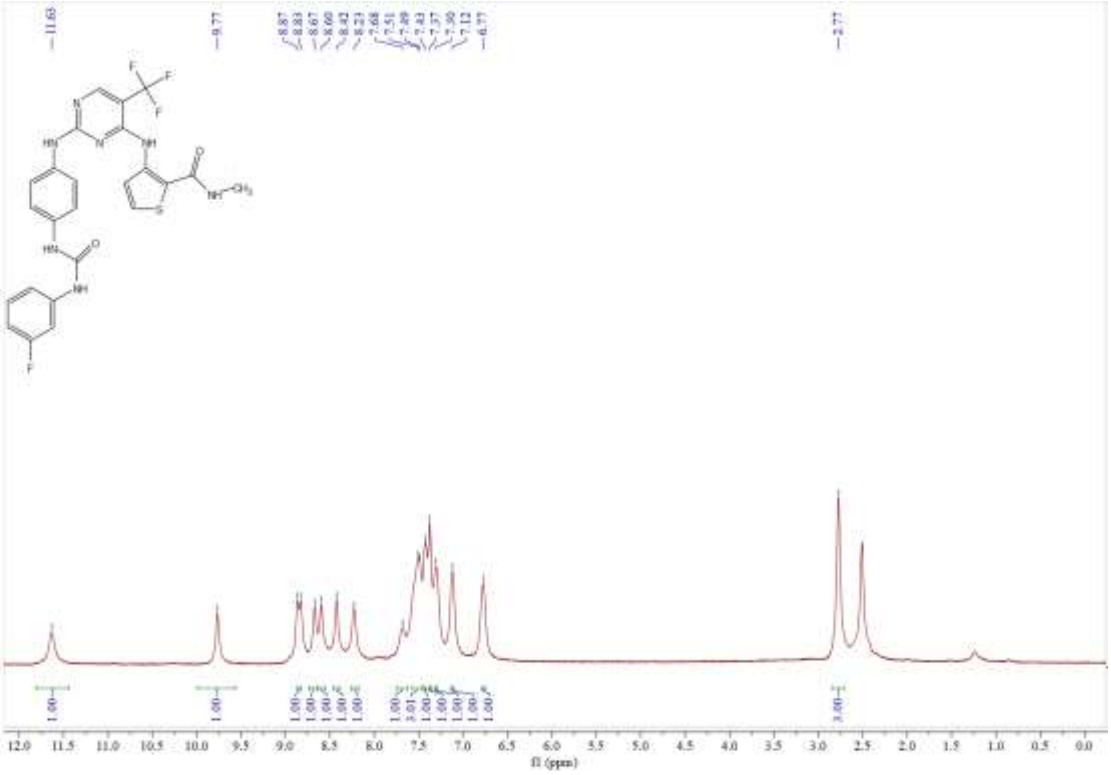


**Figure S90**. $^{1}$H NMR spectrum of compound **9w**

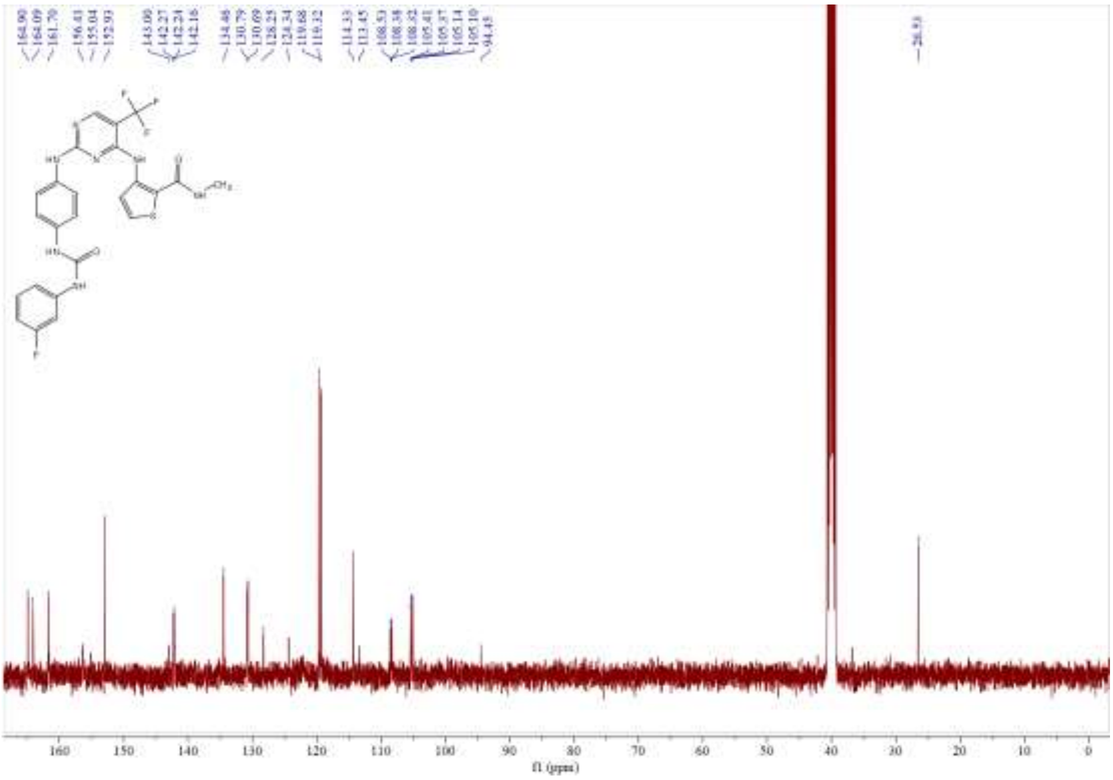

**Figure S91**. $^{13}C$ NMR spectrum of compound **9w**

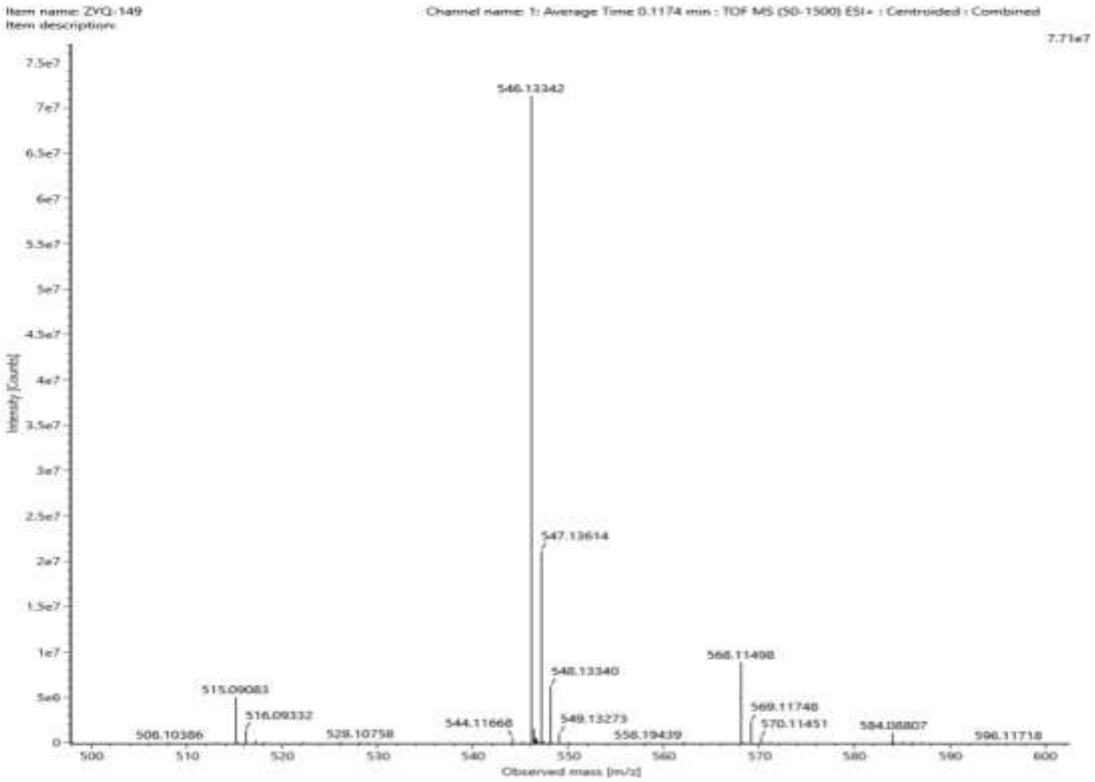


**Figure S92**. HR-MS (ESI) spectrogram of compound **9w**

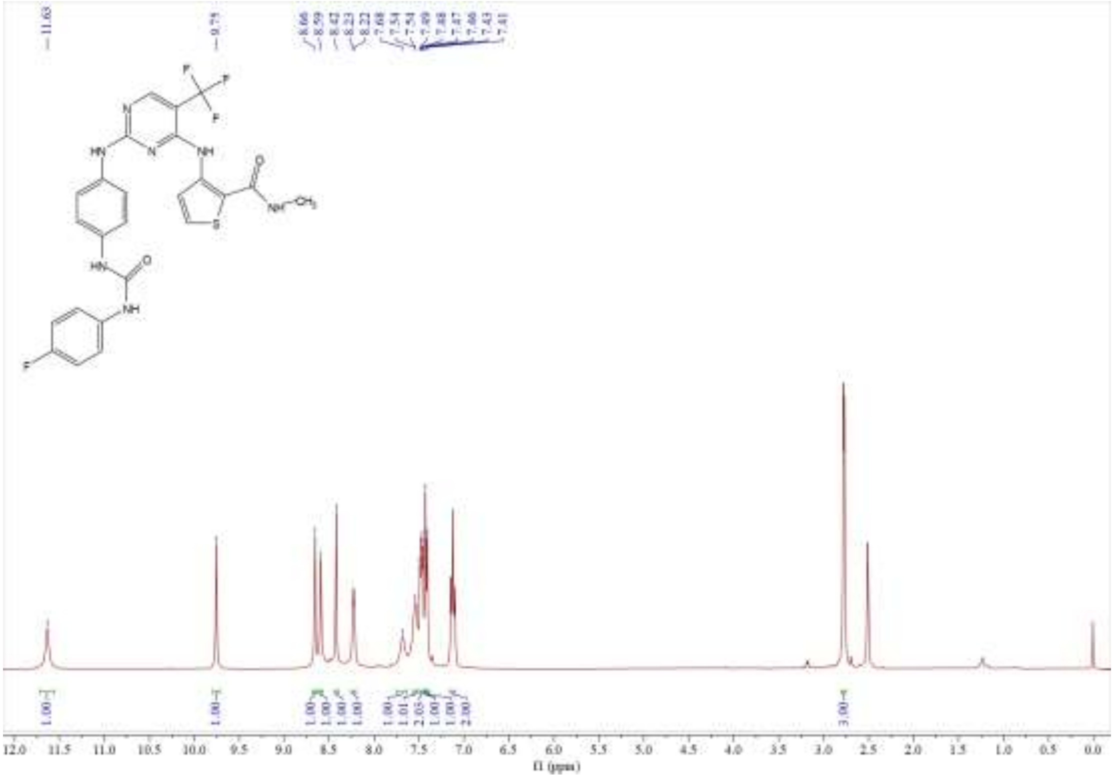

**Figure S93**. $^{1}H$ NMR spectrum of compound **9x**

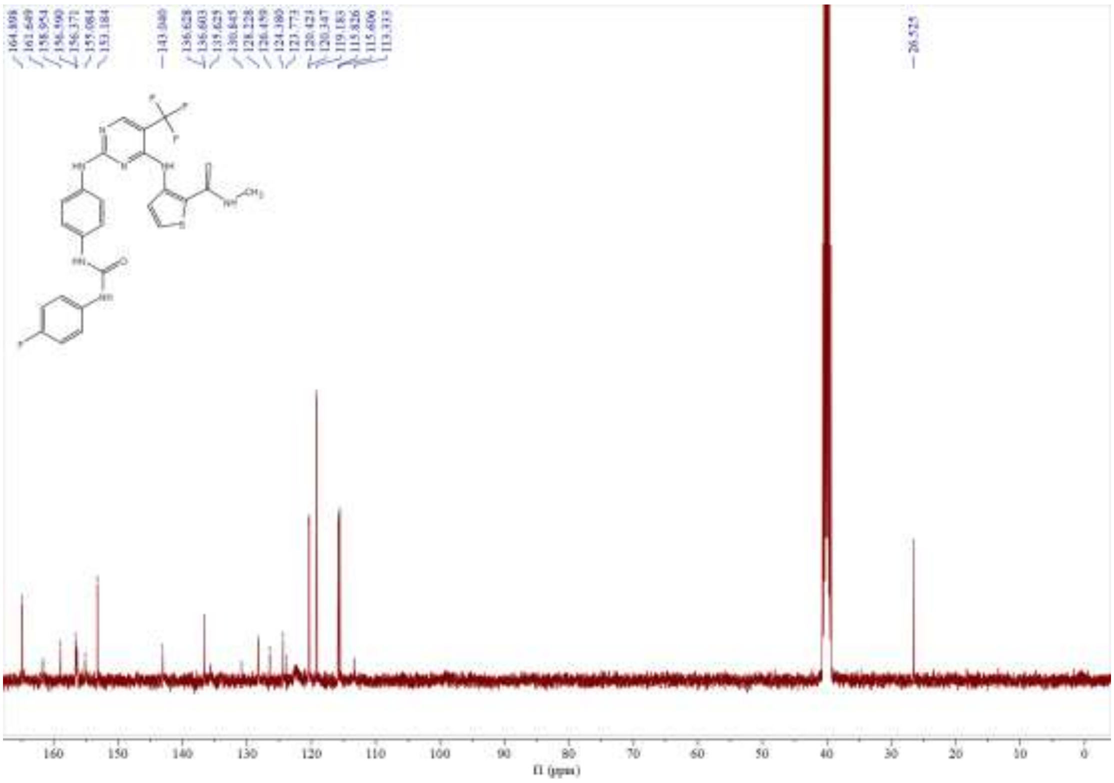

**Figure S94**. $^{13}C$ NMR spectrum of compound **9x**

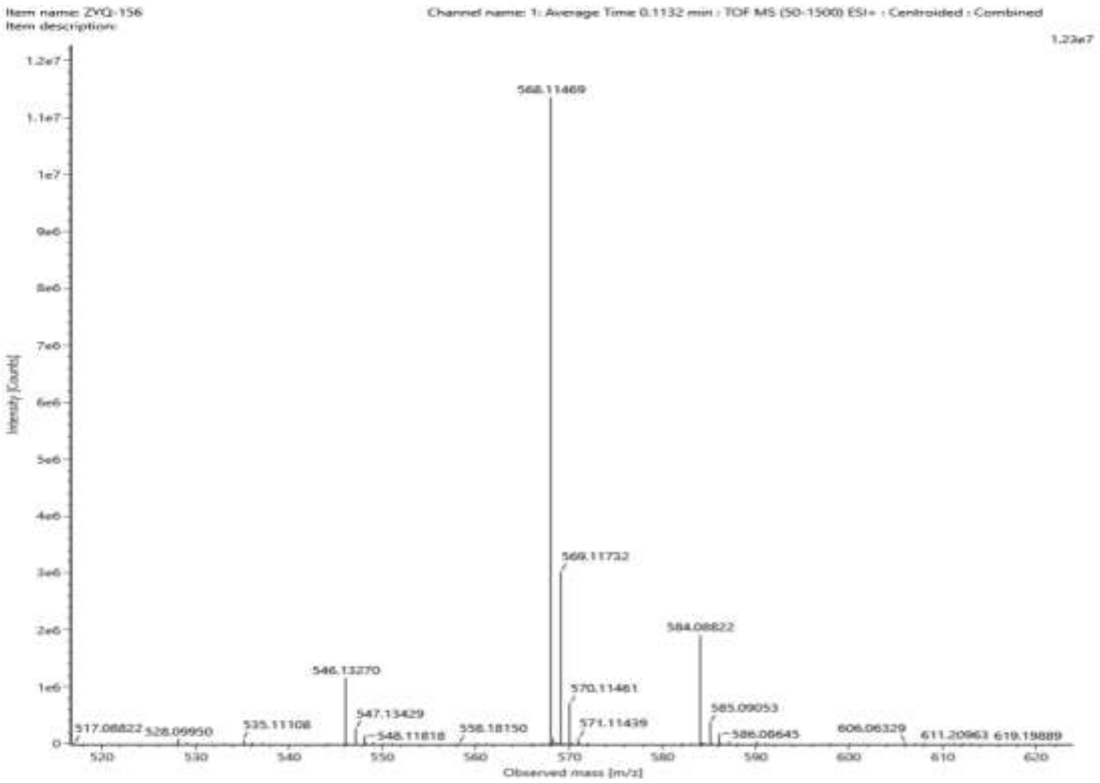


**Figure S95**. HR-MS (ESI) spectrogram of compound **9x**

**Predict ADMET Compound 9c**

ADMETlab 2.0

CNC(=O)c1sccc1N=c1[nH]c(=Nc2ccc(NC(=O)c3cccc(F)c3)cc2)[nH]cc1C(F)(F)F

## 1. Physicochemical Property

| Property | Value | Comment |
|---|---|---|
| Molecular Weight | 530.11 | Contain hydrogen atoms. Optimal:100~600 |
| Volume | 481.501 | Van der Waals volume |
| Density | 1.101 | Density = MW / Volume |
| nHA | 8 | Number of hydrogen bond acceptors. Optimal:0~12 |
| nHD | 4 | Number of hydrogen bond donors. Optimal:0~7 |
| nRot | 8 | Number of rotatable bonds. Optimal:0~11 |
| nRing | 4 | Number of rings. Optimal:0~6 |
| MaxRing | 6 | Number of atoms in the biggest ring. Optimal:0~18 |
| nHet | 13 | Number of heteroatoms. Optimal:1~15 |
| fChar | 0 | Formal charge. Optimal:-4 ~4 |
| nRig | 27 | Number of rigid bonds. Optimal:0~30 |
| Flexibility | 0.296 | Flexibility = nRot /nRig |
| Stereo Centers | 0 | Optimal: ≤ 2 |
| TPSA | 114.5 | Topological Polar Surface Area. Optimal:0~140 |
| logS | -4.816 | Log of the aqueous solubility. Optimal: -4~0.5 log mol/L |
| logP | 4.158 | Log of the octanol/water partition coefficient. Optimal: 0~3 |
| logD | 3.637 | logP at physiological pH 7.4. Optimal: 1~3 |

## 2. Medicinal Chemistry

| Property | Value | Decision | Comment |
|---|---|---|---|
| QED | 0.282 | ● | ■ A measure of drug-likeness based on the concept of desirability; ■ Attractive: > 0.67; unattractive: 0.49~0.67; too complex: < 0.34 |
| SAscore | 3.328 | ● | ■ Synthetic accessibility score is designed to estimate ease of synthesis of drug-like molecules. ■ SAscore ≥ 6, difficult to synthesize; SAscore <6, easy to synthesize |
| Fsp3 | 0.083 | ● | ■ The number of sp3 hybridized carbons / total carbon count, correlating with melting point and solubility. ■ $Fsp^3$ ≥0.42 is considered a suitable value. |
| MCE-18 | 26.0 | ● | ■ MCE-18 stands for medicinal chemistry evolution. ■ MCE-18≥45 is considered a suitable value. |

ADMETlab 2.0

Information Classification: General

| | | | |
|---|---|---|---|
| NPscore | -1.424 | - | ■ Natural product-likeness score.<br>■ This score is typically in the range from –5 to 5. The higher the score is, the higher the probability is that the molecule is a NP. |
| Lipinski Rule | Accepted | ● | ■ MW ≤ 500; logP ≤ 5; Hacc ≤ 10; Hdon ≤ 5<br>■ If two properties are out of range, a poor absorption or permeability is possible, one is acceptable. |
| Pfizer Rule | Accepted | ● | logP > 3; TPSA < 75<br>Compounds with a high log P (>3) and low TPSA (<75) are likely to be toxic. |
| GSK Rule | Rejected | ● | ■ MW ≤ 400; logP ≤ 4<br>■ Compounds satisfying the GSK rule may have a more favorable ADMET profile |
| Golden Triangle | Rejected | ● | ■ 200 ≤ MW ≤ 50; -2 ≤ logD ≤ 5<br>■ Compounds satisfying the Golden Triangle rule may have a more favorable ADMET profile. |
| PAINS | 0 alerts | - | Pan Assay Interference Compounds, frequent hitters, Alpha-screen artifacts and reactive compound. |
| ALARM NMR | 3 alerts | - | Thiol reactive compounds. |
| BMS | 0 alerts | - | Undesirable, reactive compounds. |
| Chelator Rule | 0 alerts | - | Chelating compounds. |

## 3. Absorption

| Property | Value | Decision | Comment |
|---|---|---|---|
| Caco-2 Permeability | -5.789 | ● | Optimal: higher than -5.15 Log unit |
| MDCK Permeability | 2.6e-05 | ● | ■ low permeability: < 2 × $10^{-6}$ cm/s<br>■ medium permeability: 2–20 × $10^{-6}$ cm/s<br>■ high passive permeability: > 20 × $10^{-6}$ cm/s |
| Pgp-inhibitor | 0.798 | ● | ■ Category 1: Inhibitor; Category 0: Non-inhibitor;<br>■ The output value is the probability of being Pgp-inhibitor |
| Pgp-substrate | 0.033 | ● | ■ Category 1: substrate; Category 0: Non-substrate;<br>■ The output value is the probability of being Pgp-substrate |
| HIA | 0.297 | ● | ■ Human Intestinal Absorption<br>■ Category 1: HIA+( HIA < 30%); Category 0: HIA-( HIA < 30%); The output value is the probability of being HIA+ |
| $F_{20\%}$ | 0.001 | ● | ■ 20% Bioavailability<br>■ Category 1: $F_{20\%}$+ (bioavailability < 20%); Category 0: $F_{20\%}$- (bioavailability ≥ 20%); The output value is the probability of being $F_{20\%}$+ |

ADMETlab 2.0

| | | | |
|---|---|---|---|
| $F_{30\%}$ | 0.002 | ● | ■ 30% Bioavailability<br>■ Category 1: $F_{30\%}$+ (bioavailability < 30%); Category 0: $F_{30\%}$- (bioavailability ≥ 30%); The output value is the probability of being $F_{30\%}$+ |

## 4. Distribution

| Property | Value | Decision | Comment |
|---|---|---|---|
| PPB | 98.63% | ● | ■ Plasma Protein Binding<br>■ Optimal: < 90%. Drugs with high protein-bound may have a low therapeutic index. |
| VD | 0.775 | ● | ■ Volume Distribution<br>■ Optimal: 0.04-20L/kg |
| BBB Penetration | 0.12 | ● | ■ Blood-Brain Barrier Penetration<br>■ Category 1: BBB+; Category 0: BBB-; The output value is the probability of being BBB+ |
| Fu | 1.197% | ● | ■ The fraction unbound in plasms<br>■ Low: <5%; Middle: 5~20%; High: > 20% |

## 5. Metabolism

| Property | Value | Comment |
|---|---|---|
| CYP1A2 inhibitor | 0.616 | ■ Category 1: Inhibitor; Category 0: Non-inhibitor;<br>■ The output value is the probability of being inhibitor. |
| CYP1A2 substrate | 0.919 | ■ Category 1: Substrate; Category 0: Non-substrate;<br>■ The output value is the probability of being substrate. |
| CYP2C19 inhibitor | 0.953 | ■ Category 1: Inhibitor; Category 0: Non-inhibitor;<br>■ The output value is the probability of being inhibitor. |
| CYP2C19 substrate | 0.051 | ■ Category 1: Substrate; Category 0: Non-substrate;<br>■ The output value is the probability of being substrate. |
| CYP2C9 inhibitor | 0.933 | ■ Category 1: Inhibitor; Category 0: Non-inhibitor;<br>■ The output value is the probability of being inhibitor. |
| CYP2C9 substrate | 0.034 | ■ Category 1: Substrate; Category 0: Non-substrate;<br>■ The output value is the probability of being substrate. |
| CYP2D6 inhibitor | 0.062 | ■ Category 1: Inhibitor; Category 0: Non-inhibitor;<br>■ The output value is the probability of being inhibitor. |
| CYP2D6 substrate | 0.012 | ■ Category 1: Substrate; Category 0: Non-substrate;<br>■ The output value is the probability of being substrate. |
| CYP3A4 inhibitor | 0.908 | ■ Category 1: Inhibitor; Category 0: Non-inhibitor;<br>■ The output value is the probability of being inhibitor. |
| CYP3A4 substrate | 0.694 | ■ Category 1: Substrate; Category 0: Non-substrate;<br>■ The output value is the probability of being substrate. |

ADMETlab 2.0

## 6. Excretion

| Property | Value | Decision | Comment |
|---|---|---|---|
| CL | 4.699 | ● (red) | ■ Clearance<br>■ High: >15 mL/min/kg; moderate: 5-15 mL/min/kg; low: <5 mL/min/kg |
| $T_{1/2}$ | 0.113 | - | ■ Category 1: long half-life ; Category 0: short half-life;<br>■ long half-life: >3h; short half-life: <3h<br>■ The output value is the probability of having long half-life. |

## 7. Toxicity

| Property | Value | Decision | Comment |
|---|---|---|---|
| hERG Blockers | 0.913 | ● (red) | ■ Category 1: active; Category 0: inactive;<br>■ The output value is the probability of being active. |
| H-HT | 0.997 | ● (red) | ■ Human Hepatotoxicity<br>■ Category 1: H-HT positive(+); Category 0: H-HT negative(-);<br>■ The output value is the probability of being toxic. |
| DILI | 0.984 | ● (red) | ■ Drug Induced Liver Injury.<br>■ Category 1: drugs with a high risk of DILI; Category 0: drugs with no risk of DILI. The output value is the probability of being toxic. |
| AMES Toxicity | 0.912 | ● (red) | ■ Category 1: Ames positive(+); Category 0: Ames negative(-);<br>■ The output value is the probability of being toxic. |
| Rat Oral Acute Toxicity | 0.978 | ● (red) | ■ Category 0: low-toxicity; Category 1: high-toxicity;<br>■ The output value is the probability of being highly toxic. |
| FDAMDD | 0.955 | ● (red) | ■ Maximum Recommended Daily Dose<br>■ Category 1: FDAMDD (+); Category 0: FDAMDD (-)<br>■ The output value is the probability of being positive. |
| Skin Sensitization | 0.093 | ● (green) | ■ Category 1: Sensitizer; Category 0: Non-sensitizer;<br>■ The output value is the probability of being sensitizer. |
| Carcinogencity | 0.175 | ● (green) | ■ Category 1: carcinogens; Category 0: non-carcinogens;<br>■ The output value is the probability of being toxic. |
| Eye Corrosion | 0.003 | ● (green) | ■ Category 1: corrosives ; Category 0: noncorrosives<br>■ The output value is the probability of being corrosives. |
| Eye Irritation | 0.008 | ● (green) | ■ Category 1: irritants ; Category 0: nonirritants<br>■ The output value is the probability of being irritants. |

ADMETlab 2.0

| | | | |
|---|---|---|---|
| Respiratory Toxicity | 0.949 | ● | ■ Category 1: respiratory toxicants; Category 0: respiratory nontoxicants<br>■ The output value is the probability of being toxic. |

## 8. Environmental toxicity

| Property | Value | Comment |
|---|---|---|
| Bioconcentration Factors | 0.928 | ■ Bioconcentration factors are used for considering secondary poisoning potential and assessing risks to human health via the food chain.<br>■ The unit is –log10[(mg/L)/(1000*MW)] |
| $IGC_{50}$ | 4.31 | ■ Tetrahymena pyriformis 50 percent growth inhibition concentration<br>■ The unit is –log10[(mg/L)/(1000*MW)] |
| $LC_{50}FM$ | 5.695 | ■ 96-hour fathead minnow 50 percent lethal concentration<br>■ The unit is –log10[(mg/L)/(1000*MW)] |
| $LC_{50}DM$ | 6.149 | ■ 48-hour daphnia magna 50 percent lethal concentration<br>■ The unit is –log10[(mg/L)/(1000*MW)] |

## 9. Tox21 pathway

| Property | Value | Decision | Comment |
|---|---|---|---|
| NR-AR | 0.014 | ● | ■ Androgen receptor<br>■ Category 1: actives ; Category 0: inactives;<br>■ The output value is the probability of being active. |
| NR-AR-LBD | 0.011 | ● | ■ Androgen receptor ligand-binding domain<br>■ Category 1: actives ; Category 0: inactives;<br>■ The output value is the probability of being active. |
| NR-AhR | 0.979 | ● | ■ Aryl hydrocarbon receptor<br>■ Category 1: actives ; Category 0: inactives;<br>■ The output value is the probability of being active. |
| NR-Aromatase | 0.945 | ● | ■ Category 1: actives ; Category 0: inactives;<br>■ The output value is the probability of being active. |
| NR-ER | 0.551 | ● | ■ Estrogen receptor<br>■ Category 1: actives ; Category 0: inactives;<br>■ The output value is the probability of being active. |
| NR-ER-LBD | 0.008 | ● | ■ Estrogen receptor ligand-binding domain<br>■ Category 1: actives ; Category 0: inactives;<br>■ The output value is the probability of being active. |
| NR-PPAR-gamma | 0.44 | ● | ■ Peroxisome proliferator-activated receptor gamma<br>■ Category 1: actives ; Category 0: inactives;<br>■ The output value is the probability of being active. |
| SR-ARE | 0.879 | ● | ■ Antioxidant response element<br>■ Category 1: actives ; Category 0: inactives;<br>■ The output value is the probability of being active. |
| SR-ATAD5 | 0.101 | ● | ■ ATPase family AAA domain-containing protein 5<br>■ Category 1: actives ; Category 0: inactives;<br>■ The output value is the probability of being active. |

| | | | |
|---|---|---|---|
| SR-HSE | 0.051 | | ■ Heat shock factor response element<br>■ Category 1: actives ; Category 0: inactives;<br>■ The output value is the probability of being active. |
| SR-MMP | 0.951 | | ■ Mitochondrial membrane potential<br>■ Category 1: actives ; Category 0: inactives;<br>■ The output value is the probability of being active. |
| SR-p53 | 0.932 | | ■ Category 1: actives ; Category 0: inactives;<br>■ The output value is the probability of being active. |

## 10. Toxicophore Rules

| Property | Value | Comment |
|---|---|---|
| Acute Toxicity Rule | 0 alerts | ■ 20 substructures<br>■ acute toxicity during oral administration |
| Genotoxic Carcinogenicity Rule | 1 alerts | ■ 117 substructures<br>■ carcinogenicity or mutagenicity |
| NonGenotoxic Carcinogenicity Rule | 1 alerts | ■ 23 substructures<br>■ carcinogenicity through nongenotoxic mechanisms |
| Skin Sensitization Rule | 4 alerts | ■ 155 substructures<br>■ skin irritation |
| Aquatic Toxicity Rule | 3 alerts | ■ 99 substructures<br>■ toxicity to liquid(water) |
| NonBiodegradable Rule | 2 alerts | ■ 19 substructures<br>■ non-biodegradable |
| SureChEMBL Rule | 0 alerts | ■ 164 substructures<br>■ MedChem unfriendly status |

ADMETlab 2.0

**Predict ADMET Compound 9e**

ADMETlab 2.0

CNC(=O)c1sccc1N=c1[nH]c(=Nc2ccc(NC(=O)c3ccc(F)c(F)c3)cc2)[nH]cc1C(F)(F)F

## 1. Physicochemical Property

| Property | Value | Comment |
|---|---|---|
| Molecular Weight | 548.11 | Contain hydrogen atoms. Optimal:100~600 |
| Volume | 487.568 | Van der Waals volume |
| Density | 1.124 | Density = MW / Volume |
| nHA | 8 | Number of hydrogen bond acceptors. Optimal:0~12 |
| nHD | 4 | Number of hydrogen bond donors. Optimal:0~7 |
| nRot | 8 | Number of rotatable bonds. Optimal:0~11 |
| nRing | 4 | Number of rings. Optimal:0~6 |
| MaxRing | 6 | Number of atoms in the biggest ring. Optimal:0~18 |
| nHet | 14 | Number of heteroatoms. Optimal:1~15 |
| fChar | 0 | Formal charge. Optimal:-4 ~4 |
| nRig | 27 | Number of rigid bonds. Optimal:0~30 |
| Flexibility | 0.296 | Flexibility = nRot /nRig |
| Stereo Centers | 0 | Optimal: ≤ 2 |
| TPSA | 114.5 | Topological Polar Surface Area. Optimal:0~140 |
| logS | -4.878 | Log of the aqueous solubility. Optimal: -4~0.5 log mol/L |
| logP | 4.347 | Log of the octanol/water partition coefficient. Optimal: 0~3 |
| logD | 3.604 | logP at physiological pH 7.4. Optimal: 1~3 |

## 2. Medicinal Chemistry

| Property | Value | Decision | Comment |
|---|---|---|---|
| QED | 0.269 | ● | ■ A measure of drug-likeness based on the concept of desirability; ■ Attractive: > 0.67; unattractive: 0.49~0.67; too complex: < 0.34 |
| SAscore | 3.374 | ● | ■ Synthetic accessibility score is designed to estimate ease of synthesis of drug-like molecules. ■ SAscore ≥ 6, difficult to synthesize; SAscore <6, easy to synthesize |
| Fsp3 | 0.083 | ● | ■ The number of sp3 hybridized carbons / total carbon count, correlating with melting point and solubility. ■ Fsp$^3$ ≥0.42 is considered a suitable value. |
| MCE-18 | 27.0 | ● | ■ MCE-18 stands for medicinal chemistry evolution. ■ MCE-18≥45 is considered a suitable value. |

| | | | |
|---|---|---|---|
| NPscore | -1.508 | - | ■ Natural product-likeness score.<br>■ This score is typically in the range from –5 to 5. The higher the score is, the higher the probability is that the molecule is a NP. |
| Lipinski Rule | Accepted | ● | ■ MW ≤ 500; logP ≤ 5; Hacc ≤ 10; Hdon ≤ 5<br>■ If two properties are out of range, a poor absorption or permeability is possible, one is acceptable. |
| Pfizer Rule | Accepted | ● | logP > 3; TPSA < 75<br>Compounds with a high log P (>3) and low TPSA (<75) are likely to be toxic. |
| GSK Rule | Rejected | ● | ■ MW ≤ 400; logP ≤ 4<br>■ Compounds satisfying the GSK rule may have a more favorable ADMET profile |
| Golden Triangle | Rejected | ● | ■ 200 ≤ MW ≤ 50; -2 ≤ logD ≤ 5<br>■ Compounds satisfying the Golden Triangle rule may have a more favorable ADMET profile. |
| PAINS | 0 alerts | - | Pan Assay Interference Compounds, frequent hitters, Alpha-screen artifacts and reactive compound. |
| ALARM NMR | 3 alerts | - | Thiol reactive compounds. |
| BMS | 0 alerts | - | Undesirable, reactive compounds. |
| Chelator Rule | 0 alerts | - | Chelating compounds. |

## 3. Absorption

| Property | Value | Decision | Comment |
|---|---|---|---|
| Caco-2 Permeability | -5.859 | ● | Optimal: higher than -5.15 Log unit |
| MDCK Permeability | 2.5e-05 | ● | ■ low permeability: < 2 × $10^{-6}$ cm/s<br>■ medium permeability: 2–20 × $10^{-6}$ cm/s<br>■ high passive permeability: > 20 × $10^{-6}$ cm/s |
| Pgp-inhibitor | 0.901 | ● | ■ Category 1: Inhibitor; Category 0: Non-inhibitor;<br>■ The output value is the probability of being Pgp-inhibitor |
| Pgp-substrate | 0.014 | ● | ■ Category 1: substrate; Category 0: Non-substrate;<br>■ The output value is the probability of being Pgp-substrate |
| HIA | 0.048 | ● | ■ Human Intestinal Absorption<br>■ Category 1: HIA+( HIA < 30%); Category 0: HIA-( HIA < 30%); The output value is the probability of being HIA+ |
| $F_{20\%}$ | 0.002 | ● | ■ 20% Bioavailability<br>■ Category 1: $F_{20\%}$+ (bioavailability < 20%); Category 0: $F_{20\%}$- (bioavailability ≥ 20%); The output value is the probability of being $F_{20\%}$+ |

ADMETlab 2.0

| | | | |
|---|---|---|---|
| $F_{30\%}$ | 0.003 | 🟢 | ■ 30% Bioavailability<br>■ Category 1: $F_{30\%}$+ (bioavailability < 30%); Category 0: $F_{30\%}$- (bioavailability ≥ 30%); The output value is the probability of being $F_{30\%}$+ |

## 4. Distribution

| Property | Value | Decision | Comment |
|---|---|---|---|
| PPB | 99.11% | 🔴 | ■ Plasma Protein Binding<br>■ Optimal: < 90%. Drugs with high protein-bound may have a low therapeutic index. |
| VD | 0.879 | 🟢 | ■ Volume Distribution<br>■ Optimal: 0.04-20L/kg |
| BBB Penetration | 0.055 | 🟢 | ■ Blood-Brain Barrier Penetration<br>■ Category 1: BBB+; Category 0: BBB-; The output value is the probability of being BBB+ |
| Fu | 0.947% | 🔴 | ■ The fraction unbound in plasms<br>■ Low: <5%; Middle: 5~20%; High: > 20% |

## 5. Metabolism

| Property | Value | Comment |
|---|---|---|
| CYP1A2 inhibitor | 0.6 | ■ Category 1: Inhibitor; Category 0: Non-inhibitor;<br>■ The output value is the probability of being inhibitor. |
| CYP1A2 substrate | 0.899 | ■ Category 1: Substrate; Category 0: Non-substrate;<br>■ The output value is the probability of being substrate. |
| CYP2C19 inhibitor | 0.956 | ■ Category 1: Inhibitor; Category 0: Non-inhibitor;<br>■ The output value is the probability of being inhibitor. |
| CYP2C19 substrate | 0.053 | ■ Category 1: Substrate; Category 0: Non-substrate;<br>■ The output value is the probability of being substrate. |
| CYP2C9 inhibitor | 0.924 | ■ Category 1: Inhibitor; Category 0: Non-inhibitor;<br>■ The output value is the probability of being inhibitor. |
| CYP2C9 substrate | 0.041 | ■ Category 1: Substrate; Category 0: Non-substrate;<br>■ The output value is the probability of being substrate. |
| CYP2D6 inhibitor | 0.065 | ■ Category 1: Inhibitor; Category 0: Non-inhibitor;<br>■ The output value is the probability of being inhibitor. |
| CYP2D6 substrate | 0.015 | ■ Category 1: Substrate; Category 0: Non-substrate;<br>■ The output value is the probability of being substrate. |
| CYP3A4 inhibitor | 0.902 | ■ Category 1: Inhibitor; Category 0: Non-inhibitor;<br>■ The output value is the probability of being inhibitor. |
| CYP3A4 substrate | 0.67 | ■ Category 1: Substrate; Category 0: Non-substrate;<br>■ The output value is the probability of being substrate. |

## 6. Excretion

| Property | Value | Decision | Comment |
|---|---|---|---|
| CL | 4.418 | 🔴 | ■ Clearance<br>■ High: >15 mL/min/kg; moderate: 5-15 mL/min/kg; low: <5 mL/min/kg |
| $T_{1/2}$ | 0.074 | - | ■ Category 1: long half-life ; Category 0: short half-life;<br>■ long half-life: >3h; short half-life: <3h<br>■ The output value is the probability of having long half-life. |

## 7. Toxicity

| Property | Value | Decision | Comment |
|---|---|---|---|
| hERG Blockers | 0.921 | 🔴 | ■ Category 1: active; Category 0: inactive;<br>■ The output value is the probability of being active. |
| H-HT | 0.999 | 🔴 | ■ Human Hepatotoxicity<br>■ Category 1: H-HT positive(+); Category 0: H-HT negative(-);<br>■ The output value is the probability of being toxic. |
| DILI | 0.983 | 🔴 | ■ Drug Induced Liver Injury.<br>■ Category 1: drugs with a high risk of DILI; Category 0: drugs with no risk of DILI. The output value is the probability of being toxic. |
| AMES Toxicity | 0.943 | 🔴 | ■ Category 1: Ames positive(+); Category 0: Ames negative(-);<br>■ The output value is the probability of being toxic. |
| Rat Oral Acute Toxicity | 0.984 | 🔴 | ■ Category 0: low-toxicity; Category 1: high-toxicity;<br>■ The output value is the probability of being highly toxic. |
| FDAMDD | 0.941 | 🔴 | ■ Maximum Recommended Daily Dose<br>■ Category 1: FDAMDD (+); Category 0: FDAMDD (-)<br>■ The output value is the probability of being positive. |
| Skin Sensiti zation | 0.064 | 🟢 | ■ Category 1: Sensitizer; Category 0: Non-sensitizer;<br>■ The output value is the probability of being sensitizer. |
| Carcinogen city | 0.183 | 🟢 | ■ Category 1: carcinogens; Category 0: non-carcinogens;<br>■ The output value is the probability of being toxic. |
| Eye Corrosion | 0.003 | 🟢 | ■ Category 1: corrosives ; Category 0: noncorrosives<br>■ The output value is the probability of being corrosives. |
| Eye Irritation | 0.008 | 🟢 | ■ Category 1: irritants ; Category 0: nonirritants<br>■ The output value is the probability of being irritants. |

ADMETlab 2.0

| | | | |
|---|---|---|---|
| Respiratory Toxicity | 0.955 | 🔴 | ■ Category 1: respiratory toxicants; Category 0: respiratory nontoxicants<br>■ The output value is the probability of being toxic. |

## 8. Environmental toxicity

| Property | Value | Comment |
|---|---|---|
| Bioconcentration Factors | 1.246 | ■ Bioconcentration factors are used for considering secondary poisoning potential and assessing risks to human health via the food chain.<br>■ The unit is –log10[(mg/L)/(1000*MW)] |
| $IGC_{50}$ | 4.316 | ■ Tetrahymena pyriformis 50 percent growth inhibition concentration<br>■ The unit is –log10[(mg/L)/(1000*MW)] |
| $LC_{50}FM$ | 5.734 | ■ 96-hour fathead minnow 50 percent lethal concentration<br>■ The unit is –log10[(mg/L)/(1000*MW)] |
| $LC_{50}DM$ | 6.363 | ■ 48-hour daphnia magna 50 percent lethal concentration<br>■ The unit is –log10[(mg/L)/(1000*MW)] |

## 9. Tox21 pathway

| Property | Value | Decision | Comment |
|---|---|---|---|
| NR-AR | 0.042 | 🟢 | ■ Androgen receptor<br>■ Category 1: actives ; Category 0: inactives;<br>■ The output value is the probability of being active. |
| NR-AR-LBD | 0.079 | 🟢 | ■ Androgen receptor ligand-binding domain<br>■ Category 1: actives ; Category 0: inactives;<br>■ The output value is the probability of being active. |
| NR-AhR | 0.97 | 🔴 | ■ Aryl hydrocarbon receptor<br>■ Category 1: actives ; Category 0: inactives;<br>■ The output value is the probability of being active. |
| NR-Aromatase | 0.961 | 🔴 | ■ Category 1: actives ; Category 0: inactives;<br>■ The output value is the probability of being active. |
| NR-ER | 0.714 | 🔴 | ■ Estrogen receptor<br>■ Category 1: actives ; Category 0: inactives;<br>■ The output value is the probability of being active. |
| NR-ER-LBD | 0.019 | 🟢 | ■ Estrogen receptor ligand-binding domain<br>■ Category 1: actives ; Category 0: inactives;<br>■ The output value is the probability of being active. |
| NR-PPAR-gamma | 0.583 | 🟠 | ■ Peroxisome proliferator-activated receptor gamma<br>■ Category 1: actives ; Category 0: inactives;<br>■ The output value is the probability of being active. |
| SR-ARE | 0.899 | 🔴 | ■ Antioxidant response element<br>■ Category 1: actives ; Category 0: inactives;<br>■ The output value is the probability of being active. |
| SR-ATAD5 | 0.274 | 🟢 | ■ ATPase family AAA domain-containing protein 5<br>■ Category 1: actives ; Category 0: inactives;<br>■ The output value is the probability of being active. |

ADMETlab 2.0

| | | | |
|---|---|---|---|
| SR-HSE | 0.051 | ● | ■ Heat shock factor response element<br>■ Category 1: actives ; Category 0: inactives;<br>■ The output value is the probability of being active. |
| SR-MMP | 0.959 | ● | ■ Mitochondrial membrane potential<br>■ Category 1: actives ; Category 0: inactives;<br>■ The output value is the probability of being active. |
| SR-p53 | 0.955 | ● | ■ Category 1: actives ; Category 0: inactives;<br>■ The output value is the probability of being active. |

## 10. Toxicophore Rules

| Property | Value | Comment |
|---|---|---|
| Acute Toxicity Rule | 0 alerts | ■ 20 substructures<br>■ acute toxicity during oral administration |
| Genotoxic Carcinogenicity Rule | 1 alerts | ■ 117 substructures<br>■ carcinogenicity or mutagenicity |
| NonGenotoxic Carcinogenicity Rule | 0 alerts | ■ 23 substructures<br>■ carcinogenicity through nongenotoxic mechanisms |
| Skin Sensitization Rule | 4 alerts | ■ 155 substructures<br>■ skin irritation |
| Aquatic Toxicity Rule | 3 alerts | ■ 99 substructures<br>■ toxicity to liquid(water) |
| NonBiodegradable Rule | 2 alerts | ■ 19 substructures<br>■ non-biodegradable |
| SureChEMBL Rule | 0 alerts | ■ 164 substructures<br>■ MedChem unfriendly status |

ADMETlab 2.0

**Predict ADMET Compound 9h**

ADMETlab 2.0

CNC(=O)c1sccc1N=c1[nH]c(=Nc2ccc(NC(=O)c3cc(OC)c(OC)c(OC)c3)cc2)[nH]cc1C(F)(F)F

## 1. Physicochemical Property

| Property | Value | Comment |
|---|---|---|
| Molecular Weight | 602.16 | Contain hydrogen atoms. Optimal:100~600 |
| Volume | 553.692 | Van der Waals volume |
| Density | 1.088 | Density = MW / Volume |
| nHA | 11 | Number of hydrogen bond acceptors. Optimal:0~12 |
| nHD | 4 | Number of hydrogen bond donors. Optimal:0~7 |
| nRot | 11 | Number of rotatable bonds. Optimal:0~11 |
| nRing | 4 | Number of rings. Optimal:0~6 |
| MaxRing | 6 | Number of atoms in the biggest ring. Optimal:0~18 |
| nHet | 15 | Number of heteroatoms. Optimal:1~15 |
| fChar | 0 | Formal charge. Optimal:-4 ~4 |
| nRig | 27 | Number of rigid bonds. Optimal:0~30 |
| Flexibility | 0.407 | Flexibility = nRot /nRig |
| Stereo Centers | 0 | Optimal: ≤ 2 |
| TPSA | 142.19 | Topological Polar Surface Area. Optimal:0~140 |
| logS | -4.714 | Log of the aqueous solubility. Optimal: -4~0.5 log mol/L |
| logP | 3.625 | Log of the octanol/water partition coefficient. Optimal: 0~3 |
| logD | 3.366 | logP at physiological pH 7.4. Optimal: 1~3 |

## 2. Medicinal Chemistry

| Property | Value | Decision | Comment |
|---|---|---|---|
| QED | 0.235 | 🔴 | ■ A measure of drug-likeness based on the concept of desirability; ■ Attractive: > 0.67; unattractive: 0.49~0.67; too complex: < 0.34 |
| SAscore | 3.406 | 🟢 | ■ Synthetic accessibility score is designed to estimate ease of synthesis of drug-like molecules. ■ SAscore ≥ 6, difficult to synthesize; SAscore <6, easy to synthesize |
| Fsp3 | 0.185 | 🔴 | ■ The number of sp3 hybridized carbons / total carbon count, correlating with melting point and solubility. ■ Fsp$^3$ ≥0.42 is considered a suitable value. |
| MCE-18 | 28.0 | 🔴 | ■ MCE-18 stands for medicinal chemistry evolution. ■ MCE-18≥45 is considered a suitable value. |

ADMETlab 2.0

| | | | |
|---|---|---|---|
| NPscore | -0.93 | - | ■ Natural product-likeness score.<br>■ This score is typically in the range from –5 to 5. The higher the score is, the higher the probability is that the molecule is a NP. |
| Lipinski Rule | Rejected | ● | ■ MW ≤ 500; logP ≤ 5; Hacc ≤ 10; Hdon ≤ 5<br>■ If two properties are out of range, a poor absorption or permeability is possible, one is acceptable. |
| Pfizer Rule | Accepted | ● | logP > 3; TPSA < 75<br>Compounds with a high log P (>3) and low TPSA (<75) are likely to be toxic. |
| GSK Rule | Rejected | ● | ■ MW ≤ 400; logP ≤ 4<br>■ Compounds satisfying the GSK rule may have a more favorable ADMET profile |
| Golden Triangle | Rejected | ● | ■ 200 ≤ MW ≤ 50; -2 ≤ logD ≤ 5<br>■ Compounds satisfying the Golden Triangle rule may have a more favorable ADMET profile. |
| PAINS | 0 alerts | - | Pan Assay Interference Compounds, frequent hitters, Alpha-screen artifacts and reactive compound. |
| ALARM NMR | 5 alerts | - | Thiol reactive compounds. |
| BMS | 0 alerts | - | Undesirable, reactive compounds. |
| Chelator Rule | 0 alerts | - | Chelating compounds. |

## 3. Absorption

| Property | Value | Decision | Comment |
|---|---|---|---|
| Caco-2 Permeability | -5.852 | ● | Optimal: higher than -5.15 Log unit |
| MDCK Permeability | 1.9e-05 | ● | ■ low permeability: $< 2 \times 10^{-6}$ cm/s<br>■ medium permeability: $2–20 \times 10^{-6}$ cm/s<br>■ high passive permeability: $> 20 \times 10^{-6}$ cm/s |
| Pgp-inhibitor | 0.973 | ● | ■ Category 1: Inhibitor; Category 0: Non-inhibitor;<br>■ The output value is the probability of being Pgp-inhibitor |
| Pgp-substrate | 0.804 | ● | ■ Category 1: substrate; Category 0: Non-substrate;<br>■ The output value is the probability of being Pgp-substrate |
| HIA | 0.232 | ● | ■ Human Intestinal Absorption<br>■ Category 1: HIA+( HIA < 30%); Category 0: HIA-( HIA < 30%); The output value is the probability of being HIA+ |
| $F_{20\%}$ | 0.008 | ● | ■ 20% Bioavailability<br>■ Category 1: $F_{20\%}$+ (bioavailability < 20%); Category 0: $F_{20\%}$- (bioavailability ≥ 20%); The output value is the probability of being $F_{20\%}$+ |

ADMETlab 2.0

| | | | |
|---|---|---|---|
| $F_{30\%}$ | 0.009 | 🟢 | ■ 30% Bioavailability<br>■ Category 1: $F_{30\%}$+ (bioavailability < 30%); Category 0: $F_{30\%}$- (bioavailability ≥ 30%); The output value is the probability of being $F_{30\%}$+ |

## 4. Distribution

| Property | Value | Decision | Comment |
|---|---|---|---|
| PPB | 98.74% | 🔴 | ■ Plasma Protein Binding<br>■ Optimal: < 90%. Drugs with high protein-bound may have a low therapeutic index. |
| VD | 0.812 | 🟢 | ■ Volume Distribution<br>■ Optimal: 0.04-20L/kg |
| BBB Penetration | 0.039 | 🟢 | ■ Blood-Brain Barrier Penetration<br>■ Category 1: BBB+; Category 0: BBB-; The output value is the probability of being BBB+ |
| Fu | 2.216% | 🔴 | ■ The fraction unbound in plasms<br>■ Low: <5%; Middle: 5~20%; High: > 20% |

## 5. Metabolism

| Property | Value | Comment |
|---|---|---|
| CYP1A2 inhibitor | 0.405 | ■ Category 1: Inhibitor; Category 0: Non-inhibitor;<br>■ The output value is the probability of being inhibitor. |
| CYP1A2 substrate | 0.975 | ■ Category 1: Substrate; Category 0: Non-substrate;<br>■ The output value is the probability of being substrate. |
| CYP2C19 inhibitor | 0.836 | ■ Category 1: Inhibitor; Category 0: Non-inhibitor;<br>■ The output value is the probability of being inhibitor. |
| CYP2C19 substrate | 0.06 | ■ Category 1: Substrate; Category 0: Non-substrate;<br>■ The output value is the probability of being substrate. |
| CYP2C9 inhibitor | 0.833 | ■ Category 1: Inhibitor; Category 0: Non-inhibitor;<br>■ The output value is the probability of being inhibitor. |
| CYP2C9 substrate | 0.066 | ■ Category 1: Substrate; Category 0: Non-substrate;<br>■ The output value is the probability of being substrate. |
| CYP2D6 inhibitor | 0.009 | ■ Category 1: Inhibitor; Category 0: Non-inhibitor;<br>■ The output value is the probability of being inhibitor. |
| CYP2D6 substrate | 0.025 | ■ Category 1: Substrate; Category 0: Non-substrate;<br>■ The output value is the probability of being substrate. |
| CYP3A4 inhibitor | 0.885 | ■ Category 1: Inhibitor; Category 0: Non-inhibitor;<br>■ The output value is the probability of being inhibitor. |
| CYP3A4 substrate | 0.914 | ■ Category 1: Substrate; Category 0: Non-substrate;<br>■ The output value is the probability of being substrate. |

## 6. Excretion

| Property | Value | Decision | Comment |
|---|---|---|---|
| CL | 5.578 | ● | ■ Clearance<br>■ High: >15 mL/min/kg; moderate: 5-15 mL/min/kg; low: <5 mL/min/kg |
| $T_{1/2}$ | 0.406 | - | ■ Category 1: long half-life ; Category 0: short half-life;<br>■ long half-life: >3h; short half-life: <3h<br>■ The output value is the probability of having long half-life. |

## 7. Toxicity

| Property | Value | Decision | Comment |
|---|---|---|---|
| hERG Blockers | 0.914 | ● | ■ Category 1: active; Category 0: inactive;<br>■ The output value is the probability of being active. |
| H-HT | 0.994 | ● | ■ Human Hepatotoxicity<br>■ Category 1: H-HT positive(+); Category 0: H-HT negative(-);<br>■ The output value is the probability of being toxic. |
| DILI | 0.981 | ● | ■ Drug Induced Liver Injury.<br>■ Category 1: drugs with a high risk of DILI; Category 0: drugs with no risk of DILI. The output value is the probability of being toxic. |
| AMES Toxicity | 0.912 | ● | ■ Category 1: Ames positive(+); Category 0: Ames negative(-);<br>■ The output value is the probability of being toxic. |
| Rat Oral Acute Toxicity | 0.958 | ● | ■ Category 0: low-toxicity; Category 1: high-toxicity;<br>■ The output value is the probability of being highly toxic. |
| FDAMDD | 0.918 | ● | ■ Maximum Recommended Daily Dose<br>■ Category 1: FDAMDD (+); Category 0: FDAMDD (-)<br>■ The output value is the probability of being positive. |
| Skin Sensiti zation | 0.126 | ● | ■ Category 1: Sensitizer; Category 0: Non-sensitizer;<br>■ The output value is the probability of being sensitizer. |
| Carcinogen city | 0.074 | ● | ■ Category 1: carcinogens; Category 0: non-carcinogens;<br>■ The output value is the probability of being toxic. |
| Eye Corrosion | 0.003 | ● | ■ Category 1: corrosives ; Category 0: noncorrosives<br>■ The output value is the probability of being corrosives. |
| Eye Irritation | 0.007 | ● | ■ Category 1: irritants ; Category 0: nonirritants<br>■ The output value is the probability of being irritants. |

ADMETlab 2.0

| | | | |
|---|---|---|---|
| Respiratory Toxicity | 0.943 | ● | ■ Category 1: respiratory toxicants; Category 0: respiratory nontoxicants<br>■ The output value is the probability of being toxic. |

## 8. Environmental toxicity

| Property | Value | Comment |
|---|---|---|
| Bioconcentration Factors | 1.052 | ■ Bioconcentration factors are used for considering secondary poisoning potential and assessing risks to human health via the food chain.<br>■ The unit is –log10[(mg/L)/(1000*MW)] |
| $IGC_{50}$ | 4.09 | ■ Tetrahymena pyriformis 50 percent growth inhibition concentration<br>■ The unit is –log10[(mg/L)/(1000*MW)] |
| $LC_{50}FM$ | 6.123 | ■ 96-hour fathead minnow 50 percent lethal concentration<br>■ The unit is –log10[(mg/L)/(1000*MW)] |
| $LC_{50}DM$ | 6.143 | ■ 48-hour daphnia magna 50 percent lethal concentration<br>■ The unit is –log10[(mg/L)/(1000*MW)] |

## 9. Tox21 pathway

| Property | Value | Decision | Comment |
|---|---|---|---|
| NR-AR | 0.06 | ● | ■ Androgen receptor<br>■ Category 1: actives ; Category 0: inactives;<br>■ The output value is the probability of being active. |
| NR-AR-LBD | 0.035 | ● | ■ Androgen receptor ligand-binding domain<br>■ Category 1: actives ; Category 0: inactives;<br>■ The output value is the probability of being active. |
| NR-AhR | 0.98 | ● | ■ Aryl hydrocarbon receptor<br>■ Category 1: actives ; Category 0: inactives;<br>■ The output value is the probability of being active. |
| NR-Aromatase | 0.879 | ● | ■ Category 1: actives ; Category 0: inactives;<br>■ The output value is the probability of being active. |
| NR-ER | 0.581 | ● | ■ Estrogen receptor<br>■ Category 1: actives ; Category 0: inactives;<br>■ The output value is the probability of being active. |
| NR-ER-LBD | 0.033 | ● | ■ Estrogen receptor ligand-binding domain<br>■ Category 1: actives ; Category 0: inactives;<br>■ The output value is the probability of being active. |
| NR-PPAR-gamma | 0.53 | ● | ■ Peroxisome proliferator-activated receptor gamma<br>■ Category 1: actives ; Category 0: inactives;<br>■ The output value is the probability of being active. |
| SR-ARE | 0.892 | ● | ■ Antioxidant response element<br>■ Category 1: actives ; Category 0: inactives;<br>■ The output value is the probability of being active. |
| SR-ATAD5 | 0.824 | ● | ■ ATPase family AAA domain-containing protein 5<br>■ Category 1: actives ; Category 0: inactives;<br>■ The output value is the probability of being active. |

ADMETlab 2.0

| | | | |
|---|---|---|---|
| SR-HSE | 0.1 | ● | ■ Heat shock factor response element<br>■ Category 1: actives ; Category 0: inactives;<br>■ The output value is the probability of being active. |
| SR-MMP | 0.945 | ● | ■ Mitochondrial membrane potential<br>■ Category 1: actives ; Category 0: inactives;<br>■ The output value is the probability of being active. |
| SR-p53 | 0.959 | ● | ■ Category 1: actives ; Category 0: inactives;<br>■ The output value is the probability of being active. |

## 10. Toxicophore Rules

| Property | Value | Comment |
|---|---|---|
| Acute Toxicity Rule | 0 alerts | ■ 20 substructures<br>■ acute toxicity during oral administration |
| Genotoxic Carcinogenicity Rule | 1 alerts | ■ 117 substructures<br>■ carcinogenicity or mutagenicity |
| NonGenotoxic Carcinogenicity Rule | 0 alerts | ■ 23 substructures<br>■ carcinogenicity through nongenotoxic mechanisms |
| Skin Sensitization Rule | 6 alerts | ■ 155 substructures<br>■ skin irritation |
| Aquatic Toxicity Rule | 2 alerts | ■ 99 substructures<br>■ toxicity to liquid(water) |
| NonBiodegradable Rule | 1 alerts | ■ 19 substructures<br>■ non-biodegradable |
| SureChEMBL Rule | 0 alerts | ■ 164 substructures<br>■ MedChem unfriendly status |

ADMETlab 2.0

**Predict ADMET Compound 9k**

ADMETlab 2.0

CNC(=O)c1sccc1N=c1[nH]c(=Nc2ccc(NC(=O)/C=C/c3cccc(F)c3)cc2)[nH]cc1C(F)(F)F

## 1. Physicochemical Property

| Property | Value | Comment |
|---|---|---|
| Molecular Weight | 556.13 | Contain hydrogen atoms. Optimal:100~600 |
| Volume | 513.456 | Van der Waals volume |
| Density | 1.083 | Density = MW / Volume |
| nHA | 8 | Number of hydrogen bond acceptors. Optimal:0~12 |
| nHD | 4 | Number of hydrogen bond donors. Optimal:0~7 |
| nRot | 9 | Number of rotatable bonds. Optimal:0~11 |
| nRing | 4 | Number of rings. Optimal:0~6 |
| MaxRing | 6 | Number of atoms in the biggest ring. Optimal:0~18 |
| nHet | 13 | Number of heteroatoms. Optimal:1~15 |
| fChar | 0 | Formal charge. Optimal:-4 ~4 |
| nRig | 28 | Number of rigid bonds. Optimal:0~30 |
| Flexibility | 0.321 | Flexibility = nRot /nRig |
| Stereo Centers | 0 | Optimal: ≤ 2 |
| TPSA | 114.5 | Topological Polar Surface Area. Optimal:0~140 |
| logS | -5.14 | Log of the aqueous solubility. Optimal: -4~0.5 log mol/L |
| logP | 4.432 | Log of the octanol/water partition coefficient. Optimal: 0~3 |
| logD | 3.766 | logP at physiological pH 7.4. Optimal: 1~3 |

## 2. Medicinal Chemistry

| Property | Value | Decision | Comment |
|---|---|---|---|
| QED | 0.197 | ● | ■ A measure of drug-likeness based on the concept of desirability; ■ Attractive: > 0.67; unattractive: 0.49~0.67; too complex: < 0.34 |
| SAscore | 3.464 | ● | ■ Synthetic accessibility score is designed to estimate ease of synthesis of drug-like molecules. ■ SAscore ≥ 6, difficult to synthesize; SAscore <6, easy to synthesize |
| Fsp3 | 0.077 | ● | ■ The number of sp3 hybridized carbons / total carbon count, correlating with melting point and solubility. ■ Fsp$^3$ ≥0.42 is considered a suitable value. |
| MCE-18 | 26.0 | ● | ■ MCE-18 stands for medicinal chemistry evolution. ■ MCE-18≥45 is considered a suitable value. |

ADMETlab 2.0

| | | | |
|---|---|---|---|
| NPscore | -1.197 | - | ■ Natural product-likeness score.<br>■ This score is typically in the range from –5 to 5. The higher the score is, the higher the probability is that the molecule is a NP. |
| Lipinski Rule | Accepted | ● (green) | ■ MW ≤ 500; logP ≤ 5; Hacc ≤ 10; Hdon ≤ 5<br>■ If two properties are out of range, a poor absorption or permeability is possible, one is acceptable. |
| Pfizer Rule | Accepted | ● (green) | logP > 3; TPSA < 75<br>Compounds with a high log P (>3) and low TPSA (<75) are likely to be toxic. |
| GSK Rule | Rejected | ● (red) | ■ MW ≤ 400; logP ≤ 4<br>■ Compounds satisfying the GSK rule may have a more favorable ADMET profile |
| Golden Triangle | Rejected | ● (red) | ■ 200 ≤ MW ≤ 50; -2 ≤ logD ≤ 5<br>■ Compounds satisfying the Golden Triangle rule may have a more favorable ADMET profile. |
| PAINS | 0 alerts | - | Pan Assay Interference Compounds, frequent hitters, Alpha-screen artifacts and reactive compound. |
| ALARM NMR | 4 alerts | - | Thiol reactive compounds. |
| BMS | 0 alerts | - | Undesirable, reactive compounds. |
| Chelator Rule | 0 alerts | - | Chelating compounds. |

## 3. Absorption

| Property | Value | Decision | Comment |
|---|---|---|---|
| Caco-2 Permeability | -5.568 | ● (red) | Optimal: higher than -5.15 Log unit |
| MDCK Permeability | 2.4e-05 | ● (green) | ■ low permeability: $< 2 \times 10^{-6}$ cm/s<br>■ medium permeability: $2–20 \times 10^{-6}$ cm/s<br>■ high passive permeability: $> 20 \times 10^{-6}$ cm/s |
| Pgp-inhibitor | 0.977 | ● (red) | ■ Category 1: Inhibitor; Category 0: Non-inhibitor;<br>■ The output value is the probability of being Pgp-inhibitor |
| Pgp-substrate | 0.009 | ● (green) | ■ Category 1: substrate; Category 0: Non-substrate;<br>■ The output value is the probability of being Pgp-substrate |
| HIA | 0.737 | ● (red) | ■ Human Intestinal Absorption<br>■ Category 1: HIA+( HIA < 30%); Category 0: HIA-( HIA < 30%); The output value is the probability of being HIA+ |
| $F_{20\%}$ | 0.001 | ● (green) | ■ 20% Bioavailability<br>■ Category 1: $F_{20\%}$+ (bioavailability < 20%); Category 0: $F_{20\%}$- (bioavailability ≥ 20%); The output value is the probability of being $F_{20\%}$+ |

ADMETlab 2.0

| | | | |
|---|---|---|---|
| $F_{30\%}$ | 0.002 | 🟢 | ■ 30% Bioavailability<br>■ Category 1: $F_{30\%}$+ (bioavailability < 30%); Category 0: $F_{30\%}$- (bioavailability ≥ 30%); The output value is the probability of being $F_{30\%}$+ |

## 4. Distribution

| Property | Value | Decision | Comment |
|---|---|---|---|
| PPB | 98.92% | 🔴 | ■ Plasma Protein Binding<br>■ Optimal: < 90%. Drugs with high protein-bound may have a low therapeutic index. |
| VD | 0.584 | 🟢 | ■ Volume Distribution<br>■ Optimal: 0.04-20L/kg |
| BBB Penetration | 0.061 | 🟢 | ■ Blood-Brain Barrier Penetration<br>■ Category 1: BBB+; Category 0: BBB-; The output value is the probability of being BBB+ |
| Fu | 0.950% | 🔴 | ■ The fraction unbound in plasms<br>■ Low: <5%; Middle: 5~20%; High: > 20% |

## 5. Metabolism

| Property | Value | Comment |
|---|---|---|
| CYP1A2 inhibitor | 0.595 | ■ Category 1: Inhibitor; Category 0: Non-inhibitor;<br>■ The output value is the probability of being inhibitor. |
| CYP1A2 substrate | 0.899 | ■ Category 1: Substrate; Category 0: Non-substrate;<br>■ The output value is the probability of being substrate. |
| CYP2C19 inhibitor | 0.957 | ■ Category 1: Inhibitor; Category 0: Non-inhibitor;<br>■ The output value is the probability of being inhibitor. |
| CYP2C19 substrate | 0.051 | ■ Category 1: Substrate; Category 0: Non-substrate;<br>■ The output value is the probability of being substrate. |
| CYP2C9 inhibitor | 0.931 | ■ Category 1: Inhibitor; Category 0: Non-inhibitor;<br>■ The output value is the probability of being inhibitor. |
| CYP2C9 substrate | 0.086 | ■ Category 1: Substrate; Category 0: Non-substrate;<br>■ The output value is the probability of being substrate. |
| CYP2D6 inhibitor | 0.032 | ■ Category 1: Inhibitor; Category 0: Non-inhibitor;<br>■ The output value is the probability of being inhibitor. |
| CYP2D6 substrate | 0.016 | ■ Category 1: Substrate; Category 0: Non-substrate;<br>■ The output value is the probability of being substrate. |
| CYP3A4 inhibitor | 0.908 | ■ Category 1: Inhibitor; Category 0: Non-inhibitor;<br>■ The output value is the probability of being inhibitor. |
| CYP3A4 substrate | 0.705 | ■ Category 1: Substrate; Category 0: Non-substrate;<br>■ The output value is the probability of being substrate. |

## 6. Excretion

| Property | Value | Decision | Comment |
|---|---|---|---|
| CL | 4.251 | ● | ■ Clearance<br>■ High: >15 mL/min/kg; moderate: 5-15 mL/min/kg; low: <5 mL/min/kg |
| $T_{1/2}$ | 0.083 | - | ■ Category 1: long half-life ; Category 0: short half-life;<br>■ long half-life: >3h; short half-life: <3h<br>■ The output value is the probability of having long half-life. |

## 7. Toxicity

| Property | Value | Decision | Comment |
|---|---|---|---|
| hERG Blockers | 0.942 | ● | ■ Category 1: active; Category 0: inactive;<br>■ The output value is the probability of being active. |
| H-HT | 0.997 | ● | ■ Human Hepatotoxicity<br>■ Category 1: H-HT positive(+); Category 0: H-HT negative(-);<br>■ The output value is the probability of being toxic. |
| DILI | 0.982 | ● | ■ Drug Induced Liver Injury.<br>■ Category 1: drugs with a high risk of DILI; Category 0: drugs with no risk of DILI. The output value is the probability of being toxic. |
| AMES Toxicity | 0.938 | ● | ■ Category 1: Ames positive(+); Category 0: Ames negative(-);<br>■ The output value is the probability of being toxic. |
| Rat Oral Acute Toxicity | 0.971 | ● | ■ Category 0: low-toxicity; Category 1: high-toxicity;<br>■ The output value is the probability of being highly toxic. |
| FDAMDD | 0.96 | ● | ■ Maximum Recommended Daily Dose<br>■ Category 1: FDAMDD (+); Category 0: FDAMDD (-)<br>■ The output value is the probability of being positive. |
| Skin Sensiti zation | 0.156 | ● | ■ Category 1: Sensitizer; Category 0: Non-sensitizer;<br>■ The output value is the probability of being sensitizer. |
| Carcinogen city | 0.27 | ● | ■ Category 1: carcinogens; Category 0: non-carcinogens;<br>■ The output value is the probability of being toxic. |
| Eye Corrosion | 0.003 | ● | ■ Category 1: corrosives ; Category 0: noncorrosives<br>■ The output value is the probability of being corrosives. |
| Eye Irritation | 0.009 | ● | ■ Category 1: irritants ; Category 0: nonirritants<br>■ The output value is the probability of being irritants. |

ADMETlab 2.0

| | | | |
|---|---|---|---|
| Respiratory Toxicity | 0.946 | ● | ■ Category 1: respiratory toxicants; Category 0: respiratory nontoxicants<br>■ The output value is the probability of being toxic. |

## 8. Environmental toxicity

| Property | Value | Comment |
|---|---|---|
| Bioconcentration Factors | 1.292 | ■ Bioconcentration factors are used for considering secondary poisoning potential and assessing risks to human health via the food chain.<br>■ The unit is –log10[(mg/L)/(1000*MW)] |
| $IGC_{50}$ | 4.413 | ■ Tetrahymena pyriformis 50 percent growth inhibition concentration<br>■ The unit is –log10[(mg/L)/(1000*MW)] |
| $LC_{50}FM$ | 5.958 | ■ 96-hour fathead minnow 50 percent lethal concentration<br>■ The unit is –log10[(mg/L)/(1000*MW)] |
| $LC_{50}DM$ | 6.198 | ■ 48-hour daphnia magna 50 percent lethal concentration<br>■ The unit is –log10[(mg/L)/(1000*MW)] |

## 9. Tox21 pathway

| Property | Value | Decision | Comment |
|---|---|---|---|
| NR-AR | 0.009 | ● | ■ Androgen receptor<br>■ Category 1: actives ; Category 0: inactives;<br>■ The output value is the probability of being active. |
| NR-AR-LBD | 0.646 | ● | ■ Androgen receptor ligand-binding domain<br>■ Category 1: actives ; Category 0: inactives;<br>■ The output value is the probability of being active. |
| NR-AhR | 0.984 | ● | ■ Aryl hydrocarbon receptor<br>■ Category 1: actives ; Category 0: inactives;<br>■ The output value is the probability of being active. |
| NR-Aromatase | 0.948 | ● | ■ Category 1: actives ; Category 0: inactives;<br>■ The output value is the probability of being active. |
| NR-ER | 0.79 | ● | ■ Estrogen receptor<br>■ Category 1: actives ; Category 0: inactives;<br>■ The output value is the probability of being active. |
| NR-ER-LBD | 0.008 | ● | ■ Estrogen receptor ligand-binding domain<br>■ Category 1: actives ; Category 0: inactives;<br>■ The output value is the probability of being active. |
| NR-PPAR-gamma | 0.813 | ● | ■ Peroxisome proliferator-activated receptor gamma<br>■ Category 1: actives ; Category 0: inactives;<br>■ The output value is the probability of being active. |
| SR-ARE | 0.919 | ● | ■ Antioxidant response element<br>■ Category 1: actives ; Category 0: inactives;<br>■ The output value is the probability of being active. |
| SR-ATAD5 | 0.872 | ● | ■ ATPase family AAA domain-containing protein 5<br>■ Category 1: actives ; Category 0: inactives;<br>■ The output value is the probability of being active. |

| | | | |
|---|---|---|---|
| SR-HSE | 0.109 | ● | ■ Heat shock factor response element<br>■ Category 1: actives ; Category 0: inactives;<br>■ The output value is the probability of being active. |
| SR-MMP | 0.952 | ● | ■ Mitochondrial membrane potential<br>■ Category 1: actives ; Category 0: inactives;<br>■ The output value is the probability of being active. |
| SR-p53 | 0.977 | ● | ■ Category 1: actives ; Category 0: inactives;<br>■ The output value is the probability of being active. |

## 10. Toxicophore Rules

| Property | Value | Comment |
|---|---|---|
| Acute Toxicity Rule | 0 alerts | ■ 20 substructures<br>■ acute toxicity during oral administration |
| Genotoxic Carcinogenicity Rule | 1 alerts | ■ 117 substructures<br>■ carcinogenicity or mutagenicity |
| NonGenotoxic Carcinogenicity Rule | 2 alerts | ■ 23 substructures<br>■ carcinogenicity through nongenotoxic mechanisms |
| Skin Sensitization Rule | 6 alerts | ■ 155 substructures<br>■ skin irritation |
| Aquatic Toxicity Rule | 6 alerts | ■ 99 substructures<br>■ toxicity to liquid(water) |
| NonBiodegradable Rule | 2 alerts | ■ 19 substructures<br>■ non-biodegradable |
| SureChEMBL Rule | 0 alerts | ■ 164 substructures<br>■ MedChem unfriendly status |

ADMETlab 2.0

Predict ADMET Compound 9t

ADMETlab 2.0

C#CC(=O)Nc1ccc(N=c2[nH]cc(C(F)(F)F)c(=Nc3ccsc3C(=O)NC)[nH]2)cc1

## 1. Physicochemical Property

| Property | Value | Comment |
|---|---|---|
| Molecular Weight | 460.09 | Contain hydrogen atoms. Optimal:100~600 |
| Volume | 417.442 | Van der Waals volume |
| Density | 1.102 | Density = MW / Volume |
| nHA | 8 | Number of hydrogen bond acceptors. Optimal:0~12 |
| nHD | 4 | Number of hydrogen bond donors. Optimal:0~7 |
| nRot | 7 | Number of rotatable bonds. Optimal:0~11 |
| nRing | 3 | Number of rings. Optimal:0~6 |
| MaxRing | 6 | Number of atoms in the biggest ring. Optimal:0~18 |
| nHet | 12 | Number of heteroatoms. Optimal:1~15 |
| fChar | 0 | Formal charge. Optimal:-4 ~4 |
| nRig | 22 | Number of rigid bonds. Optimal:0~30 |
| Flexibility | 0.318 | Flexibility = nRot /nRig |
| Stereo Centers | 0 | Optimal: ≤ 2 |
| TPSA | 114.5 | Topological Polar Surface Area. Optimal:0~140 |
| logS | -4.209 | Log of the aqueous solubility. Optimal: -4~0.5 log mol/L |
| logP | 2.995 | Log of the octanol/water partition coefficient. Optimal: 0~3 |
| logD | 3.229 | logP at physiological pH 7.4. Optimal: 1~3 |

## 2. Medicinal Chemistry

| Property | Value | Decision | Comment |
|---|---|---|---|
| QED | 0.449 | ● | ■ A measure of drug-likeness based on the concept of desirability; ■ Attractive: > 0.67; unattractive: 0.49~0.67; too complex: < 0.34 |
| SAscore | 3.717 | ● | ■ Synthetic accessibility score is designed to estimate ease of synthesis of drug-like molecules. ■ SAscore ≥ 6, difficult to synthesize; SAscore <6, easy to synthesize |
| Fsp3 | 0.1 | ● | ■ The number of sp3 hybridized carbons / total carbon count, correlating with melting point and solubility. ■ $Fsp^3$ ≥0.42 is considered a suitable value. |
| MCE-18 | 21.0 | ● | ■ MCE-18 stands for medicinal chemistry evolution. ■ MCE-18≥45 is considered a suitable value. |

ADMETlab 2.0

| | | | |
|---|---|---|---|
| NPscore | -1.019 | - | ■ Natural product-likeness score.<br>■ This score is typically in the range from –5 to 5. The higher the score is, the higher the probability is that the molecule is a NP. |
| Lipinski Rule | Accepted | 🟢 | ■ MW ≤ 500; logP ≤ 5; Hacc ≤ 10; Hdon ≤ 5<br>■ If two properties are out of range, a poor absorption or permeability is possible, one is acceptable. |
| Pfizer Rule | Accepted | 🟢 | logP > 3; TPSA < 75<br>Compounds with a high log P (>3) and low TPSA (<75) are likely to be toxic. |
| GSK Rule | Rejected | 🔴 | ■ MW ≤ 400; logP ≤ 4<br>■ Compounds satisfying the GSK rule may have a more favorable ADMET profile |
| Golden Triangle | Accepted | 🟢 | ■ 200 ≤ MW ≤ 50; -2 ≤ logD ≤ 5<br>■ Compounds satisfying the Golden Triangle rule may have a more favorable ADMET profile. |
| PAINS | 0 alerts | - | Pan Assay Interference Compounds, frequent hitters, Alpha-screen artifacts and reactive compound. |
| ALARM NMR | 3 alerts | - | Thiol reactive compounds. |
| BMS | 1 alerts | - | Undesirable, reactive compounds. |
| Chelator Rule | 0 alerts | - | Chelating compounds. |

## 3. Absorption

| Property | Value | Decision | Comment |
|---|---|---|---|
| Caco-2 Permeability | -5.104 | 🟢 | Optimal: higher than -5.15 Log unit |
| MDCK Permeability | 2.3e-05 | 🟢 | ■ low permeability: < 2 × $10^{-6}$ cm/s<br>■ medium permeability: 2–20 × $10^{-6}$ cm/s<br>■ high passive permeability: > 20 × $10^{-6}$ cm/s |
| Pgp-inhibitor | 0.001 | 🟢 | ■ Category 1: Inhibitor; Category 0: Non-inhibitor;<br>■ The output value is the probability of being Pgp-inhibitor |
| Pgp-substrate | 0.003 | 🟢 | ■ Category 1: substrate; Category 0: Non-substrate;<br>■ The output value is the probability of being Pgp-substrate |
| HIA | 0.767 | 🔴 | ■ Human Intestinal Absorption<br>■ Category 1: HIA+( HIA < 30%); Category 0: HIA-( HIA < 30%); The output value is the probability of being HIA+ |
| $F_{20\%}$ | 0.093 | 🟢 | ■ 20% Bioavailability<br>■ Category 1: $F_{20\%}$+ (bioavailability < 20%); Category 0: $F_{20\%}$- (bioavailability ≥ 20%); The output value is the probability of being $F_{20\%}$+ |

ADMETlab 2.0

| | | | |
|---|---|---|---|
| $F_{30\%}$ | 0.015 | ● | ■ 30% Bioavailability<br>■ Category 1: $F_{30\%}$+ (bioavailability < 30%); Category 0: $F_{30\%}$- (bioavailability ≥ 30%); The output value is the probability of being $F_{30\%}$+ |

## 4. Distribution

| Property | Value | Decision | Comment |
|---|---|---|---|
| PPB | 93.98% | ● | ■ Plasma Protein Binding<br>■ Optimal: < 90%. Drugs with high protein-bound may have a low therapeutic index. |
| VD | 0.844 | ● | ■ Volume Distribution<br>■ Optimal: 0.04-20L/kg |
| BBB Penetration | 0.704 | ● | ■ Blood-Brain Barrier Penetration<br>■ Category 1: BBB+; Category 0: BBB-; The output value is the probability of being BBB+ |
| Fu | 2.402% | ● | ■ The fraction unbound in plasms<br>■ Low: <5%; Middle: 5~20%; High: > 20% |

## 5. Metabolism

| Property | Value | Comment |
|---|---|---|
| CYP1A2 inhibitor | 0.534 | ■ Category 1: Inhibitor; Category 0: Non-inhibitor;<br>■ The output value is the probability of being inhibitor. |
| CYP1A2 substrate | 0.938 | ■ Category 1: Substrate; Category 0: Non-substrate;<br>■ The output value is the probability of being substrate. |
| CYP2C19 inhibitor | 0.938 | ■ Category 1: Inhibitor; Category 0: Non-inhibitor;<br>■ The output value is the probability of being inhibitor. |
| CYP2C19 substrate | 0.054 | ■ Category 1: Substrate; Category 0: Non-substrate;<br>■ The output value is the probability of being substrate. |
| CYP2C9 inhibitor | 0.819 | ■ Category 1: Inhibitor; Category 0: Non-inhibitor;<br>■ The output value is the probability of being inhibitor. |
| CYP2C9 substrate | 0.053 | ■ Category 1: Substrate; Category 0: Non-substrate;<br>■ The output value is the probability of being substrate. |
| CYP2D6 inhibitor | 0.023 | ■ Category 1: Inhibitor; Category 0: Non-inhibitor;<br>■ The output value is the probability of being inhibitor. |
| CYP2D6 substrate | 0.011 | ■ Category 1: Substrate; Category 0: Non-substrate;<br>■ The output value is the probability of being substrate. |
| CYP3A4 inhibitor | 0.864 | ■ Category 1: Inhibitor; Category 0: Non-inhibitor;<br>■ The output value is the probability of being inhibitor. |
| CYP3A4 substrate | 0.741 | ■ Category 1: Substrate; Category 0: Non-substrate;<br>■ The output value is the probability of being substrate. |

ADMETlab 2.0

## 6. Excretion

| Property | Value | Decision | Comment |
|---|---|---|---|
| CL | 5.943 | ● | ■ Clearance<br>■ High: >15 mL/min/kg; moderate: 5-15 mL/min/kg; low: <5 mL/min/kg |
| $T_{1/2}$ | 0.225 | - | ■ Category 1: long half-life ; Category 0: short half-life;<br>■ long half-life: >3h; short half-life: <3h<br>■ The output value is the probability of having long half-life. |

## 7. Toxicity

| Property | Value | Decision | Comment |
|---|---|---|---|
| hERG Blockers | 0.678 | ● | ■ Category 1: active; Category 0: inactive;<br>■ The output value is the probability of being active. |
| H-HT | 0.995 | ● | ■ Human Hepatotoxicity<br>■ Category 1: H-HT positive(+); Category 0: H-HT negative(-);<br>■ The output value is the probability of being toxic. |
| DILI | 0.987 | ● | ■ Drug Induced Liver Injury.<br>■ Category 1: drugs with a high risk of DILI; Category 0: drugs with no risk of DILI. The output value is the probability of being toxic. |
| AMES Toxicity | 0.887 | ● | ■ Category 1: Ames positive(+); Category 0: Ames negative(-);<br>■ The output value is the probability of being toxic. |
| Rat Oral Acute Toxicity | 0.993 | ● | ■ Category 0: low-toxicity; Category 1: high-toxicity;<br>■ The output value is the probability of being highly toxic. |
| FDAMDD | 0.886 | ● | ■ Maximum Recommended Daily Dose<br>■ Category 1: FDAMDD (+); Category 0: FDAMDD (-)<br>■ The output value is the probability of being positive. |
| Skin Sensiti zation | 0.364 | ● | ■ Category 1: Sensitizer; Category 0: Non-sensitizer;<br>■ The output value is the probability of being sensitizer. |
| Carcinogen city | 0.316 | ● | ■ Category 1: carcinogens; Category 0: non-carcinogens;<br>■ The output value is the probability of being toxic. |
| Eye Corrosion | 0.003 | ● | ■ Category 1: corrosives ; Category 0: noncorrosives<br>■ The output value is the probability of being corrosives. |
| Eye Irritation | 0.01 | ● | ■ Category 1: irritants ; Category 0: nonirritants<br>■ The output value is the probability of being irritants. |

ADMETlab 2.0

| | | | |
|---|---|---|---|
| Respiratory Toxicity | 0.982 | ● | ■ Category 1: respiratory toxicants; Category 0: respiratory nontoxicants<br>■ The output value is the probability of being toxic. |

## 8. Environmental toxicity

| Property | Value | Comment |
|---|---|---|
| Bioconcentration Factors | 0.584 | ■ Bioconcentration factors are used for considering secondary poisoning potential and assessing risks to human health via the food chain.<br>■ The unit is –log10[(mg/L)/(1000*MW)] |
| $IGC_{50}$ | 4.639 | ■ Tetrahymena pyriformis 50 percent growth inhibition concentration<br>■ The unit is –log10[(mg/L)/(1000*MW)] |
| $LC_{50}FM$ | 5.817 | ■ 96-hour fathead minnow 50 percent lethal concentration<br>■ The unit is –log10[(mg/L)/(1000*MW)] |
| $LC_{50}DM$ | 6.006 | ■ 48-hour daphnia magna 50 percent lethal concentration<br>■ The unit is –log10[(mg/L)/(1000*MW)] |

## 9. Tox21 pathway

| Property | Value | Decision | Comment |
|---|---|---|---|
| NR-AR | 0.018 | ● | ■ Androgen receptor<br>■ Category 1: actives ; Category 0: inactives;<br>■ The output value is the probability of being active. |
| NR-AR-LBD | 0.024 | ● | ■ Androgen receptor ligand-binding domain<br>■ Category 1: actives ; Category 0: inactives;<br>■ The output value is the probability of being active. |
| NR-AhR | 0.98 | ● | ■ Aryl hydrocarbon receptor<br>■ Category 1: actives ; Category 0: inactives;<br>■ The output value is the probability of being active. |
| NR-Aromatase | 0.91 | ● | ■ Category 1: actives ; Category 0: inactives;<br>■ The output value is the probability of being active. |
| NR-ER | 0.365 | ● | ■ Estrogen receptor<br>■ Category 1: actives ; Category 0: inactives;<br>■ The output value is the probability of being active. |
| NR-ER-LBD | 0.01 | ● | ■ Estrogen receptor ligand-binding domain<br>■ Category 1: actives ; Category 0: inactives;<br>■ The output value is the probability of being active. |
| NR-PPAR-gamma | 0.331 | ● | ■ Peroxisome proliferator-activated receptor gamma<br>■ Category 1: actives ; Category 0: inactives;<br>■ The output value is the probability of being active. |
| SR-ARE | 0.922 | ● | ■ Antioxidant response element<br>■ Category 1: actives ; Category 0: inactives;<br>■ The output value is the probability of being active. |
| SR-ATAD5 | 0.494 | ● | ■ ATPase family AAA domain-containing protein 5<br>■ Category 1: actives ; Category 0: inactives;<br>■ The output value is the probability of being active. |

ADMETlab 2.0

| | | | |
|---|---|---|---|
| SR-HSE | 0.101 | ● | ■ Heat shock factor response element<br>■ Category 1: actives ; Category 0: inactives;<br>■ The output value is the probability of being active. |
| SR-MMP | 0.958 | ● | ■ Mitochondrial membrane potential<br>■ Category 1: actives ; Category 0: inactives;<br>■ The output value is the probability of being active. |
| SR-p53 | 0.962 | ● | ■ Category 1: actives ; Category 0: inactives;<br>■ The output value is the probability of being active. |

## 10. Toxicophore Rules

| Property | Value | Comment |
|---|---|---|
| Acute Toxicity Rule | 0 alerts | ■ 20 substructures<br>■ acute toxicity during oral administration |
| Genotoxic Carcinogenicity Rule | 1 alerts | ■ 117 substructures<br>■ carcinogenicity or mutagenicity |
| NonGenotoxic Carcinogenicity Rule | 0 alerts | ■ 23 substructures<br>■ carcinogenicity through nongenotoxic mechanisms |
| Skin Sensitization Rule | 4 alerts | ■ 155 substructures<br>■ skin irritation |
| Aquatic Toxicity Rule | 3 alerts | ■ 99 substructures<br>■ toxicity to liquid(water) |
| NonBiodegradable Rule | 1 alerts | ■ 19 substructures<br>■ non-biodegradable |
| SureChEMBL Rule | 1 alerts | ■ 164 substructures<br>■ MedChem unfriendly status |

ADMETlab 2.0

**Predict ADMET Compound 9u**

**ADMETlab 2.0**

CNC(=O)c1sccc1N=c1[nH]c(=Nc2ccc(NC(=O)/C=C/c3cccc(F)c3)cc2)[nH]cc1C(F)(F)F

## 1. Physicochemical Property

| Property | Value | Comment |
|---|---|---|
| Molecular Weight | 556.13 | Contain hydrogen atoms. Optimal:100~600 |
| Volume | 513.456 | Van der Waals volume |
| Density | 1.083 | Density = MW / Volume |
| nHA | 8 | Number of hydrogen bond acceptors. Optimal:0~12 |
| nHD | 4 | Number of hydrogen bond donors. Optimal:0~7 |
| nRot | 9 | Number of rotatable bonds. Optimal:0~11 |
| nRing | 4 | Number of rings. Optimal:0~6 |
| MaxRing | 6 | Number of atoms in the biggest ring. Optimal:0~18 |
| nHet | 13 | Number of heteroatoms. Optimal:1~15 |
| fChar | 0 | Formal charge. Optimal:-4 ~4 |
| nRig | 28 | Number of rigid bonds. Optimal:0~30 |
| Flexibility | 0.321 | Flexibility = nRot /nRig |
| Stereo Centers | 0 | Optimal: ≤ 2 |
| TPSA | 114.5 | Topological Polar Surface Area. Optimal:0~140 |
| logS | -5.14 | Log of the aqueous solubility. Optimal: -4~0.5 log mol/L |
| logP | 4.432 | Log of the octanol/water partition coefficient. Optimal: 0~3 |
| logD | 3.766 | logP at physiological pH 7.4. Optimal: 1~3 |

## 2. Medicinal Chemistry

| Property | Value | Decision | Comment |
|---|---|---|---|
| QED | 0.197 | ● | ■ A measure of drug-likeness based on the concept of desirability; ■ Attractive: > 0.67; unattractive: 0.49~0.67; too complex: < 0.34 |
| SAscore | 3.464 | ● | ■ Synthetic accessibility score is designed to estimate ease of synthesis of drug-like molecules. ■ SAscore ≥ 6, difficult to synthesize; SAscore <6, easy to synthesize |
| Fsp3 | 0.077 | ● | ■ The number of sp3 hybridized carbons / total carbon count, correlating with melting point and solubility. ■ $Fsp^3$ ≥0.42 is considered a suitable value. |
| MCE-18 | 26.0 | ● | ■ MCE-18 stands for medicinal chemistry evolution. ■ MCE-18≥45 is considered a suitable value. |

| | | | |
|---|---|---|---|
| NPscore | -1.197 | - | ■ Natural product-likeness score.<br>■ This score is typically in the range from –5 to 5. The higher the score is, the higher the probability is that the molecule is a NP. |
| Lipinski Rule | Accepted | 🟢 | ■ MW ≤ 500; logP ≤ 5; Hacc ≤ 10; Hdon ≤ 5<br>■ If two properties are out of range, a poor absorption or permeability is possible, one is acceptable. |
| Pfizer Rule | Accepted | 🟢 | logP > 3; TPSA < 75<br>Compounds with a high log P (>3) and low TPSA (<75) are likely to be toxic. |
| GSK Rule | Rejected | 🔴 | ■ MW ≤ 400; logP ≤ 4<br>■ Compounds satisfying the GSK rule may have a more favorable ADMET profile |
| Golden Triangle | Rejected | 🔴 | ■ 200 ≤ MW ≤ 50; -2 ≤ logD ≤ 5<br>■ Compounds satisfying the Golden Triangle rule may have a more favorable ADMET profile. |
| PAINS | 0 alerts | - | Pan Assay Interference Compounds, frequent hitters, Alpha-screen artifacts and reactive compound. |
| ALARM NMR | 4 alerts | - | Thiol reactive compounds. |
| BMS | 0 alerts | - | Undesirable, reactive compounds. |
| Chelator Rule | 0 alerts | - | Chelating compounds. |

## 3. Absorption

| Property | Value | Decision | Comment |
|---|---|---|---|
| Caco-2 Permeability | -5.568 | 🔴 | Optimal: higher than -5.15 Log unit |
| MDCK Permeability | 2.4e-05 | 🟢 | ■ low permeability: < 2 × $10^{-6}$ cm/s<br>■ medium permeability: 2–20 × $10^{-6}$ cm/s<br>■ high passive permeability: > 20 × $10^{-6}$ cm/s |
| Pgp-inhibitor | 0.977 | 🔴 | ■ Category 1: Inhibitor; Category 0: Non-inhibitor;<br>■ The output value is the probability of being Pgp-inhibitor |
| Pgp-substrate | 0.009 | 🟢 | ■ Category 1: substrate; Category 0: Non-substrate;<br>■ The output value is the probability of being Pgp-substrate |
| HIA | 0.737 | 🔴 | ■ Human Intestinal Absorption<br>■ Category 1: HIA+( HIA < 30%); Category 0: HIA-( HIA < 30%); The output value is the probability of being HIA+ |
| $F_{20\%}$ | 0.001 | 🟢 | ■ 20% Bioavailability<br>■ Category 1: $F_{20\%}$+ (bioavailability < 20%); Category 0: $F_{20\%}$- (bioavailability ≥ 20%); The output value is the probability of being $F_{20\%}$+ |

ADMETlab 2.0

| | | | |
|---|---|---|---|
| $F_{30\%}$ | 0.002 | ● | ■ 30% Bioavailability<br>■ Category 1: $F_{30\%}$+ (bioavailability < 30%); Category 0: $F_{30\%}$- (bioavailability ≥ 30%); The output value is the probability of being $F_{30\%}$+ |

## 4. Distribution

| Property | Value | Decision | Comment |
|---|---|---|---|
| PPB | 98.92% | ● | ■ Plasma Protein Binding<br>■ Optimal: < 90%. Drugs with high protein-bound may have a low therapeutic index. |
| VD | 0.584 | ● | ■ Volume Distribution<br>■ Optimal: 0.04-20L/kg |
| BBB Penetration | 0.061 | ● | ■ Blood-Brain Barrier Penetration<br>■ Category 1: BBB+; Category 0: BBB-; The output value is the probability of being BBB+ |
| Fu | 0.950% | ● | ■ The fraction unbound in plasms<br>■ Low: <5%; Middle: 5~20%; High: > 20% |

## 5. Metabolism

| Property | Value | Comment |
|---|---|---|
| CYP1A2 inhibitor | 0.595 | ■ Category 1: Inhibitor; Category 0: Non-inhibitor;<br>■ The output value is the probability of being inhibitor. |
| CYP1A2 substrate | 0.899 | ■ Category 1: Substrate; Category 0: Non-substrate;<br>■ The output value is the probability of being substrate. |
| CYP2C19 inhibitor | 0.957 | ■ Category 1: Inhibitor; Category 0: Non-inhibitor;<br>■ The output value is the probability of being inhibitor. |
| CYP2C19 substrate | 0.051 | ■ Category 1: Substrate; Category 0: Non-substrate;<br>■ The output value is the probability of being substrate. |
| CYP2C9 inhibitor | 0.931 | ■ Category 1: Inhibitor; Category 0: Non-inhibitor;<br>■ The output value is the probability of being inhibitor. |
| CYP2C9 substrate | 0.086 | ■ Category 1: Substrate; Category 0: Non-substrate;<br>■ The output value is the probability of being substrate. |
| CYP2D6 inhibitor | 0.032 | ■ Category 1: Inhibitor; Category 0: Non-inhibitor;<br>■ The output value is the probability of being inhibitor. |
| CYP2D6 substrate | 0.016 | ■ Category 1: Substrate; Category 0: Non-substrate;<br>■ The output value is the probability of being substrate. |
| CYP3A4 inhibitor | 0.908 | ■ Category 1: Inhibitor; Category 0: Non-inhibitor;<br>■ The output value is the probability of being inhibitor. |
| CYP3A4 substrate | 0.705 | ■ Category 1: Substrate; Category 0: Non-substrate;<br>■ The output value is the probability of being substrate. |

## 6. Excretion

| Property | Value | Decision | Comment |
|---|---|---|---|
| CL | 4.251 | ● (red) | ■ Clearance<br>■ High: >15 mL/min/kg; moderate: 5-15 mL/min/kg; low: <5 mL/min/kg |
| $T_{1/2}$ | 0.083 | - | ■ Category 1: long half-life ; Category 0: short half-life;<br>■ long half-life: >3h; short half-life: <3h<br>■ The output value is the probability of having long half-life. |

## 7. Toxicity

| Property | Value | Decision | Comment |
|---|---|---|---|
| hERG Blockers | 0.942 | ● (red) | ■ Category 1: active; Category 0: inactive;<br>■ The output value is the probability of being active. |
| H-HT | 0.997 | ● (red) | ■ Human Hepatotoxicity<br>■ Category 1: H-HT positive(+); Category 0: H-HT negative(-);<br>■ The output value is the probability of being toxic. |
| DILI | 0.982 | ● (red) | ■ Drug Induced Liver Injury.<br>■ Category 1: drugs with a high risk of DILI; Category 0: drugs with no risk of DILI. The output value is the probability of being toxic. |
| AMES Toxicity | 0.938 | ● (red) | ■ Category 1: Ames positive(+); Category 0: Ames negative(-);<br>■ The output value is the probability of being toxic. |
| Rat Oral Acute Toxicity | 0.971 | ● (red) | ■ Category 0: low-toxicity; Category 1: high-toxicity;<br>■ The output value is the probability of being highly toxic. |
| FDAMDD | 0.96 | ● (red) | ■ Maximum Recommended Daily Dose<br>■ Category 1: FDAMDD (+); Category 0: FDAMDD (-)<br>■ The output value is the probability of being positive. |
| Skin Sensiti zation | 0.156 | ● (green) | ■ Category 1: Sensitizer; Category 0: Non-sensitizer;<br>■ The output value is the probability of being sensitizer. |
| Carcinogen city | 0.27 | ● (green) | ■ Category 1: carcinogens; Category 0: non-carcinogens;<br>■ The output value is the probability of being toxic. |
| Eye Corrosion | 0.003 | ● (green) | ■ Category 1: corrosives ; Category 0: noncorrosives<br>■ The output value is the probability of being corrosives. |
| Eye Irritation | 0.009 | ● (green) | ■ Category 1: irritants ; Category 0: nonirritants<br>■ The output value is the probability of being irritants. |

ADMETlab 2.0

| | | | |
|---|---|---|---|
| Respiratory Toxicity | 0.946 | 🔴 | ■ Category 1: respiratory toxicants; Category 0: respiratory nontoxicants<br>■ The output value is the probability of being toxic. |

## 8. Environmental toxicity

| Property | Value | Comment |
|---|---|---|
| Bioconcentration Factors | 1.292 | ■ Bioconcentration factors are used for considering secondary poisoning potential and assessing risks to human health via the food chain.<br>■ The unit is –log10[(mg/L)/(1000*MW)] |
| $IGC_{50}$ | 4.413 | ■ Tetrahymena pyriformis 50 percent growth inhibition concentration<br>■ The unit is –log10[(mg/L)/(1000*MW)] |
| $LC_{50}FM$ | 5.958 | ■ 96-hour fathead minnow 50 percent lethal concentration<br>■ The unit is –log10[(mg/L)/(1000*MW)] |
| $LC_{50}DM$ | 6.198 | ■ 48-hour daphnia magna 50 percent lethal concentration<br>■ The unit is –log10[(mg/L)/(1000*MW)] |

## 9. Tox21 pathway

| Property | Value | Decision | Comment |
|---|---|---|---|
| NR-AR | 0.009 | 🟢 | ■ Androgen receptor<br>■ Category 1: actives ; Category 0: inactives;<br>■ The output value is the probability of being active. |
| NR-AR-LBD | 0.646 | 🟠 | ■ Androgen receptor ligand-binding domain<br>■ Category 1: actives ; Category 0: inactives;<br>■ The output value is the probability of being active. |
| NR-AhR | 0.984 | 🔴 | ■ Aryl hydrocarbon receptor<br>■ Category 1: actives ; Category 0: inactives;<br>■ The output value is the probability of being active. |
| NR-Aromatase | 0.948 | 🔴 | ■ Category 1: actives ; Category 0: inactives;<br>■ The output value is the probability of being active. |
| NR-ER | 0.79 | 🔴 | ■ Estrogen receptor<br>■ Category 1: actives ; Category 0: inactives;<br>■ The output value is the probability of being active. |
| NR-ER-LBD | 0.008 | 🟢 | ■ Estrogen receptor ligand-binding domain<br>■ Category 1: actives ; Category 0: inactives;<br>■ The output value is the probability of being active. |
| NR-PPAR-gamma | 0.813 | 🔴 | ■ Peroxisome proliferator-activated receptor gamma<br>■ Category 1: actives ; Category 0: inactives;<br>■ The output value is the probability of being active. |
| SR-ARE | 0.919 | 🔴 | ■ Antioxidant response element<br>■ Category 1: actives ; Category 0: inactives;<br>■ The output value is the probability of being active. |
| SR-ATAD5 | 0.872 | 🔴 | ■ ATPase family AAA domain-containing protein 5<br>■ Category 1: actives ; Category 0: inactives;<br>■ The output value is the probability of being active. |

| | | | |
|---|---|---|---|
| SR-HSE | 0.109 | | ■ Heat shock factor response element<br>■ Category 1: actives ; Category 0: inactives;<br>■ The output value is the probability of being active. |
| SR-MMP | 0.952 | | ■ Mitochondrial membrane potential<br>■ Category 1: actives ; Category 0: inactives;<br>■ The output value is the probability of being active. |
| SR-p53 | 0.977 | | ■ Category 1: actives ; Category 0: inactives;<br>■ The output value is the probability of being active. |

## 10. Toxicophore Rules

| Property | Value | Comment |
|---|---|---|
| Acute Toxicity Rule | 0 alerts | ■ 20 substructures<br>■ acute toxicity during oral administration |
| Genotoxic Carcinogenicity Rule | 1 alerts | ■ 117 substructures<br>■ carcinogenicity or mutagenicity |
| NonGenotoxic Carcinogenicity Rule | 2 alerts | ■ 23 substructures<br>■ carcinogenicity through nongenotoxic mechanisms |
| Skin Sensitization Rule | 6 alerts | ■ 155 substructures<br>■ skin irritation |
| Aquatic Toxicity Rule | 6 alerts | ■ 99 substructures<br>■ toxicity to liquid(water) |
| NonBiodegradable Rule | 2 alerts | ■ 19 substructures<br>■ non-biodegradable |
| SureChEMBL Rule | 0 alerts | ■ 164 substructures<br>■ MedChem unfriendly status |

ADMETlab 2.0

**Predict ADMET Compound 9v**

ADMETlab 2.0

CNC(=O)c1sccc1N=c1[nH]c(=Nc2ccc(NC(=O)/C=C/c3ccc(F)cc3)cc2)[nH]cc1C(F)(F)F

## 1. Physicochemical Property

| Property | Value | Comment |
|---|---|---|
| Molecular Weight | 556.13 | Contain hydrogen atoms. Optimal:100~600 |
| Volume | 513.456 | Van der Waals volume |
| Density | 1.083 | Density = MW / Volume |
| nHA | 8 | Number of hydrogen bond acceptors. Optimal:0~12 |
| nHD | 4 | Number of hydrogen bond donors. Optimal:0~7 |
| nRot | 9 | Number of rotatable bonds. Optimal:0~11 |
| nRing | 4 | Number of rings. Optimal:0~6 |
| MaxRing | 6 | Number of atoms in the biggest ring. Optimal:0~18 |
| nHet | 13 | Number of heteroatoms. Optimal:1~15 |
| fChar | 0 | Formal charge. Optimal:-4 ~4 |
| nRig | 28 | Number of rigid bonds. Optimal:0~30 |
| Flexibility | 0.321 | Flexibility = nRot /nRig |
| Stereo Centers | 0 | Optimal: ≤ 2 |
| TPSA | 114.5 | Topological Polar Surface Area. Optimal:0~140 |
| logS | -5.076 | Log of the aqueous solubility. Optimal: -4~0.5 log mol/L |
| logP | 4.441 | Log of the octanol/water partition coefficient. Optimal: 0~3 |
| logD | 3.73 | logP at physiological pH 7.4. Optimal: 1~3 |

## 2. Medicinal Chemistry

| Property | Value | Decision | Comment |
|---|---|---|---|
| QED | 0.197 | ● | ■ A measure of drug-likeness based on the concept of desirability; ■ Attractive: > 0.67; unattractive: 0.49~0.67; too complex: < 0.34 |
| SAscore | 3.423 | ● | ■ Synthetic accessibility score is designed to estimate ease of synthesis of drug-like molecules. ■ SAscore ≥ 6, difficult to synthesize; SAscore <6, easy to synthesize |
| Fsp3 | 0.077 | ● | ■ The number of sp3 hybridized carbons / total carbon count, correlating with melting point and solubility. ■ $Fsp^3$ ≥0.42 is considered a suitable value. |
| MCE-18 | 26.0 | ● | ■ MCE-18 stands for medicinal chemistry evolution. ■ MCE-18≥45 is considered a suitable value. |

ADMETlab 2.0

| | | | |
|---|---|---|---|
| NPscore | -1.122 | - | ■ Natural product-likeness score.<br>■ This score is typically in the range from –5 to 5. The higher the score is, the higher the probability is that the molecule is a NP. |
| Lipinski Rule | Accepted | ● | ■ MW ≤ 500; logP ≤ 5; Hacc ≤ 10; Hdon ≤ 5<br>■ If two properties are out of range, a poor absorption or permeability is possible, one is acceptable. |
| Pfizer Rule | Accepted | ● | logP > 3; TPSA < 75<br>Compounds with a high log P (>3) and low TPSA (<75) are likely to be toxic. |
| GSK Rule | Rejected | ● | ■ MW ≤ 400; logP ≤ 4<br>■ Compounds satisfying the GSK rule may have a more favorable ADMET profile |
| Golden Triangle | Rejected | ● | ■ 200 ≤ MW ≤ 50; -2 ≤ logD ≤ 5<br>■ Compounds satisfying the Golden Triangle rule may have a more favorable ADMET profile. |
| PAINS | 0 alerts | - | Pan Assay Interference Compounds, frequent hitters, Alpha-screen artifacts and reactive compound. |
| ALARM NMR | 4 alerts | - | Thiol reactive compounds. |
| BMS | 0 alerts | - | Undesirable, reactive compounds. |
| Chelator Rule | 0 alerts | - | Chelating compounds. |

## 3. Absorption

| Property | Value | Decision | Comment |
|---|---|---|---|
| Caco-2 Permeability | -5.494 | ● | Optimal: higher than -5.15 Log unit |
| MDCK Permeability | 2.4e-05 | ● | ■ low permeability: < 2 × $10^{-6}$ cm/s<br>■ medium permeability: 2–20 × $10^{-6}$ cm/s<br>■ high passive permeability: > 20 × $10^{-6}$ cm/s |
| Pgp-inhibitor | 0.979 | ● | ■ Category 1: Inhibitor; Category 0: Non-inhibitor;<br>■ The output value is the probability of being Pgp-inhibitor |
| Pgp-substrate | 0.011 | ● | ■ Category 1: substrate; Category 0: Non-substrate;<br>■ The output value is the probability of being Pgp-substrate |
| HIA | 0.708 | ● | ■ Human Intestinal Absorption<br>■ Category 1: HIA+( HIA < 30%); Category 0: HIA-( HIA < 30%); The output value is the probability of being HIA+ |
| $F_{20\%}$ | 0.001 | ● | ■ 20% Bioavailability<br>■ Category 1: $F_{20\%}$+ (bioavailability < 20%); Category 0: $F_{20\%}$- (bioavailability ≥ 20%); The output value is the probability of being $F_{20\%}$+ |

ADMETlab 2.0

| | | | |
|---|---|---|---|
| $F_{30\%}$ | 0.002 | ● | ■ 30% Bioavailability<br>■ Category 1: $F_{30\%}$+ (bioavailability < 30%); Category 0: $F_{30\%}$- (bioavailability ≥ 30%); The output value is the probability of being $F_{30\%}$+ |

## 4. Distribution

| Property | Value | Decision | Comment |
|---|---|---|---|
| PPB | 98.83% | ● | ■ Plasma Protein Binding<br>■ Optimal: < 90%. Drugs with high protein-bound may have a low therapeutic index. |
| VD | 0.606 | ● | ■ Volume Distribution<br>■ Optimal: 0.04-20L/kg |
| BBB Penetration | 0.047 | ● | ■ Blood-Brain Barrier Penetration<br>■ Category 1: BBB+; Category 0: BBB-; The output value is the probability of being BBB+ |
| Fu | 0.972% | ● | ■ The fraction unbound in plasms<br>■ Low: <5%; Middle: 5~20%; High: > 20% |

## 5. Metabolism

| Property | Value | Comment |
|---|---|---|
| CYP1A2 inhibitor | 0.581 | ■ Category 1: Inhibitor; Category 0: Non-inhibitor;<br>■ The output value is the probability of being inhibitor. |
| CYP1A2 substrate | 0.89 | ■ Category 1: Substrate; Category 0: Non-substrate;<br>■ The output value is the probability of being substrate. |
| CYP2C19 inhibitor | 0.954 | ■ Category 1: Inhibitor; Category 0: Non-inhibitor;<br>■ The output value is the probability of being inhibitor. |
| CYP2C19 substrate | 0.051 | ■ Category 1: Substrate; Category 0: Non-substrate;<br>■ The output value is the probability of being substrate. |
| CYP2C9 inhibitor | 0.917 | ■ Category 1: Inhibitor; Category 0: Non-inhibitor;<br>■ The output value is the probability of being inhibitor. |
| CYP2C9 substrate | 0.095 | ■ Category 1: Substrate; Category 0: Non-substrate;<br>■ The output value is the probability of being substrate. |
| CYP2D6 inhibitor | 0.03 | ■ Category 1: Inhibitor; Category 0: Non-inhibitor;<br>■ The output value is the probability of being inhibitor. |
| CYP2D6 substrate | 0.017 | ■ Category 1: Substrate; Category 0: Non-substrate;<br>■ The output value is the probability of being substrate. |
| CYP3A4 inhibitor | 0.892 | ■ Category 1: Inhibitor; Category 0: Non-inhibitor;<br>■ The output value is the probability of being inhibitor. |
| CYP3A4 substrate | 0.737 | ■ Category 1: Substrate; Category 0: Non-substrate;<br>■ The output value is the probability of being substrate. |

## 6. Excretion

| Property | Value | Decision | Comment |
|---|---|---|---|
| CL | 4.153 | ● | ■ Clearance<br>■ High: >15 mL/min/kg; moderate: 5-15 mL/min/kg; low: <5 mL/min/kg |
| $T_{1/2}$ | 0.066 | - | ■ Category 1: long half-life ; Category 0: short half-life;<br>■ long half-life: >3h; short half-life: <3h<br>■ The output value is the probability of having long half-life. |

## 7. Toxicity

| Property | Value | Decision | Comment |
|---|---|---|---|
| hERG Blockers | 0.939 | ● | ■ Category 1: active; Category 0: inactive;<br>■ The output value is the probability of being active. |
| H-HT | 0.997 | ● | ■ Human Hepatotoxicity<br>■ Category 1: H-HT positive(+); Category 0: H-HT negative(-);<br>■ The output value is the probability of being toxic. |
| DILI | 0.983 | ● | ■ Drug Induced Liver Injury.<br>■ Category 1: drugs with a high risk of DILI; Category 0: drugs with no risk of DILI. The output value is the probability of being toxic. |
| AMES Toxicity | 0.949 | ● | ■ Category 1: Ames positive(+); Category 0: Ames negative(-);<br>■ The output value is the probability of being toxic. |
| Rat Oral Acute Toxicity | 0.983 | ● | ■ Category 0: low-toxicity; Category 1: high-toxicity;<br>■ The output value is the probability of being highly toxic. |
| FDAMDD | 0.95 | ● | ■ Maximum Recommended Daily Dose<br>■ Category 1: FDAMDD (+); Category 0: FDAMDD (-)<br>■ The output value is the probability of being positive. |
| Skin Sensiti zation | 0.135 | ● | ■ Category 1: Sensitizer; Category 0: Non-sensitizer;<br>■ The output value is the probability of being sensitizer. |
| Carcinogen city | 0.257 | ● | ■ Category 1: carcinogens; Category 0: non-carcinogens;<br>■ The output value is the probability of being toxic. |
| Eye Corrosion | 0.003 | ● | ■ Category 1: corrosives ; Category 0: noncorrosives<br>■ The output value is the probability of being corrosives. |
| Eye Irritation | 0.009 | ● | ■ Category 1: irritants ; Category 0: nonirritants<br>■ The output value is the probability of being irritants. |

ADMETlab 2.0

Information Classification: General

| | | | |
|---|---|---|---|
| Respiratory Toxicity | 0.935 | ● | ■ Category 1: respiratory toxicants; Category 0: respiratory nontoxicants<br>■ The output value is the probability of being toxic. |

## 8. Environmental toxicity

| Property | Value | Comment |
|---|---|---|
| Bioconcentration Factors | 1.364 | ■ Bioconcentration factors are used for considering secondary poisoning potential and assessing risks to human health via the food chain.<br>■ The unit is –log10[(mg/L)/(1000*MW)] |
| $IGC_{50}$ | 4.431 | ■ Tetrahymena pyriformis 50 percent growth inhibition concentration<br>■ The unit is –log10[(mg/L)/(1000*MW)] |
| $LC_{50}FM$ | 5.971 | ■ 96-hour fathead minnow 50 percent lethal concentration<br>■ The unit is –log10[(mg/L)/(1000*MW)] |
| $LC_{50}DM$ | 6.222 | ■ 48-hour daphnia magna 50 percent lethal concentration<br>■ The unit is –log10[(mg/L)/(1000*MW)] |

## 9. Tox21 pathway

| Property | Value | Decision | Comment |
|---|---|---|---|
| NR-AR | 0.008 | ● | ■ Androgen receptor<br>■ Category 1: actives ; Category 0: inactives;<br>■ The output value is the probability of being active. |
| NR-AR-LBD | 0.719 | ● | ■ Androgen receptor ligand-binding domain<br>■ Category 1: actives ; Category 0: inactives;<br>■ The output value is the probability of being active. |
| NR-AhR | 0.984 | ● | ■ Aryl hydrocarbon receptor<br>■ Category 1: actives ; Category 0: inactives;<br>■ The output value is the probability of being active. |
| NR-Aromatase | 0.968 | ● | ■ Category 1: actives ; Category 0: inactives;<br>■ The output value is the probability of being active. |
| NR-ER | 0.79 | ● | ■ Estrogen receptor<br>■ Category 1: actives ; Category 0: inactives;<br>■ The output value is the probability of being active. |
| NR-ER-LBD | 0.011 | ● | ■ Estrogen receptor ligand-binding domain<br>■ Category 1: actives ; Category 0: inactives;<br>■ The output value is the probability of being active. |
| NR-PPAR-gamma | 0.808 | ● | ■ Peroxisome proliferator-activated receptor gamma<br>■ Category 1: actives ; Category 0: inactives;<br>■ The output value is the probability of being active. |
| SR-ARE | 0.924 | ● | ■ Antioxidant response element<br>■ Category 1: actives ; Category 0: inactives;<br>■ The output value is the probability of being active. |
| SR-ATAD5 | 0.861 | ● | ■ ATPase family AAA domain-containing protein 5<br>■ Category 1: actives ; Category 0: inactives;<br>■ The output value is the probability of being active. |

ADMETlab 2.0

Information Classification: General

| | | | |
|---|---|---|---|
| SR-HSE | 0.123 | ● | ■ Heat shock factor response element<br>■ Category 1: actives ; Category 0: inactives;<br>■ The output value is the probability of being active. |
| SR-MMP | 0.953 | ● | ■ Mitochondrial membrane potential<br>■ Category 1: actives ; Category 0: inactives;<br>■ The output value is the probability of being active. |
| SR-p53 | 0.978 | ● | ■ Category 1: actives ; Category 0: inactives;<br>■ The output value is the probability of being active. |

## 10. Toxicophore Rules

| Property | Value | Comment |
|---|---|---|
| Acute Toxicity Rule | 0 alerts | ■ 20 substructures<br>■ acute toxicity during oral administration |
| Genotoxic Carcinogenicity Rule | 1 alerts | ■ 117 substructures<br>■ carcinogenicity or mutagenicity |
| NonGenotoxic Carcinogenicity Rule | 2 alerts | ■ 23 substructures<br>■ carcinogenicity through nongenotoxic mechanisms |
| Skin Sensitization Rule | 6 alerts | ■ 155 substructures<br>■ skin irritation |
| Aquatic Toxicity Rule | 6 alerts | ■ 99 substructures<br>■ toxicity to liquid(water) |
| NonBiodegradable Rule | 2 alerts | ■ 19 substructures<br>■ non-biodegradable |
| SureChEMBL Rule | 0 alerts | ■ 164 substructures<br>■ MedChem unfriendly status |

ADMETlab 2.0

**Predict ADMET Gefitinib**

ADMETlab 2.0

COc1cc2[nH]cnc(=Nc3ccc(F)c(Cl)c3)c2cc1OCCCN1CCOCC1

## 1. Physicochemical Property

| Property | Value | Comment |
|---|---|---|
| Molecular Weight | 446.15 | Contain hydrogen atoms. Optimal:100~600 |
| Volume | 425.387 | Van der Waals volume |
| Density | 1.049 | Density = MW / Volume |
| nHA | 7 | Number of hydrogen bond acceptors. Optimal:0~12 |
| nHD | 1 | Number of hydrogen bond donors. Optimal:0~7 |
| nRot | 7 | Number of rotatable bonds. Optimal:0~11 |
| nRing | 4 | Number of rings. Optimal:0~6 |
| MaxRing | 10 | Number of atoms in the biggest ring. Optimal:0~18 |
| nHet | 9 | Number of heteroatoms. Optimal:1~15 |
| fChar | 0 | Formal charge. Optimal:-4 ~4 |
| nRig | 24 | Number of rigid bonds. Optimal:0~30 |
| Flexibility | 0.292 | Flexibility = nRot /nRig |
| Stereo Centers | 0 | Optimal: ≤ 2 |
| TPSA | 71.97 | Topological Polar Surface Area. Optimal:0~140 |
| logS | -4.477 | Log of the aqueous solubility. Optimal: -4~0.5 log mol/L |
| logP | 3.822 | Log of the octanol/water partition coefficient. Optimal: 0~3 |
| logD | 3.465 | logP at physiological pH 7.4. Optimal: 1~3 |

## 2. Medicinal Chemistry

| Property | Value | Decision | Comment |
|---|---|---|---|
| QED | 0.56 | 🔴 | ■ A measure of drug-likeness based on the concept of desirability; ■ Attractive: > 0.67; unattractive: 0.49~0.67; too complex: < 0.34 |
| SAscore | 2.823 | 🟢 | ■ Synthetic accessibility score is designed to estimate ease of synthesis of drug-like molecules. ■ SAscore ≥ 6, difficult to synthesize; SAscore <6, easy to synthesize |
| Fsp3 | 0.364 | 🔴 | ■ The number of sp3 hybridized carbons / total carbon count, correlating with melting point and solubility. ■ $Fsp^3$ ≥0.42 is considered a suitable value. |
| MCE-18 | 47.6 | 🟢 | ■ MCE-18 stands for medicinal chemistry evolution. ■ MCE-18≥45 is considered a suitable value. |

ADMETlab 2.0

| | | | |
|---|---|---|---|
| NPscore | -1.395 | - | ■ Natural product-likeness score.<br>■ This score is typically in the range from –5 to 5. The higher the score is, the higher the probability is that the molecule is a NP. |
| Lipinski Rule | Accepted | ● | ■ MW ≤ 500; logP ≤ 5; Hacc ≤ 10; Hdon ≤ 5<br>■ If two properties are out of range, a poor absorption or permeability is possible, one is acceptable. |
| Pfizer Rule | Rejected | ● | logP > 3; TPSA < 75<br>Compounds with a high log P (>3) and low TPSA (<75) are likely to be toxic. |
| GSK Rule | Rejected | ● | ■ MW ≤ 400; logP ≤ 4<br>■ Compounds satisfying the GSK rule may have a more favorable ADMET profile |
| Golden Triangle | Accepted | ● | ■ 200 ≤ MW ≤ 50; -2 ≤ logD ≤ 5<br>■ Compounds satisfying the Golden Triangle rule may have a more favorable ADMET profile. |
| PAINS | 0 alerts | - | Pan Assay Interference Compounds, frequent hitters, Alpha-screen artifacts and reactive compound. |
| ALARM NMR | 4 alerts | - | Thiol reactive compounds. |
| BMS | 0 alerts | - | Undesirable, reactive compounds. |
| Chelator Rule | 0 alerts | - | Chelating compounds. |

## 3. Absorption

| Property | Value | Decision | Comment |
|---|---|---|---|
| Caco-2 Permeability | -4.968 | ● | Optimal: higher than -5.15 Log unit |
| MDCK Permeability | 2.6e-05 | ● | ■ low permeability: < 2 × $10^{-6}$ cm/s<br>■ medium permeability: 2–20 × $10^{-6}$ cm/s<br>■ high passive permeability: > 20 × $10^{-6}$ cm/s |
| Pgp-inhibitor | 0.935 | ● | ■ Category 1: Inhibitor; Category 0: Non-inhibitor;<br>■ The output value is the probability of being Pgp-inhibitor |
| Pgp-substrate | 0.985 | ● | ■ Category 1: substrate; Category 0: Non-substrate;<br>■ The output value is the probability of being Pgp-substrate |
| HIA | 0.002 | ● | ■ Human Intestinal Absorption<br>■ Category 1: HIA+( HIA < 30%); Category 0: HIA-( HIA < 30%); The output value is the probability of being HIA+ |
| $F_{20\%}$ | 0.008 | ● | ■ 20% Bioavailability<br>■ Category 1: $F_{20\%}$+ (bioavailability < 20%); Category 0: $F_{20\%}$- (bioavailability ≥ 20%); The output value is the probability of being $F_{20\%}$+ |

ADMETlab 2.0

| | | | |
|---|---|---|---|
| $F_{30\%}$ | 0.007 | ● | ■ 30% Bioavailability<br>■ Category 1: $F_{30\%}$+ (bioavailability < 30%); Category 0: $F_{30\%}$- (bioavailability ≥ 30%); The output value is the probability of being $F_{30\%}$+ |

## 4. Distribution

| Property | Value | Decision | Comment |
|---|---|---|---|
| PPB | 95.59% | ● | ■ Plasma Protein Binding<br>■ Optimal: < 90%. Drugs with high protein-bound may have a low therapeutic index. |
| VD | 2.581 | ● | ■ Volume Distribution<br>■ Optimal: 0.04-20L/kg |
| BBB Penetration | 0.578 | ● | ■ Blood-Brain Barrier Penetration<br>■ Category 1: BBB+; Category 0: BBB-; The output value is the probability of being BBB+ |
| Fu | 5.057% | ● | ■ The fraction unbound in plasms<br>■ Low: <5%; Middle: 5~20%; High: > 20% |

## 5. Metabolism

| Property | Value | Comment |
|---|---|---|
| CYP1A2 inhibitor | 0.875 | ■ Category 1: Inhibitor; Category 0: Non-inhibitor;<br>■ The output value is the probability of being inhibitor. |
| CYP1A2 substrate | 0.741 | ■ Category 1: Substrate; Category 0: Non-substrate;<br>■ The output value is the probability of being substrate. |
| CYP2C19 inhibitor | 0.684 | ■ Category 1: Inhibitor; Category 0: Non-inhibitor;<br>■ The output value is the probability of being inhibitor. |
| CYP2C19 substrate | 0.742 | ■ Category 1: Substrate; Category 0: Non-substrate;<br>■ The output value is the probability of being substrate. |
| CYP2C9 inhibitor | 0.237 | ■ Category 1: Inhibitor; Category 0: Non-inhibitor;<br>■ The output value is the probability of being inhibitor. |
| CYP2C9 substrate | 0.553 | ■ Category 1: Substrate; Category 0: Non-substrate;<br>■ The output value is the probability of being substrate. |
| CYP2D6 inhibitor | 0.966 | ■ Category 1: Inhibitor; Category 0: Non-inhibitor;<br>■ The output value is the probability of being inhibitor. |
| CYP2D6 substrate | 0.874 | ■ Category 1: Substrate; Category 0: Non-substrate;<br>■ The output value is the probability of being substrate. |
| CYP3A4 inhibitor | 0.909 | ■ Category 1: Inhibitor; Category 0: Non-inhibitor;<br>■ The output value is the probability of being inhibitor. |
| CYP3A4 substrate | 0.814 | ■ Category 1: Substrate; Category 0: Non-substrate;<br>■ The output value is the probability of being substrate. |

ADMETlab 2.0

## 6. Excretion

| Property | Value | Decision | Comment |
|---|---|---|---|
| CL | 8.197 | ● | ■ Clearance<br>■ High: >15 mL/min/kg; moderate: 5-15 mL/min/kg; low: <5 mL/min/kg |
| $T_{1/2}$ | 0.107 | - | ■ Category 1: long half-life ; Category 0: short half-life;<br>■ long half-life: >3h; short half-life: <3h<br>■ The output value is the probability of having long half-life. |

## 7. Toxicity

| Property | Value | Decision | Comment |
|---|---|---|---|
| hERG Blockers | 0.928 | ● | ■ Category 1: active; Category 0: inactive;<br>■ The output value is the probability of being active. |
| H-HT | 0.833 | ● | ■ Human Hepatotoxicity<br>■ Category 1: H-HT positive(+); Category 0: H-HT negative(-);<br>■ The output value is the probability of being toxic. |
| DILI | 0.948 | ● | ■ Drug Induced Liver Injury.<br>■ Category 1: drugs with a high risk of DILI; Category 0: drugs with no risk of DILI. The output value is the probability of being toxic. |
| AMES Toxicity | 0.411 | ● | ■ Category 1: Ames positive(+); Category 0: Ames negative(-);<br>■ The output value is the probability of being toxic. |
| Rat Oral Acute Toxicity | 0.693 | ● | ■ Category 0: low-toxicity; Category 1: high-toxicity;<br>■ The output value is the probability of being highly toxic. |
| FDAMDD | 0.372 | ● | ■ Maximum Recommended Daily Dose<br>■ Category 1: FDAMDD (+); Category 0: FDAMDD (-)<br>■ The output value is the probability of being positive. |
| Skin Sensiti zation | 0.902 | ● | ■ Category 1: Sensitizer; Category 0: Non-sensitizer;<br>■ The output value is the probability of being sensitizer. |
| Carcinogen city | 0.074 | ● | ■ Category 1: carcinogens; Category 0: non-carcinogens;<br>■ The output value is the probability of being toxic. |
| Eye Corrosion | 0.003 | ● | ■ Category 1: corrosives ; Category 0: noncorrosives<br>■ The output value is the probability of being corrosives. |
| Eye Irritation | 0.012 | ● | ■ Category 1: irritants ; Category 0: nonirritants<br>■ The output value is the probability of being irritants. |

ADMETlab 2.0

| | | | |
|---|---|---|---|
| Respiratory Toxicity | 0.972 | ● | ■ Category 1: respiratory toxicants; Category 0: respiratory nontoxicants<br>■ The output value is the probability of being toxic. |

## 8. Environmental toxicity

| Property | Value | Comment |
|---|---|---|
| Bioconcentration Factors | 1.747 | ■ Bioconcentration factors are used for considering secondary poisoning potential and assessing risks to human health via the food chain.<br>■ The unit is –log10[(mg/L)/(1000*MW)] |
| $IGC_{50}$ | 3.869 | ■ Tetrahymena pyriformis 50 percent growth inhibition concentration<br>■ The unit is –log10[(mg/L)/(1000*MW)] |
| $LC_{50}FM$ | 5.068 | ■ 96-hour fathead minnow 50 percent lethal concentration<br>■ The unit is –log10[(mg/L)/(1000*MW)] |
| $LC_{50}DM$ | 6.645 | ■ 48-hour daphnia magna 50 percent lethal concentration<br>■ The unit is –log10[(mg/L)/(1000*MW)] |

## 9. Tox21 pathway

| Property | Value | Decision | Comment |
|---|---|---|---|
| NR-AR | 0.039 | ● | ■ Androgen receptor<br>■ Category 1: actives ; Category 0: inactives;<br>■ The output value is the probability of being active. |
| NR-AR-LBD | 0.007 | ● | ■ Androgen receptor ligand-binding domain<br>■ Category 1: actives ; Category 0: inactives;<br>■ The output value is the probability of being active. |
| NR-AhR | 0.961 | ● | ■ Aryl hydrocarbon receptor<br>■ Category 1: actives ; Category 0: inactives;<br>■ The output value is the probability of being active. |
| NR-Aromatase | 0.95 | ● | ■ Category 1: actives ; Category 0: inactives;<br>■ The output value is the probability of being active. |
| NR-ER | 0.232 | ● | ■ Estrogen receptor<br>■ Category 1: actives ; Category 0: inactives;<br>■ The output value is the probability of being active. |
| NR-ER-LBD | 0.025 | ● | ■ Estrogen receptor ligand-binding domain<br>■ Category 1: actives ; Category 0: inactives;<br>■ The output value is the probability of being active. |
| NR-PPAR-gamma | 0.009 | ● | ■ Peroxisome proliferator-activated receptor gamma<br>■ Category 1: actives ; Category 0: inactives;<br>■ The output value is the probability of being active. |
| SR-ARE | 0.807 | ● | ■ Antioxidant response element<br>■ Category 1: actives ; Category 0: inactives;<br>■ The output value is the probability of being active. |
| SR-ATAD5 | 0.31 | ● | ■ ATPase family AAA domain-containing protein 5<br>■ Category 1: actives ; Category 0: inactives;<br>■ The output value is the probability of being active. |

ADMETlab 2.0

| | | | |
|---|---|---|---|
| SR-HSE | 0.856 | ● | ■ Heat shock factor response element<br>■ Category 1: actives ; Category 0: inactives;<br>■ The output value is the probability of being active. |
| SR-MMP | 0.696 | ● | ■ Mitochondrial membrane potential<br>■ Category 1: actives ; Category 0: inactives;<br>■ The output value is the probability of being active. |
| SR-p53 | 0.829 | ● | ■ Category 1: actives ; Category 0: inactives;<br>■ The output value is the probability of being active. |

## 10. Toxicophore Rules

| Property | Value | Comment |
|---|---|---|
| Acute Toxicity Rule | 0 alerts | ■ 20 substructures<br>■ acute toxicity during oral administration |
| Genotoxic Carcinogenicity Rule | 0 alerts | ■ 117 substructures<br>■ carcinogenicity or mutagenicity |
| NonGenotoxic Carcinogenicity Rule | 0 alerts | ■ 23 substructures<br>■ carcinogenicity through nongenotoxic mechanisms |
| Skin Sensitization Rule | 1 alerts | ■ 155 substructures<br>■ skin irritation |
| Aquatic Toxicity Rule | 1 alerts | ■ 99 substructures<br>■ toxicity to liquid(water) |
| NonBiodegradable Rule | 2 alerts | ■ 19 substructures<br>■ non-biodegradable |
| SureChEMBL Rule | 0 alerts | ■ 164 substructures<br>■ MedChem unfriendly status |

ADMETlab 2.0
